# Mapping the global end-of-life vehicles to scale up circular economy

**DOI:** 10.1016/j.isci.2026.117216

**Published:** 2026-09-03

**Authors:** Xianlai Zeng, Moisés Gómez, Zhengyang Zhang, Xianyang Zeng, Jinhui Li, Kazuyo Matsubae

**Affiliations:** 1 State Key Laboratory of Iron and Steel Industry Environmental Protection, School of Environment, Tsinghua University, Beijing 100084, China; 2 Graduate School of Environmental Studies, Tohoku University, Sendai 980-8572, Japan; 3 School of Mathematical Sciences, Liaocheng University, Liaocheng, Shandong 252000, China

**Keywords:** circularity, vehicle, ELV, recycling, sustainability

## Abstract

The vehicle industry is significantly improving our quality of life and promoting economic development. However, the large number of end-of-life vehicles (ELVs) deserves more attention since many valuable resources are embodied in them. This work predicts ELV generation for both commercial vehicles and passenger cars in 144 countries. Our results show that while typical developed countries like Japan, Germany, and France peaked their ELV generation in the 2010s, China has experienced a dramatic increase, with nearly a 45-fold increase from 2000 to 2025, and since 2020 become the largest generator of ELVs over the US. With the rapid transition toward electric vehicles, its recycling through a circular economy is facing a new challenge and therefore requires more specific efforts. Key findings call for global efforts to improve the ELV circular economy through the full implementation of a circular economy so that both resource concerns and associated environmental impacts from ELVs can be mitigated.

## Highlights

• ELV generation is mapped for 144 countries from 2000 to 2025
• China’s ELV generation rose 45-fold and became the world’s largest
• ELV generation peaked in many developed countries during the 2010s
• Vehicle electrification requires tailored circular recycling systems

## Graphical abstract

**Figure:**
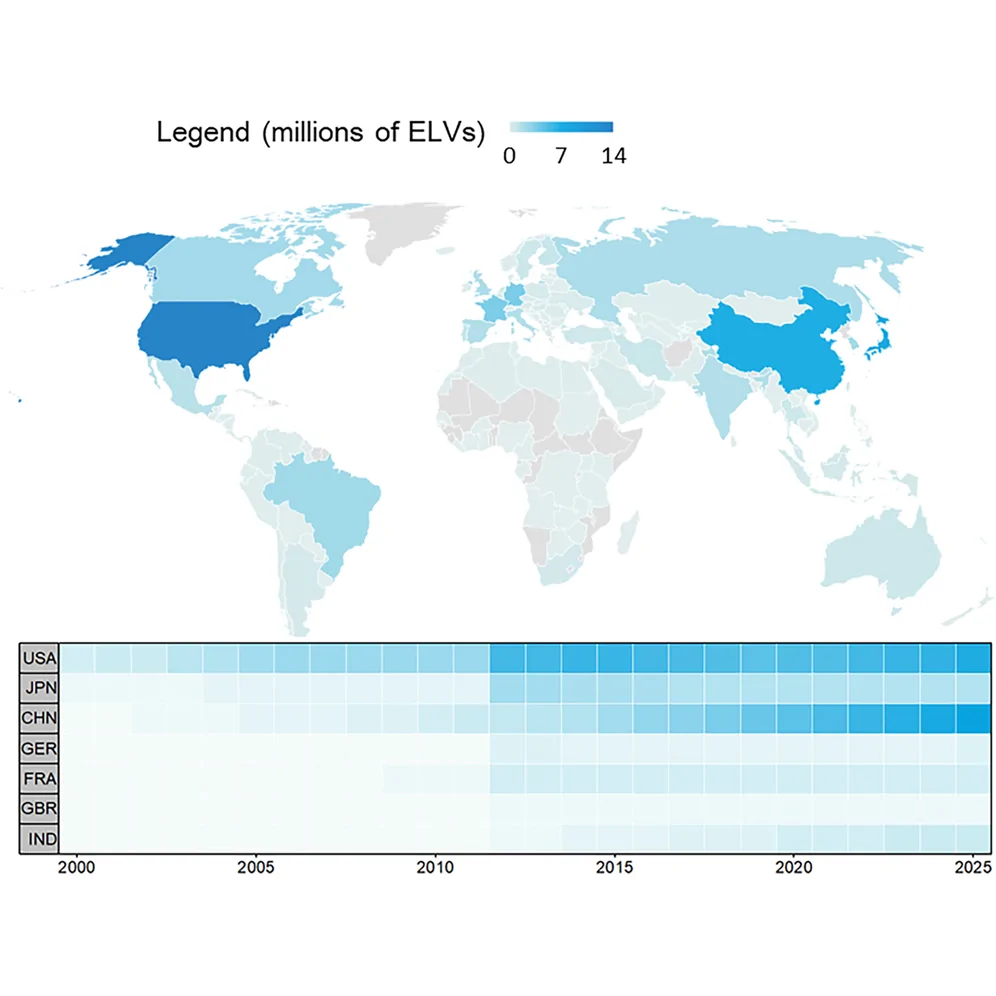

## Teaser

Environmental assessment; Computer modeling; Economics

## Introduction

Vehicle has greatly improved our quality of life and business development for over a century.^1^ This industry has created millions of jobs globally and led to the rapid development of many related sectors, such as rubber, steel and iron, plastics, textiles, non-ferrous metals, electronics, and glass. Meanwhile, a circular economy for resource efficiency and waste recycling seeks to achieve the win-win objectives of environmental protection and resource conservation.^2^ It is reshaping some industries and business performance across all the supply chains. Since 2020s, the circular economy of the vehicle industry has become an increasingly important concern.^3^^,^^4^

The 1950s saw the United States (US) become an early leader in global vehicle production, responsible for about 80% of the global share. By the 1980s, this same proportion of vehicle manufacturing was collectively shared across the key industrialized nations, including the US, Japan, Germany, France, and the United Kingdom (UK). However, this scenario shifted when China increased its vehicle production drastically and became the world’s largest producer in 2009. In 2021, China produced over 21.4 million (M) units, followed by Japan and India with 6.62 and 3.63 million units, respectively. By December 2023, China’s vehicle ownership had reached 435 million units, consisting of 336 million traditional fossil fuel-based vehicles and 20.41 million electric vehicles.^5^ As China’s production of vehicles has increased, so too has the global demand for electric vehicles in response to worldwide climate change efforts. Given the substantial contribution of traditional vehicles to total carbon dioxide (CO_2_) emissions, the transition toward electric vehicles has been widely accepted as desirable and this trend is reflected in global production.^6^^,^^7^^,^^8^ For instance, passenger cars (PCs) account for approximately 12% of the total CO_2_ emissions in the European Union (EU).^1^^,^^9^

Once a vehicle has reached the end of its life span, however, it goes from wonderland to wasteland.^10^ A significant number of resources, such as metals and polymers, end up as waste in these end-of-life vehicles (ELVs) (excluding selling to the secondary market^11^). Moreover, material production contributes to almost a quarter of the global greenhouse gas (GHG) emissions, implying an urgent recycling need from these ELVs.^12^^,^^13^ Especially, with the depletion and degradation of available geological minerals, recycling ELVs through a circular economy can provide more anthropogenic minerals to support industrial development. However, there are several challenges to recoup these valuable resources, such as obtaining accurate data concerning ELVs, including their geographic distribution, realized lifetime, and usage trends.

Academically, the volume of ELVs has been estimated in several specific countries, such as China,^14^^,^^15^^,^^16^^,^^17^^,^^18^^,^^19^ Japan,^20^^,^^21^ India,^22^ Malaysia,^23^ and the US,^24^^,^^25^ but these studies cannot present a global picture of ELVs. Also, policy impacts on vehicle lifespans have not yet been considered. Since the treatment of ELVs is a global challenge and some countries do not have effective ELV collection and treatment facilities, international cooperation is critical so that global efforts can be made to improve the overall recycling efficiency of ELVs. Therefore, considering the main type of vehicle (Table S1), this study aims to estimate the total generation of ELVs and uncover the key features of ELV evolution so that valuable insights can be obtained for preparing appropriate policies to effectively manage ELVs.

## Results

### ELV generation in major countries and continents

The lifespan of different vehicles is determined by the robustness of the vehicle as well as consumers’ behaviors. The former is influenced by vehicle quality and production year, while the latter is mostly driven by maintenance and usage (i.e., total miles driven). For instance, vehicles manufactured in Germany are normally of higher quality and have longer service spans, while vehicles manufactured in developing countries are not very reliable and have to be frequently maintained and repaired in order to extend their service spans. Even the same brand vehicle may have different life spans in different countries due to different transportation infrastructure. By considering these factors, we collected all relevant vehicle life-span data from 144 countries and estimated their scales and shapes by using the Weibull distribution function (for details, see Figures S2 and S3; Tables S5–S11).

**Figure 1:**
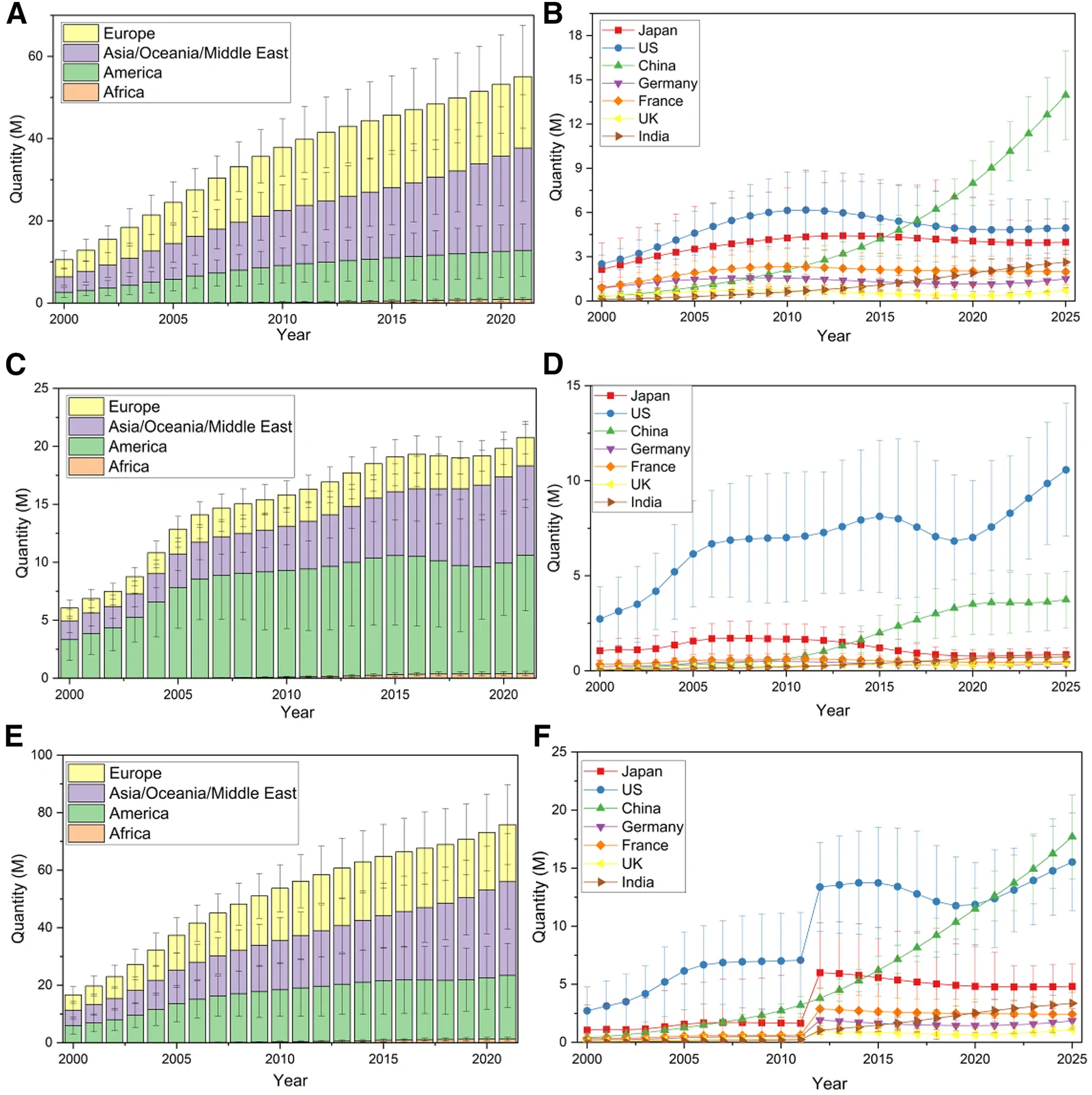
Estimated generation of ELVs from 2000 to 2025 in main countries and continents (A) EoL PCs in the main continents. (B) EoL PCs in selected countries. (C) EoL CVs in the main continents. (D) EoL CVs in selected countries. (E) ELVs in the main continents. (F) ELVs in selected countries. The error bars are derived from the uncertainty analysis in Tables S12–S15, and the unit is “millions of vehicles.”

In light of STAR Methods, global generation of end-of-life (EoL) PCs increased from 10.5 million in 2000 to 37.9 M in 2010, and then jumped to 51.5 M in 2019 (Figures 1 and S4). In 2000, Europe was the largest generator of such vehicles, followed by Asia/Oceania/Middle East, America, and Africa. In 2016, Asia/Oceania/Middle East replaced Europe as the largest generator of such vehicles. The consumption of PCs peaked in Europe and America around 2017. Regarding individual countries, Japan, Germany, France, the US, and the UK peaked in 2011, 2012, 2013, 2019, and 2019, respectively. However, the demand for PCs is still increasing, particularly in developing nations. For instance, China surpassed Japan in 2019 to become the largest generator of EoL PCs, followed by the US, Japan, Germany, France, the UK, and India (Figure 1B). The generation amount of EoL commercial vehicles (CVs) is lower than that of EoL PCs. Similarly, the global generation of EoL CVs increased quickly, with a 3-fold increase since 2000 and peaked at 19.3 M in 2016 (Figure 1C). America is the largest generator of EoL CVs, followed by Asia/Oceania/Middle East, Europe, and Africa. However, Asia/Oceania/Middle East experienced a rapid increase in EoL CVs, mainly owing to China’s substantial rise of EoL CV production. With regard to individual countries, the US has been the largest generator of EoL CVs in recent times (based on data from 2000 to 2024), with an annual generation of 6 million, which is larger than the combined total of Japan, China, Germany, France, the UK, and India (Figure 1D).

**Figure 2:**
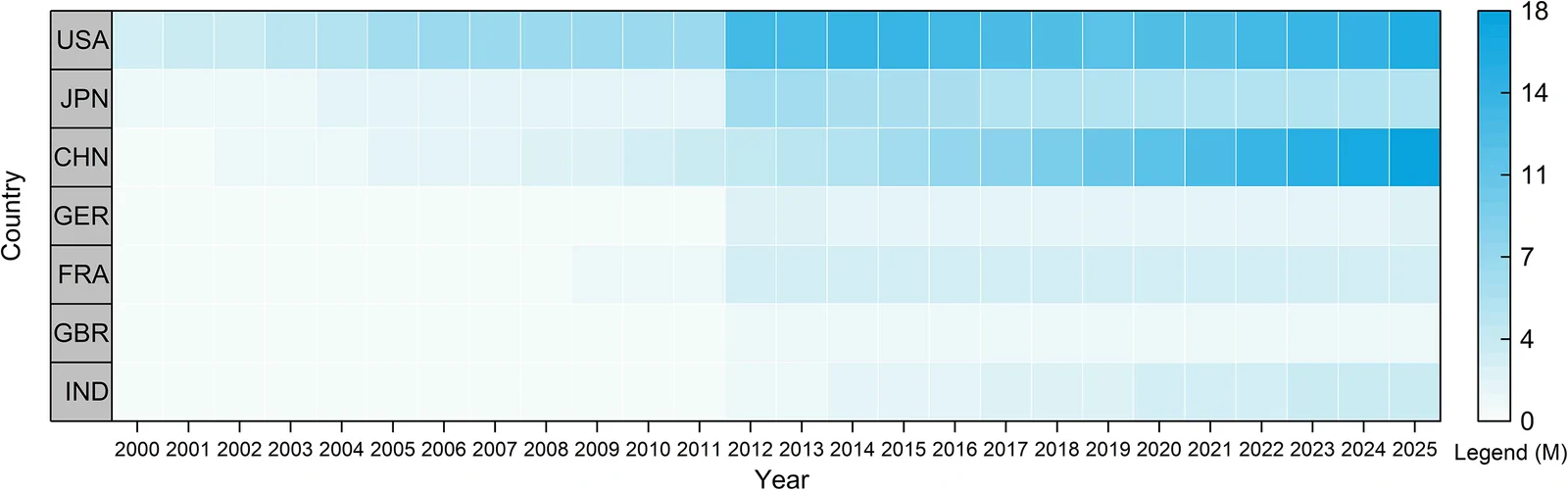
Evolution of ELV generation in main countries The detailed abbreviations are provided in Table S2, and the unit is “millions of vehicles.”

Total ELV generation has increased steadily since 2000 in the region of Asia/Oceania/Middle East where it accounted for 40% of global ELV generation in 2019. Meanwhile, Africa’s share of global ELV generation was just 2% in 2019 (Figure 1E). Typical developed countries, such as the US, Japan, Germany, and France peaked in their ELV generation in 2016, 2011, 2012, and 2012, respectively. With regard to China, this figure jumped from 0.4 M in 2000 to 11.5 M in 2020 and 17.7 M in 2025, meaning a 45-fold increase and an annual increase rate of 170%. Its discarding rate, the ratio of ELVs divided by production amount, is much less than the 6% average reported by industrial nations.^26^ India has also experienced a significant increase in ELV generation and is now leapfrogging over Germany and France (Figure 2). According to the Automobile Recycling Association, more than 14 M vehicles reach the end of their service lives each year in the US, and 80% of these total materials can be recycled or reused.

### Overall picture of ELV generation and its evolution

**Figure 3:**
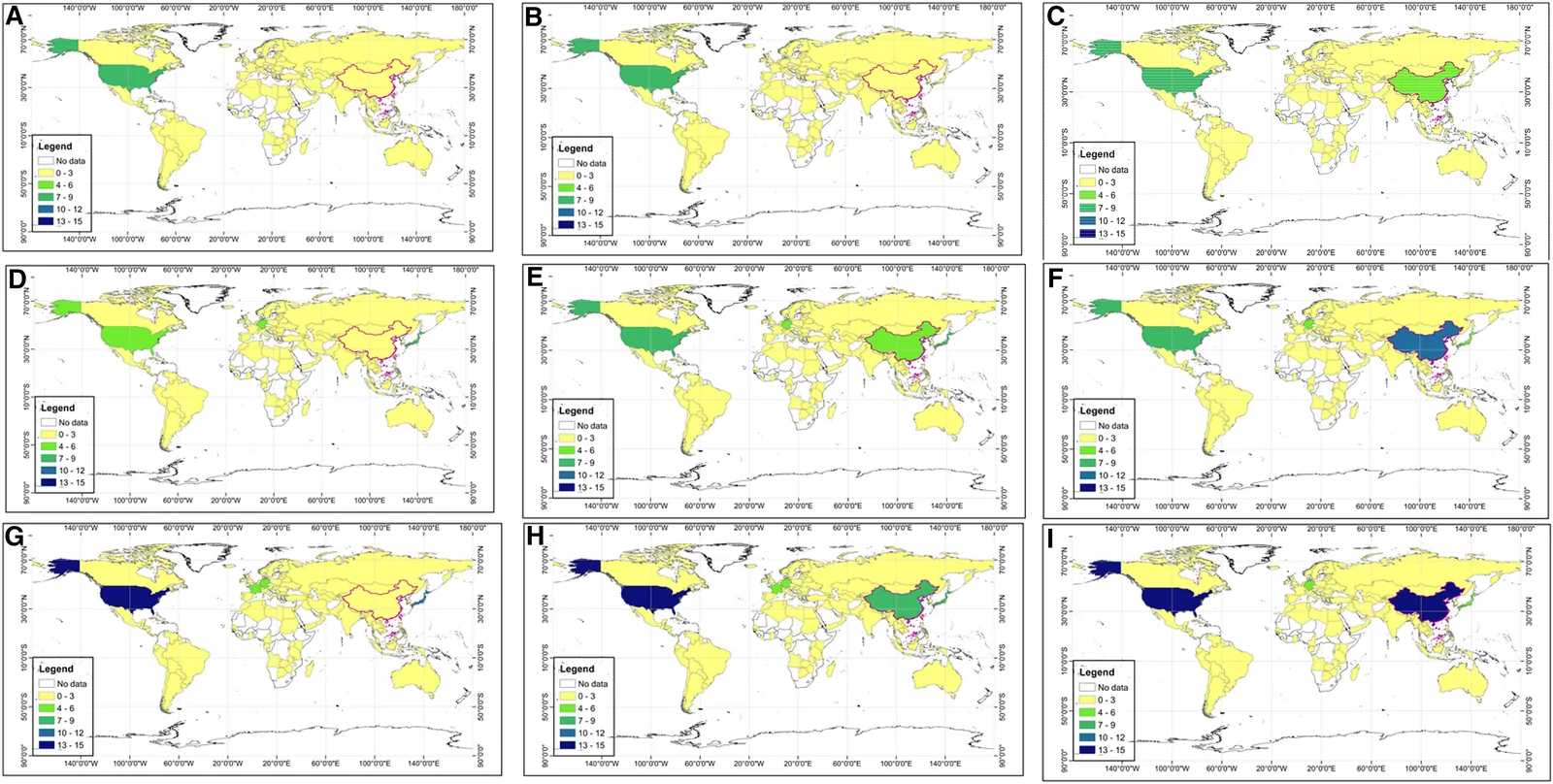
Global ELV generation (A) CVs in 2010; (B) CVs in 2015; (C) CVs in 2020; (D) PCs in 2010; (E) PCs in 2015; (F) PCs in 2020; (G) ELVs in 2010; (H) ELVs in 2015; (I) ELVs in 2020. The unit is “millions of vehicles.”

In total, 144 countries and regions were selected to map the generation of their ELVs for the years of 2010, 2015, and 2020 (Figure 3). In most countries and regions, the generation of EoL CVs is less than 9 M (Figures 3A–3C). The US, Japan, and Canada generated the most EoL CVs. However, several emerging economies such as China, India, and Brazil have quickly increased their EoL CVs. Taking China as an example, it is estimated that there were between 1 and 3 M EoL CVs in China in 2010. However, this number jumped to 4–6 million in 2020— the highest increase in EoL CVs observed in the time period explored in this study. With regard to PCs (Figures 3D–3F), over 200 M PCs were retired in China, the US, Japan, Germany, and France. Among these countries, China has emerged as the largest generator of EoL PCs. In 2020 alone, the retired PCs reached 10–12 M in China, significantly higher than the 1 M retired during the previous decade. In summary, advanced economies and populous developing countries are not only the major consumers of PCs but also the leading generators of EoL PCs. These countries tend to upgrade their vehicles more frequently, resulting in shorter lifespans of their vehicles and a higher pressure to deal with their ELVs.

From a regional point of view, most African countries generate fewer than 0.4 million units ELVs, which is the lowest number among the countries under consideration (Figures 3G–3I). Most developed countries and regions did not increase their ELVs in the last decade, meaning that that they had already peaked in their ELV generation in the 2010s. The top two generators, namely the US and China, generate over 10 million units each, annually. China has become the world’s largest automobile producer and generator of ELVs, with an estimated 13–17 M ELVs reaching the end of their service spans each year. As such, with rapid economic development, many other developing countries are now consuming more vehicles, implying a great increase of such ELVs in the near future.

## Discussion

While the production, consumption, and regulation of vehicles are well-defined in a specific region, the two parameters in the lifespan function vary from country to country and year to year. Any potential errors in the obtained results are most likely to be derived from the evolution of these two parameters. A large-scale uncertainty analysis (see STAR Methods) was conducted in this study to validate the presented ELV generation numbers. In total, 584 counts of uncertainty analysis on EoL CVs and PCs from 2000 to 2025 were carried out for the four main continents and seven main countries under consideration. All distributions (see Tables S12–S15) demonstrated a satisfactory convergence in the peak and range of probabilities for the ELV generation results. Finally, the obtained results of ELV generation were well validated by the estimating process.

**Figure 4:**
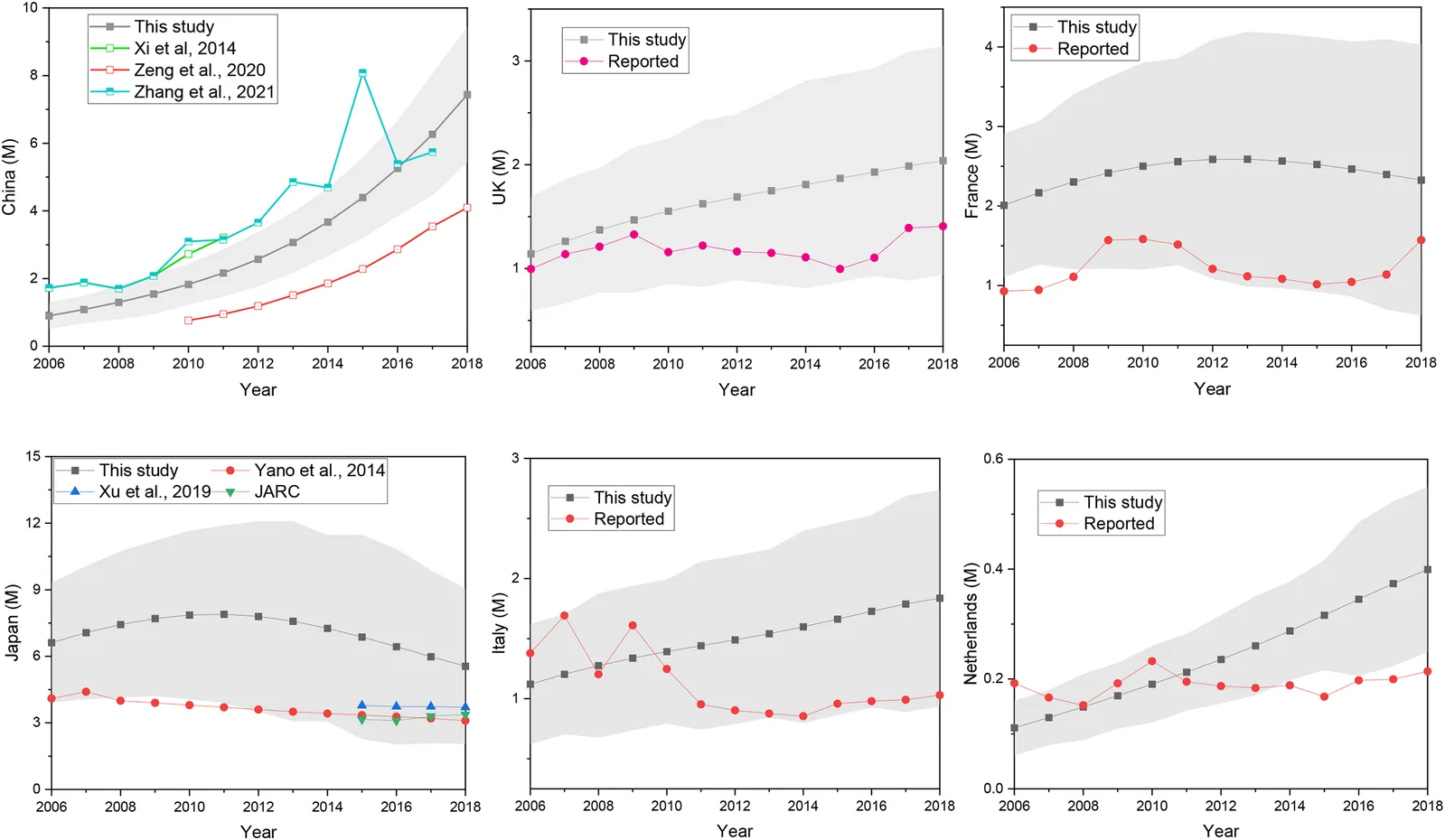
Comparison between this study and other researches on PCs The shaded areas are derived from the uncertainty analysis in Tables S13 and S15, and the unit is “millions of vehicles.”

We also compared the obtained results with the previously reported data (Figure 4; Table S16). Close to 5.3 M PCs and light goods vehicles (light goods road vehicles with a gross vehicle weight of not more than 3,500 kg, designed exclusively or primarily, to carry goods, e.g., vans and pick-ups) were scrapped in the EU in 2017. In 2017, scrapped PCs and light goods vehicles in the EU weighed a total of 5.7 million tonnes; 94% of parts and materials were reused and recovered, while 88% of parts and materials were reused and recycled. This comparison well validates the findings in this study.

We first analyzed historical sales and production data for vehicles in 144 countries and projected ELV generation for both CVs and PCs. The obtained results show that, while typical developed countries, such as the US, Japan, Germany, and France peaked in their ELV generation in the 2010s, China has experienced a dramatic increase in generation, with nearly a 50-fold increase from 2000 to 2025, making it the largest generator of ELVs in 2021. Additionally, although there is still a considerable number of secondhand vehicles exported outside countries and regions, the importation and exportation of ELVs or reused vehicles are not considered in the results.^27^ The closed-loop recycling of ELVs has great potential to reduce the demand for various virgin materials, which in turn contributes to a more sustainable transition toward a circular economy vision.^28^^,^^29^

With the rapid promotion of electric vehicle integration, more attention should be paid to their recycling through a circular economy, since they consist of different materials and as such should be treated differently. Many countries have released their carbon neutrality targets, in which electric vehicles play a key role. Moreover, the projected population growth and the increasing purchasing power in many low- and middle-income countries will lead to a significant increase in such vehicles, especially in China, India, and Africa, which in turn will lead to more ELVs. In these developing countries, ELV management, however, is more generally shaped by institutional fragmentation, infrastructure gaps, and the distinct role of informal recycling markets.^30^ Consequently, it is critical to initiate more collaborative efforts between vehicle manufacturers, recycling facilities, governments, and consumers.^31^ In July 2026, China released “The National Plan of Circular Economy in the 15^th^ Five-Year period,” highlighting the potential policy revision on ELV recycling and its component remanufacturing processes.

Different countries vary greatly in their approach to the circular economy for managing ELVs.^32^^,^^33^^,^^34^ Common measures include easily disassembled design for various vehicles, mandatory ELV reporting, collection, and sorting systems, the incubation of more advanced remanufacturing and refurbishing technologies, capacity-building activities to improve consumers’ awareness, and carbon labeling programs for recycled materials. In addition, international cooperation should be encouraged, so that developing countries can learn from the experiences of developed countries in scaling up the circular economy and a more sustainable future for ELVs can be achieved globally.

### Limitations of the study

Although this study provides a global assessment of ELV generation across 144 countries, several limitations should be considered. First, estimates depend on the completeness and consistency of national vehicle production, sales, and lifespan data. For countries with limited observations, Weibull parameters were derived from the best available sources or comparable cases, and changes in regulations, maintenance practices, infrastructure, and consumer behavior may cause actual vehicle lifetimes to differ from modeled values. Second, the analysis treats national vehicle systems largely as closed and does not explicitly track international flows of second-hand vehicles, cross-border ELV trade, temporary deregistration, or vehicles retained in informal use, which may shift the location and timing of retirement. Third, PCs and CVs are represented at aggregated levels without differentiating powertrain, vehicle size, material composition, or formal and informal collection pathways. Therefore, the results estimate the scale and geographic distribution of ELV generation rather than recoverable material quantities or realized recycling performance. Future studies should integrate vehicle-registry and trade data, powertrain-specific stocks, and country-level collection and treatment information.

## Resource availability

### Lead contact

Requests for further information and resources should be directed to and will be fulfilled by the lead contact, Xianlai Zeng (xlzeng@tsinghua.edu.cn).

### Materials availability

This study did not generate new unique reagents.

### Data and code availability

• The data used in this study are all available from public resources that have been appropriately cited within the manuscript or given in the online supplemental information.
• This research did not generate any custom code.
• Any additional information required to reanalyze the data is available from the lead contact upon request.

## Acknowledgments

This research was funded by the 10.13039/501100001809National Natural Science Foundation of China (92462301 and 92562305) and the 10.13039/501100012166National Key R&D Program of China (2024YFC3909400) and supported by the Tsinghua University Initiative Scientific Research Program and the Tsinghua University -Toyota Research Center. We also appreciate Prof. Yong Geng (Shanghai Jiao Tong University) and Ms. Alexandra A. Sourakov (Tsinghua University) for their valuable comments.

## Author contributions

Xianlai Zeng, conceptualization, methodology, investigation, software, visualization, funding acquisition, supervision, project administration, writing – original draft, and writing – review and editing; M.G., data curation and writing – review and editing; Z.Z. and Xianyang Zeng, formal analysis and writing – review and editing; J.L. and K.M., project administration and writing – review and editing.

## Declaration of interests

The authors declare no competing interests.

## STAR★Methods

### Key resources table

**Table:** 

REAGENT or RESOURCE	SOURCE	IDENTIFIER
**Deposited data**		

Vehicle selling amount	International Organization of Motor Vehicle Manufacturers	https://www.oica.net/category/production-statistics/2023-statistics/ https://www.theglobaleconomy.com/
World Motor Vehicle Production	The Bureau of Transportation Statistics of US	https://www.bts.gov/archive/publications/national%20transportation_statistics/table_01_23
Vehicle consumption	China Association of Automobile Producers	http://www.caam.org.cn/zongheshuju/20180510/1605217108.html

**Software**		

ArcGIS 10.8	ESRI	https://www.esri.com/en-us/home
Origin 2026	OriginLab	https://www.originlab.com/
Excel 2024	Microsoft Office	https://www.microsoftstore.com.cn/software/office
Crystal Ball 10	Oracle	https://www.oracle.com/applications/crystalball/

### Method details

#### Vehicle production and consumption data

For the purposes of this research question, “vehicle” encompasses the following types: car, multi-purpose vehicle, sport utility vehicle, minivan, pickup, cross passenger car, and light commercial vehicle, which can be broadly categorized into two main types, as “commercial vehicle” (CV) and “passenger car” (PC) [see Table S1]. A dataset was assembled of all available historical sales and production numbers of vehicles in the world: four big continents and 144 countries or regions in 1999–2017 (Table S3), production of vehicle in 1960–1988 (Table S4), production and consumption in 2005–2015 (Figure S1), selling amount in 1990–2024, and PC production in the U.S., Japan, and Germany in 1960–1988 (Table S5).

#### Weibull distribution function

While ELVs are generated through the consumption of vehicles, the lifespan of vehicles can also be affected by local or national regulations. For instance, the maximum lifespan of taxi car should be no more than eight years in China. Previous studies indicate that the Weibull distribution function can appropriately represent the durable product lifespan. In applying this model to ELVs, the regulated lifespan should also be considered and embedded into the conventional Weibull distribution function. Its revised probability distribution function (PDF) is illustrated as

(Equation 1) $$f\left(x\right)=\left\{ \begin{array}{cc} 0 & x>L\\ e^{-{\left(x/\eta \right)}^{\beta }} & x=L\\ \frac{\beta }{\eta }{\left(\frac{x}{\eta }\right)}^{\beta -1}e^{-{\left(x/\eta \right)}^{\beta }} & 0\leq x<L\\ 0 & x<0 \end{array}\right.$$

The cumulative distribution function (CDF) for the revised Weibull distribution is

(Equation 2) $$F\left(x\right)=1-e^{-{\left(x/\eta \right)}^{\beta }}$$

where *η* is the scale parameter as the lifespan (*η* > 0), *β* is the shape parameter (*β* > 0), and L is the regulated maximum lifespan of vehicle.

#### Mathematical model for ELV generation

Based on net product production (as inflow) and the lifespan function, the annual ELV generation (as outflow) can be determined by Equation 3:

(Equation 3) $$D\left(2000+x\right)=\int_{0}^{n}f\left(2000+x\right)P\left(2000+x\right)dx=\sum_{i=1}^{2}\left[P_{i}\left(2000\right)\times f_{i}\left(x\right)+P_{i}\left(2001\right)\times f_{i}\left(x-1\right)+\cdots +P_{i}\left(x+1999\right)\times f_{i}\left(1\right)\right]$$

where *x* is the year; *D*(*x*) is the total weight of ELV generation in the year *x* (in million, M); *i* is the *i*^th^ category of product; *n* is the total number items in the vehicle category; *P*_*i*_(20yy) is the net weight of the *i*^th^ product in the year 20yy (in M); and *f*_*i*_(x-2000) is the obsolescence rate of the *i*^th^ product since the year 2000.

### Quantification and statistical analysis

#### Uncertainty analysis

Based on all the equations, the uncertainty of the ELV generation is not only caused by the variables, including the vehicle selling or consumption, but also on Weibull function parameters. Normal distribution and 90% errors are considered to support this analysis. Here we also consider their interactive influences and adopt a Monte Carlo simulation (10^5^ iterations) to examine the uncertainty of the obtained results. The software with Excel 2024 and Crystal Ball 10 was adopted to finalize this work.
