# Supplementary material for: Mapping the global end-of-life vehicles to scale up circular economy
Source: iScience. 2026 Sep 3;29(9):117216. doi: 10.1016/j.isci.2026.117216 (PMC13571730; doi:10.1016/j.isci.2026.117216)
Supplement: Supplementary file 1 [file mmc1.pdf]

iScience, Volume *29*

Supplemental information

Mapping the global end-of-life vehicles

to scale up circular economy

Xianlai Zeng (曾现来), Moisés Gómez, Zhengyang Zhang (张政阳), Xianyang Zeng (曾现洋), Jinhui Li (李金惠), and Kazuyo Matsubae

Table S1: Vehicle type definitions

Type	Definition		Picture	Typical weight (t) [1-4]
Passenger cars 乘用车	Passenger cars are motor vehicles with at least four wheels, used for the transport of passengers, and comprising no more than eight seats in addition to the driver's seat (Does not include minivans, pickups, and sport utility vehicles).		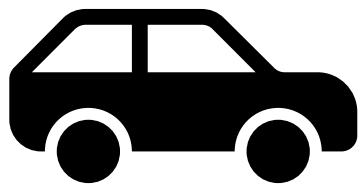	1.3, [0.5-2.2]
Commercial vehicles 商用车	Commercial vehicles are motor vehicles designed primarily for transporting goods or passengers for commercial purposes, including trucks, buses, vans, and specialized vehicles.	Light commercial vehicles	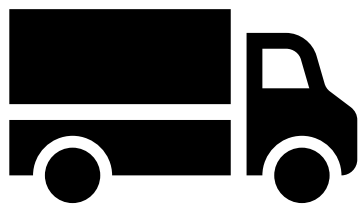	3.14, [1.8-6]
		Heavy trucks	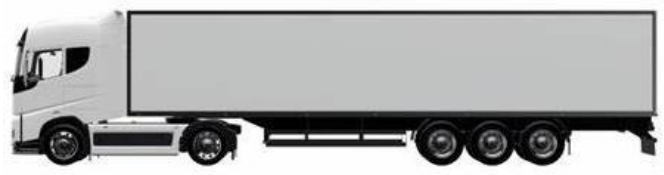	16, [14-18]
		Buses and coaches	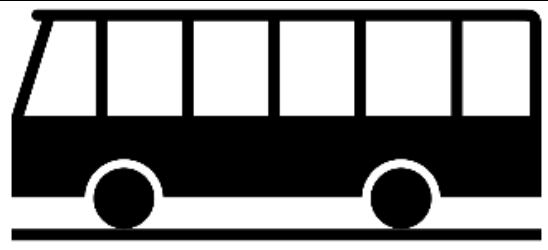	11, [8-14]

Table S2: Abbreviations, Related to Figure 2

CO_2	carbon dioxide	DE	Germany
CDF	cumulative distribution function	ET	Estonia
CV	commercial vehicle	IE	Ireland
ELVs	end-of-life vehicles	GR	Greece
EoL	end-of-Life	ES	Spain
EU	Europe Union	FR	France
GHG	global greenhouse gas	CI	Croatia
MFA	material flow analysis	IT	Italy
PC	passenger car	CY	Cyprus
PDF	probability distribution function	LV	Latvia
M	million	LT	Lithuania
LMI	lower middle-income country	LU	Luxembourg
HIC	high income country	HU	Hungary
UMI	upper middle-income country	MT	Malta
US	United States of America	NL	Netherlands
CA	Canada	AT	Austria
CN	China mainland	PL	Poland
JP	Japan	PT	Portugal
IN	India	RO	Romania
KR	Korea	SRB	Serbia
SG	Singapore	SI	Slovenia
TW	Taiwan, China	SK	Slovakia
GB	United Kingdom	FI	Finland
BE	Belgium	SE	Sweden
BG	Bulgaria	IS	Iceland
CZ	Czechia	LI	Liechtenstein
DK	Denmark	NO	Norway

Table S3: Sales and production of vehicles from 1999 to 2023

(A) Sales and production of vehicles

Year	1999	2000	2001	2002	2003	2004	2005	2006	2007	2008	2009	2010	2011	2012	2013	2014	2015	2016	2017
**Europe**	**2E+07**	**2E+07**	**2E+07**	**2E+07**	**2.1E+07**	**2.1E+07**	**2.1E+07**	**2.2E+07**	**2.3E+07**	**2.2E+07**	**1.9E+07**	**1.9E+07**	**2E+07**	**1.9E+07**	**1.8E+07**	**1.9E+07**	**1.9E+07**	**2E+07**	**2.1E+07**
**Austria**		18			248718	248718	345593	347387	339691	336000	350429	362564	396655	374829	357892	342215	349597	374545	403580
**Belgium**	34375	38926	42320	41461	900273	900273	551501	594454	602365	612692	536260	607497	643511	550519	547139	545255	571524	617854	634111
**Denmark**							211159	226339	226214	191169	131788	173566	198518	199147	210839	221713	245484	265386	263361
**Finland**	3180193	3348361	3628418	3692738	10501	10510	168121	166673	147842	160998	103016	126396	144425	126505	117368	120113	123483	136430	138375
**France**	5687692	5526615	5691677	5469309	3665990	3665990	2598183	2544165	2629186	2614829	2718599	2708884	2687052	2331731	2207373	2210927	2345092	2478472	2604942
**Germany**	1701256	1738315	1579696	1426947	5569954	5569954	3614886	3772394	3482279	3425039	4049353	3198416	3508454	3394002	3257718	3356718	3539825	3708867	3811246
**Greece**							295266	294060	306875	292865	237118	153842	104682	62518	62570	76665	82044	85008	95363
**Iceland**							20870	20198	19305	10579	2471	3395	5471	8507	8022	10629	15605	20735	24059
**Ireland**							213706	225723	236353	185620	68031	99986	102443	91728	87096	114985	150680	177719	159236
**Italy**	307220	267319	238943	231291	1141944	1142105	2495436	2606375	2777175	2421918	2357443	2164153	1942949	1545764	1420814	1493008	1726079	2050292	2190403
**Luxembourg**							53122	55512	56647	58405	51462	53993	55015	55049	50886	54322	51607	56369	58965
**Netherlands**	252290	246724	239719	250832	247503	247503	545983	568717	601534	604135	451907	542312	627757	571893	481116	450346	521178	469410	508371
**Norway**							152588	158407	182203	153247	127437	162354	180313	176909	180042	180273	190106	197991	201895
**Portugal**	2852389	3032874	2849888	2855239	226728	226728	278470	265174	276606	275127	203760	272754	191362	113435	126689	172357	213654	247343	264904
**Spain**	250742	301343	289147	276193	3011010	3012174	1959488	1953134	1939298	1362586	1074222	1114119	931404	790991	822950	1029782	1277059	1347344	1451089
**Sweden**	1973519	1813894	1685238	1821084	338756	340270	316108	329960	358717	301459	247513	334134	359066	326441	313067	352467	396693	431818	442835
**Switzerland**							293472	298697	315402	321326	294699	324779	355256	366273	343718	338404	362650	355882	355910
**UK**	60	141			1856049	1856539	2828127	2734360	2799619	2485258	2222542	2293576	2249483	2333763	2595713	2843025	3061406	3123755	2955182
**Bulgaria**							34940	45300	55336	57927	26813	20082	23529	22770	22966	27472	28053	31260	37347
**Croatia**							79675	89092	94695	100415	50859	42031	45935	35654	33819	40196	43668	53929	60795
**Cyprus**							20986	22383	27517	27533	18419	17920	15958	11563	7949	9447	11834	14559	15078
**Czech**		72	72		448360	448360	175923	183631	207038	215451	186790	186648	194945	193795	185939	215594	260070	291008	302109
**Estonia**							23511	30754	37480	29000	11489	12203	20346	23073	23383	24845	24921	27401	30420
**Hungary**	376261	455492	465268	447077	122666	122666	219461	209280	193581	174837	70808	55221	60993	68168	72975	88719	100933	123807	141814
**Latvia**							16135	21744	31090	28885	8918	10369	17929	14497	14480	16261	17764	20345	20712
**Lithuania**							19639	30443	39690	24085	6244	7534	14312	16669	17586	19039	23249	29380	36180
**Malta**							7229	7320	7119	6351	6495	5004	7030	6475	6312	7034	7929	8157	8586
**Poland**	128186	137398	144313	141513	593779	601000	271098	278869	353774	396603	321032	366577	338781	329799	353197	392476	432439	504550	576144
**Romania**	126831	181783	182003	225680	122155	122185	248462	282985	356577	325867	133561	104090	95508	80723	71016	100336	120591	142020	159808
**Slovakia**	574834	504972	347875	309733	223542	223542	75033	83519	89094	102378	92761	73820	77904	78189	75206	81960	90091	100600	108672
**Slovenia**	106897	78165	68761	79451	131646	131646	67856	67836	78398	81631	63286	66871	67451	57042	59782	61934	68973	76213	86142

**Albania**							1000	1000	1800	1800	1200	1800	2260	2500	2490	2750	2750	2750	3365
**Armenia**							11430	12450	6480	4400	4400	4400	4400	5400	5390	5600	2500	2700	2977
**Belarus**	13226	14634	16425	15400	20290	20290	13000	18000	27000	32000	17000	19000	20000	24500	24000	25800	25800	22500	25978
**Bosnia**							11500	11600	16700	14400	13205	11000	11000	10739	10520	9500	9300	10200	13347
**Georgia**							2700	3200	7300	9000	2000	5000	4000	5300	5300	5500	4200	4300	4556
**Macedonia**							10060	10790	11200	11500	6800	9500	9400	5650	2800	3500	3900	4300	3863
**Moldavia**							4550	5570	7610	11500	4200	5200	5900	5300	5400	4100	3800	4500	6022
**Russia**	1169708	1205581	1250692	1219634	1385434	1386127	1806625	2244840	2898032	3222346	1597457	2107135	2901612	3141551	2998650	2592396	1440923	1404464	1602270
**Serbia**	118132	122949	116072	126661	15194	15194	24516	30761	39947	44255	33500	31242	25321	26700	26800	27800	25000	27600	30924
**Turkey**	**297862**	**430947**	**270685**	**346565**	**823408**	**823408**	715212	617838	594762	494355	557126	760913	864439	817620	893124	807486	1011194	1007857	980394
**Ukraine**	19180	31255	31824	53823	186851	186890	276697	391301	525680	640968	181750	188408	227250	259586	232894	105957	52341	75209	94694
**America**	**1.9E+07**	**2E+07**	**1.8E+07**	**1.9E+07**	**1.9E+07**	**1.9E+07**	**2.3E+07**	**2.3E+07**	**2.4E+07**	**2.1E+07**	**1.8E+07**	**2E+07**	**2.2E+07**	**2.4E+07**	**2.5E+07**	**2.5E+07**	**2.6E+07**	**2.6E+07**	**2.6E+07**
**Canada**	3058813	2961636	2532742	2627695	2710683	2711536	1630142	1666008	1690345	1673522	1482232	1583388	1620221	1716178	1780523	1890387	1939517	1983745	2077000
**Mexico**	1549925	1935527	1841008	1821435	1564816	1577159	1168508	1184035	1150819	1073764	775751	848354	936780	1024574	1100542	1135409	1389474	1647723	1570764
**USA**	13024978	12799857	11424689	12274917	11989387	11989387	17444329	17048981	16460315	13493165	10601368	11772219	13040613	14785936	15883443	16522663	17845624	17865773	17583842
**Argentina**	304809	339632	235558	159401	260402	260402	380189	441420	558547	599950	507794	698404	883350	830058	963917	613848	644021	709482	900403
**Bahamas**							3351	2801	2903	2900	4400	3200	3000	3500	2800	2500	3500	3300	2273
**Belize**							200	300	200	400	400	250	300	400	410	400	800	900	1165
**Bolivia**							2800	3500	3500	3700	3000	11000	14000	21000	22400	30900	21400	27800	29179
**Brazil**	1350828	1681517	1817237	1792660	2210062	2317227	1714644	1927738	2462728	2820350	3141240	3515066	3633253	3802071	3767370	3498012	2568976	2050321	2238915
**Chile**	1468	5245	10519	15270	7185	7185	200000	200000	230000	252690	180696	303360	356183	362331	397643	353525	297785	319606	376682
**Columbia**	7754	23979	25448	21756	42959	42959	150000	205000	235000	210000	195000	285000	295000	285000	286800	314100	272400	246500	233960
**Costa Rica**							16000	20000	17000	19000	16500	24000	34000	36000	36200	42100	52800	60000	51884
**Cuba**							3000	2600	3500	4100	1900	2400	3500	4500	4530	4500	4500	6900	6962
**Dominica**							25000	23000	25000	28000	12000	21000	21000	21000	21300	24400	21489	24024	19210
**Ecuador**		900	2760	1680	3620	3620	93057	89558	91778	112684	92764	132172	139893	121446	113812	120015	81309	63555	118281
**Salvador**							13000	12000	14000	16000	3000	7000	8000	11000	11400	11800	12400	10600	10182
**Guadeloupe**							17327	16544	18888	17647	16928	16009	16364	15452	14716	14773	15720	16571	18738
**Guatemala**							11800	13200	16600	18300	18500	22100	24000	26000	27900	29800	31200	29900	28250
**Guyana**							5353	5604	6182	5994	5947	5717	6036	5725	5542	5534	5623	5885	7865

**Honduras**							8500	9400	11600	14000	7300	9000	10000	11000	12000	11600	11300	10200	7771
**Jamaica**							4000	4000	5000	5000	6000	3000	3500	4500	4700	5000	5900	5100	4812
**Martinique**							17664	17151	17840	16768	15647	15286	15325	13576	13018	13351	15215	16495	16963
**Nicaragua**							8000	10000	10000	10000	5000	6000	6000	8000	9300	9400	11200	12700	9108
**Panama**							25000	30000	26000	35000	33000	38000	44000	48000	48300	52200	56300	57700	49762
**Paraguay**							8000	8000	11000	21000	12000	19000	30000	30000	30600	31000	28200	22700	27029
**Peru**			24				23346	32876	51017	92539	76932	120800	150037	190761	201326	187081	173388	169718	180020
**Puerto Rico**							136000	120000	132000	110000	110000	75000	95000	97000	99500	100200	92500	112200	127301
**Trinidad**							14000	14000	8000	14000	14000	14000	14000	14500	14900	16000	19600	20200	15053
**Uruguay**	11639	14404	10530	3579	78	78	15000	15000	19000	25000	38000	55000	54969	56459	61054	56548	51318	47114	61484
**Venezuela**	5120	21190	13170	11610	37752	37752	174757	209203	318572	200341	136517	125202	120689	130553	98878	23707	14700	5200	14084
**Asia**	**1.7E+07**	**1.8E+07**	**1.8E+07**	**2E+07**	**2.4E+07**	**2.4E+07**	**2E+07**	**2.2E+07**	**2.4E+07**	**2.4E+07**	**2.8E+07**	**3.5E+07**	**3.5E+07**	**3.8E+07**	**4.1E+07**	**4.3E+07**	**4.3E+07**	**4.7E+07**	**4.9E+07**
**Australia**	302925	347122	319375	344063	411406	411406	988269	962666	1049982	1012164	937328	1035574	1008437	1112032	1136227	1113224	1155408	1178133	1188677
**Azerbaijan**							12670	13590	15740	15800	10800	13700	10850	15000	22700	25200	10500	5100	2705
**Bahrain**							20000	23000	25000	36000	38000	35000	31000	47000	53000	61100	58300	46300	37482
**Bangladesh**							15000	19100	22000	28000	38500	40400	39900	43400	42500	51900	54800	44400	42021
**Brunei**							16000	16000	11000	16000	10500	10500	17000	20000	19600	24000	17000	16200	16839
**Cambodia**							800	800	1000	1000	1220	2900	3400	3400	3400	4100	6100	6300	6042
**China Mainland**	1829953	2069069	2334440	3251225	5070527	5234496	5758189	7215972	8791528	9380502	13644794	18061936	18505114	19306435	21984079	23499001	24661602	28028175	29122531
**Hong Kong**							35863	35467	40997	44117	32900	49237	53333	53909	57549	62200	58800	43800	48259
**India**	818193	801360	814611	891946	1511157	1511157	1440455	1750892	1993721	1983071	2266269	3040390	3287737	3595508	3241302	3177005	3424836	3669277	4017539
**Indonesia**	89007	292710	279187	299257	408311	408311	533917	318904	433341	603774	486088	764710	894164	1116230	1229811	1195409	1031422	1048135	1060894
**Iraq**							30700	32200	35000	62000	63000	165000	131500	120000	117500	87700	49200	24800	39291
**Iran**	119419	277985	323246	315000	788658	788658	857500	971000	1037900	1190000	1320000	1642843	1688194	1044430	804750	1287600	1222000	1448500	1718565
**Israel**							156000	156000	220000	192000	174500	217000	227000	206500	215500	242400	260200	292700	284623
**Japan**	9895476	10140796	9777191	10257690	10511518	10511518	5852034	5739520	5309200	5082233	4609333	4956148	4210224	5369721	5375513	5562888	5046510	4970260	5238888
**Jordan**							20100	15200	14700	18000	21000	20000	22000	23000	29000	23800	22800	20500	20918
**Kazakhstan**							42572	43020	46081	44500	45000	29000	43348	98231	165710	163561	97469	97470	97471
**Kyrgyzstan**							5800	5800	5900	4900	5450	4800	5900	4800	4800	4800	4800	2000	2735
**Kuwait**							80000	84000	90000	99500	108500	110000	114500	140000	151500	152300	143800	108000	102541
**Laos**							600	650	1300	2100	2000	5000	5000	7000	9000	14700	14600	10900	9785

**Lebanon**							16000	16000	21000	34000	34500	35500	33500	37500	37500	40300	40700	37800	38363
**Malaysia**	254090	282830	358785	395000	372916	471975	551042	490748	487176	548115	536905	605156	600123	627753	655793	666487	666677	580124	591096
**Mongolia**							800	1300	1700	3750	3600	3300	3290	2290	2200	1600	1000	1700	1655
**Myanmar**							2000	2000	2000	2200	2500	2800	2800	3100	3000	1800	1800	2300	3344
**Nepal**							1400	1300	2500	1900	2200	2000	4000	5600	5500	8300	5900	9300	3670
**New Caledonia**							10670	11298	12460	12684	12215	12772	12826	11987	10375	10910	9974	9279	10294
**New Zealand**							117426	112211	115041	106190	73202	83250	84143	100795	113294	126508	134041	146753	159872
**Oman**							208300	206000	204400	198500	155500	178000	169500	204500	215000	208400	167600	154900	131149
**Pakistan**	15000	31500	17196	24868	93172	93172	167007	187644	188109	157182	111696	152354	163260	157656	141778	146882	229688	211295	269049
**Palestine**							16600	18600	19700	22900	20600	21900	24700	36600	35800	35800	35800	35800	36454
**Philippines**	30662	41840	42297	53663	70728	70728	97063	99541	117903	124449	132444	170348	165056	184248	211959	234747	288609	359572	358558
**Qatar**							85000	84000	83400	81000	55000	55500	61000	80000	85000	92900	86400	63700	49767
**Saudi Arabia**							566400	560000	555800	540000	520000	600000	590000	705000	740000	828200	830100	655500	548250
**Singapore**							125018	132483	122254	110574	79503	51891	39570	37247	34111	47434	76191	108061	110907
**South Korea**	2843114	3114998	2946329	3147584	3469464	3469464	1170495	1205713	1273253	1216125	1453875	1511373	1586405	1532087	1543564	1661868	1833786	1823041	1798796
**Sri Lanka**							4000	4200	4700	4800	5000	3000	8000	9500	12000	15500	17000	34900	16006
**Syrian**							39985	51467	41500	46800	51600	87547	39960	44000	43100	39700	39700	39700	40034
**Tajikistan**							5440	5850	5990	6200	7200	6700	7710	6300	6290	6400	6400	2300	2316
**Tahiti**							7300	7100	7700	7200	5200	5500	5000	4000	3500	3900	3400	3800	5232
**Taiwan**	353000	372613	271705	336699	430814	430814	446477	306458	276434	178809	229450	252500	285790	270078	258753	282130	262593	262346	259013
**Thailand**	322761	411721	459418	595649	927981	927981	692506	674953	631181	615270	548870	800357	794081	1423580	1330672	881810	799632	768788	873506
**Turkmenistan**							4000	4300	4420	4700	4700	4700	5200	4700	4700	4800	4800	5000	5016
**UAE**							157000	175000	165000	233592	205088	168323	243982	268900	263100	263100	256700	198500	194382
**Uzbekistan**	44433	32273	41005	29551	80729	80729	35200	38100	40800	56500	59000	56500	62000	57000	57500	58100	58100	56300	58968
**Vietnam**		6862	10673	13197	19868	19868	35264	41133	81056	111950	119459	112224	110938	80487	96692	134562	208566	271833	269570
**Yemen**							9100	10800	10700	11000	11000	4000	4000	4100	4000	4600	5600	3600	4950
**Africa**	**301461**	**328749**	**393254**	**285813**	**422017**	**422667**	**1127349**	**1328314**	**1340152**	**1278762**	**1181065**	**1273898**	**1473138**	**1600993**	**1685143**	**1699621**	**1549556**	**1315163**	**1195765**
**Algeria**							99422	109578	127030	146892	176773	133406	227925	255167	304854	246400	215400	99600	94408
**Angola**							9000	12000	5500	5500	15000	30000	15000	28000	29000	35700	18000	7300	4230
**Botswana**	4560		1608	1605			18532	17639	23378	30011	29291	28677	32411	38130	38456	34800	7500	8100	7600
**Burkina Faso**							700	700	700	900	1050	1100	1500	800	1100	900	1400	2300	1027
**Burundi**							1000	1000	1100	1000	1000	600	500	510	530	400	400	900	130
**Cameroon**							3500	4100	4000	3800	2600	2900	2900	3400	4400	4400	4200	4200	3765
**Congo**							1090	1260	1110	1300	3500	2500	2500	3000	2400	2400	3100	2300	1849
**Egypt**	76048	59765	56097	45173	49335	49335	121437	170614	206060	239833	205521	248917	271900	286300	283000	349100	332100	264100	181001

**Gabon**							3800	4000	4200	4000	4000	4000	4000	5500	5500	4900	3300	3000	2565
**Ghana**							4500	4500	5100	5500	6500	6000	9000	14500	13600	13000	4800	7400	4217
**Cote d'Ivoire**							3000	3500	4000	4000	4500	5500	5500	7500	6000	6400	7100	6400	7118
**Kenya**	332	288	288	500			6000	5000	7000	7000	7500	6000	6000	9500	13000	13500	14100	10600	11886
**Liberia**							400	300	400	500	300	500	400	410	430	400	400	600	132
**Libya**	2040	2040	6840				47000	48000	49500	51500	53000	54500	56500	29000	45000	45000	54100	46400	23600
**Madagascar**							2630	3100	3000	2700	2000	1800	2100	2200	2200	2600	2300	1400	1507
**Malawi**							1400	1670	1660	1500	1500	2200	2300	1500	1540	1700	1900	1800	1937
**Mauritius**							4800	5500	6000	6000	5000	6000	4500	9500	10200	10600	10000	11000	12597
**Morocco**	21051	19432	21545	25056	12996	12996	63655	84276	103597	121360	109969	103436	112093	130306	120755	122060	131910	163110	168913
**Nigeria**	6161	7384	8090	6168	4272	4272	23000	38000	12000	12000	51000	37000	45000	50000	52000	53900	20000	23000	6999
**Reunion**							31627	29782	32149	29979	25839	24755	26268	24949	24233	25757	27697	29547	31039
**Senegal**							5500	6500	7000	7000	6000	6000	5500	6000	6000	6800	5800	6600	6516
**South Africa**	317367	357364	407036	404441	455052	455702	617406	714315	676108	533387	395222	492907	572241	623921	650745	644504	617749	547406	555716
**Sudan**					1020	1020	3000	3000	4500	5000	12000	3500	3800	3500	2500	3000	3600	3400	2955
**Tanzania**							3000	3000	3500	3000	3000	3200	4500	6000	6700	5800	5200	4500	5358
**Tunisia**							33000	38000	39000	43000	47000	59000	47500	49000	47000	50900	48500	50800	47359
**Uganda**							4000	4500	4000	3500	3500	3500	4000	4500	5000	4900	3100	4000	4337
**Zambia**							2950	3480	3560	3600	4000	3000	3000	3500	4000	4000	3400	3200	4862
**Zimbabwe**	577	792	1481	1816	384	384	12000	11000	5000	5000	4500	3000	4300	4400	5000	5800	2500	2200	2143
**SUM**	**5.6E+07**	**5.8E+07**	**5.6E+07**	**5.9E+07**	**6.4E+07**	**6.4E+07**	**6.6E+07**	**6.8E+07**	**7.2E+07**	**6.8E+07**	**6.6E+07**	**7.5E+07**	**7.8E+07**	**8.2E+07**	**8.6E+07**	**8.8E+07**	**9E+07**	**9.4E+07**	**9.7E+07**

Note: Vehicles consist of car, multi-purpose vehicle, sport utility vehicle, minivan, pickup, cross passenger car, and light commercial vehicle.

Data from China Association of Automobile Producers http://www.caam.org.cn/

(B) Production of vehicles from 2010 to 2023

	**2010**	**2011**	**2012**	**2013**	**2014**	**2015**	**2016**	**2017**	**2018**	**2019**	**2020**	**2021**	**2022**	**2023**
**China**	18264761	18418876	19271808	22116825	23722890	24503326	28118794	29015434	27809196	25720665	25225242	26082220	27020615	30160966
**United States**	7743093	8661535	10335765	11066432	11660699	12100095	12198137	11189985	11314705	10880019	8822399	9167214	10060339	10611555
**Japan**	9628920	8398630	9943077	9630181	9774558	9278321	9204590	9693746	9728528	9684298	8067557	7846955	7835519	8997440
**India**	3557073	3927411	4174713	3898425	3840160	4160585	4519341	4782896	5174645	4516017	3490000	4399112	5456857	5851507
**South Korea**	4271741	4657094	4561766	4521429	4524932	4555957	4228509	4134913	4028834	3950617	3506774	3462404	3757049	4244000
**Germany**	5905985	6146948	5649260	5718222	5907548	6033164	5746808	5645581	5120409	4661328	3742454	3308692	3677820	4109371
**Mexico**	3981728	3907861	4002508	4219380	4664779	4029463	4396356	4137544	4100770	3986794	3176600	3145653	3509072	4002047
**Spain**	2387900	2373329	1979179	2163338	2402978	2733201	2885922	2848335	2819565	2822355	2268185	2098133	2219462	2451221
**Brazil**	3381728	3407861	3402508	3712380	3146306	2429463	2156356	2699672	2879809	2944988	2014055	2248253	2369769	2324838
**Thailand**	1644513	1457798	2429142	2457057	1880007	1915420	1944417	1988823	2167694	2013710	1427074	1685705	1883515	1841663

**Canada**	2068189	2135121	2463364	2379806	2393890	2283474	2370271	2199789	2020840	1916585	1376623	1115002	1228735	1553026
**France**	2229421	2242928	1967765	1740000	1817000	1972000	2082000	2227000	2270000	2202460	1316371	1351308	1383173	1505076
**Turkey**	1094557	1189131	1072978	1125534	1170445	1358796	1485927	1695731	1550150	1461244	1297878	1276140	1352648	1468393
**Czech Republic**	1076384	1199845	1178995	1132931	1251220	1303603	1349896	1419993	1442884	1433963	1159151	1111432	1224456	1404501
**Indonesia**	702508	838388	1052895	1206368	1298523	1098780	1177389	1216615	1343714	1286848	691286	1121967	1470146	1395717
**Iran**	1599454	1649311	1000089	743647	1090846	982337	1164710	1515396	1095526	821060	880997	894298	1064215	1188471
**Slovakia**	561933	639763	926555	975000	993000	1035503	1040000	1001520	1090000	1107902	990598	1000000	1000000	1080000
**United Kingdom**	1393463	1463999	1576945	1597433	1598879	1682156	1816622	1749385	1604328	1381405	987044	932488	876614	1025474
**Italy**	838186	790348	671768	658206	697864	1014223	1103516	1142210	1060068	915305	777165	795856	796324	880085
**Malaysia**	567715	533695	572150	601407	596600	614671	513445	460140	564800	571632	485186	481651	702225	774600
**Russia**	1403244	1990155	2233103	2184266	1886646	1384399	1303989	1551293	1767674	1719784	1435335	1566317	608060	729864
**South Africa**	472049	532545	539424	545913	566083	615658	599004	589951	610854	631983	447218	499087	555889	633337
**Poland**	609474	708133	654756	690159	693904	760603	781837	789729	809646	498158	451382	439421	483840	612882
**Argentina**	716540	828771	764495	791007	617329	533,683	472,776	472158	466649	314787	257187	434753	536893	610725
**Morocco**	42066	59477	108,743	167,452	231986	288329	345106	376286	402085	394652	248430	403007	464864	535825
**Romania**	350912	335232	337765	410997	391422	387177	359306	359250	476769	490412	438107	420755	509465	513050
**Hungary**	211461	202800	217840	222400	437599	495,370	472000	505400	430988	498158	406497	394302	441729	507225
**Uzbekistan**	156880	179560	164,180	246641	245660	185400	88152	140247	220667	271113	280080	241649	333569	425876
**Belgium**	555302	595084	538848	503504	516832	409340	399427	379140	308493	285797	267293	261038	276554	332103
**Portugal**	158723	192242	163561	154016	161509	156626	143096	175544	294366	345704	264236	289954	322404	318231
**Taiwan**	303456	343296	339038	338720	379223	351085	309531	291563	253241	251304	245615	265320	261263	285962
**Sweden**	188,969	162,814	161,080	154,173	188,987	205374	226000	291000	279000	249000	258000	257446	238955	276750
**Vietnam**	42286	31181	40,470	37,576	48871	50,000	236161	236161	237000	250000	165568	163250	232410	177435
**Kazakhstan**	3176	8195	19186	40684	40162	14477	10651	19071	31545	49400	74831	92417	112540	146989
**Netherlands**	94132	73,151	57462	29183	29,807	44,122	44,430	157280	214000	176113	127058	107021	101670	123379
**Austria**	104814	152505	143,060	166,428	154,340	125,500	108,000	99880	164900	179400	104544	136700	107500	114191
**Philippines**	65625	53921	55,360	52,260	106938	112,493	116686	141251	79763	95094	67297	85874	92223	110350
**Pakistan**	152970	162194	165700	134145	148746	229686	214650	250800	269700	186751	117375	238702	235454	79513
**Slovenia**	205711	174119	130949	93734	118591	133092	133702	189852	209378	199102	141714	95797	68130	60881
**Colombia**	109333	41714	32466	70,686	71137	78,070	79,036	74994	75476	78020	47281	40764	51455	34700
**Finland**	6665	2540	2,900	7703	45,035	69,053	55,280	91598	112104	114785	86270	93172	73044	30191
**Australia**	243495	224193	209730	215926	180311	173009	161294	98632	6371	5606	4730	5391	6077	7141
**Azerbaijan**					669	2145	415	247	1453	2360	1949	2173	2049	4649
**Algeria**					1244	19346	42008	60606	70597	60012	754	5208	2773	2456
**Ukraine**	83133	104654	76281	50449	28751	8244	5264	9542	6623	7266	4952	8153	1490	1993

**Myanmar**										15496	10753	1957		1475
**Serbia**	18033	11023	11032	113878	103150	83,630	80320	79912	56449	35115	23375	21263	4498	186
**North Korea**	4000	4000	4000	4000	4000	4000	4000	3000	3000	2000	2000	2000	2000	
**Belarus**	15249	24343	25425	22926	22990	15033	16864	13428	23235	30487	31273	29891		
**Egypt**	116683	81731	56,480	39,050	42515	36,000	36,230	36640	71600	18500	23754	23754		
**Ecuador**	32254	22335	24322	24,322	24,322	5986	4,800	2,700	2,700					
**Bulgaria**				2200	2,508									
**Tunisia**		1860	1,860	1,860	540	540	1,940	1,940	2000					
**Venezuela**	135425	104357	102409	104083	71753	19759	18300	2850	1774					
**Bangladesh**						536	540	580	580					

Data from International Organization of Motor Vehicle Manufacturers: https://www.oica.net/production-statistics/

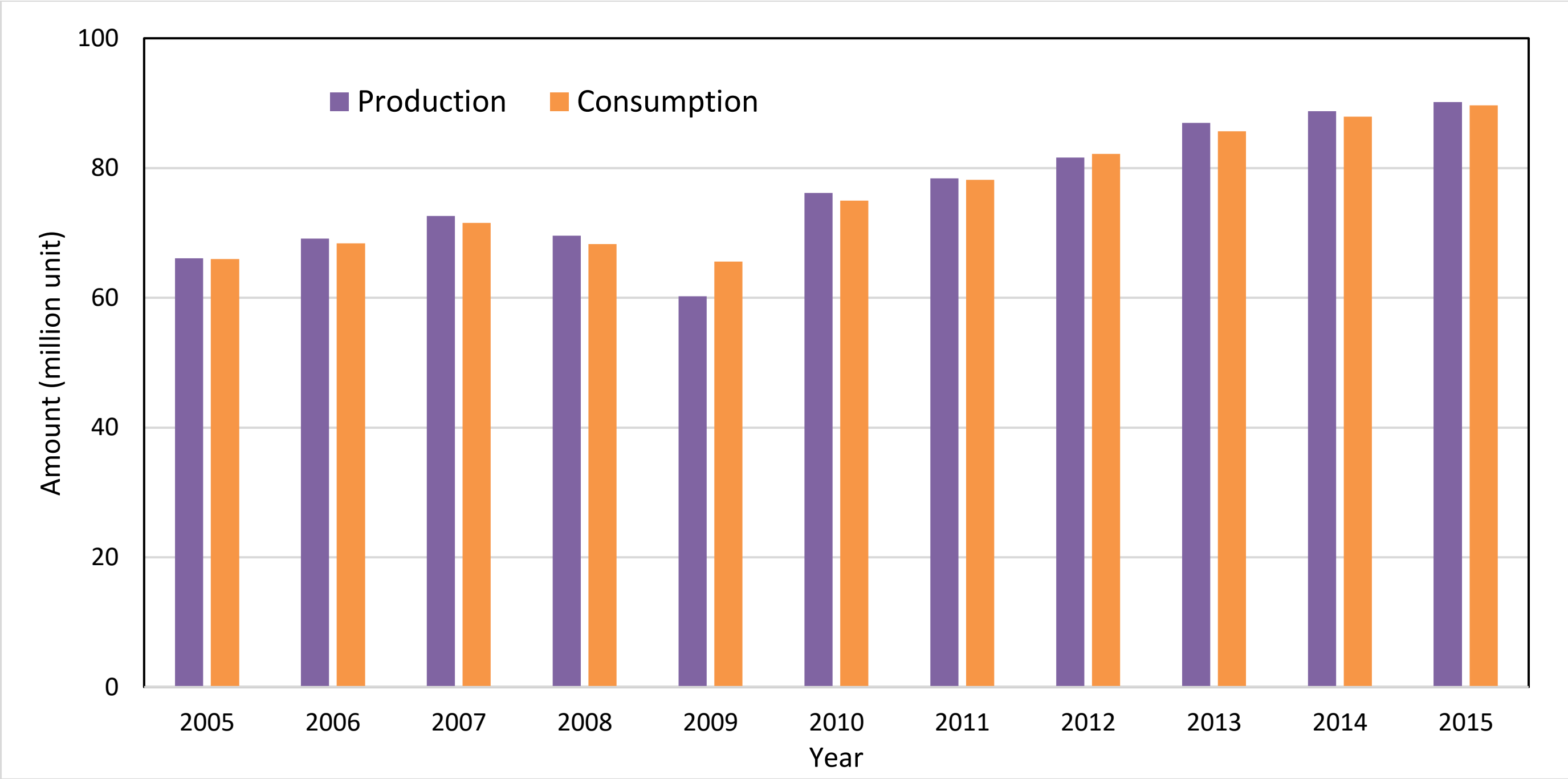

Figure S1: Comparison between production and consumption of passenger cars and commercial vehicles in the world

Data from https://www.bts.gov/archive/publications/national_transportation_statistics/table_01_23
http://www.caam.org.cn/zongheshuju/20180510/1605217108.html

Table S4: PCs production and export amount in 1960-1988

Year	U.S.		Japan		Germany		World
	Production	Export	Production	Export	Production	Export	Production
1960	6703108	100000	200000	10000	1816779	850000	12838910
1961	5522019	100000	295000	20000	1903975	900000	11390629
1962	6943334	100000	300000	50000	2109166	1000000	14011817
1963	7644377	140000	450000	80000	2414107	1200000	15941095
1964	7745492	150000	600000	100000	2650183	1380000	16734646
1965	9335227	110000	650000	150000	2733732	1400000	18951914
1966	8604712	120000	800000	200000	2830050	1500000	19151634
1967	7412659	150000	1375755	300000	2295714	1400000	18574476
1968	8848620	150000	2000000	450000	2862186	1800000	21613654
1969	8224392	300000	2600000	600000	3312539	1900000	23103996
1970	6550128	400000	3178708	700000	3627864	1950000	22494605
1971	8583653	400000	3700000	1300000	3696779	2150000	26447611
1972	8828205	500000	4000000	1400000	3521540	2000000	27886168
1973	9667152	600000	4470550	1400000	3649880	2200000	29984605
1974	7324504	650000	3980000	1700000	2839596	1700000	25946555
1975	6716951	700000	4500000	1800000	2907819	1500000	24958731
1976	8497893	700000	5000000	2500000	3546900	1820000	28793648
1977	9213654	730000	5431045	2990000	3790544	1980000	30474089
1978	9716635	780000	6000000	3000000	3890176	1950000	31183666
1979	8433662	800000	6200000	3050000	3932556	2000000	30774627
1980	6375506	600000	7000000	3850000	3520934	1900000	28577518
1981	6253138	550000	6950000	3850000	3577807	2000000	27457792
1982	5073496	400000	6800000	3700000	3761436	2200000	26626660
1983	6781184	580000	7151888	3750000	3877642	2200000	29966497
1984	7773332	600000	7040000	3950000	3790164	2250000	30478607
1985	8184821	720000	7600000	4400000	4166686	2600000	32233228
1986	7828783	700000	7809809	4500000	4310828	2520000	32849675
1987	6950000	680000	8000000	4700000	4400000	2450000	
1988	7000000	660000	8200000	4600000	4300000		

Note: data source from ref. [5].

Supplementary Text

Parameters determining of the revised Weibull distribution

The average lifespan of vehicles varies significantly in both temporal and spatial dimensions[6], with an increase of nearly 27% observed between 1969 and 2014[7]. In contrast, the service life of passenger cars (PCs) in industrialized nations has remained relatively constant over the past two decades, with a range of 10 to 15 years (Fig. S3).

Table S5: The scale and shape parameters of Weibull distribution function for vehicles

Type	η (scale)		β (shape)	
	Average	STDEV	Average	STDEV
PCs	12.71	2.62	3.52	0.49
CVs	9.94	1.06	5.58*	1.11*

Note: The calculated value is obtained from the collected data in Tables S6-S11 and Figure S2; * data source from Zeng et al. (2020)[3]; all the value fit for the normal distribution. Lifespan regulation in compulsory obsolescence in some countries.

Table S6: Average lifetime of automobiles and trucks in the U.S. (year)

Year	Passenger cars	Light trucks	All light vehicles	Year	Passenger cars	Light trucks	All light vehicles
1995	8.4	8.3	8.4	2010	10.8	10.5	10.6
1996	8.5	8.3	8.5	2011	11.1	10.8	10.9
1997	8.7	8.5	8.6	2012	11.3	11.1	11.2
1998	8.9	8.5	8.8	2013	11.4	11.3	11.4
1999	9.1	8.5	8.8	2014	11.4	11.4	11.4
2000	9.1	8.4	8.9	2015	11.5	11.5	11.5
2001	9.3	8.4	8.9	2016	11.6	11.6	11.6
2002	9.8	9.4	9.6	2017	-	-	11.7
2003	9.9	9.5	9.7	2028	-	-	11.7
2004	10	9.5	9.8	2019	-	-	11.8
2005	10.1	9.5	9.8	2020	-	-	11.9
2006	10.2	9.5	9.9	2021	-	-	12.1
2007	10.3	9.6	10	2022	13.1	11.6	12.2
2008	10.4	9.8	10.1	2023	13.6	11.8	12.5
2009	10.5	10.1	10.3				

Note: Data obtained from: https://www.bts.gov/content/average-age-automobiles-and-trucks-operation-united-states[8]

Table S7: Scale and shape parameters, and regulated lifespan of Weibull distribution function

Type		η (scale)	β (shape)	L (The regulated maximum lifespan) in China [9,10]	L (The regulated maximum lifespan) in India*
Commercial vehicle	Private passenger vehicle	14.46	4.79	10	15
	Civil vehicle	13.11	5.33	20	
	New-registration civil vehicle	11.53	5.08	20	
Passenger car	Taxi	5		8	20
	Private vehicle	14.46	4.79	None	

Note: source from refs [11,12]; * Vehicle Scrappage Policy 2021: https://www.ibef.org/blogs/vehicle-scrappage-policy-2021-expectations-and-challenges

Table S8: Percent scrappage rate by age for passenger cars and light trucks, Related to Figure S2

Model year	Passenger cars (%)			Light trucks (%)		
Age	1969-1979	1980-1987	1988-2014	1969-1979	1980-1987	1988-2014
2	1.51	0.61	0.87	0.78	0.99	0.64
3	1.84	1.45	0.92	1.32	0.65	0.69
4	2.03	1.15	1.30	1.27	1.10	0.96
5	2.56	1.74	1.62	1.61	1.27	1.13
6	3.79	3.28	2.11	2.20	1.99	1.37
7	5.30	3.94	2.56	2.99	2.23	1.56
8	7.17	5.32	3.38	3.75	2.73	2.06
9	9.40	6.86	4.24	4.40	3.71	2.74
10	11.75	8.57	5.59	5.85	4.99	4.08
11	13.84	10.13	6.97	5.33	4.79	5.09
12	15.65	11.77	8.84	6.57	6.86	5.99
13	17.18	13.13	10.64	7.08	6.54	6.94
14	18.29	14.56	12.78	7.53	9.16	8.07

Note: Data source from ref. [7].

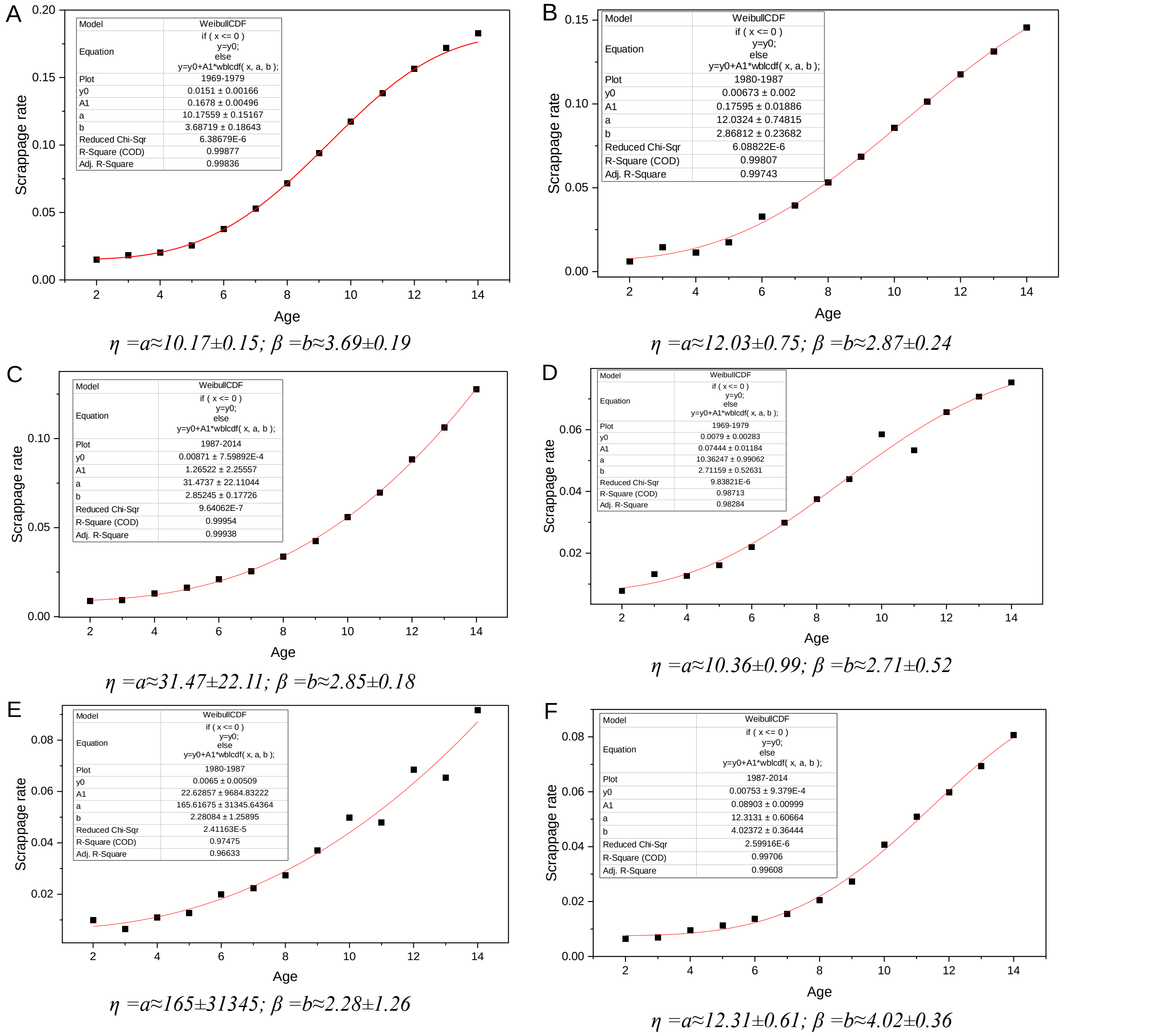

Figure S2: Fitted scale and shape parameter of Weibull distribution function, Related to Table S8

Note: (A) PCs in 1969-1979; (B) PCs in 1980-1987; (C) PCs in 1988-2004; (D) LTs in 1969-1979; (E) LTs in 1980-1987; (F) LTs in 1988-2004. The scale parameters shown in Fig. S2C and S2F would not be employed owing to the unreasonably physical significance.

Table S9: Estimated lifetime distribution parameters for PCs in 2008[6]

Country	η (scale)	β (shape)	Country	η (scale)	β (shape)
Australia	22.6	3.0	Italy	14.1	4.0
Austria	15.4	4.2	Japan	13.3	3.4
Brazil	18.5	2.8	Netherlands	15.1	3.7
Canada	15.4	4.4	South Korea	13.0	2.7
Denmark	16.7	3.5	Spain	18.0	4.7
Finland	22.0	2.9	Switzerland	14.1	3.4
France	15.2	4.2	United Kingdom	13.5	3.9
Germany	13.7	3.1	United States	16.2	2.8
Ireland	13.0	4.0			

Table S10: Temporal variation of lifetime distribution parameter for PCs in four countries [6]

Year	Germany		UK		Japan		US	
	η (scale)	β (shape)	η (scale)	β (shape)	η (scale)	β (shape)	η (scale)	β (shape)
1999							15.0	3.5
2000	13.4	3.6	13.9	4.1	10.6	3.7	15.0	3.3
2001	13.5	3.6	13.8	4.0	10.8	3.6	15.0	3.2
2002	13.6	3.5	13.7	4.0	11.0	3.6		
2003	13.6	3.3	13.7	4.1	11.1	3.5		
2004	13.7	3.2	13.6	4.2	11.4	3.5		
2005	13.9	3.0	13.7	4.2	11.6	3.4		
2006	14.1	2.9	13.6	4.1	11.7	3.4	15.9	2.7
2007	13.5	3.2	13.6	4.1	11.9	3.3		
2008	13.7	3.1	13.5	3.9	12.0	3.2	16.2	2.8
2009	13.3	2.8			12.2	3.3		
2010					12.3	3.3		

Table S11: Average of end-of-life vehicle service life in Japan (year)

Year	2005	2015	2016	2017	2018
η	12.0	14.9	15.2	15.3	15.5

Note: https://www.jarc.or.jp/renewal/wp-content/uploads/2018/10/mini10-questions-about-automobile-recycling-in-Japan.pdf

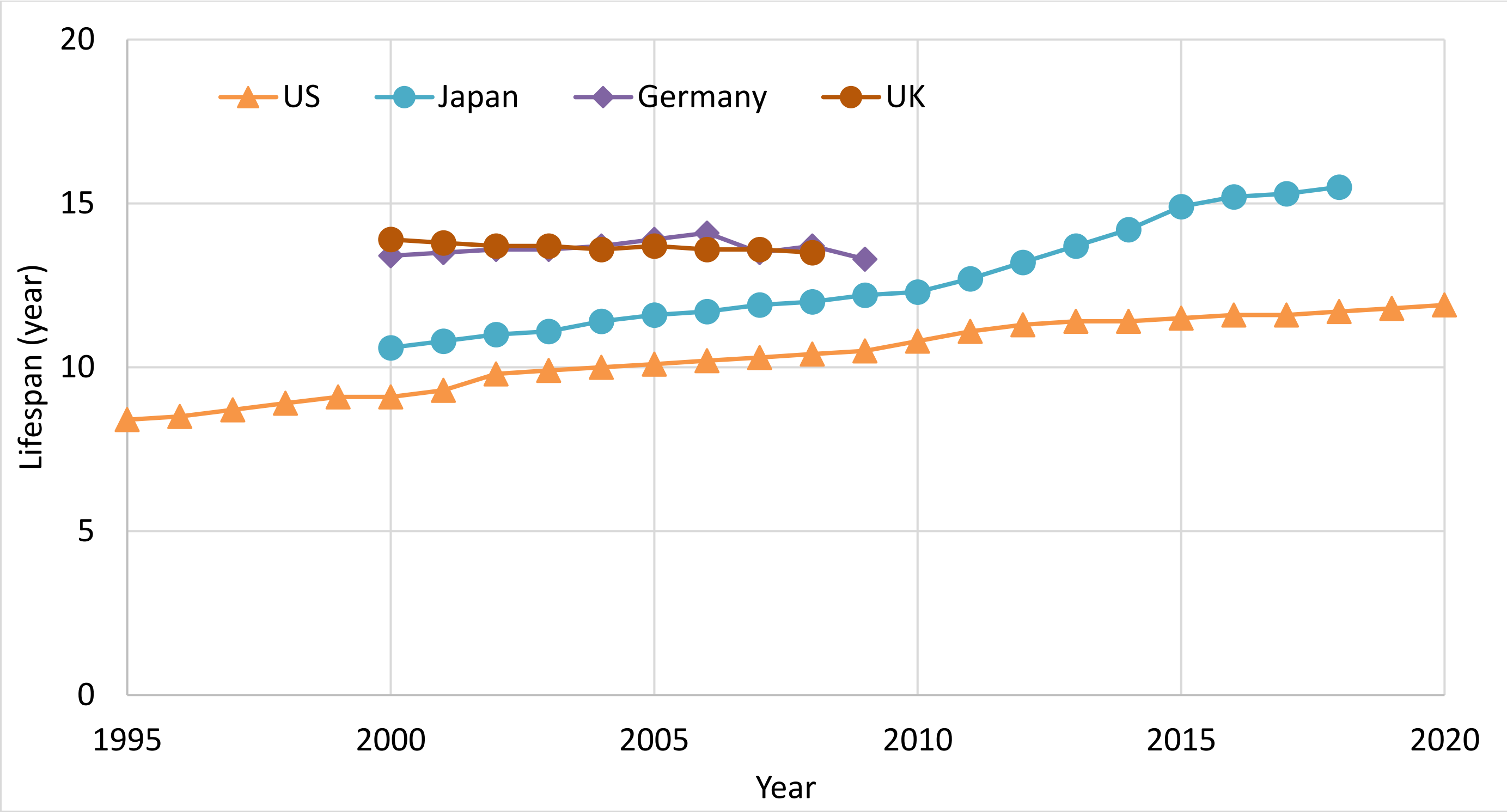

Figure S3: Historical evolution of lifespan of PCs in high-income countries

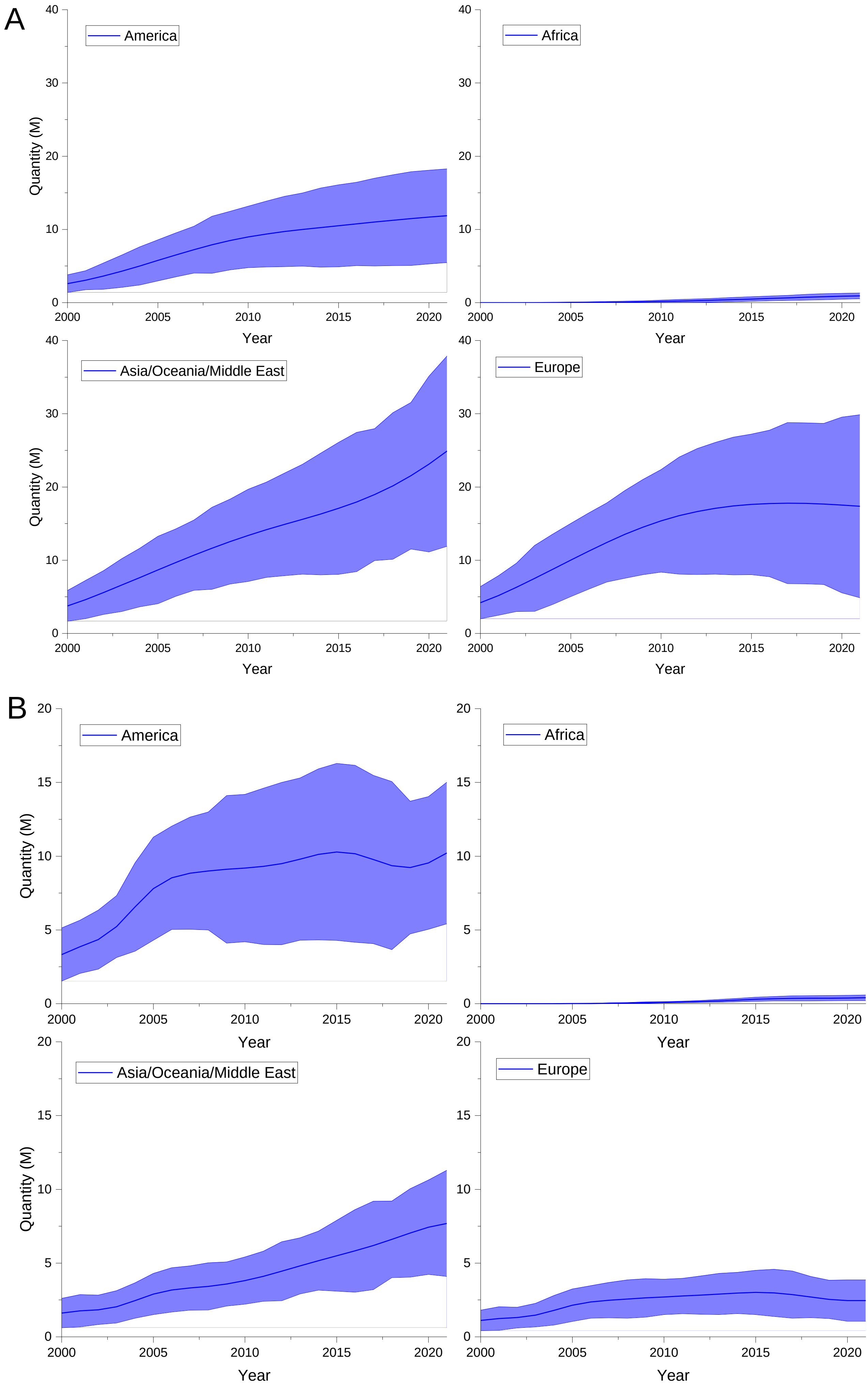

A
America
Africa
Asia/Oceania/Middle East
Europe
Quantity (M)
Year
B
America
Africa
Asia/Oceania/Middle East
Europe
Quantity (M)
Year

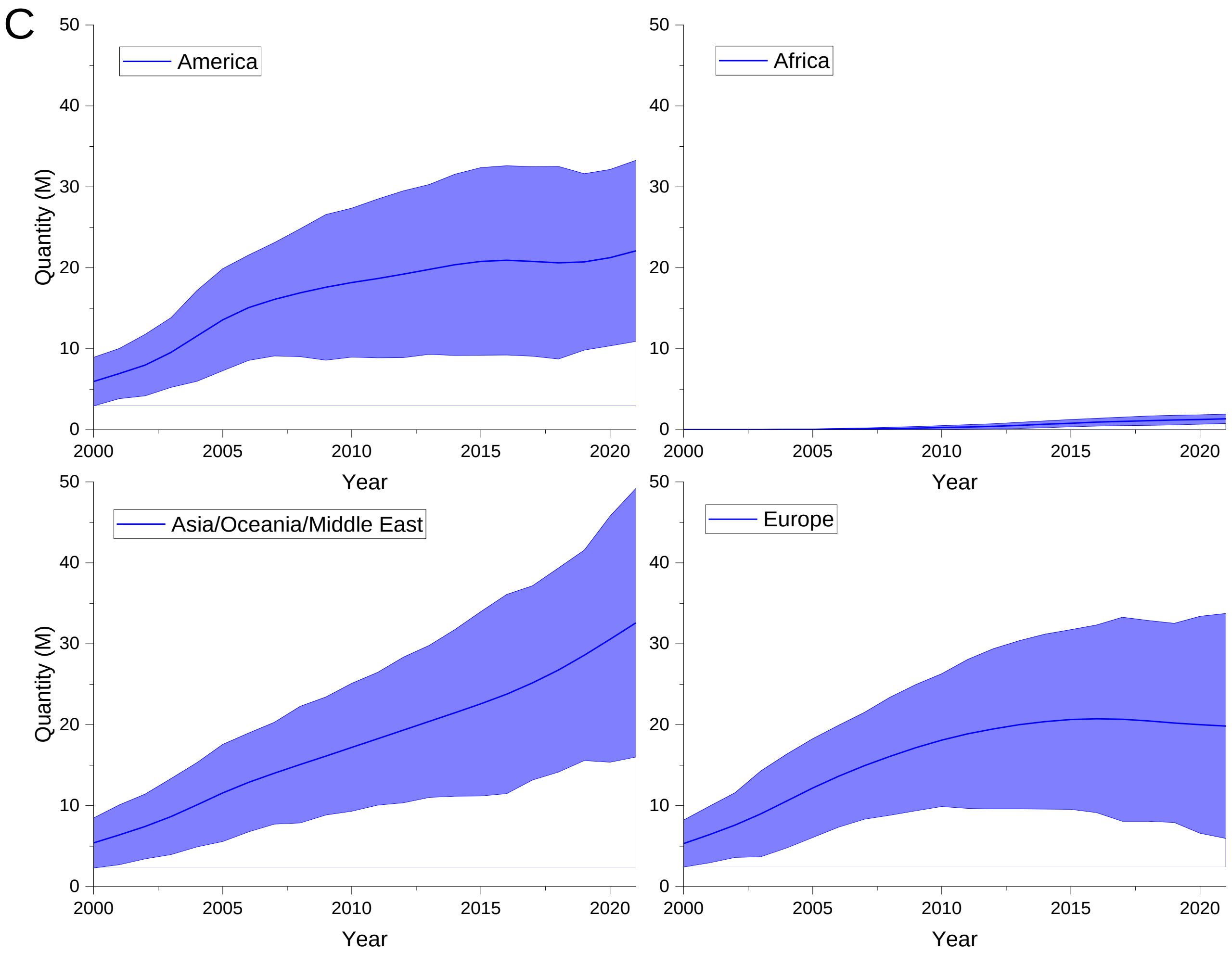

Figure S4: Estimated generation of EoL products from 2000 to 2019 in the main continents: (A) EoL PCs; (B) EoL CVs; (C) ELVs, Related to Figure 1.

Table S12: Uncertainty analysis of EoL PCs generation in main continents in 2000-2021, Related to Figure 1.

Year	Africa	America	Asia/Oceania/Middle East	Europe	Sum
2000	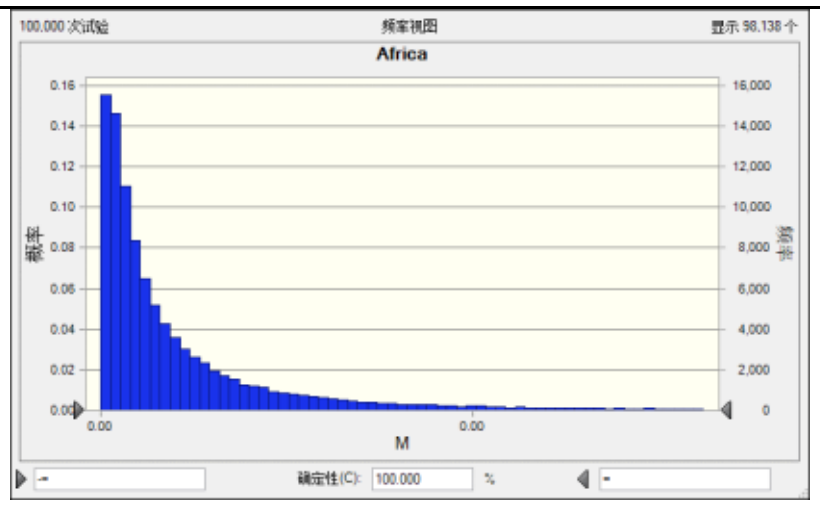	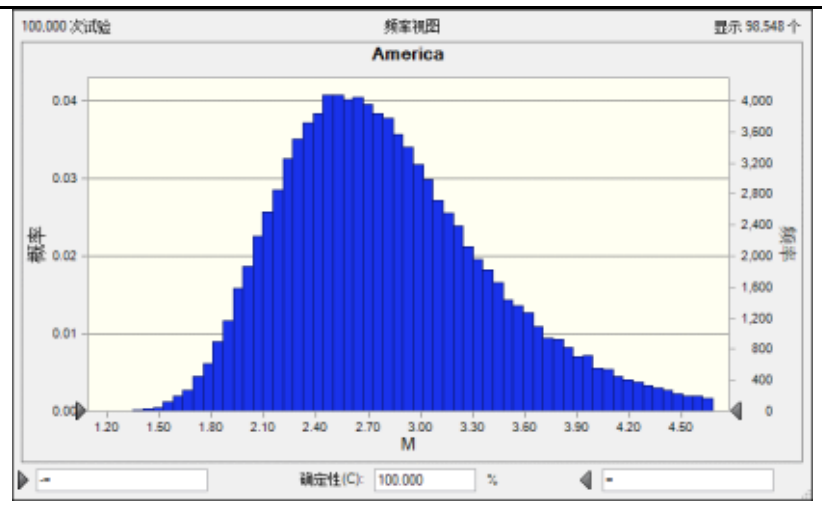	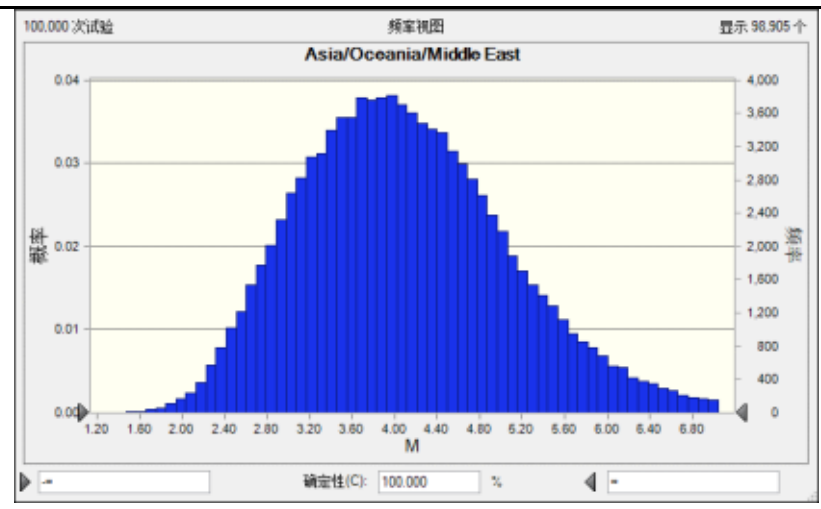	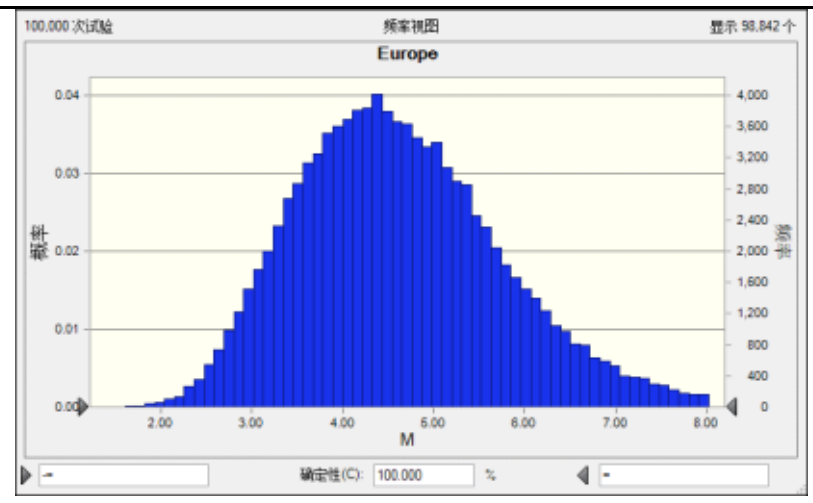	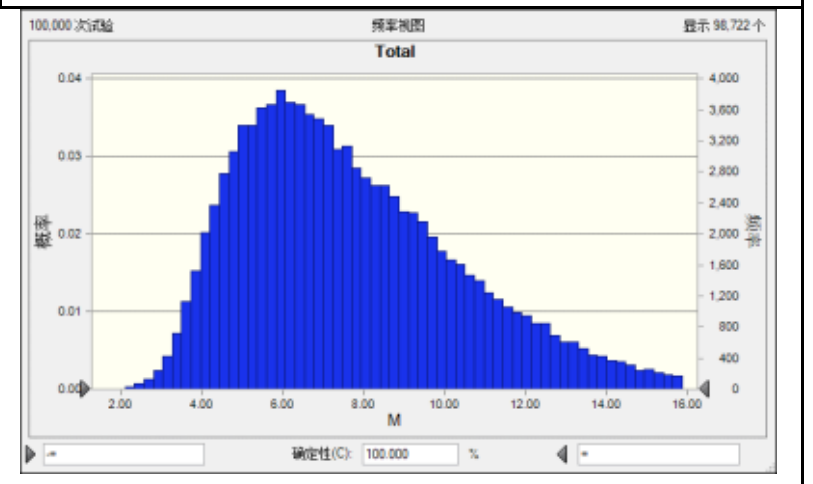
2001	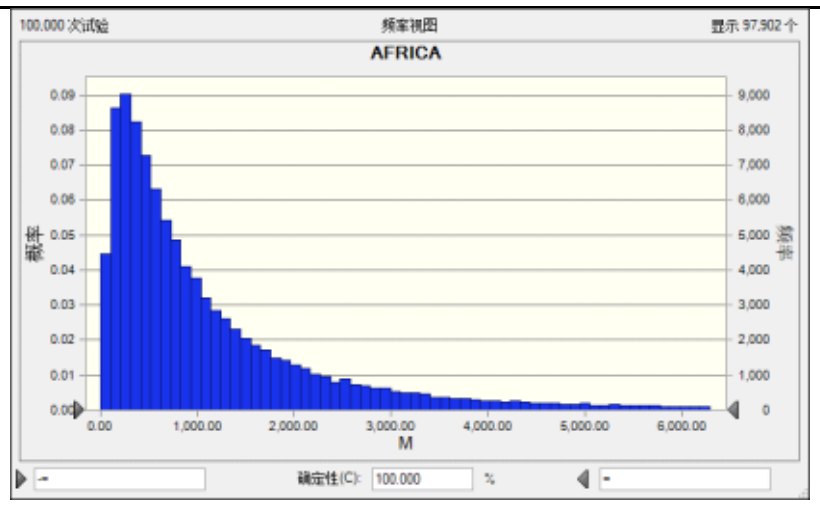	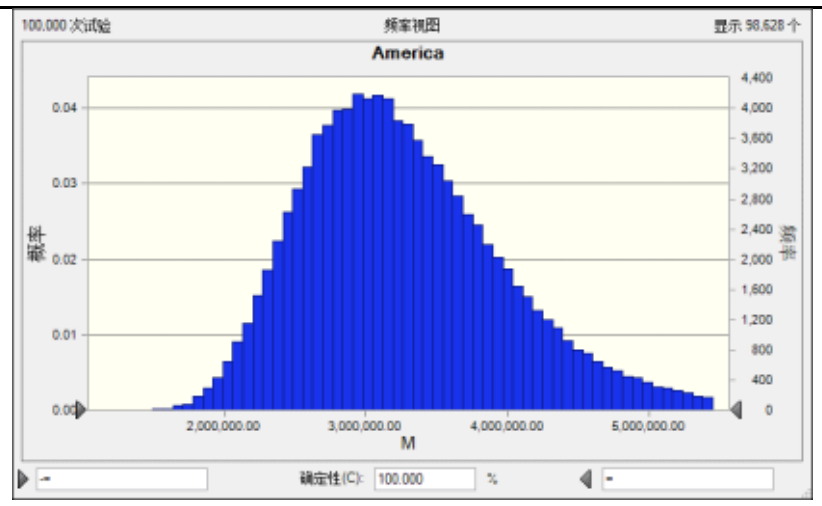	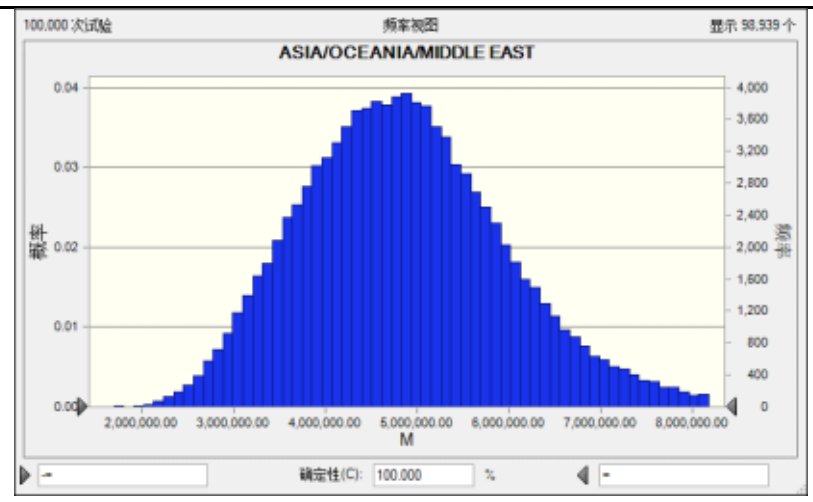	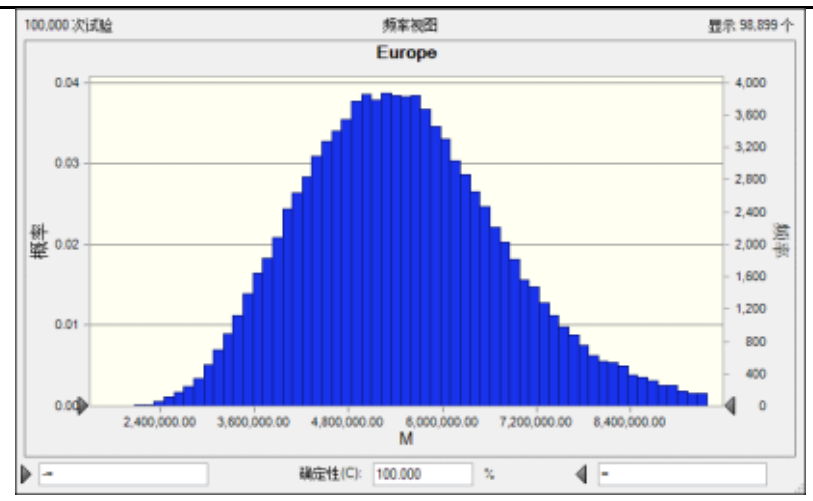	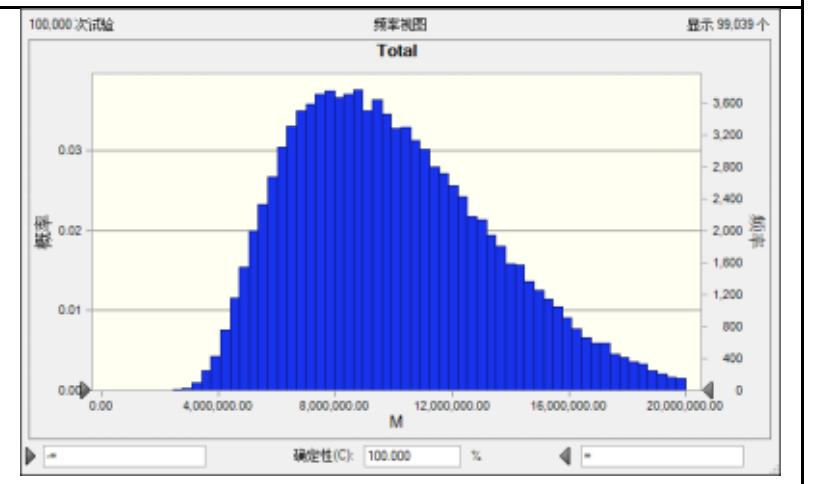
2002	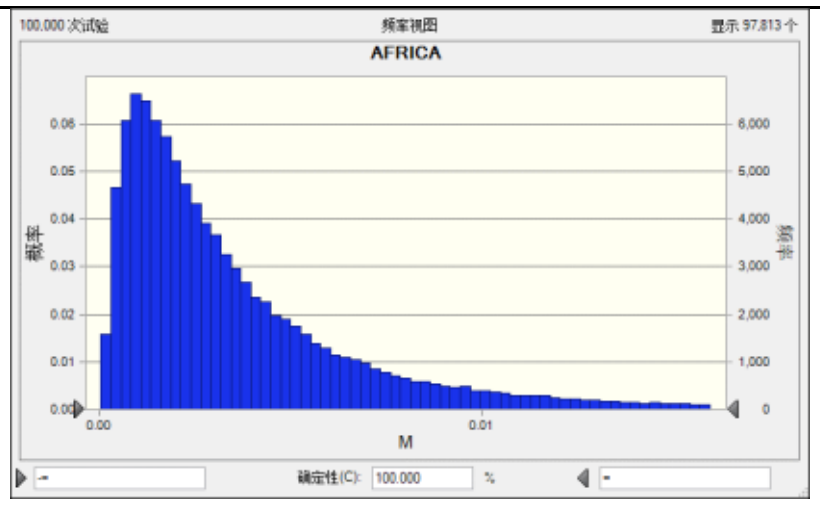	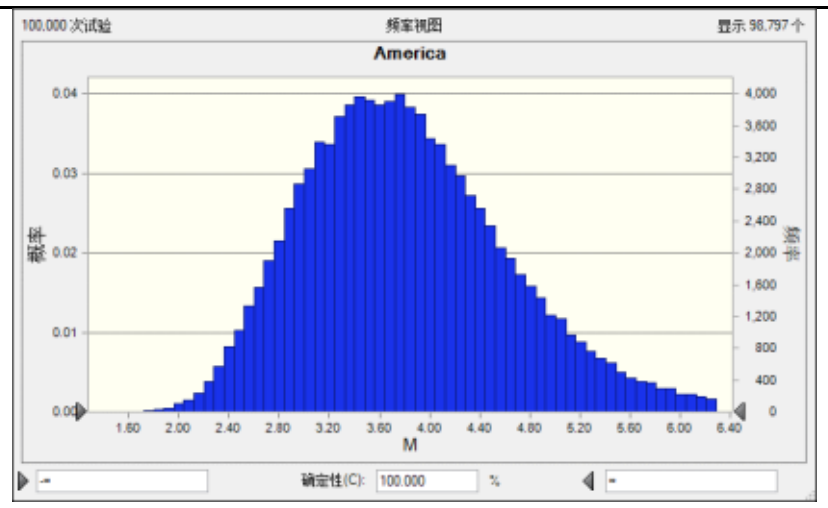	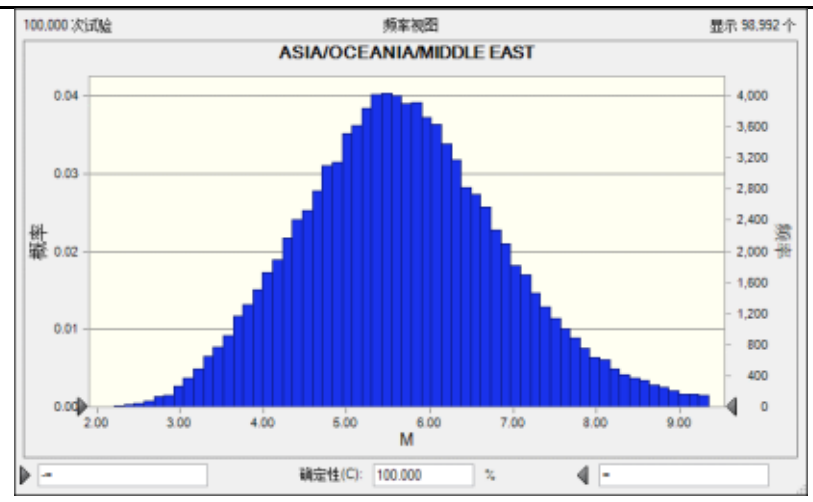	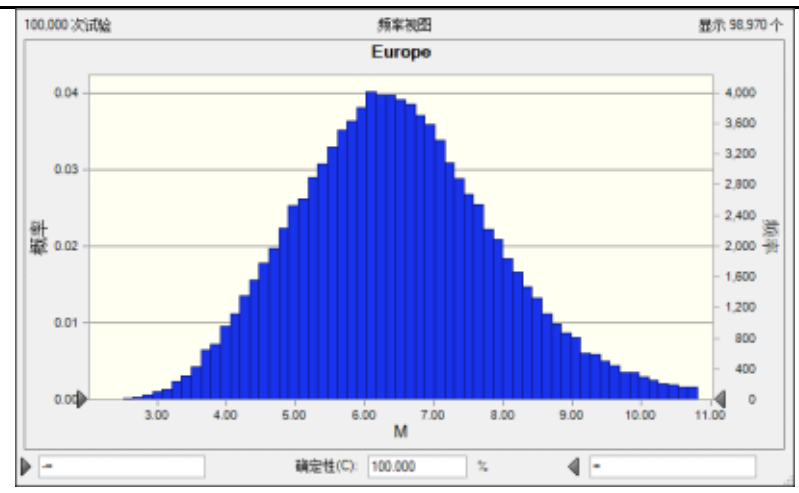	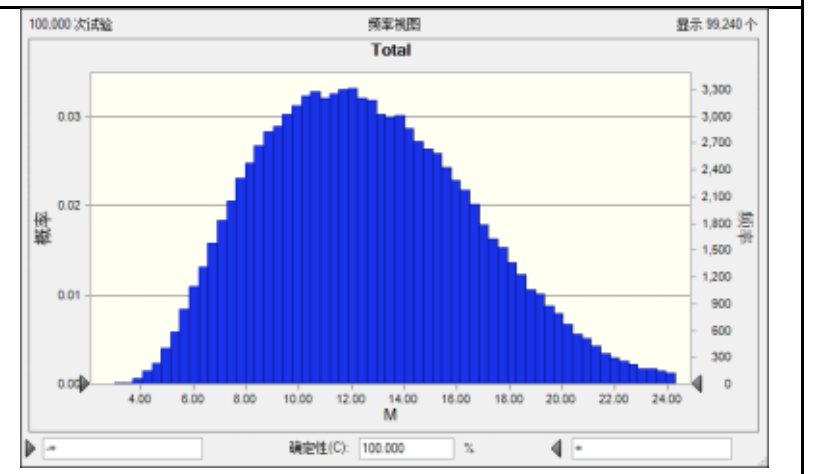
2003	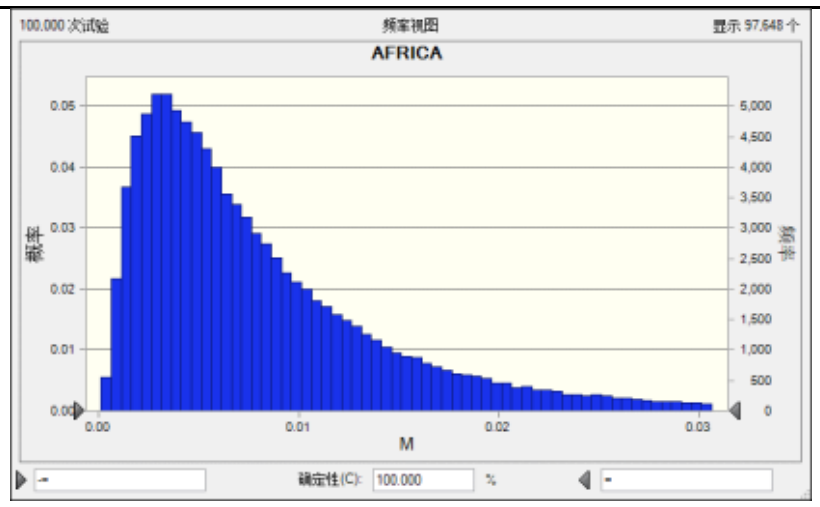	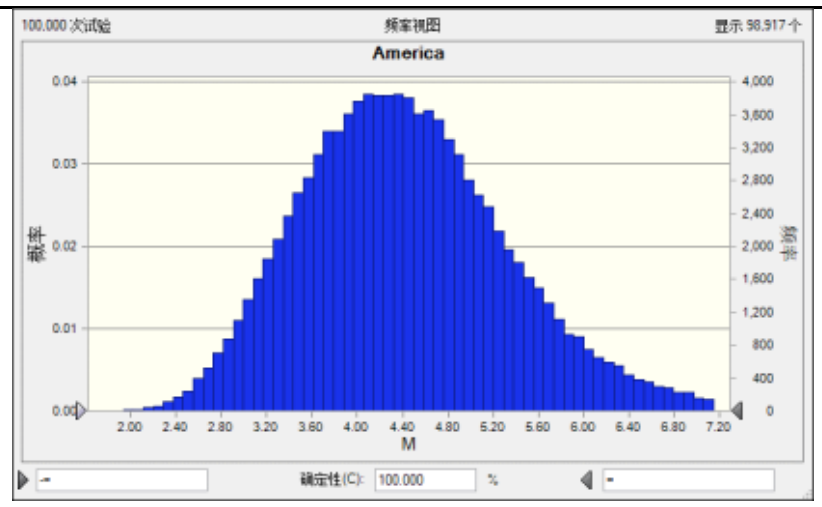	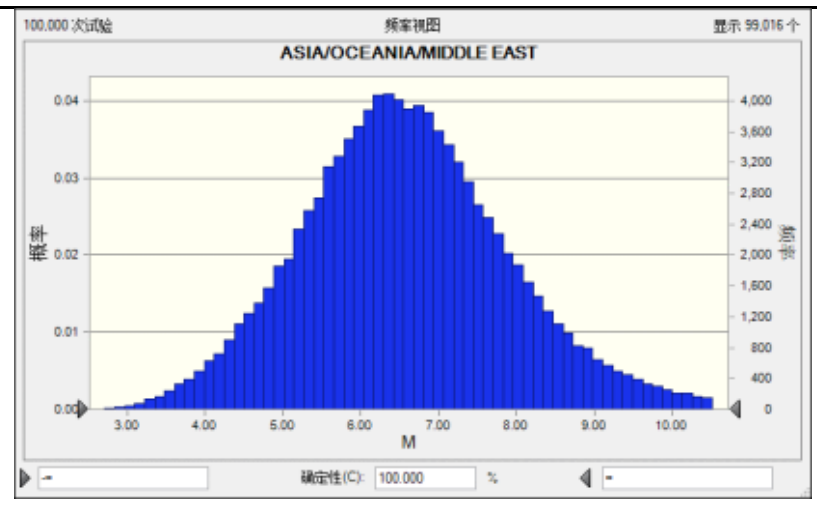	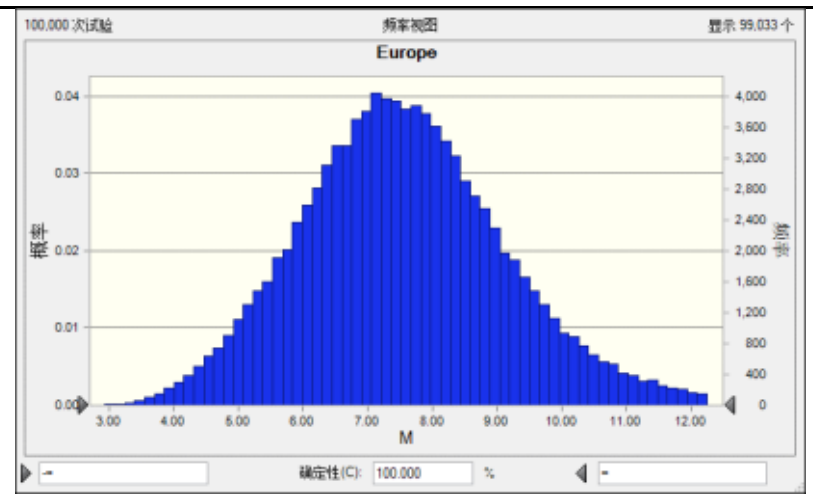	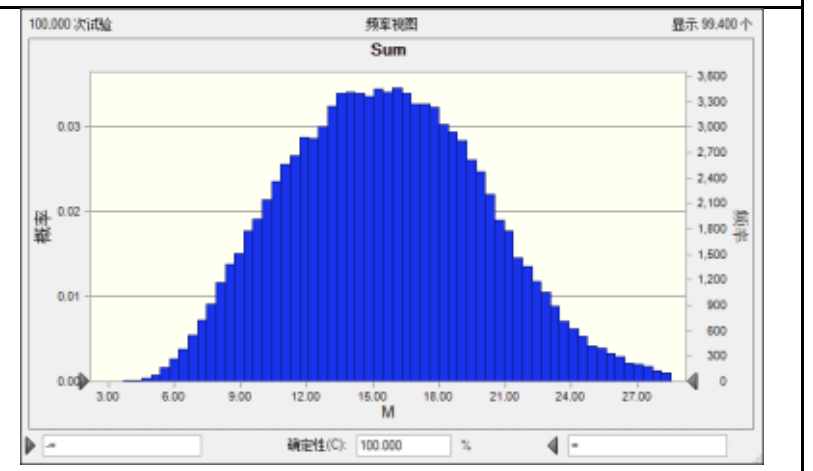
2004	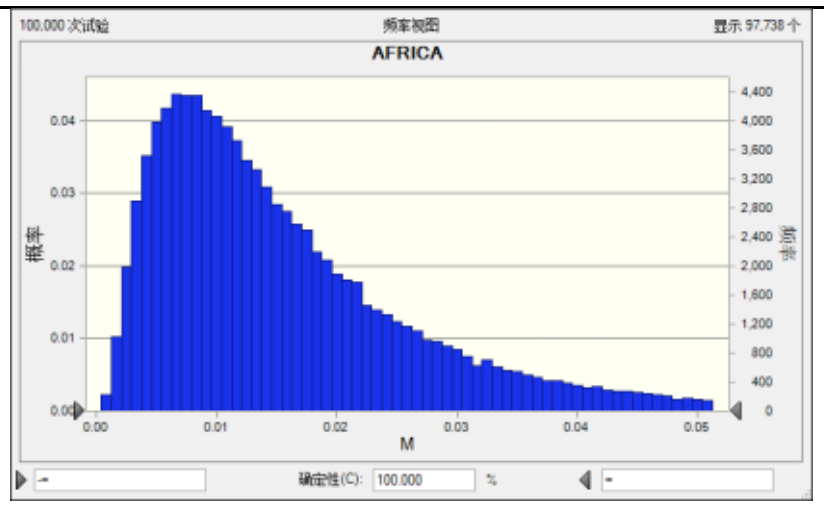	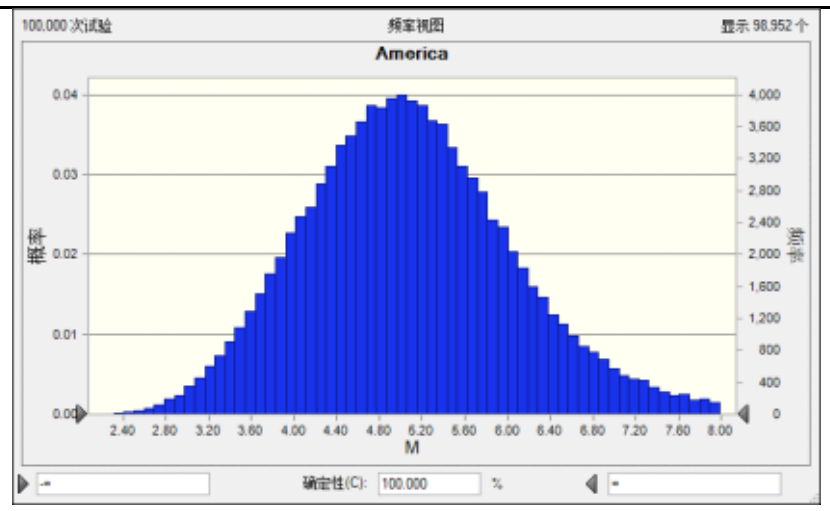	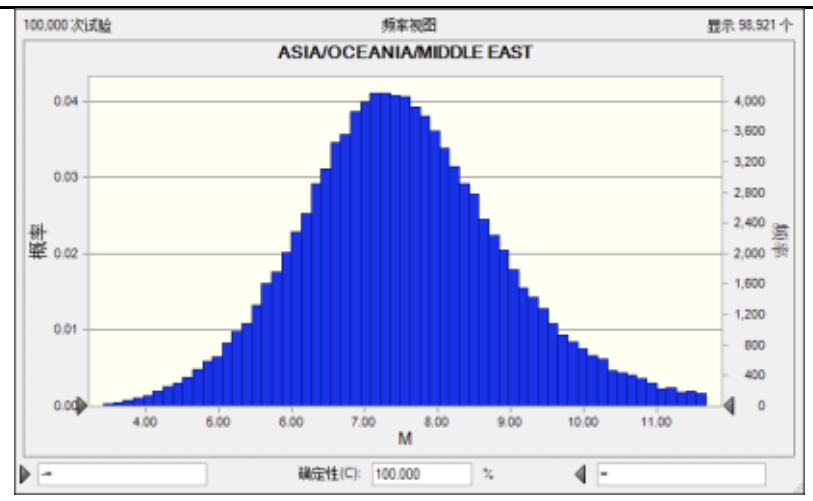	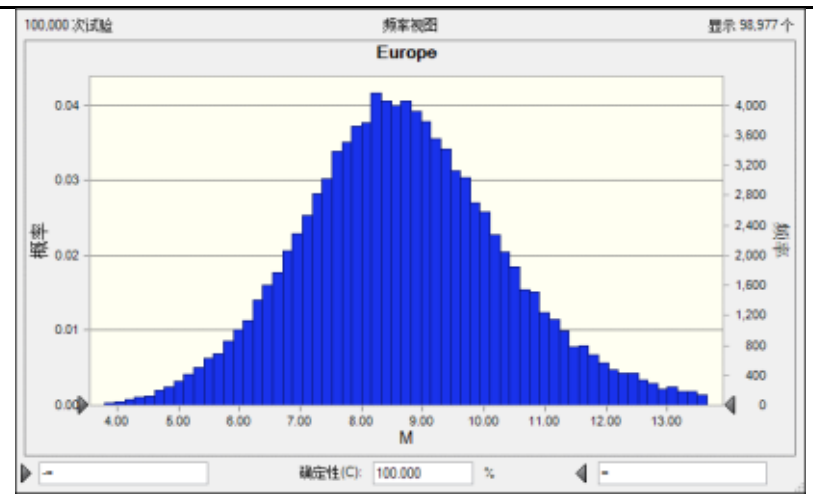	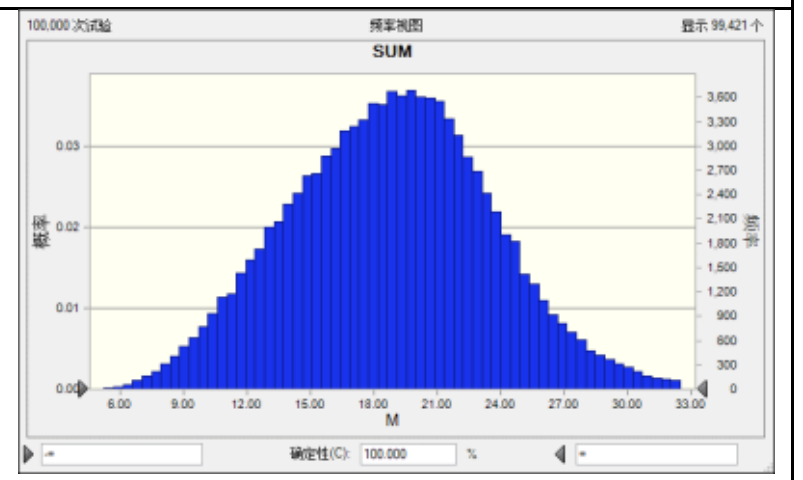

2005	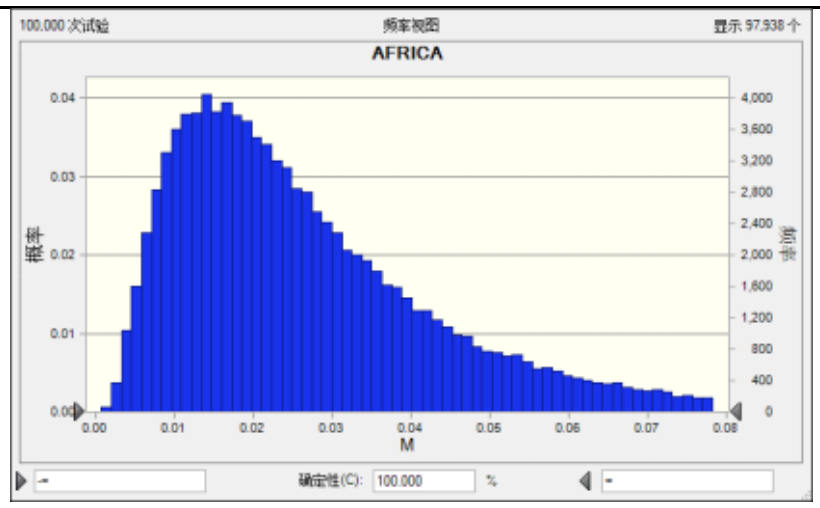	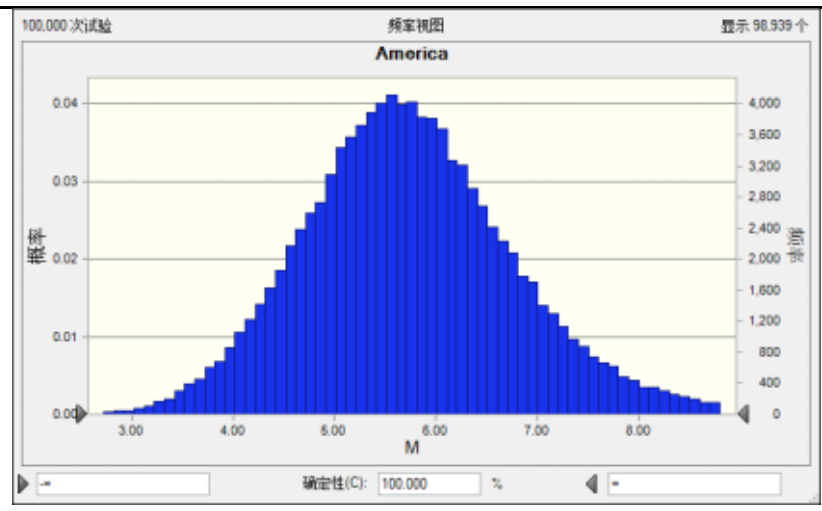	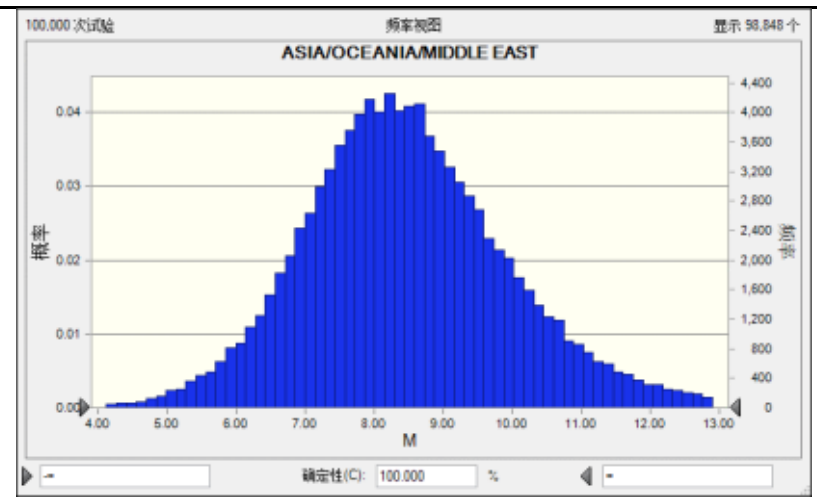	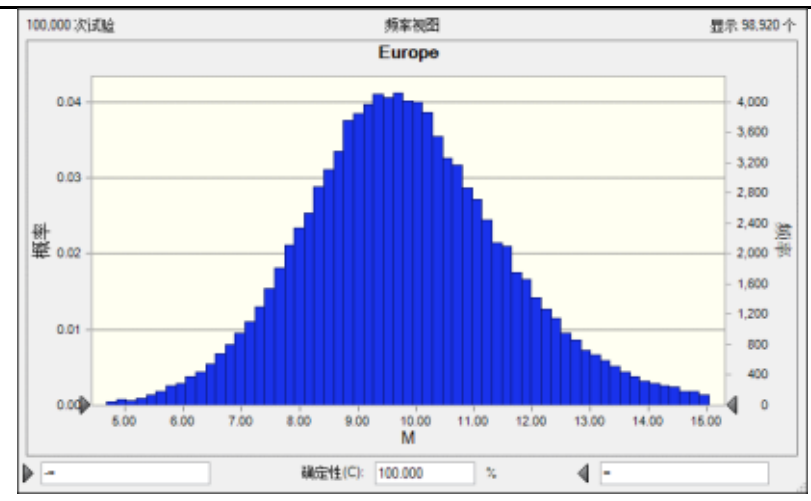	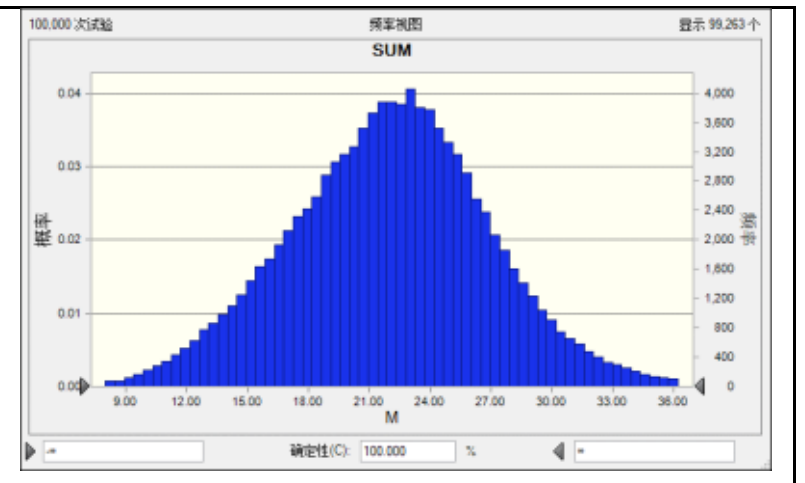
2006	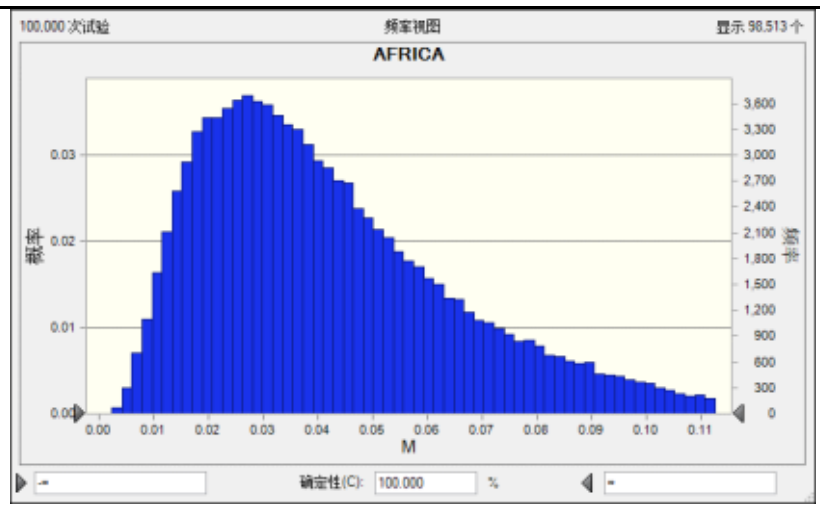	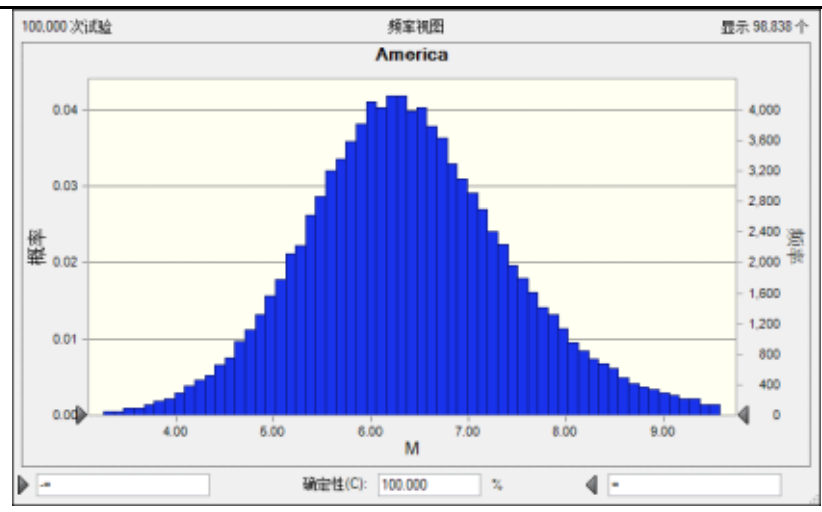	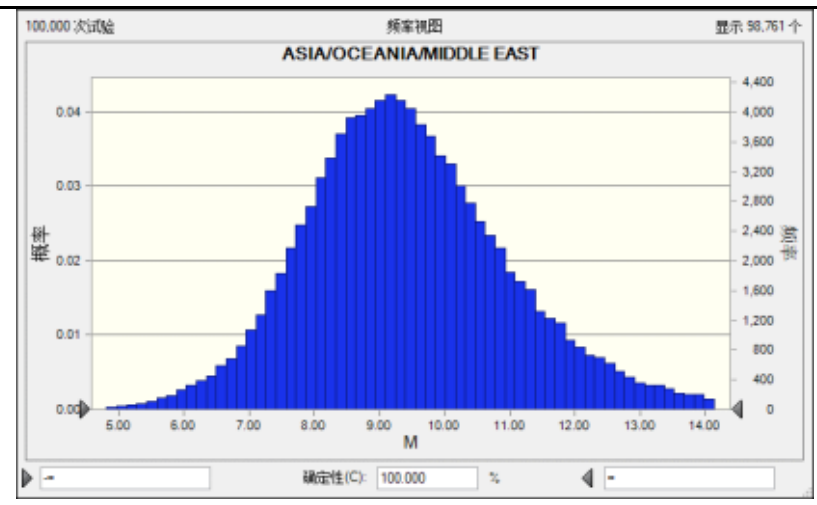	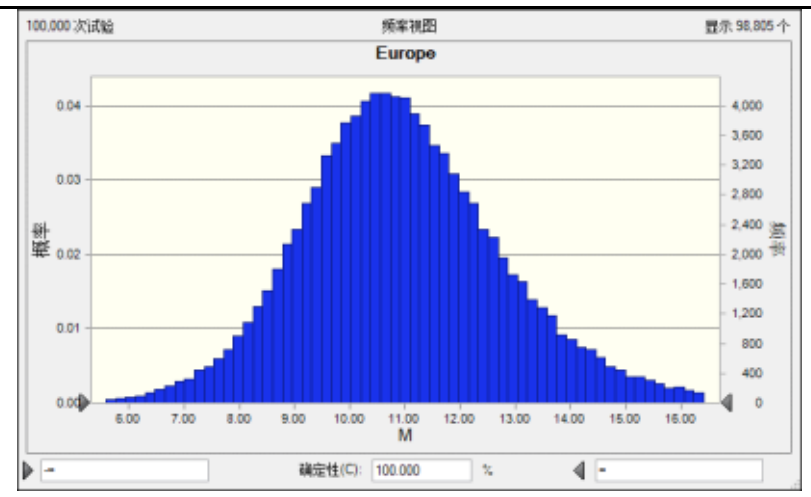	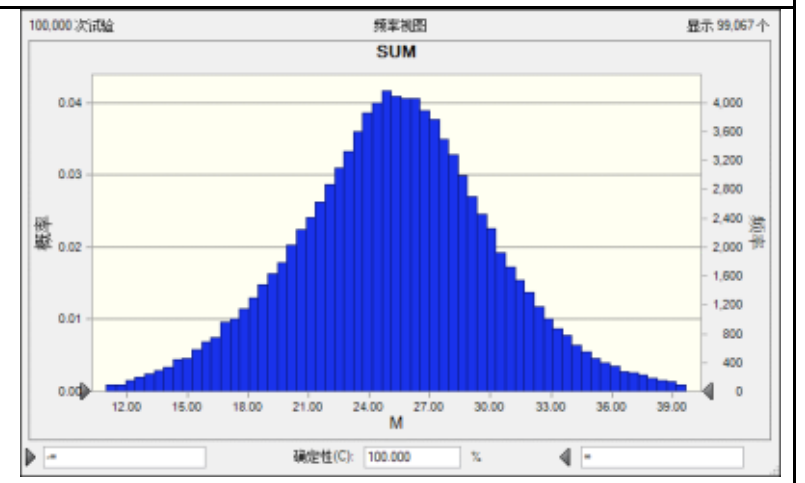
2007	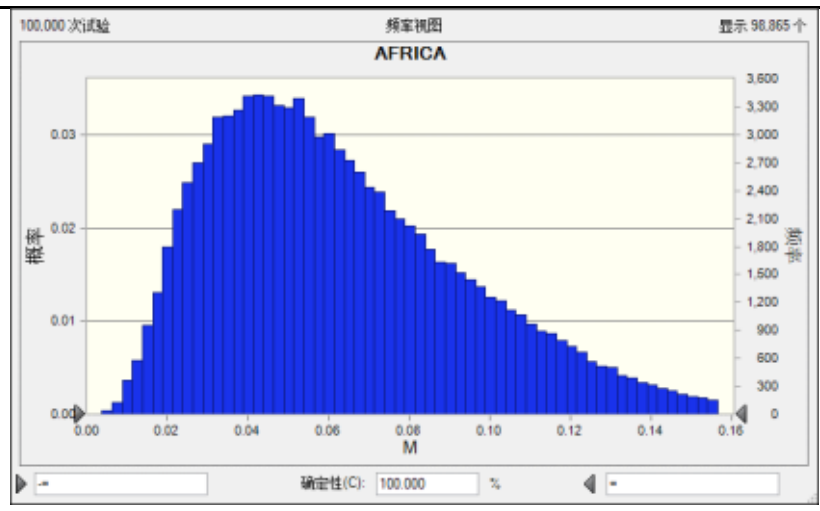	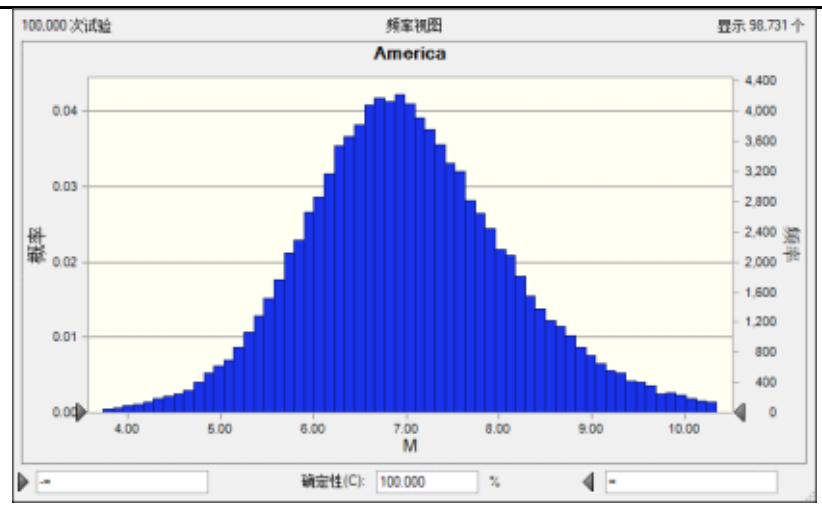	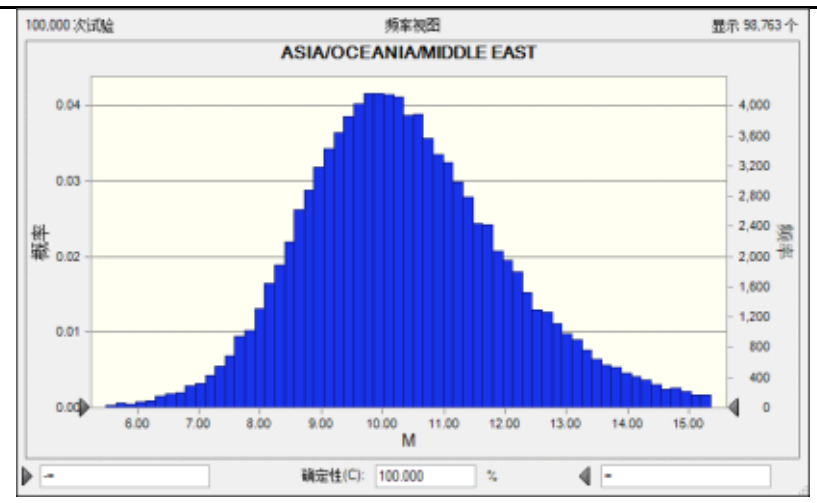	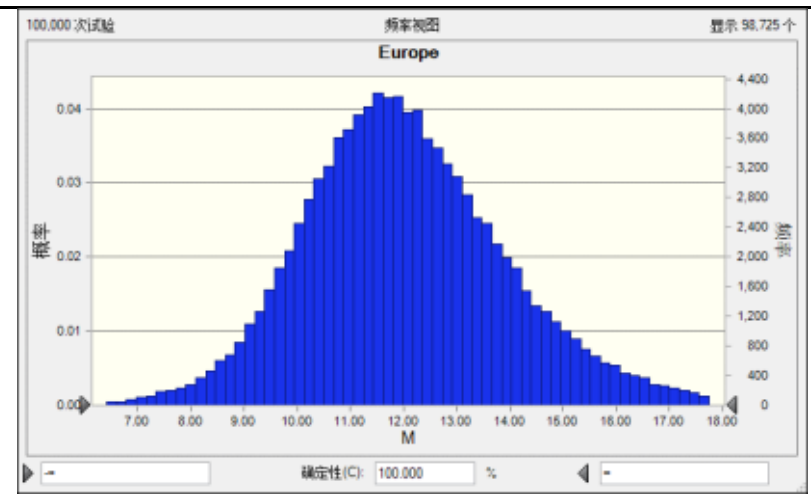	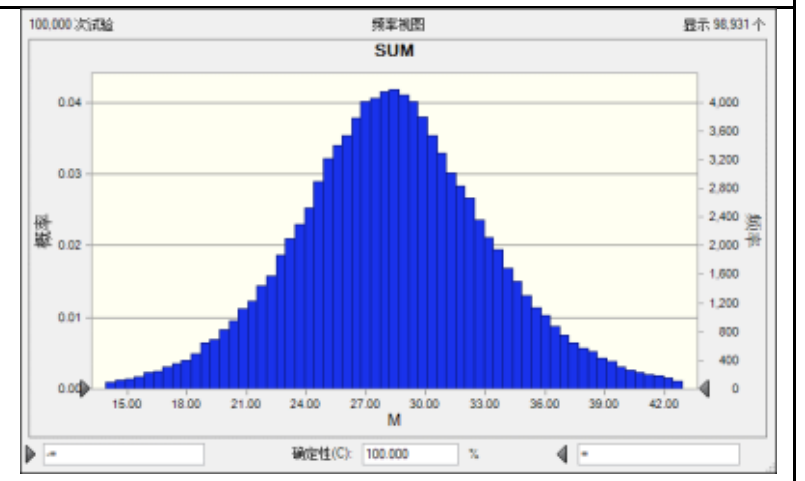
2008	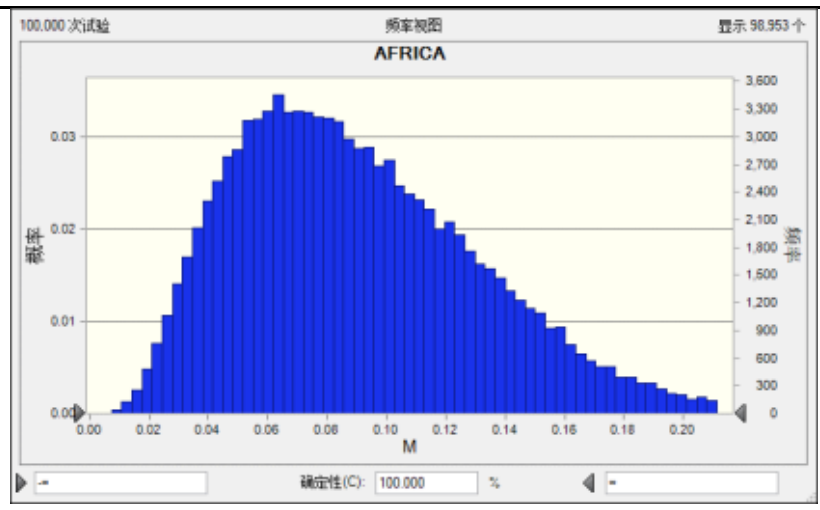	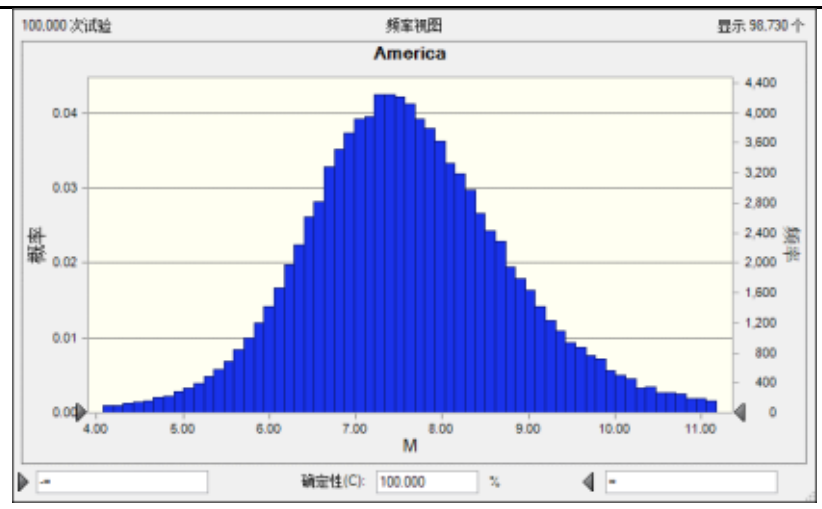	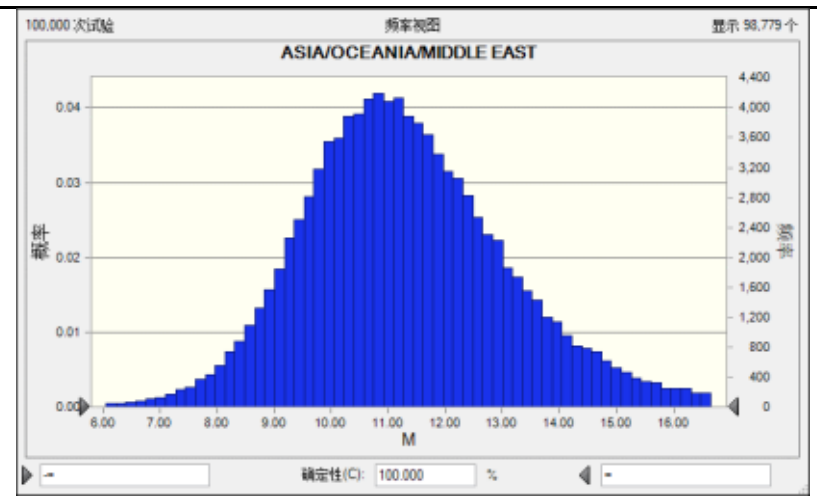	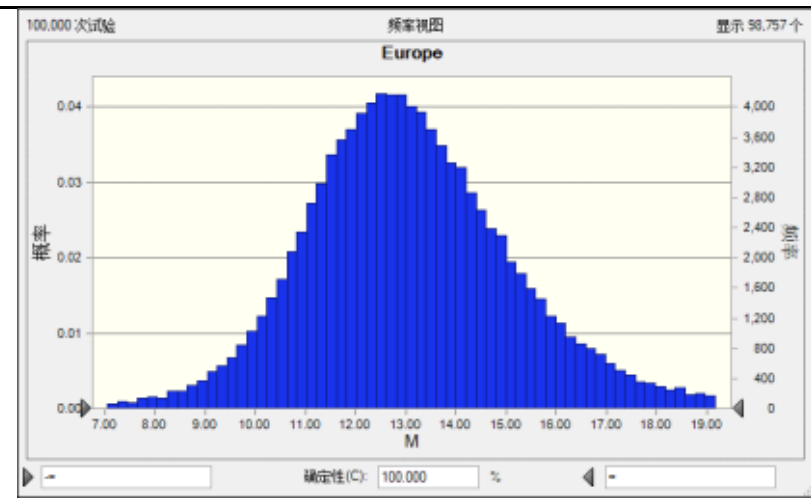	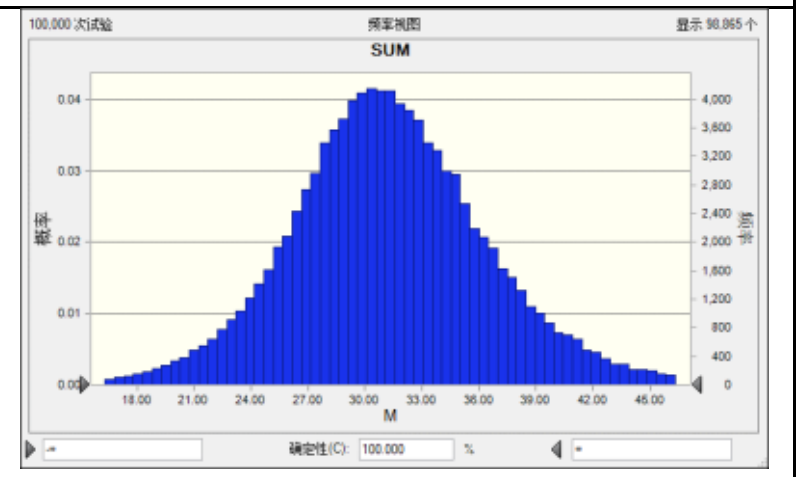
2009	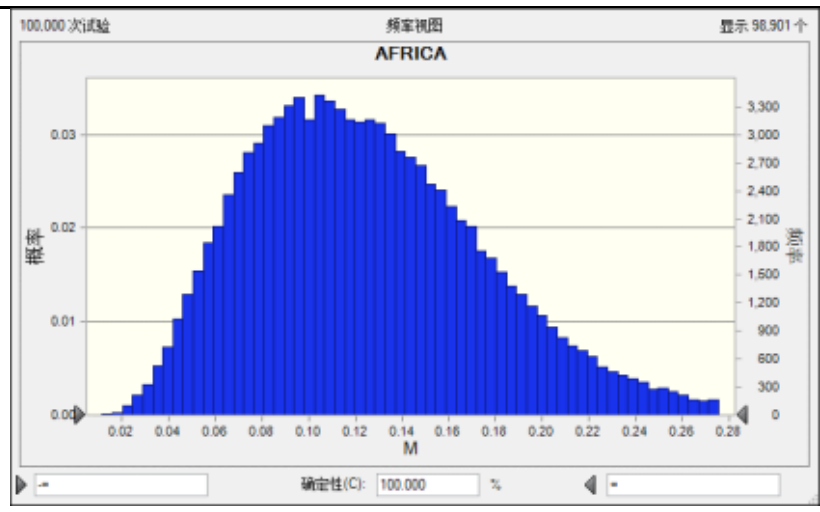	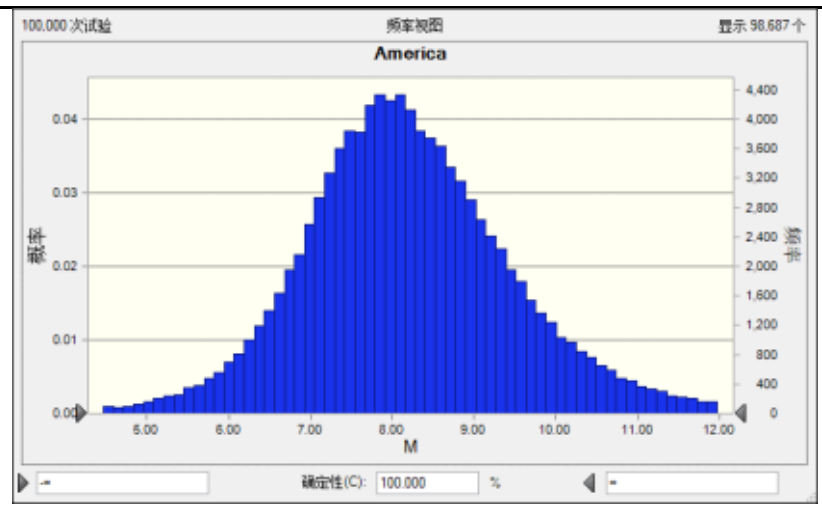	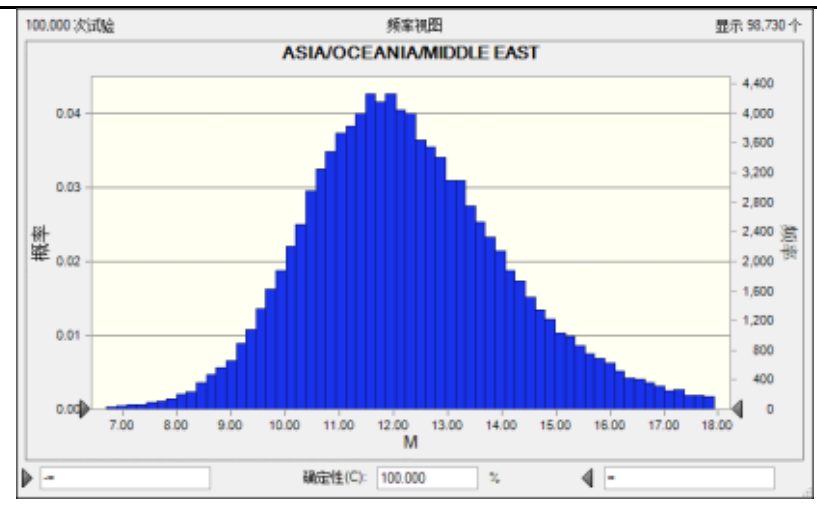	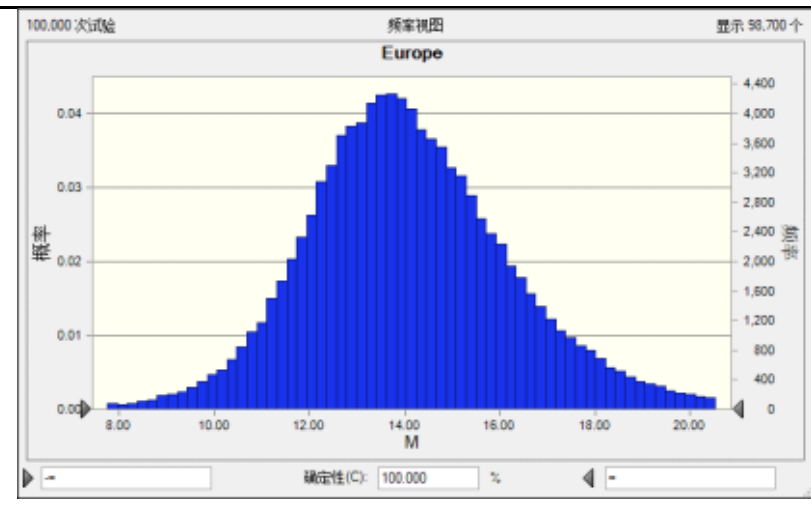	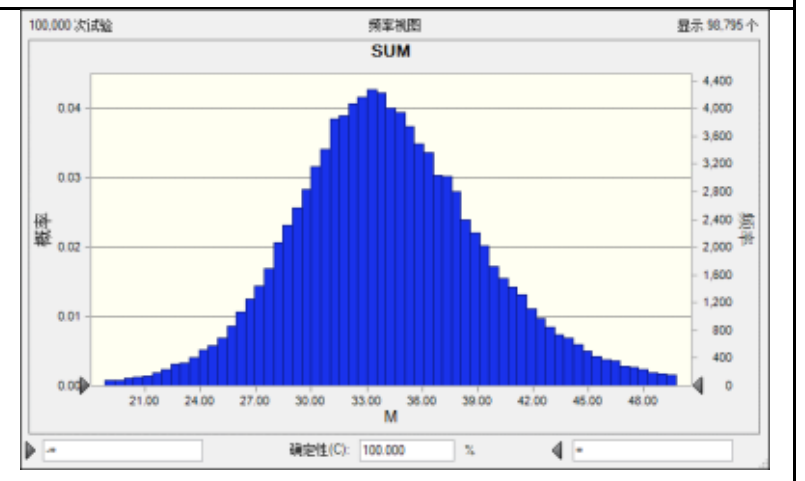

2010

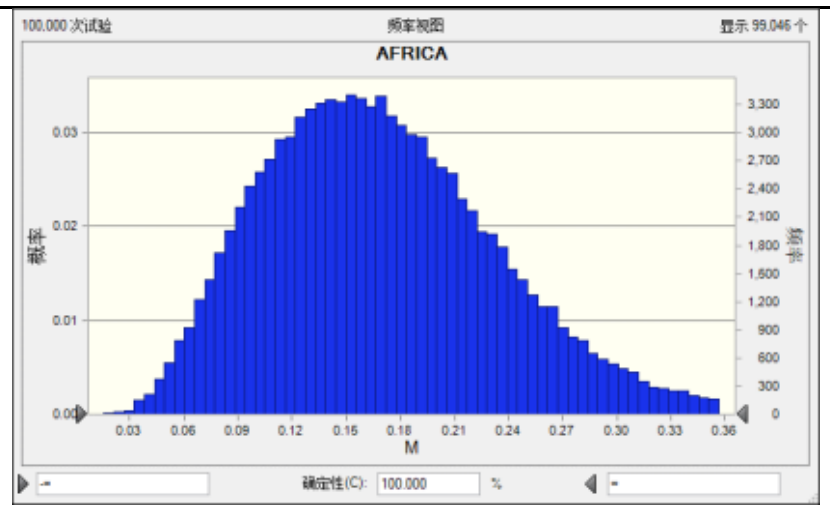

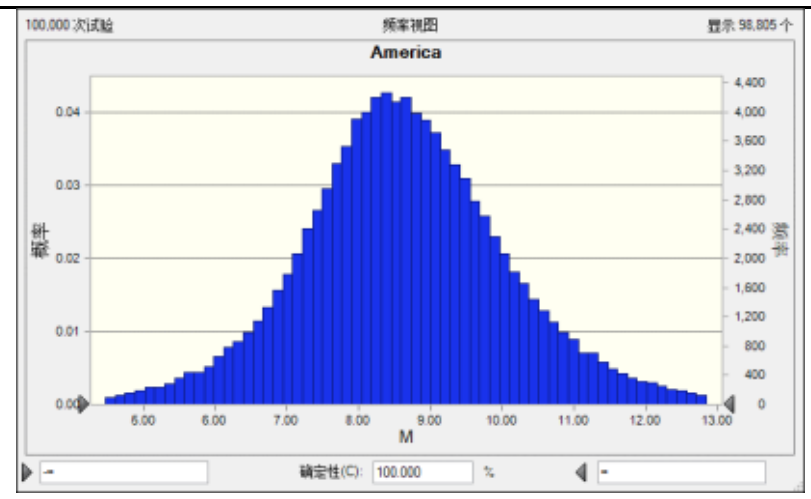

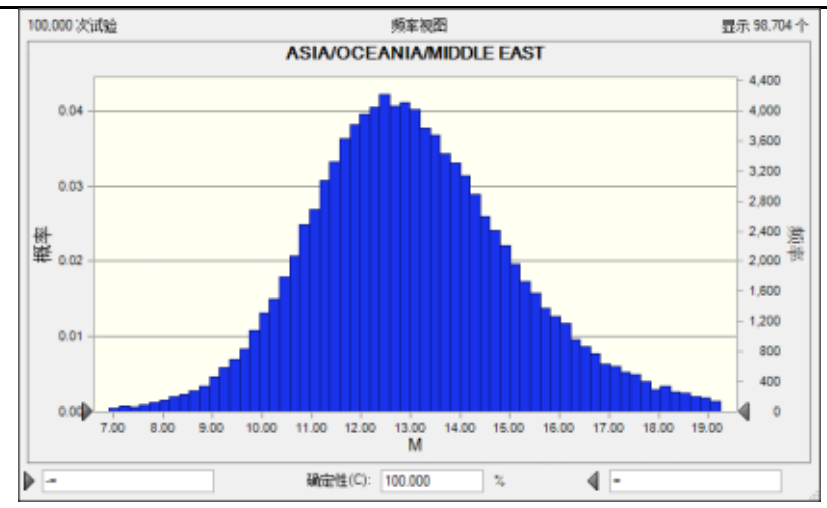

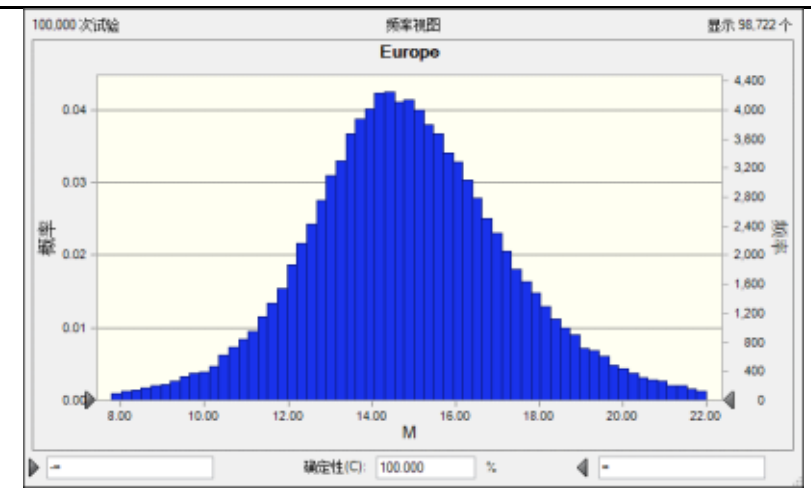

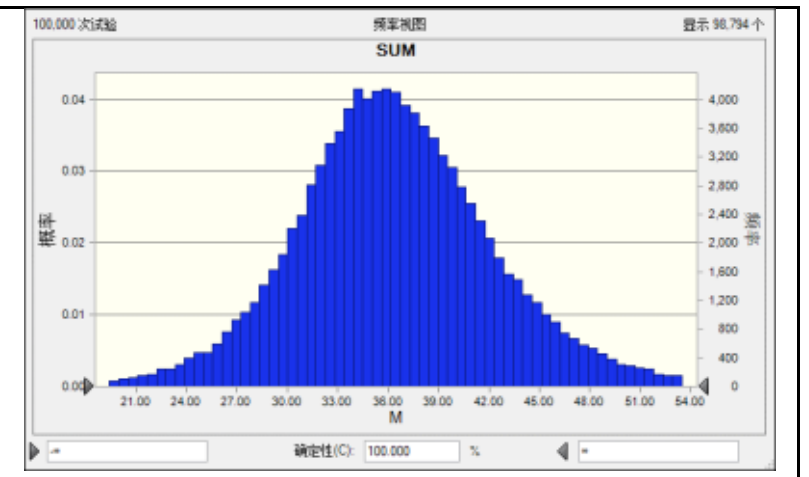

2011

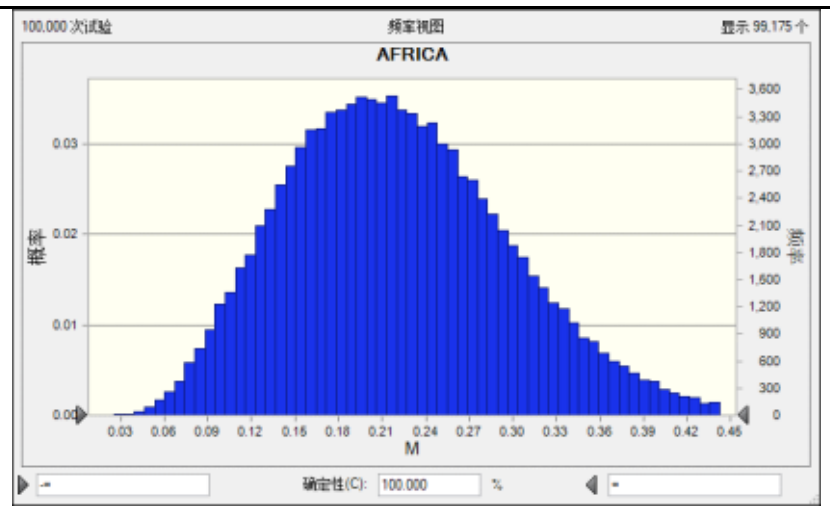

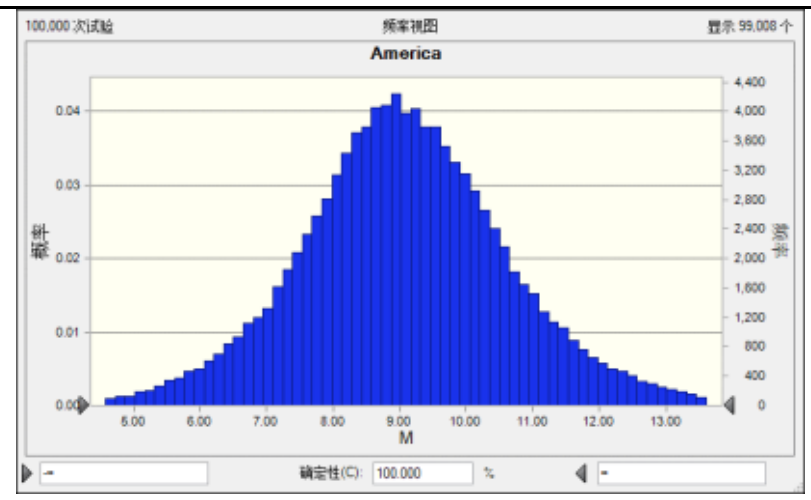

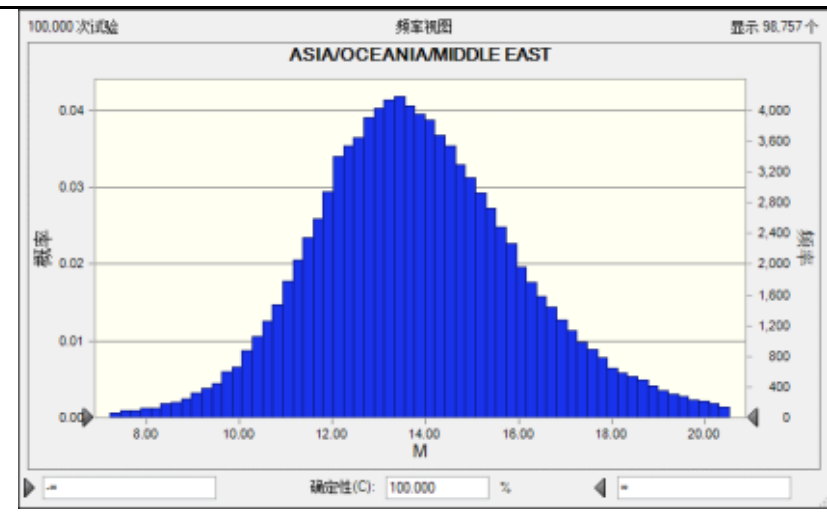

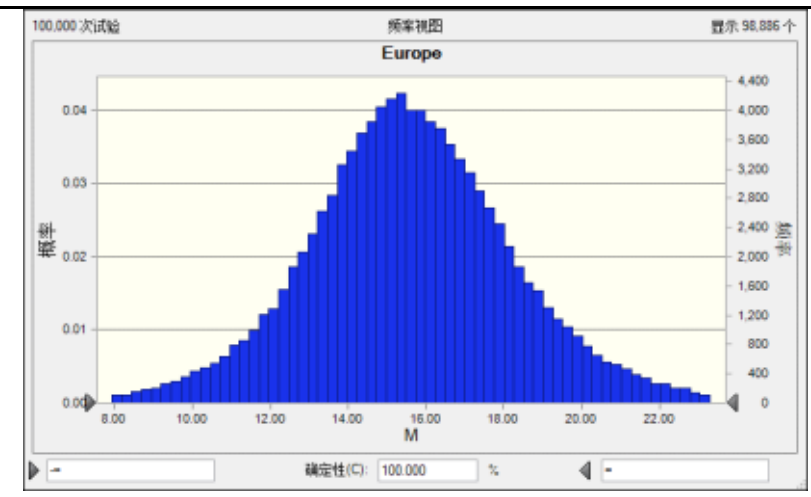

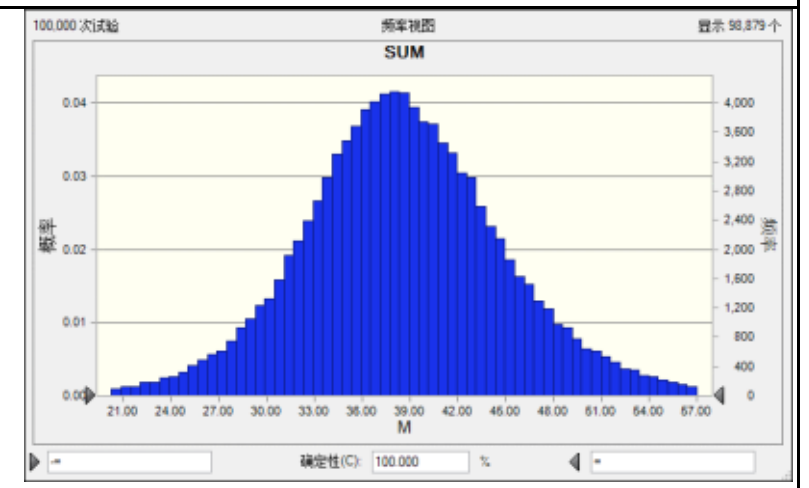

2012

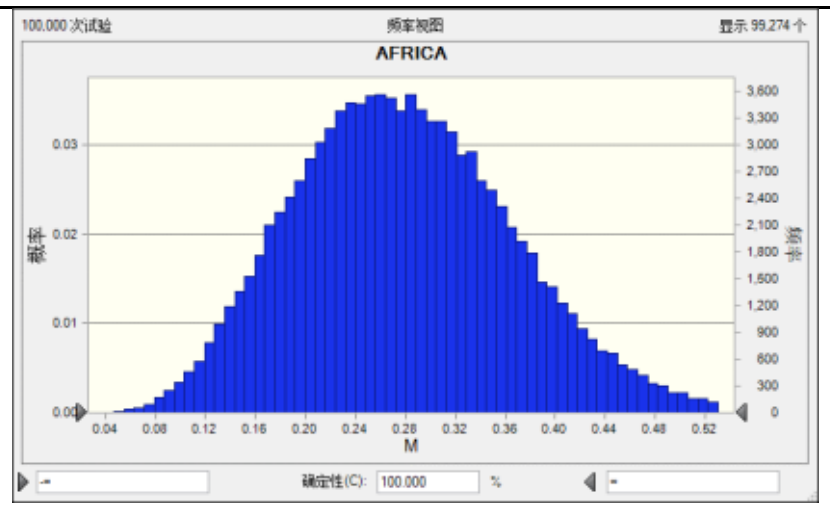

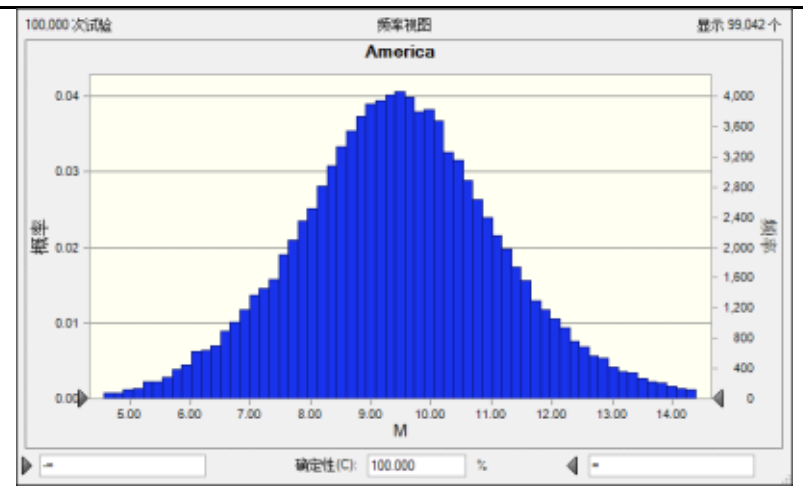

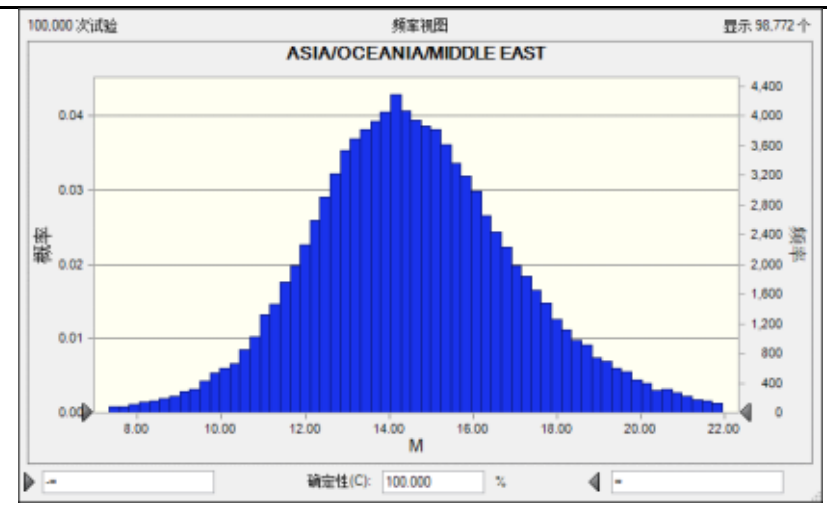

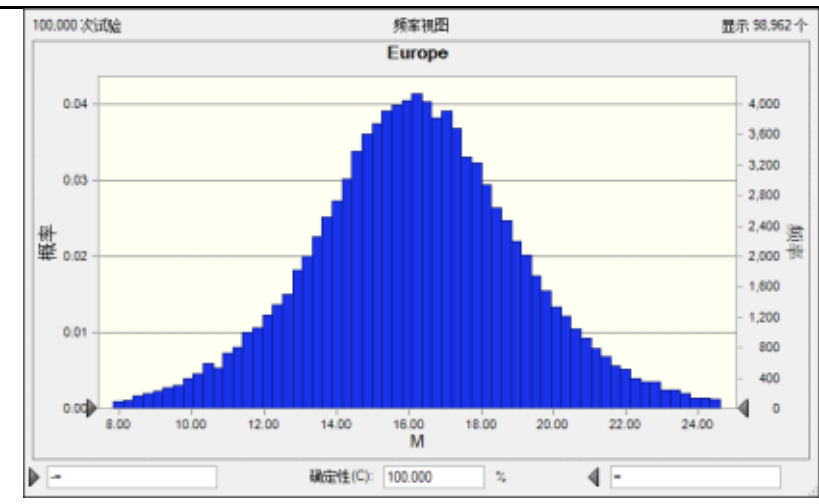

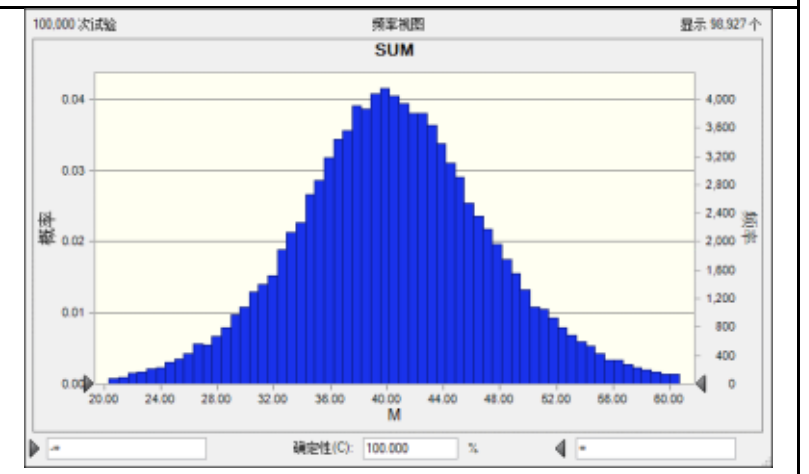

2013

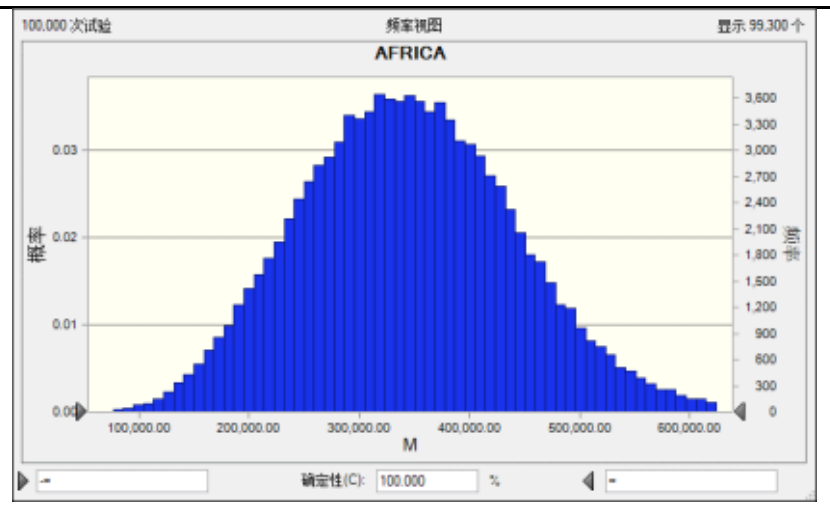

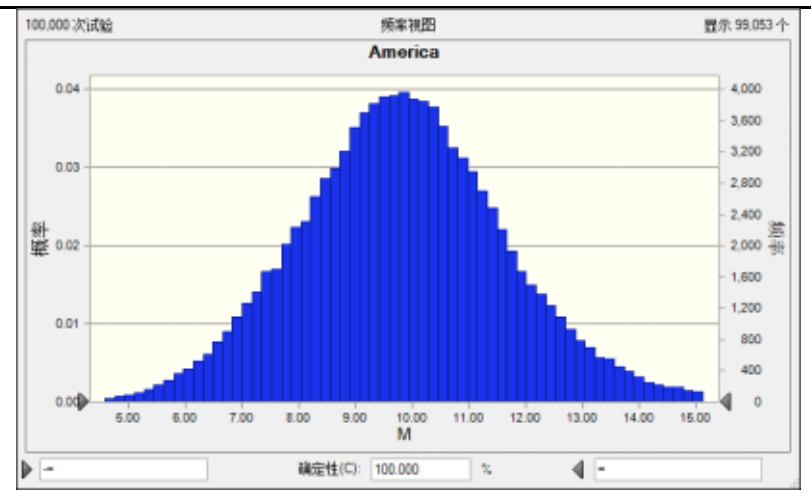

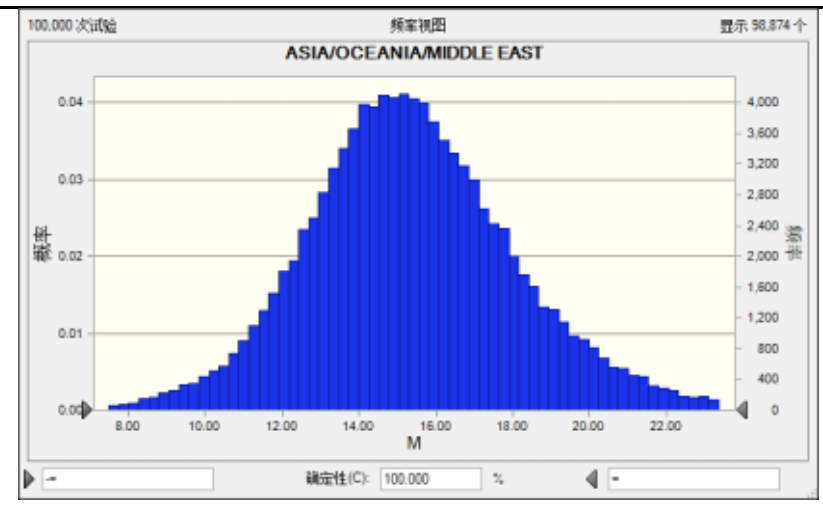

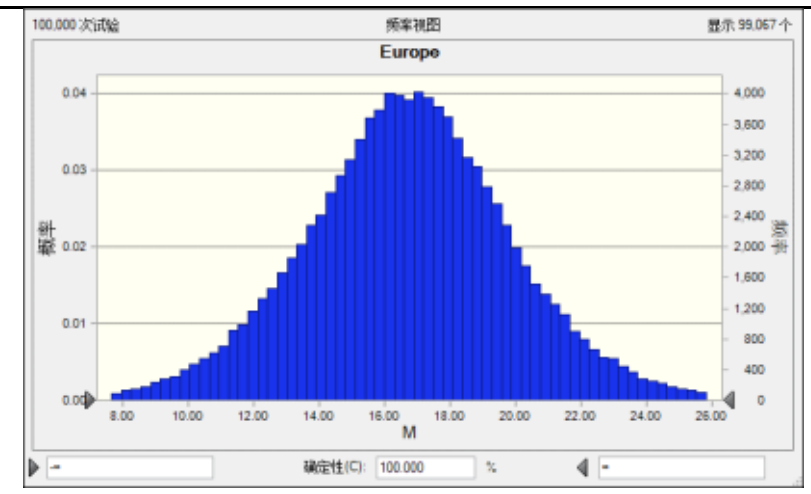

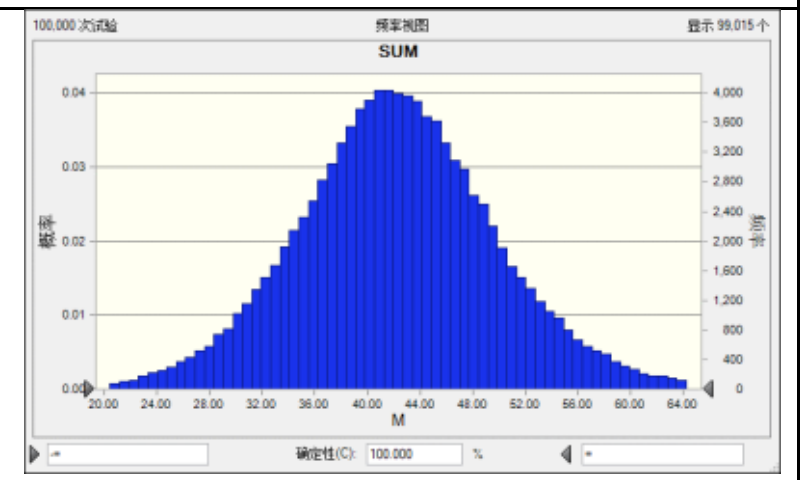

2014

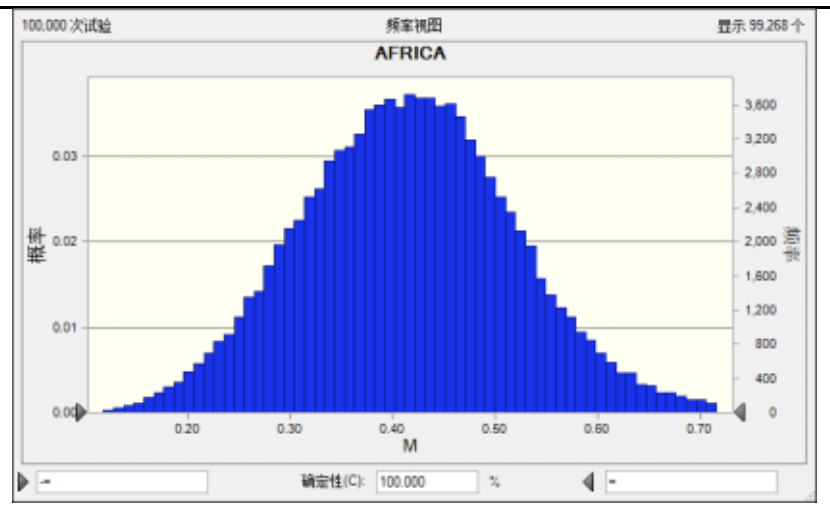

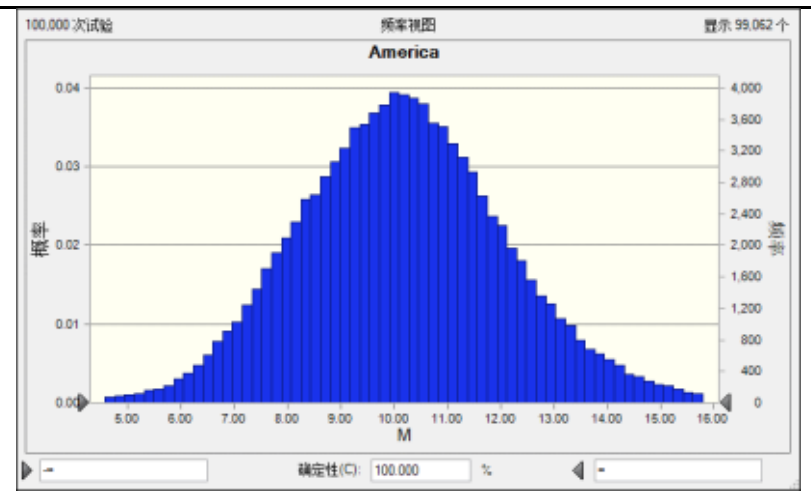

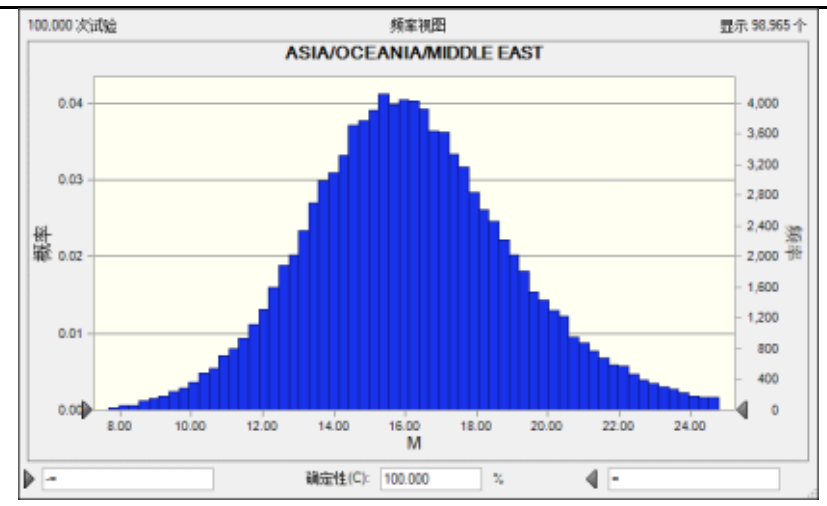

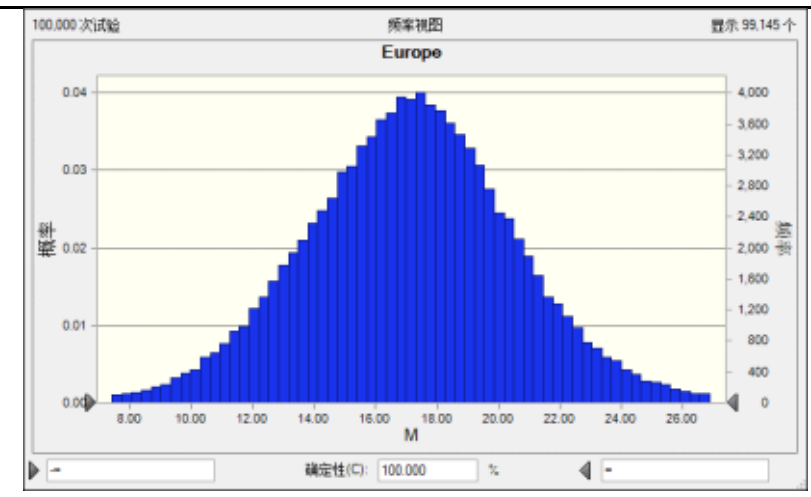

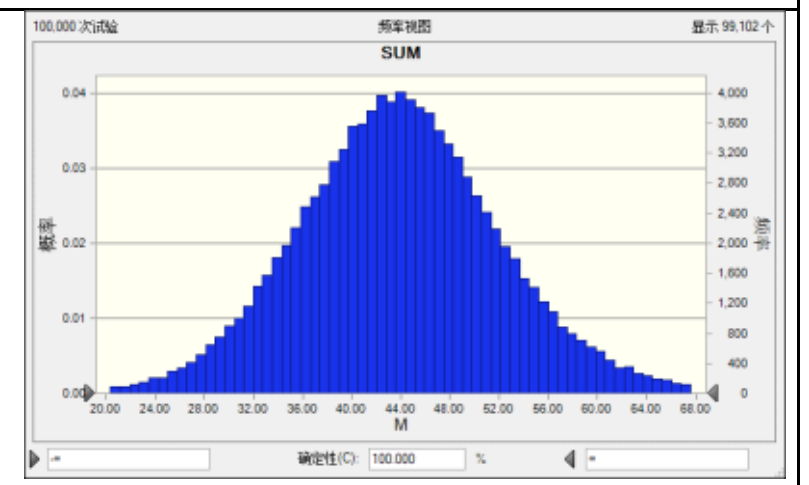

2015	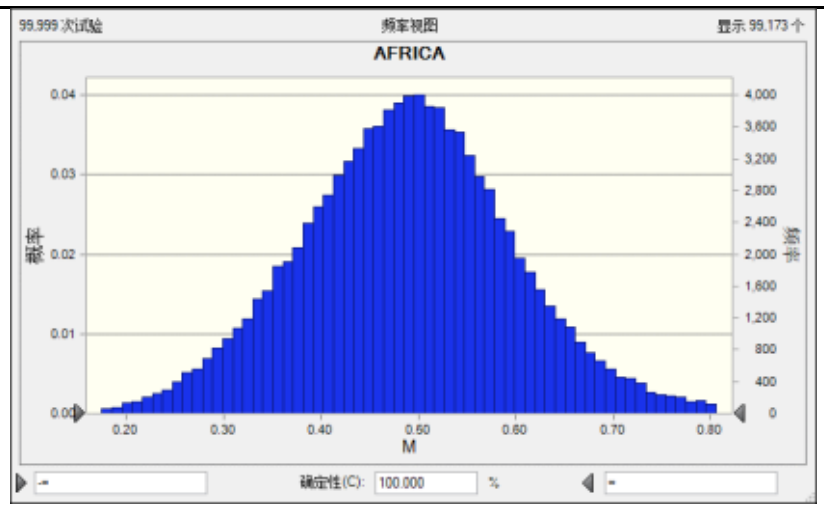	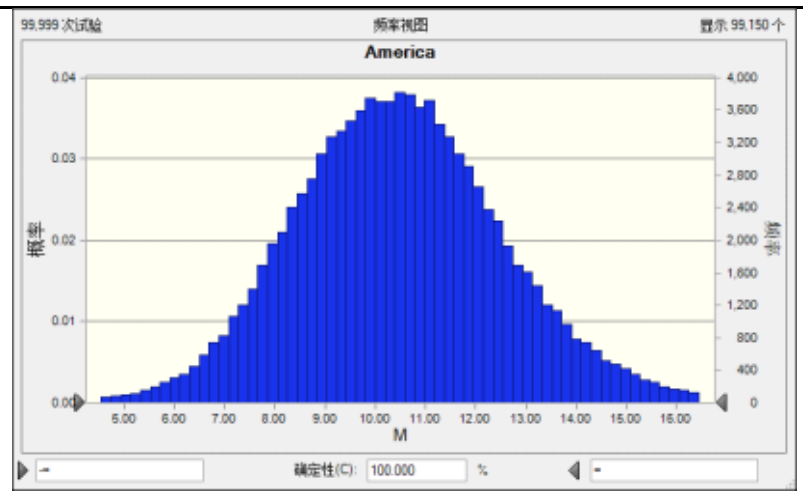	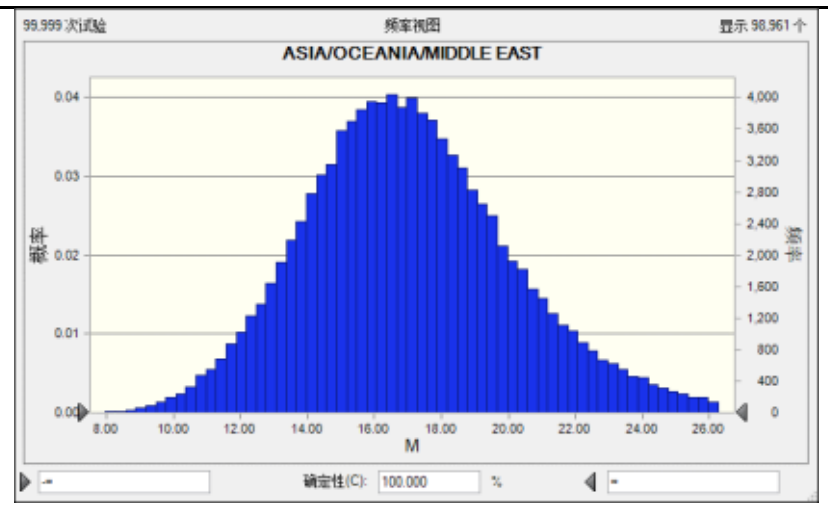	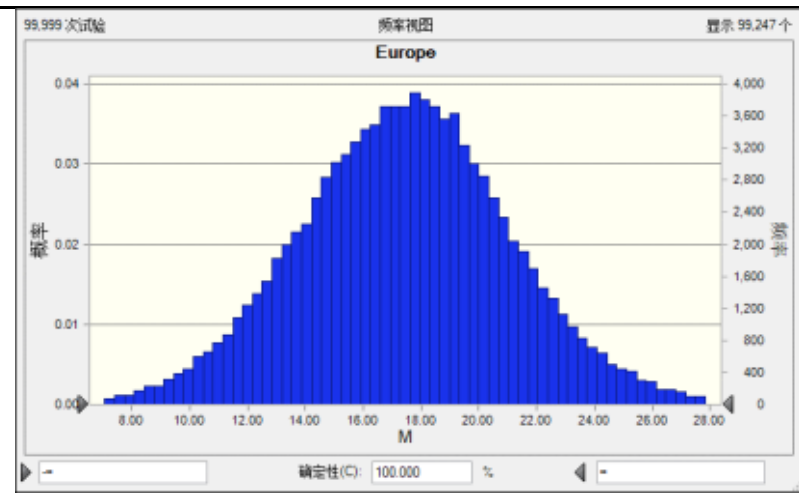	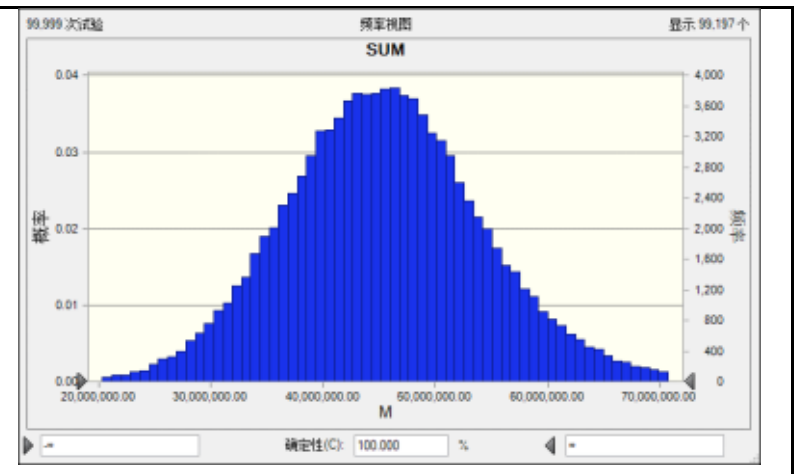
2016	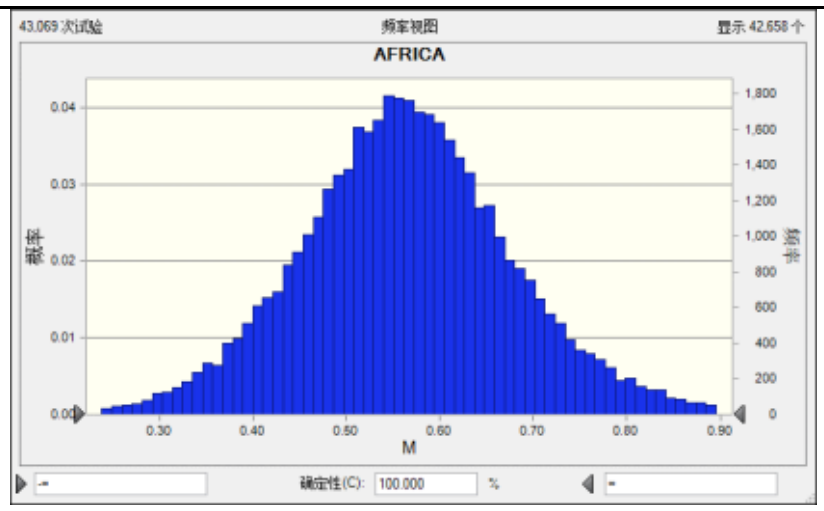	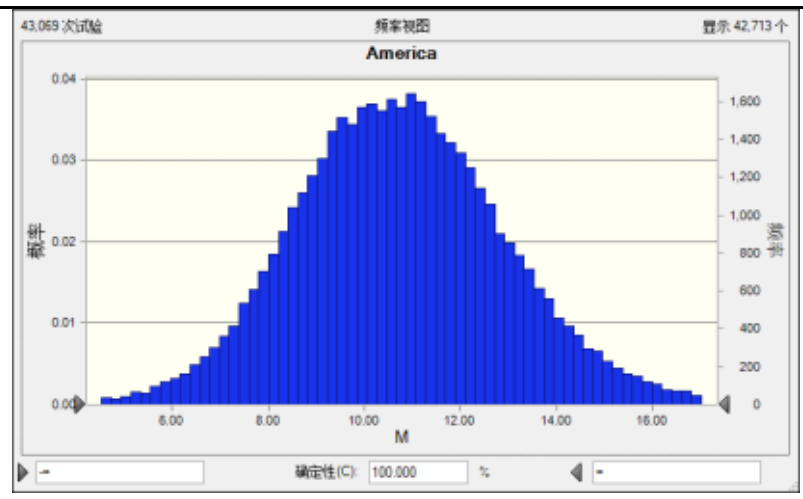	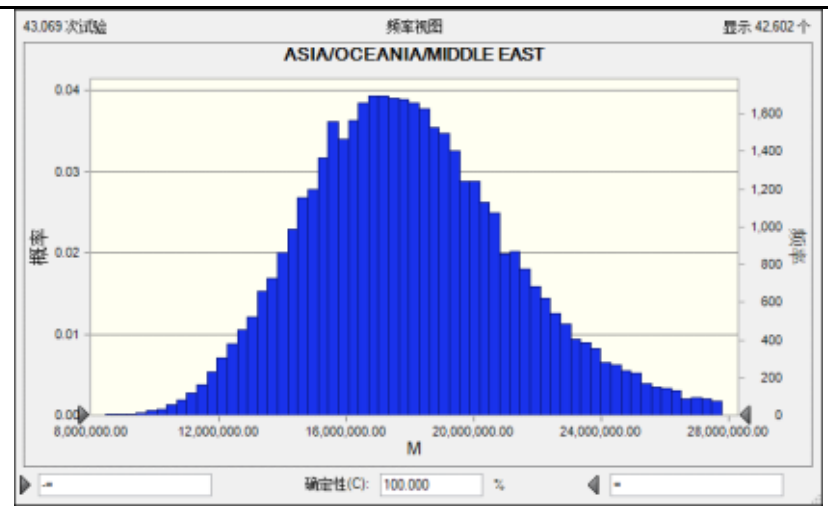	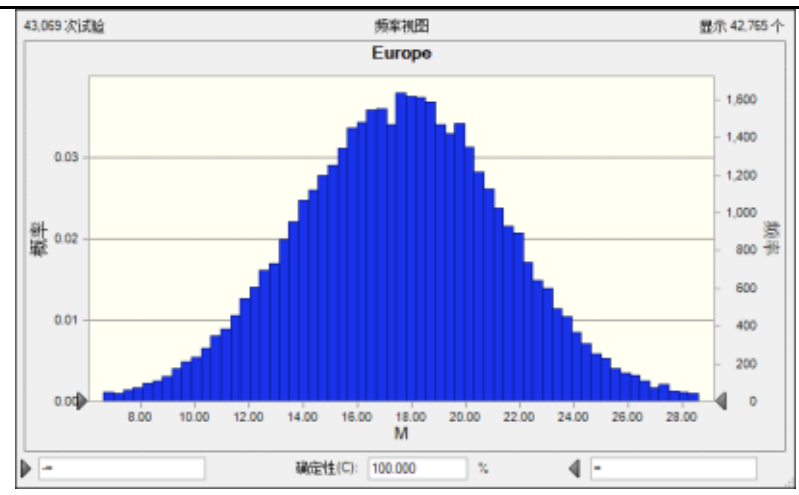	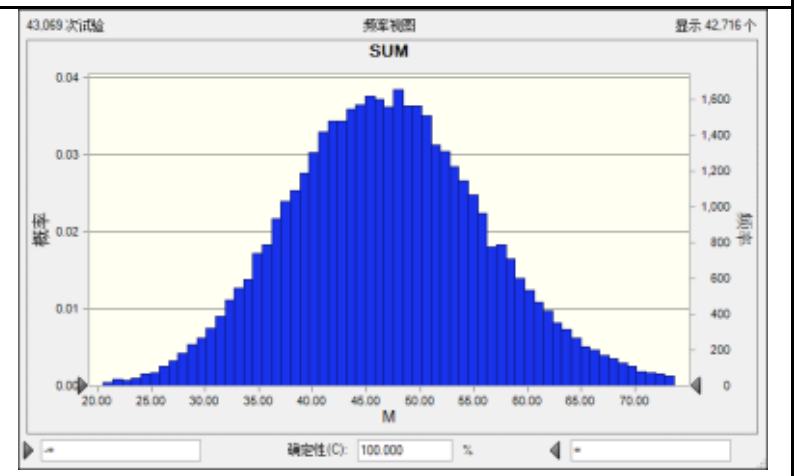
2017	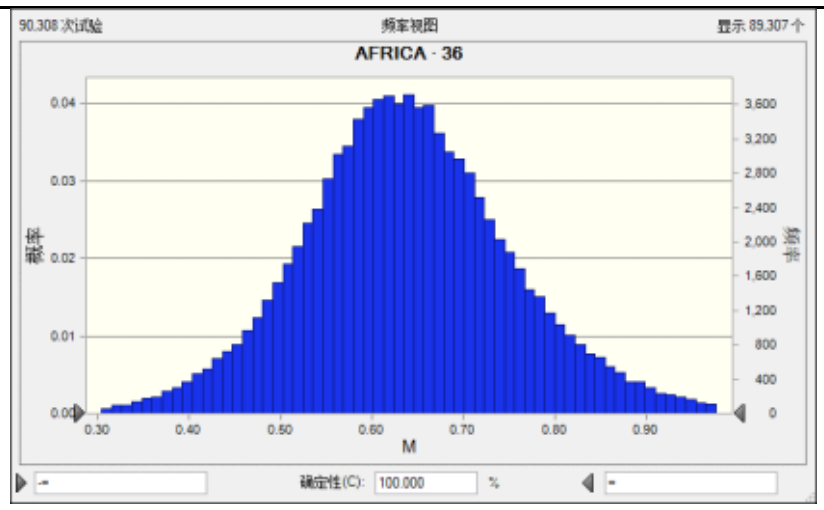	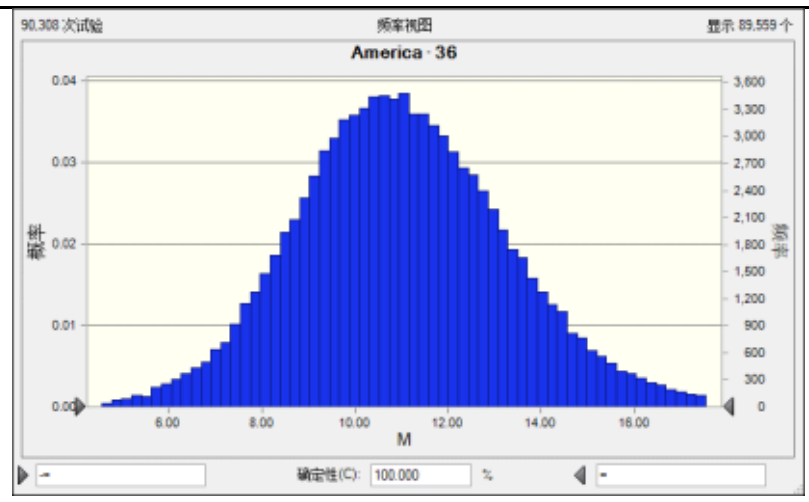	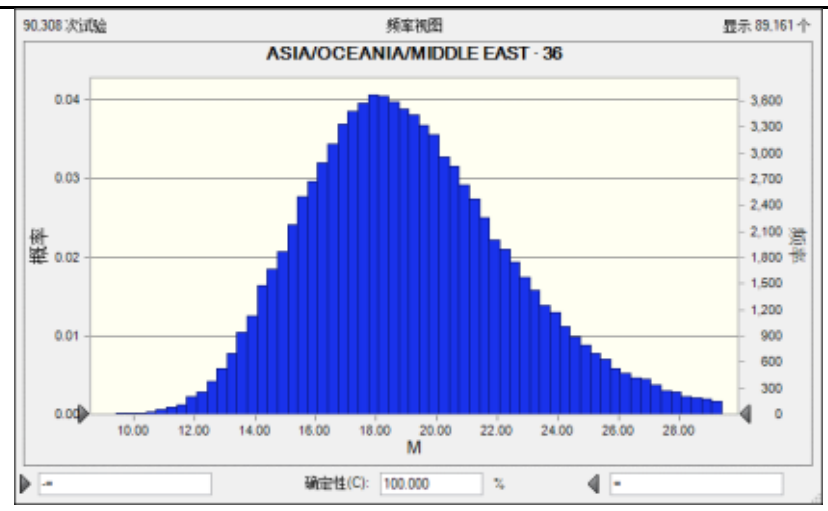	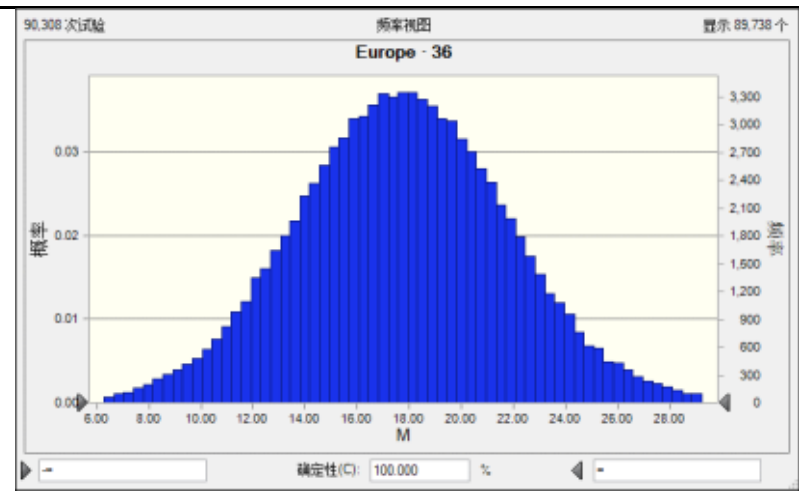	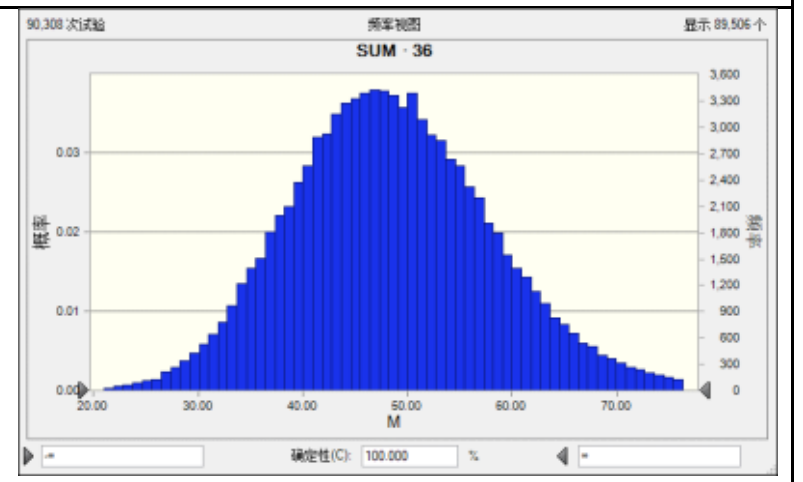
2018	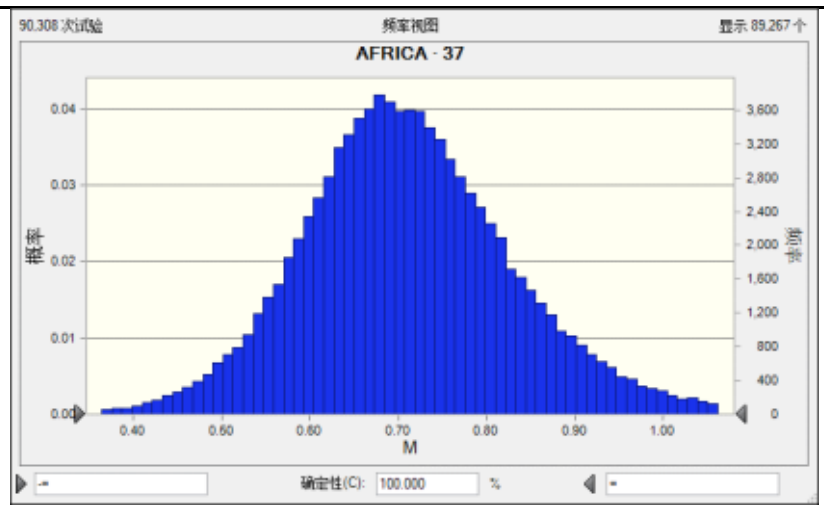	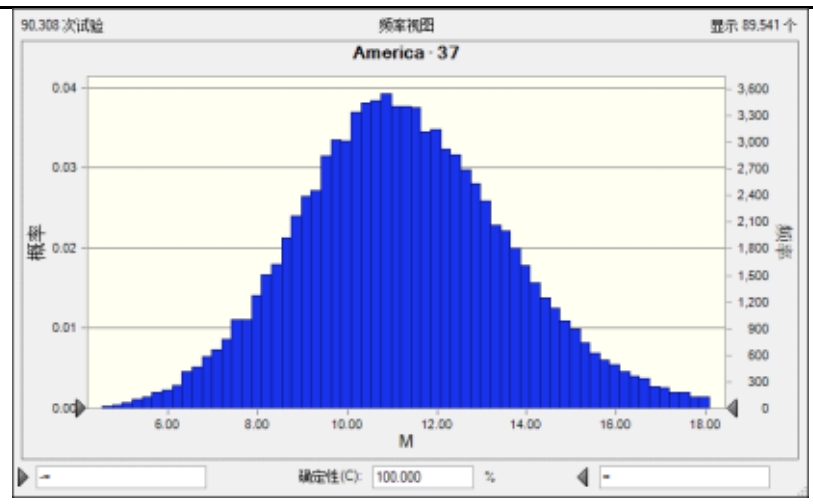	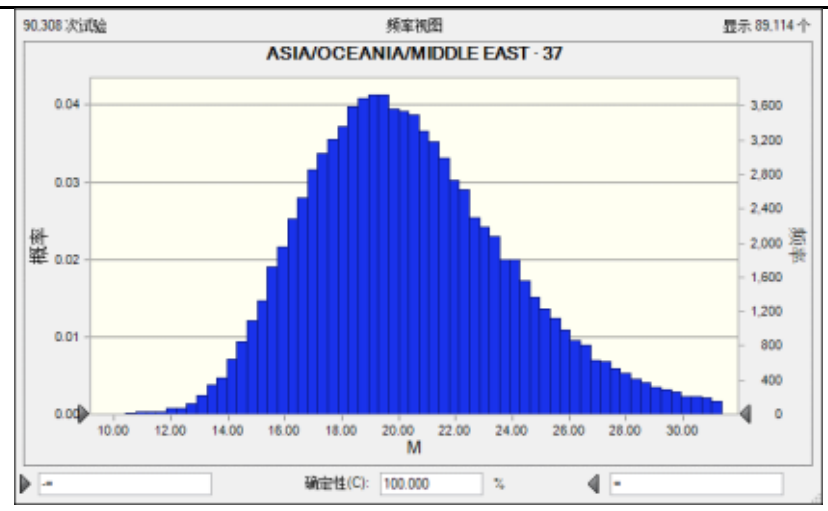	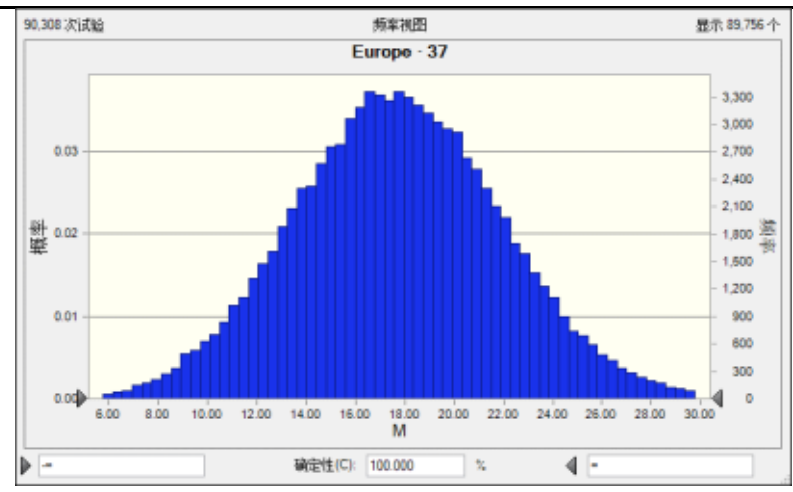	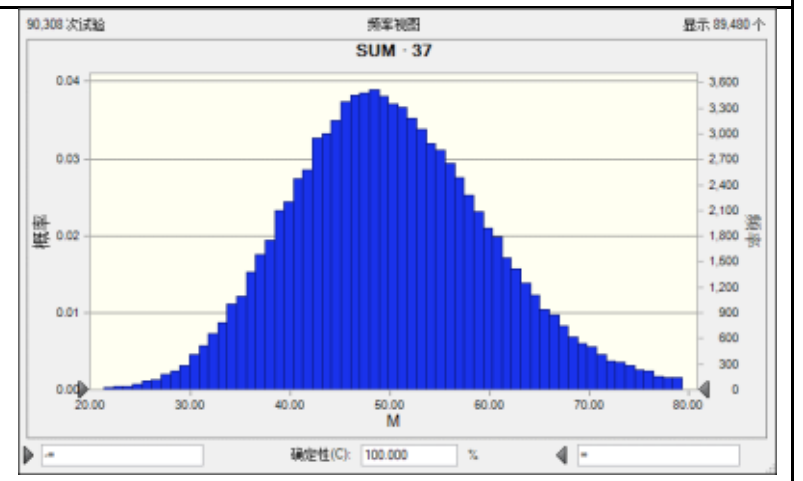
2019	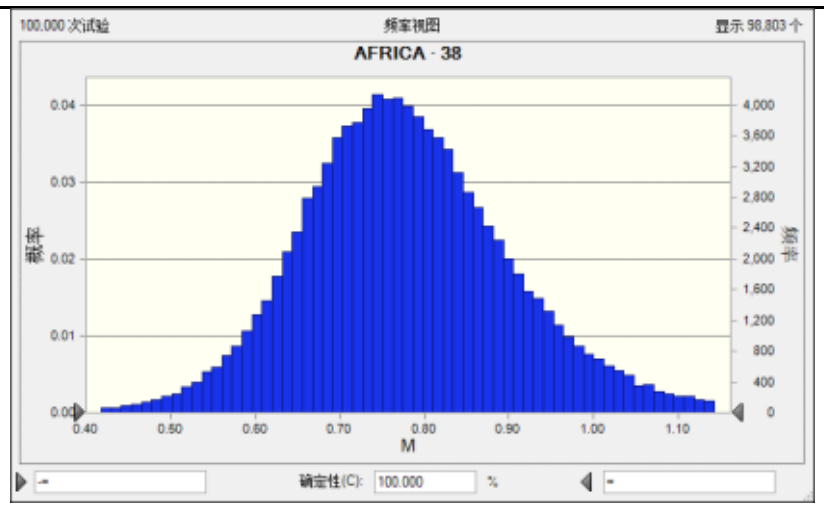	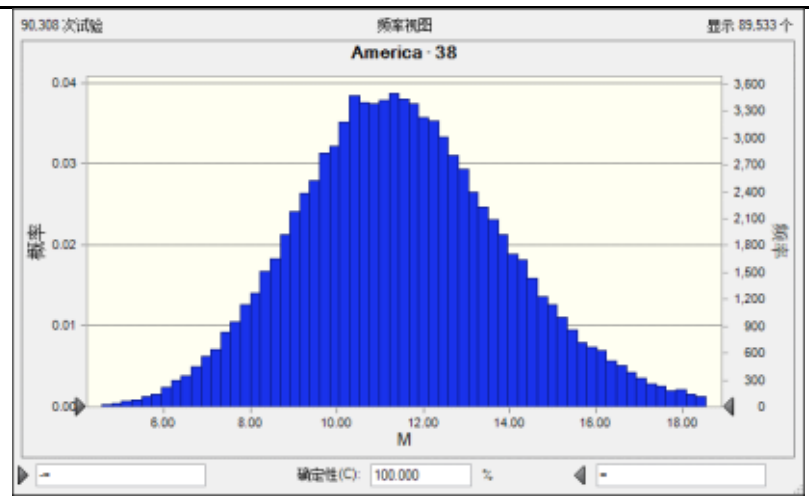	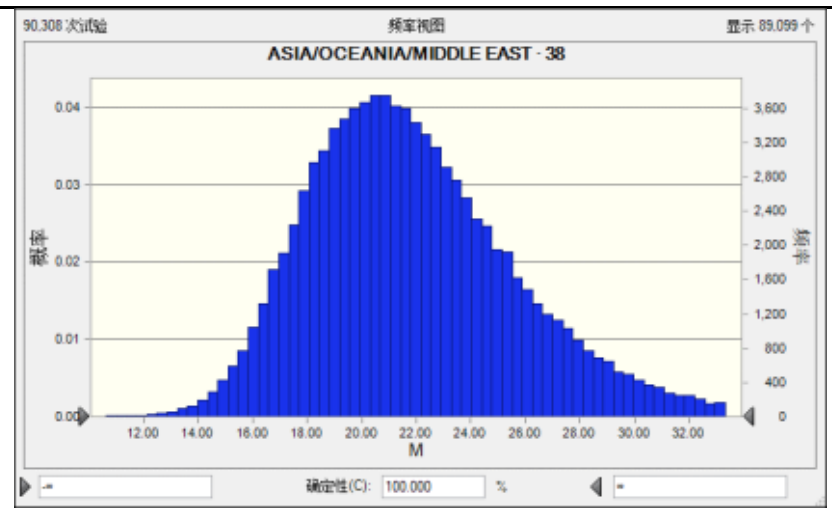	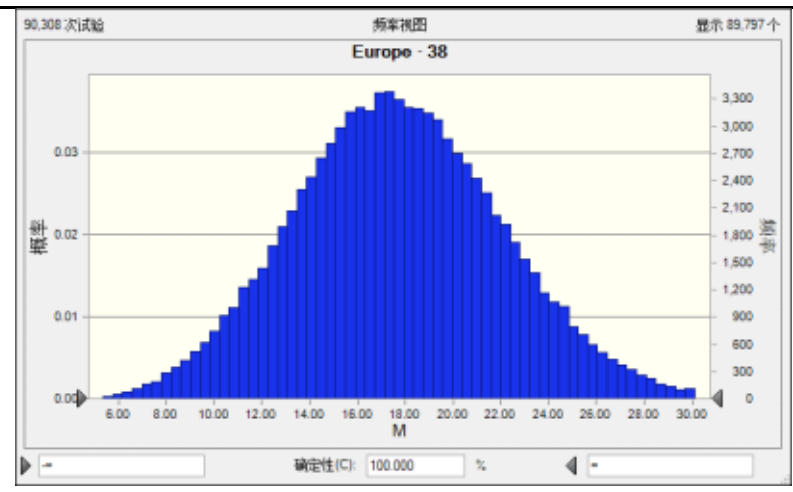	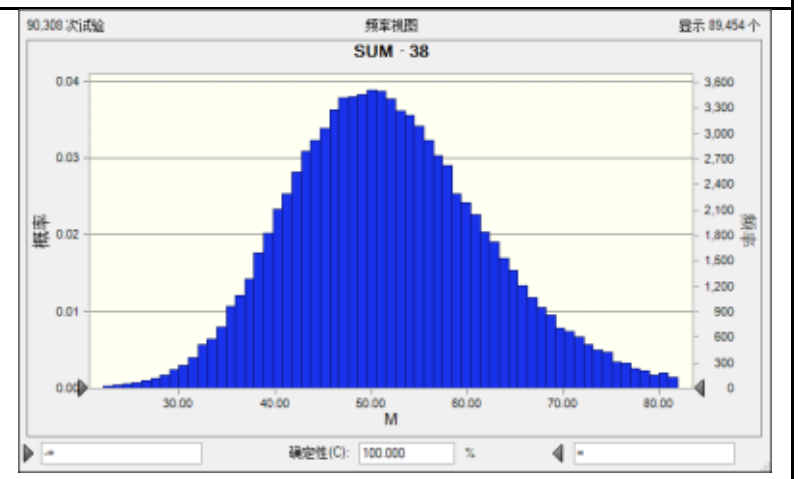

2020	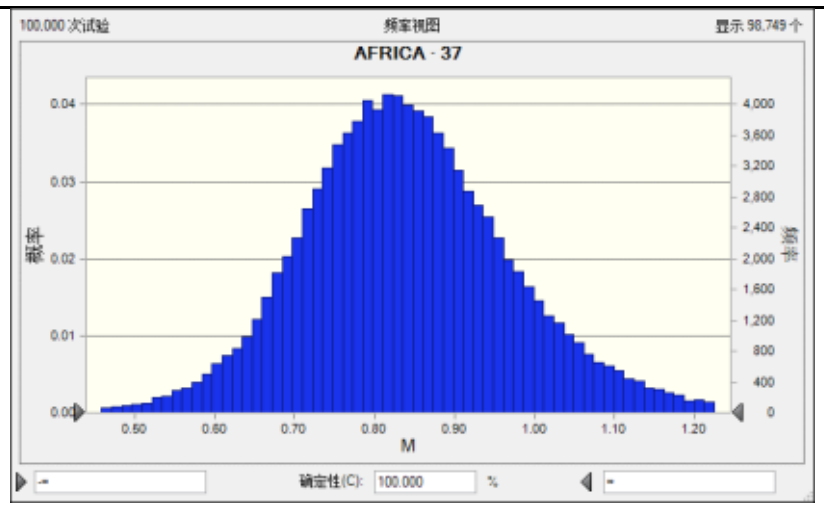 AFRICA - 37	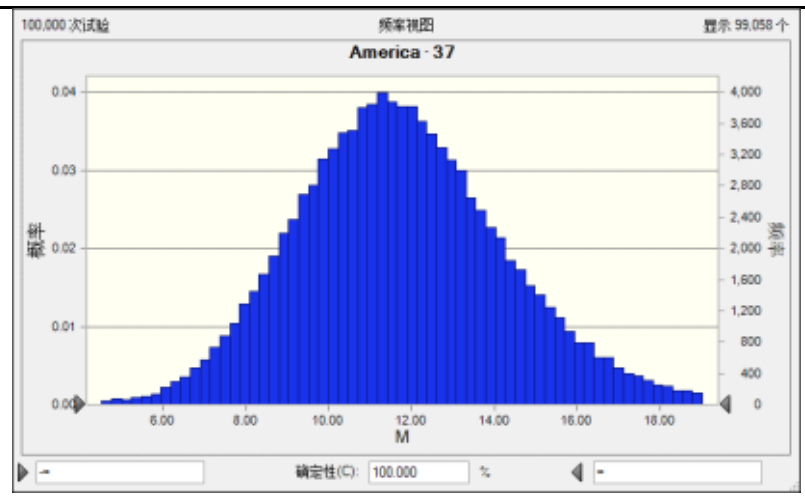 America - 37	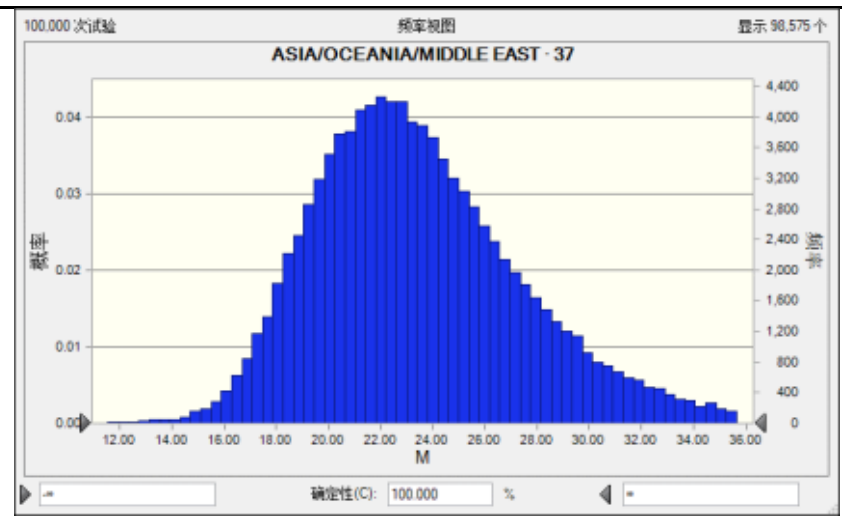 ASIA/OCEANIA/MIDDLE EAST - 37	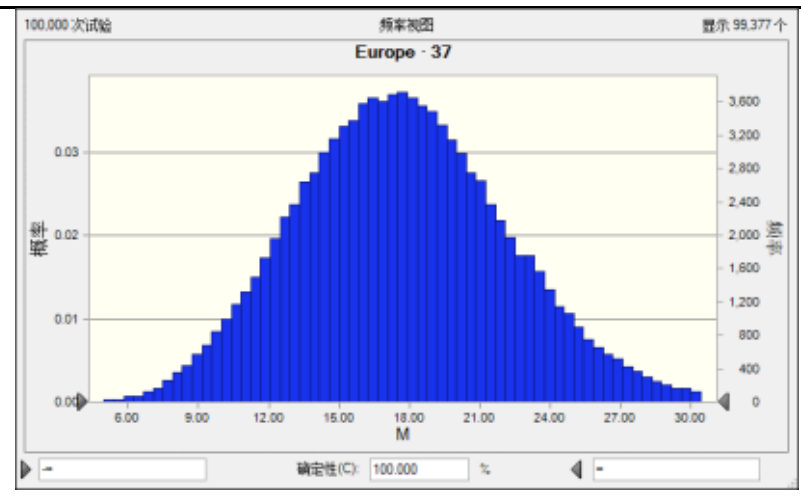 Europe - 37	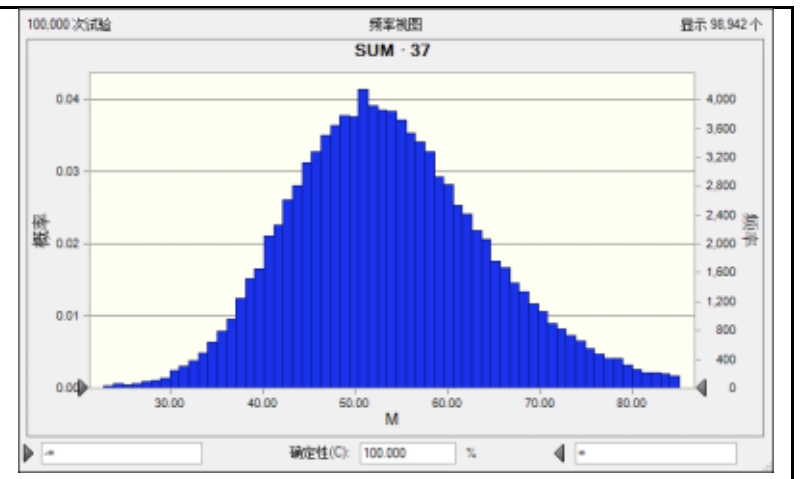 SUM - 37
2021	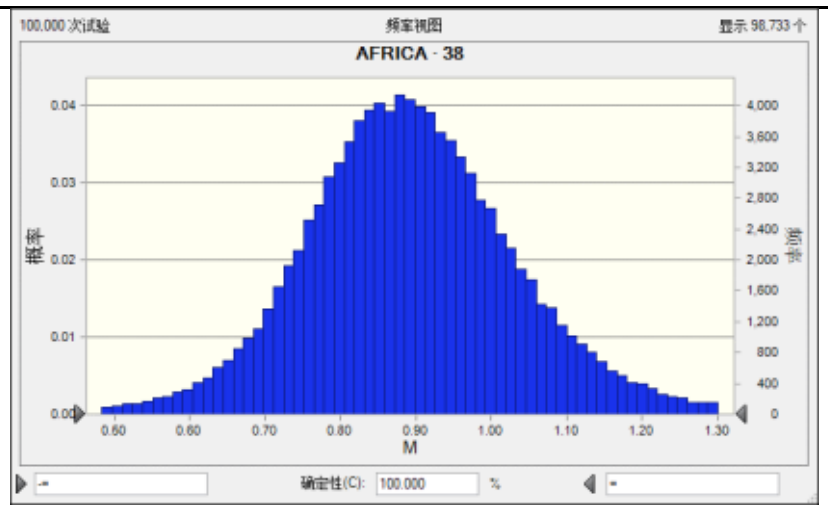 AFRICA - 38	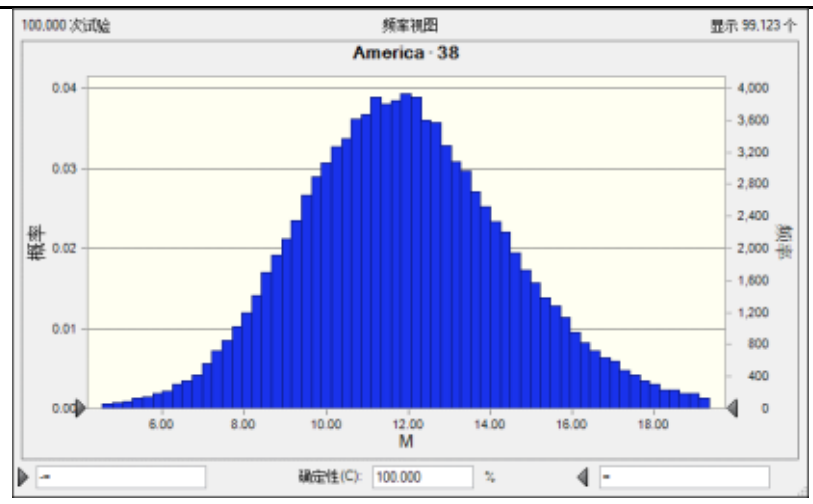 America - 38	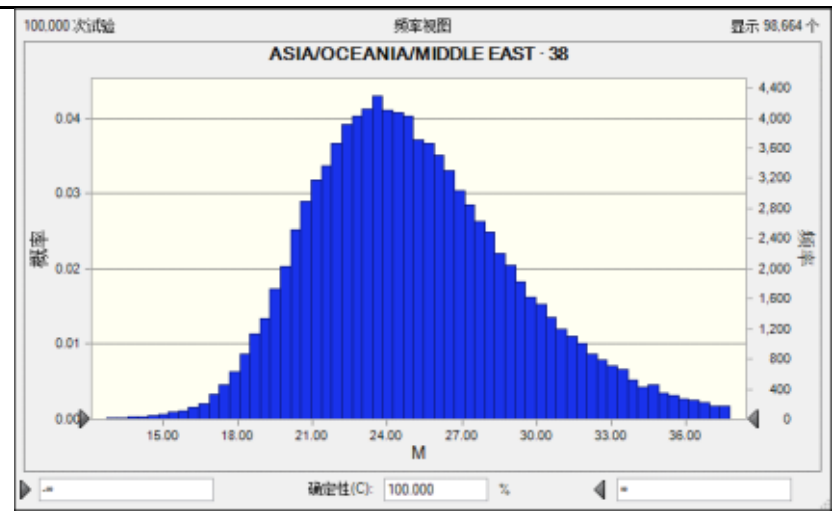 ASIA/OCEANIA/MIDDLE EAST - 38	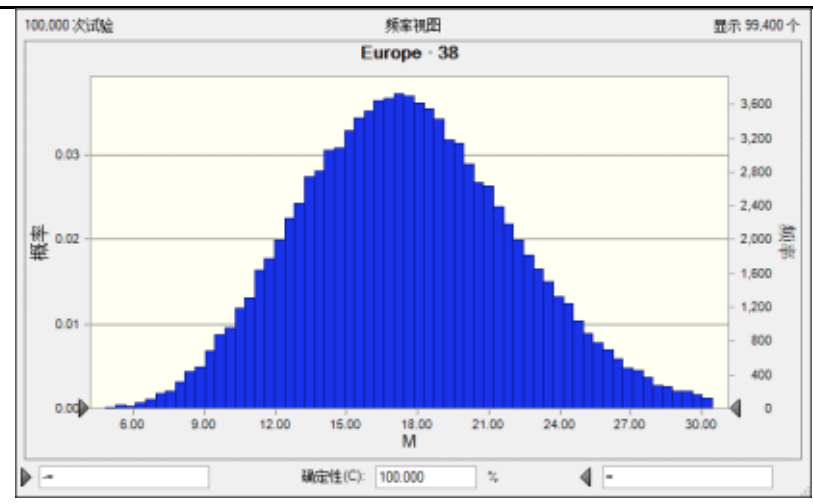 Europe - 38	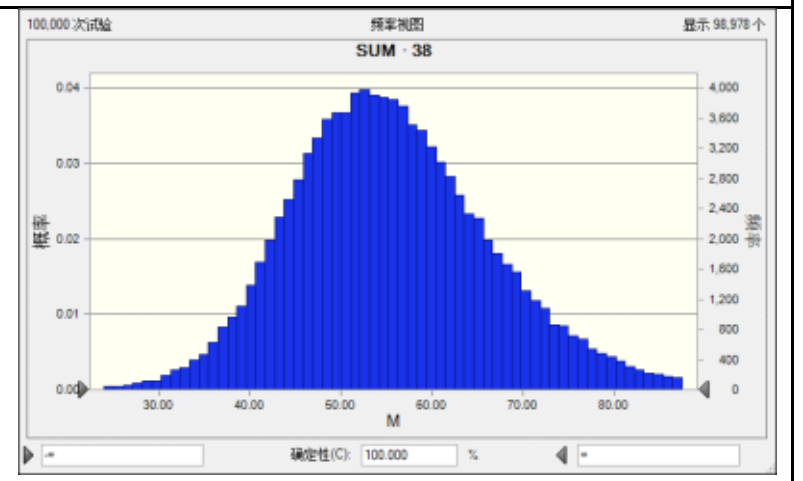 SUM - 38

Table S13: Uncertainty analysis of EoL PCs generation in main countries in 2000-2025, Related to Figures 1, 2, and 3.

Year	U.S.	Japan	China	Germany	UK	France	India
2000	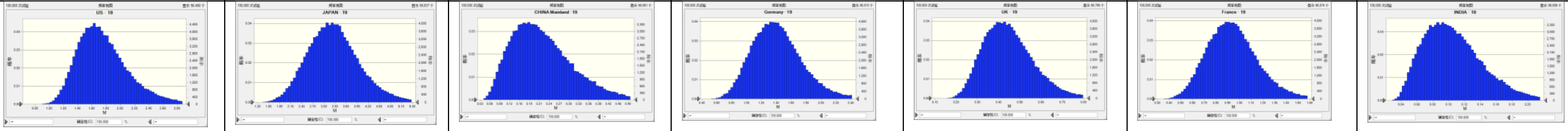						
2001	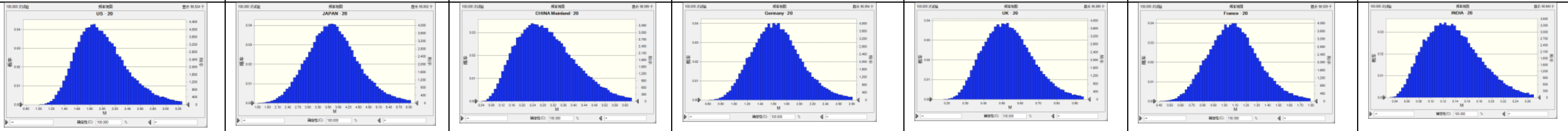						
2002	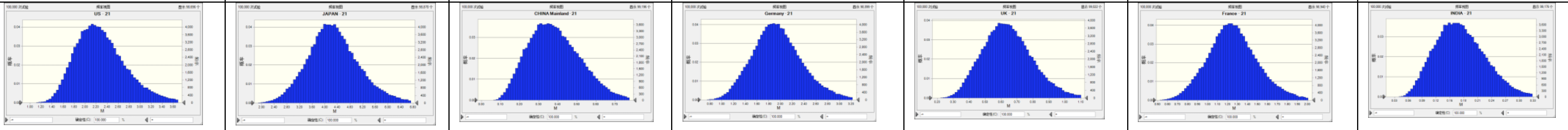						
2003	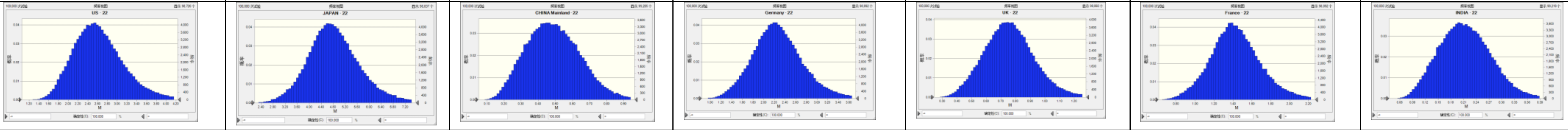						
2004	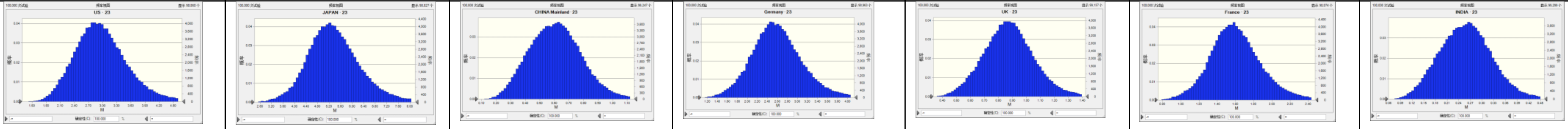						
2005	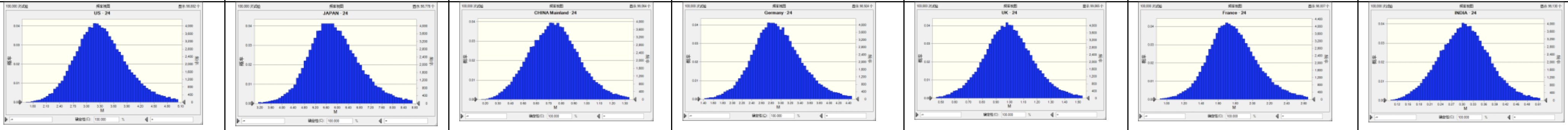						
2006	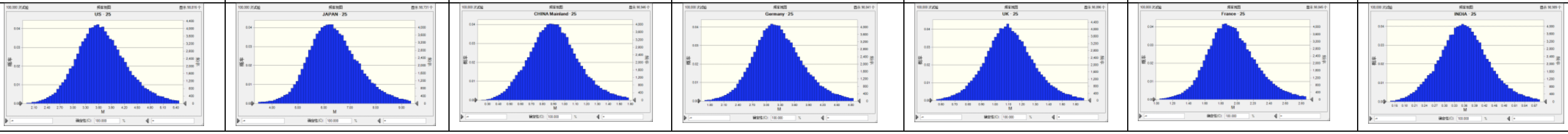						

2007	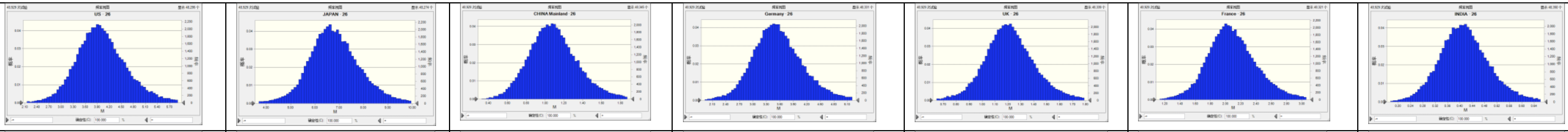
2008	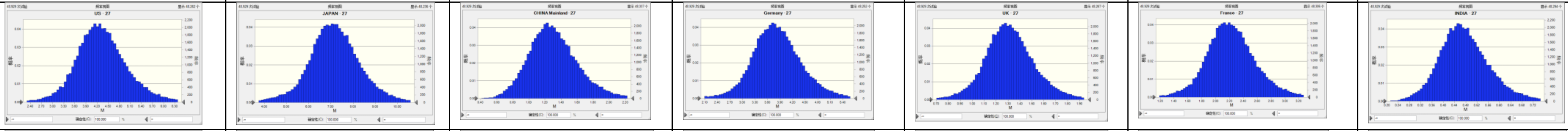
2009	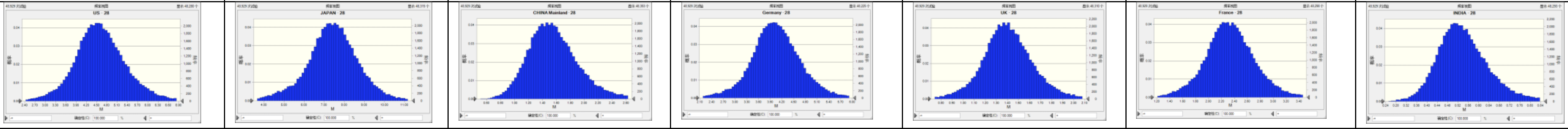
2010	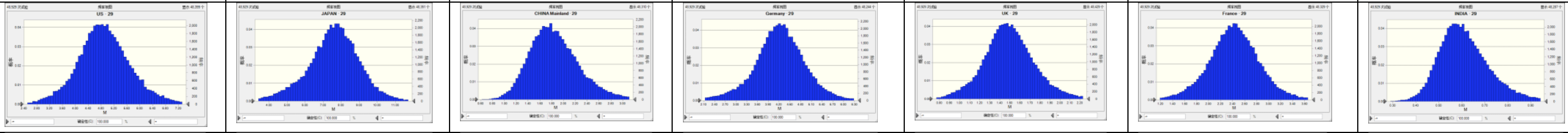
2011	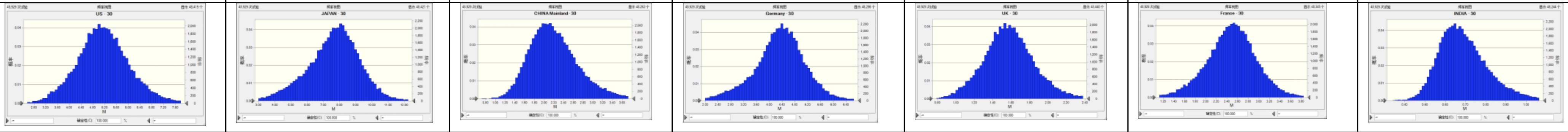
2012	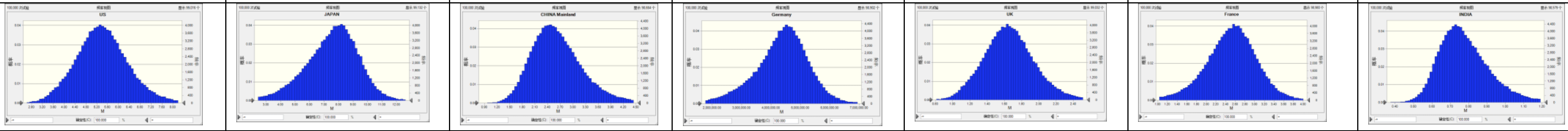
2013	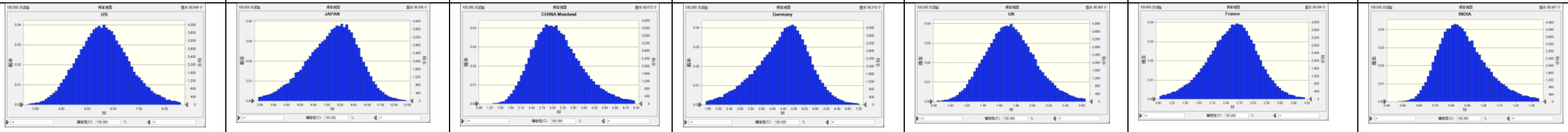
2014	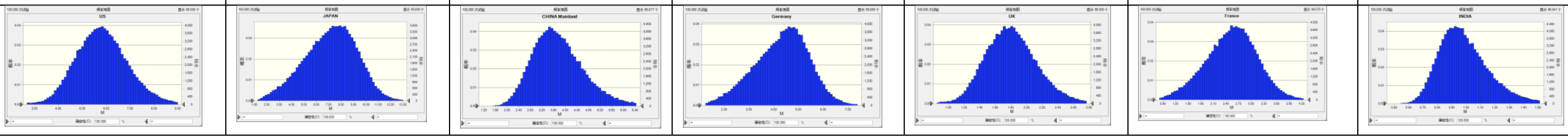

2015	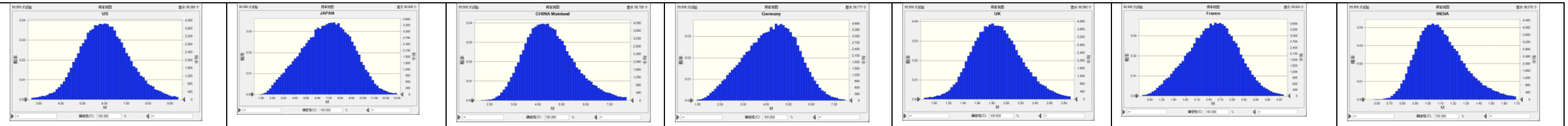
2016	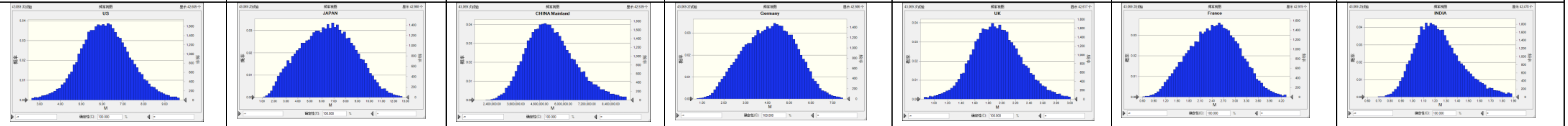
2017	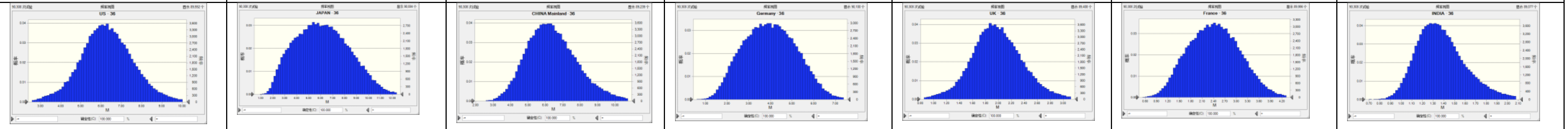
2018	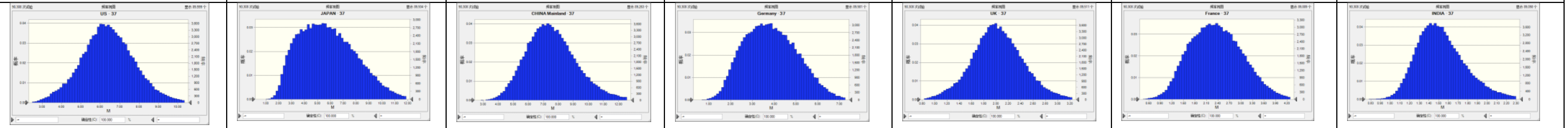
2019	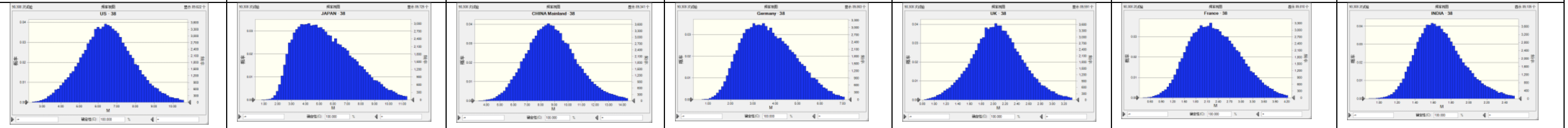
2020	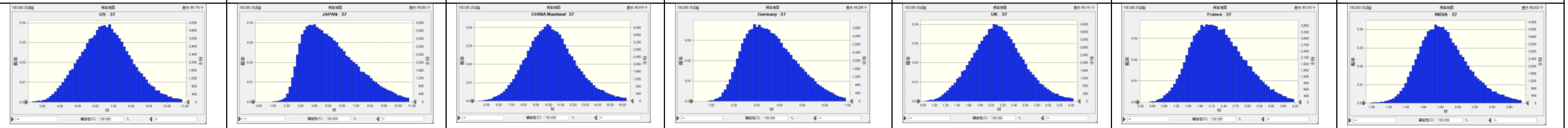
2021	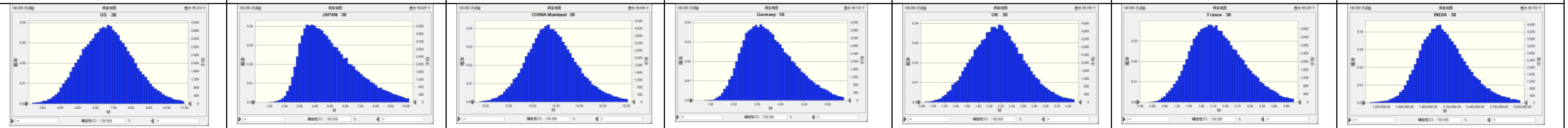
2022	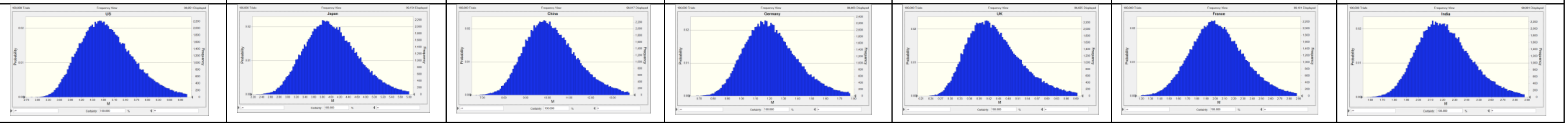

2023	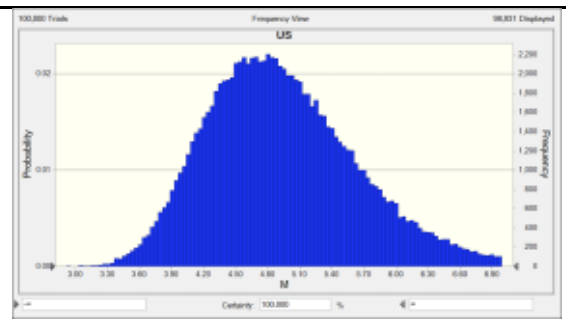	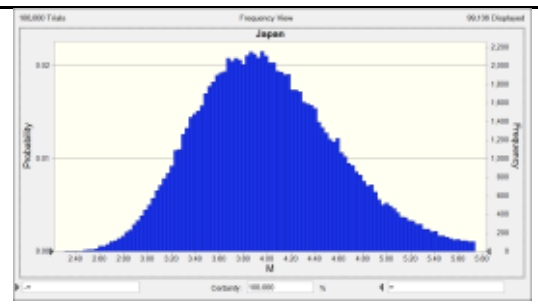	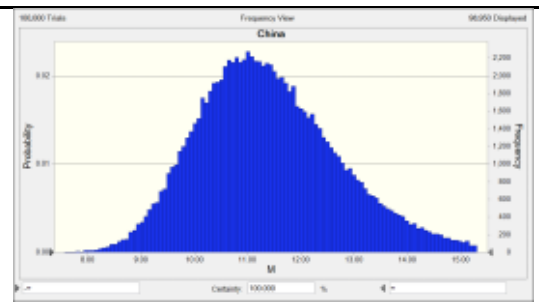	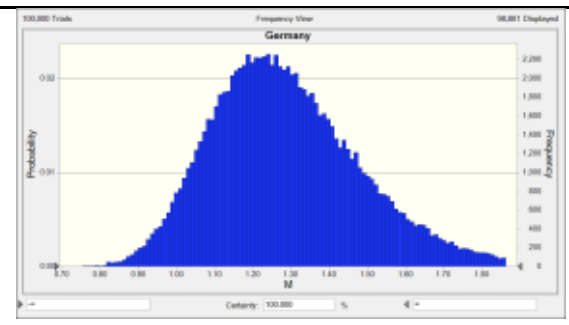	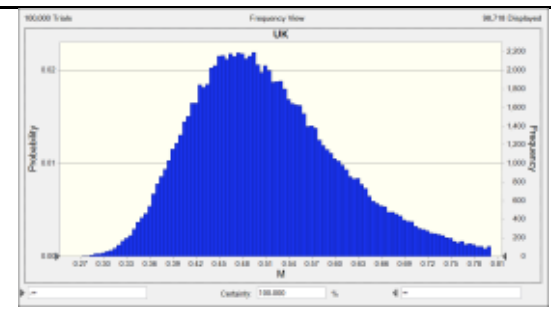	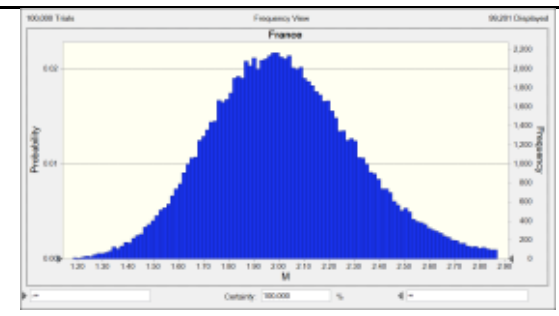	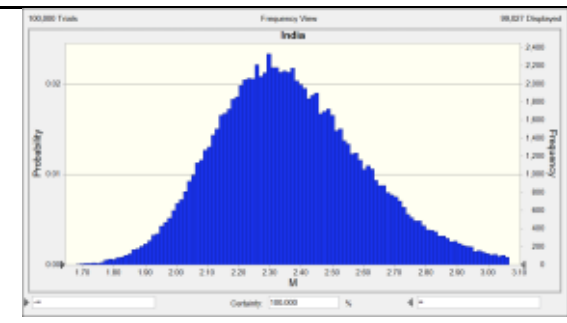
2024	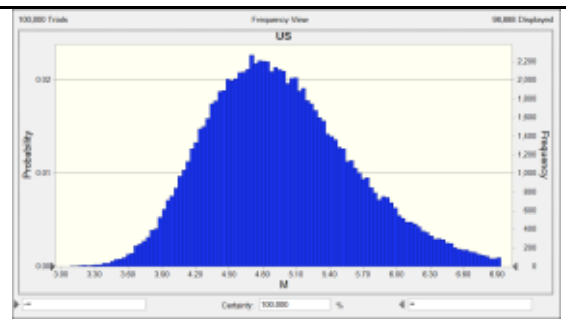	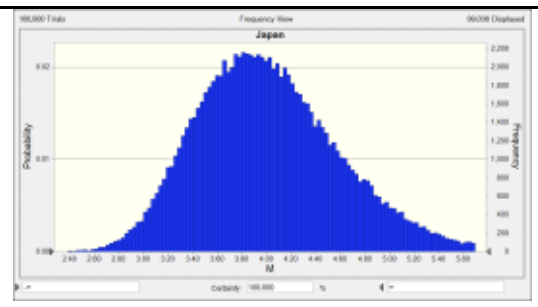	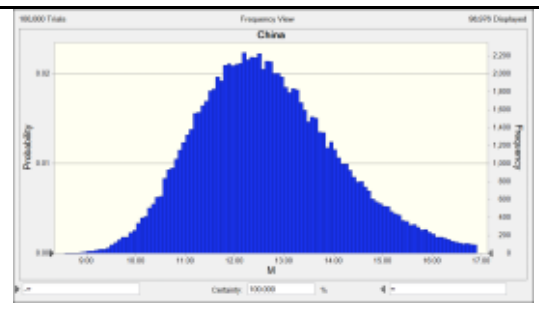	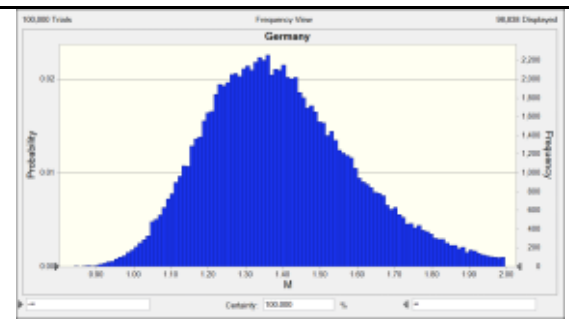	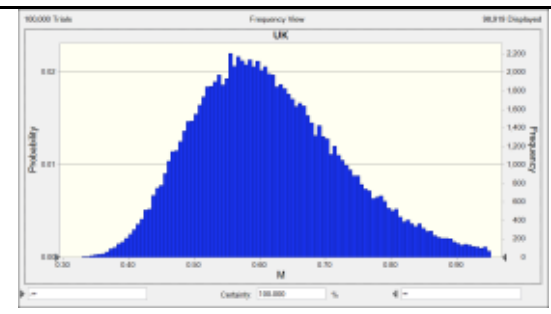	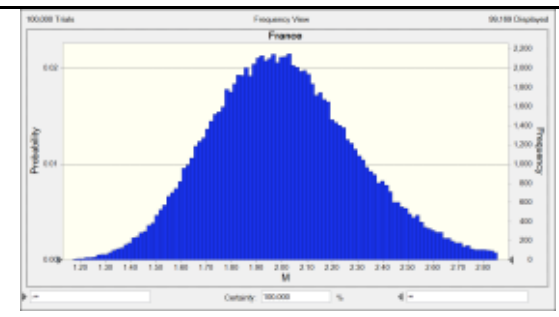	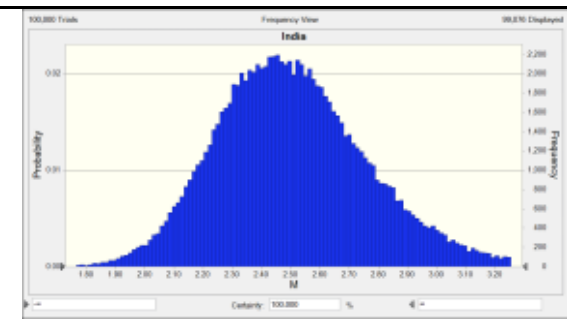
2025	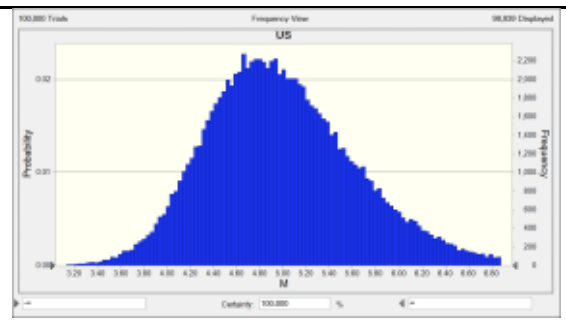	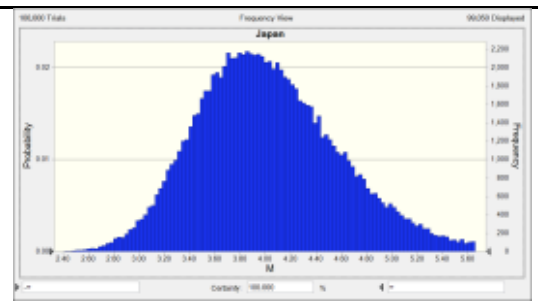	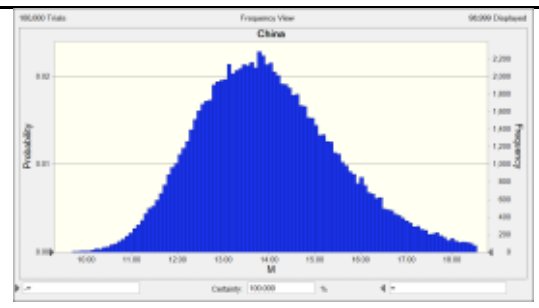	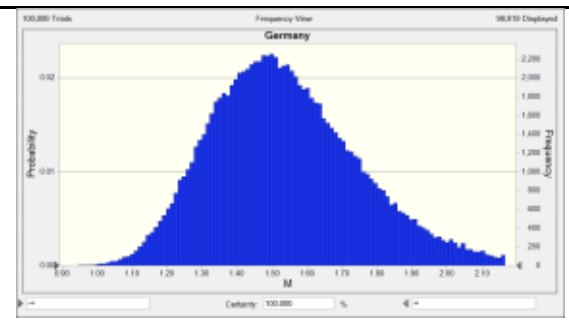	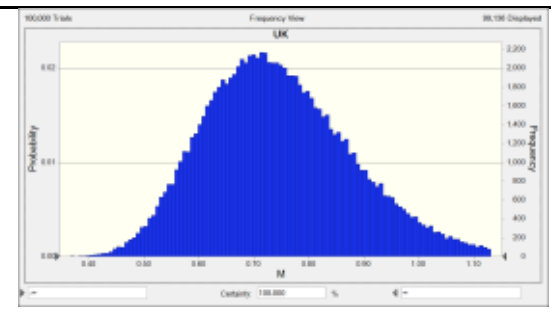	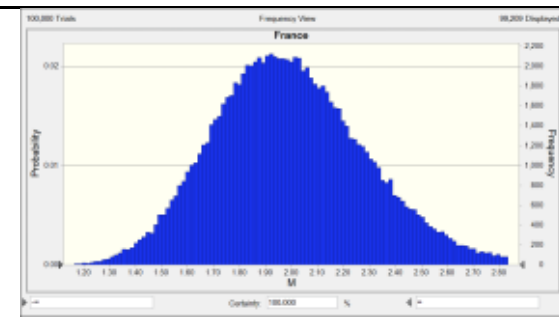	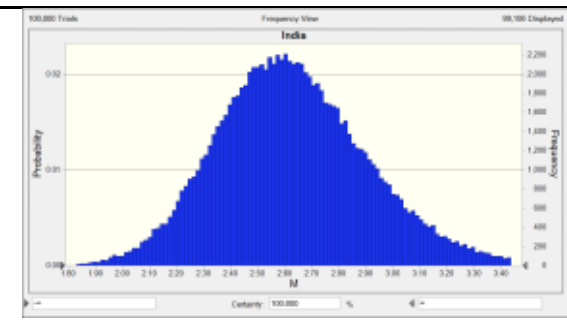

Table S14: Uncertainty analysis of EoL CVs generation in main continents in 2000-2021, Related to Figures 1, 2, and 3.

Year	Africa	America	Asia/Oceania/Middle East	Europe	Sum
2000	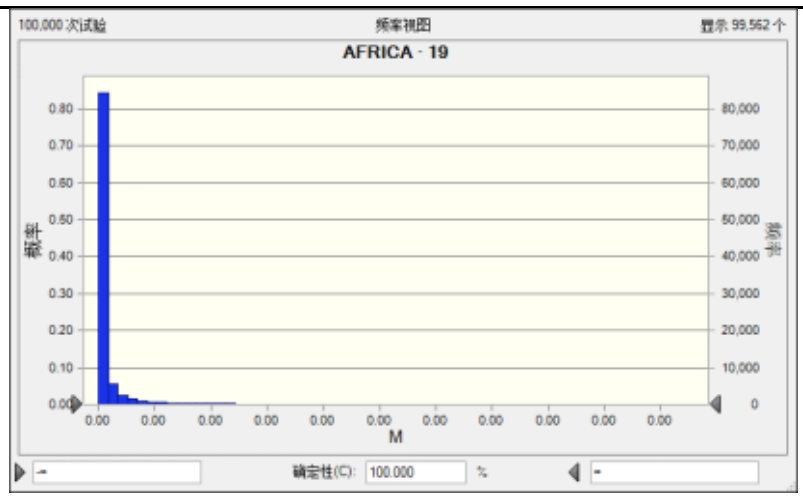	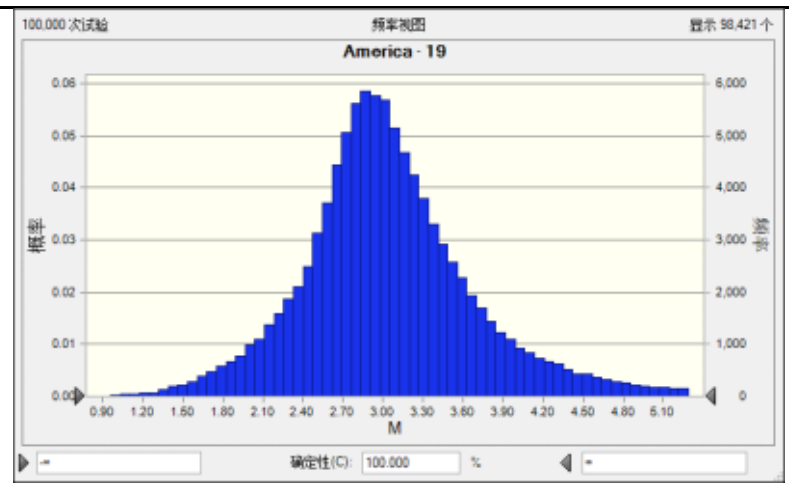	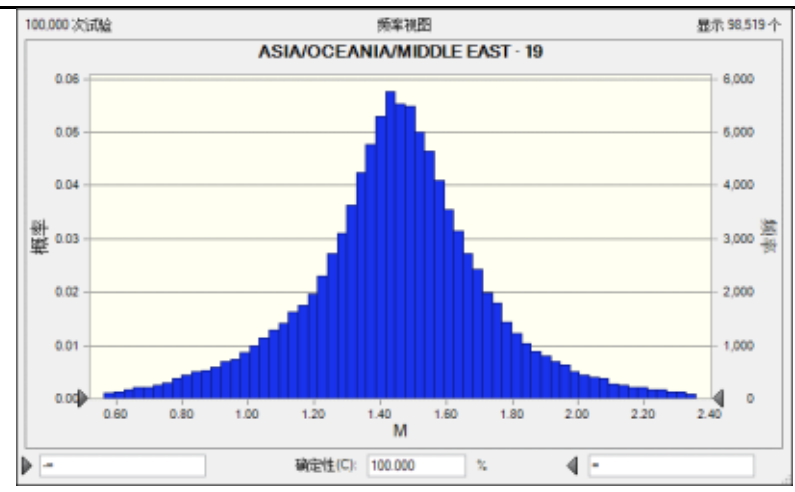	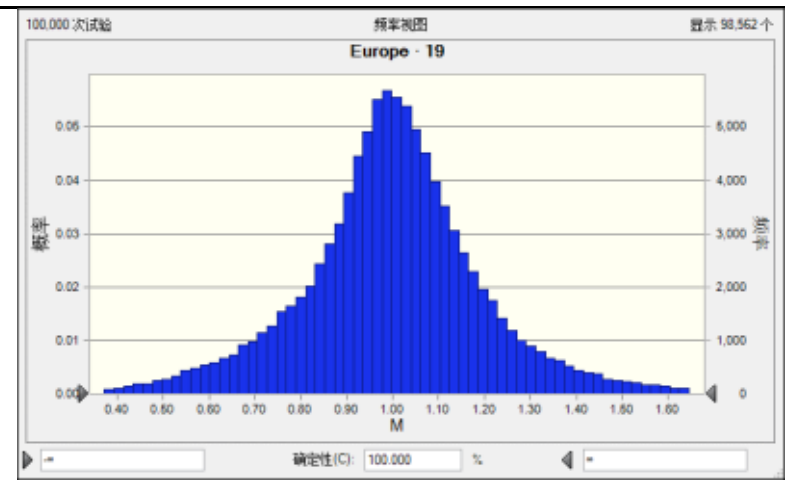	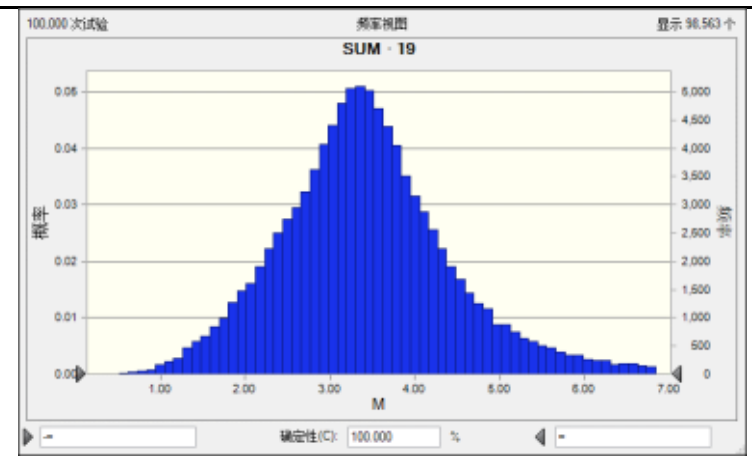
2001	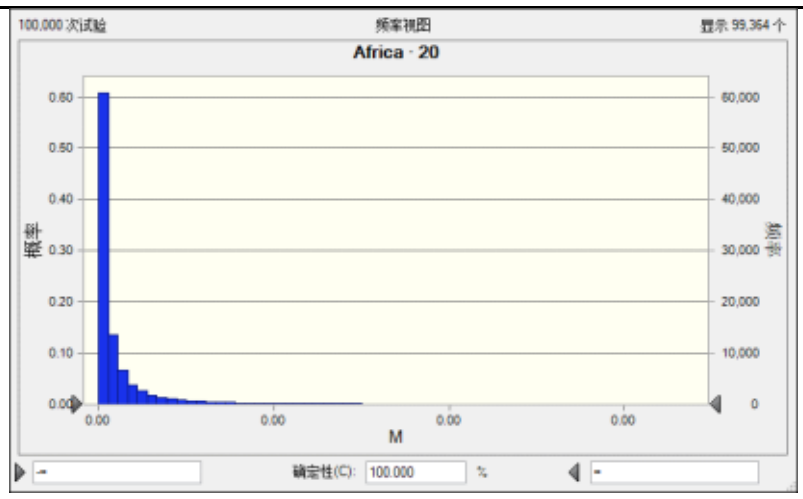	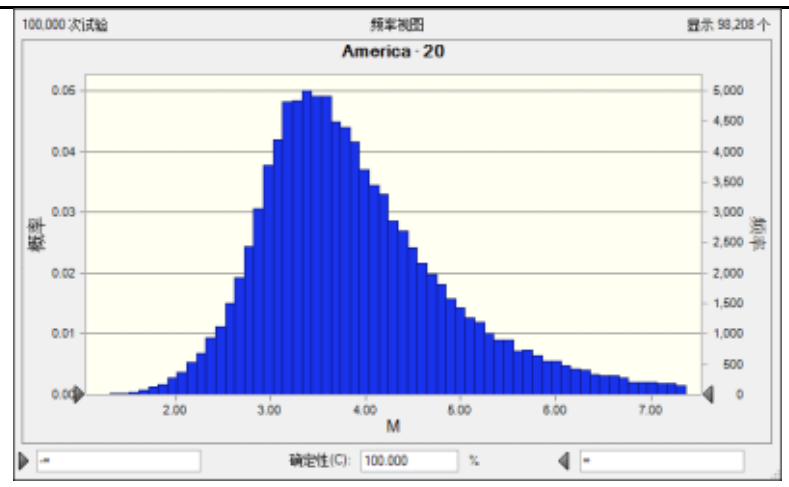	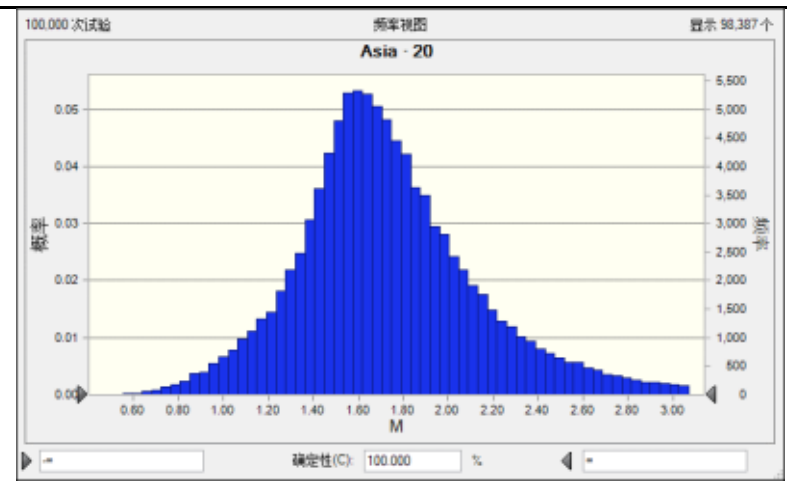	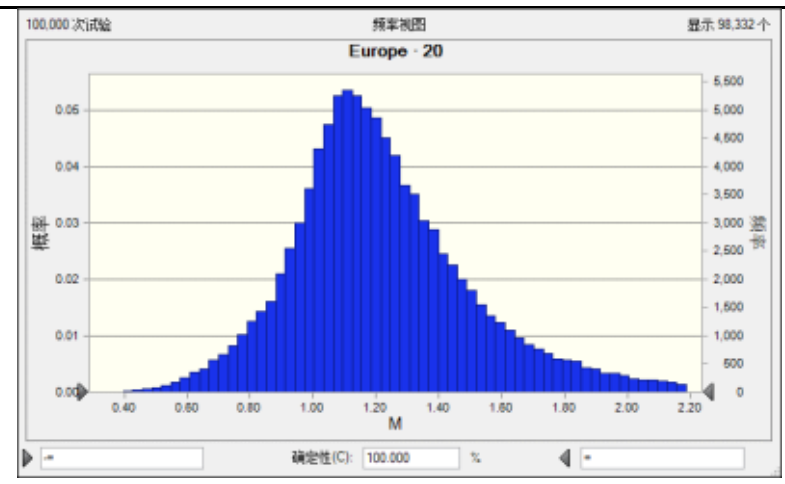	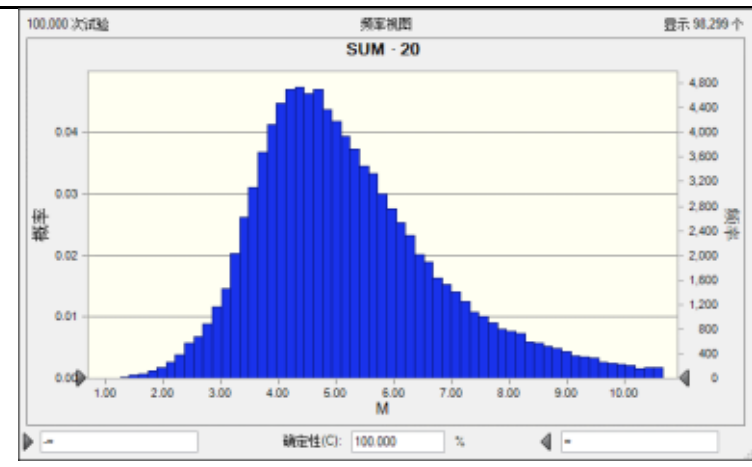

2002

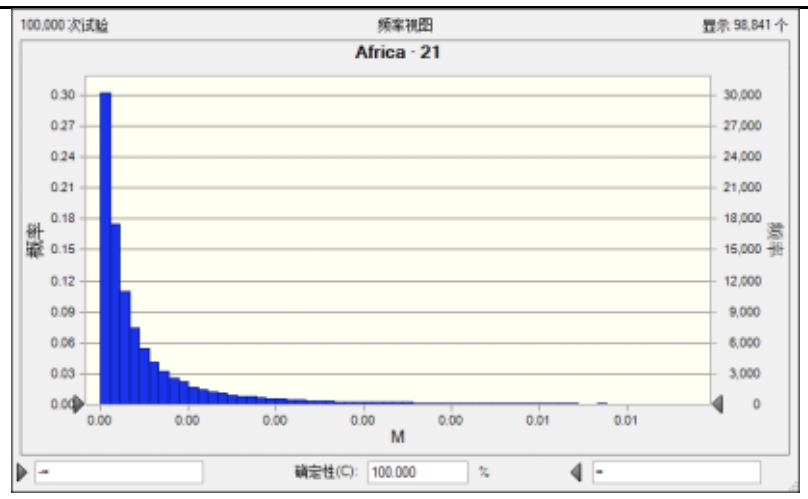

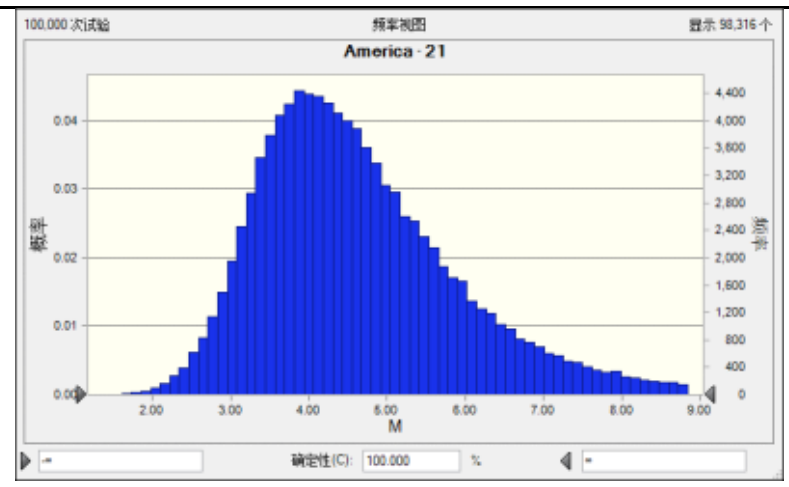

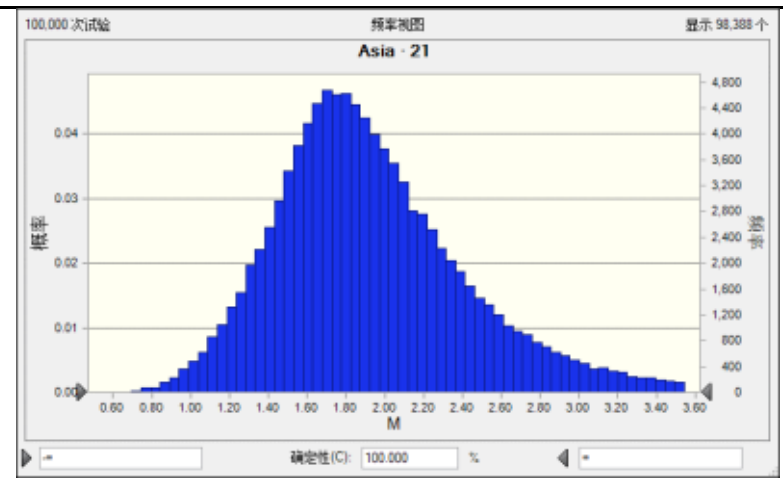

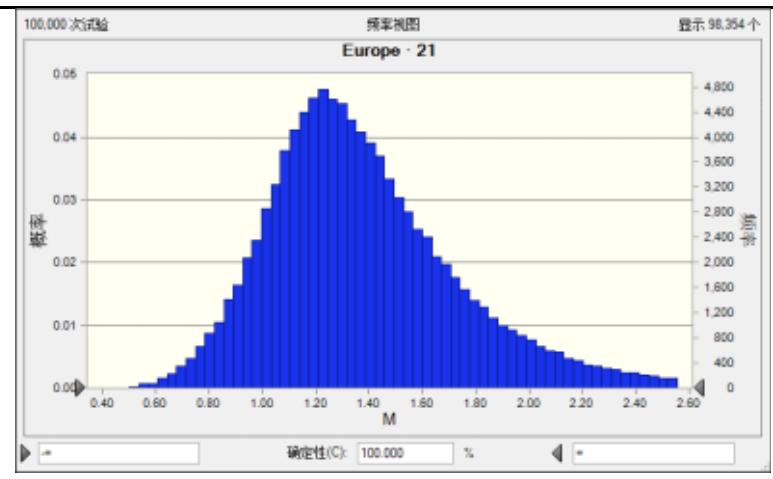

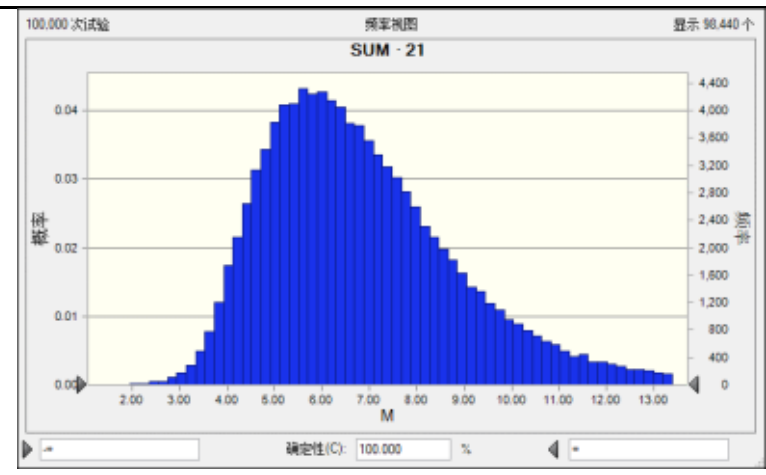

2003

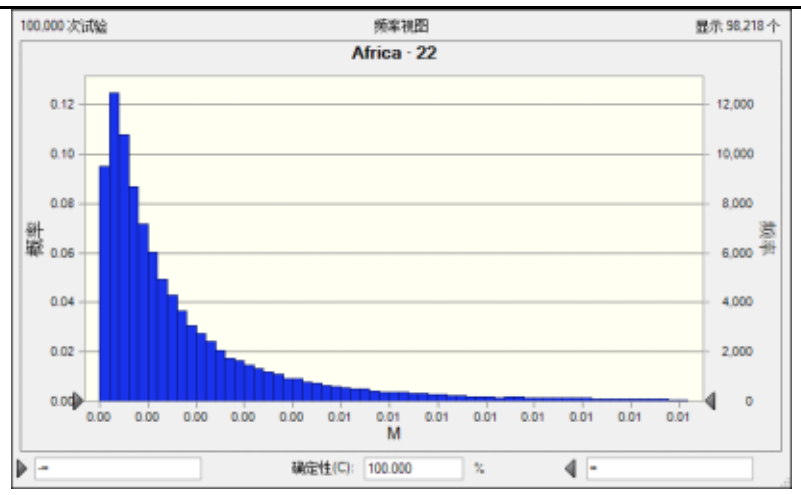

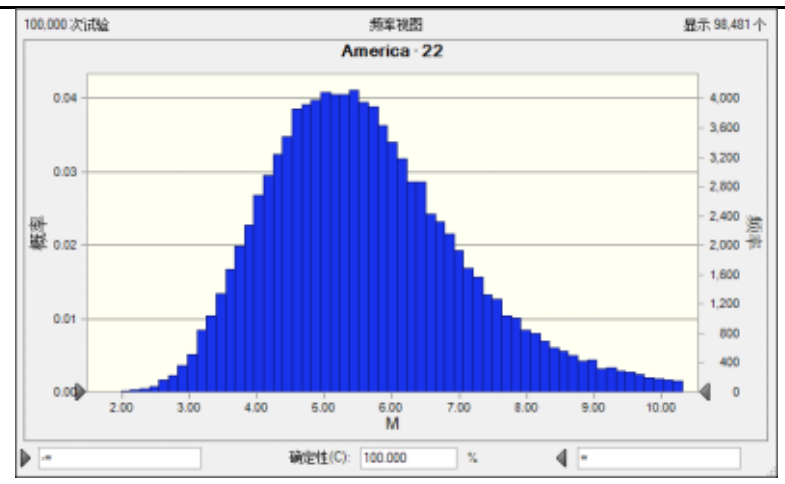

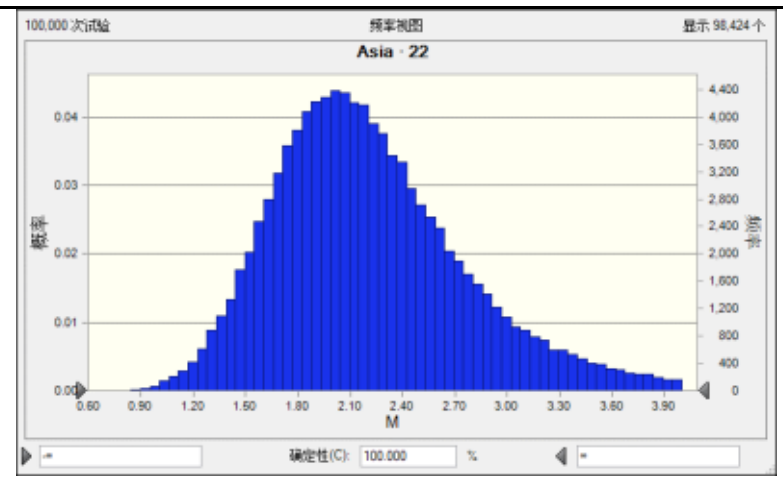

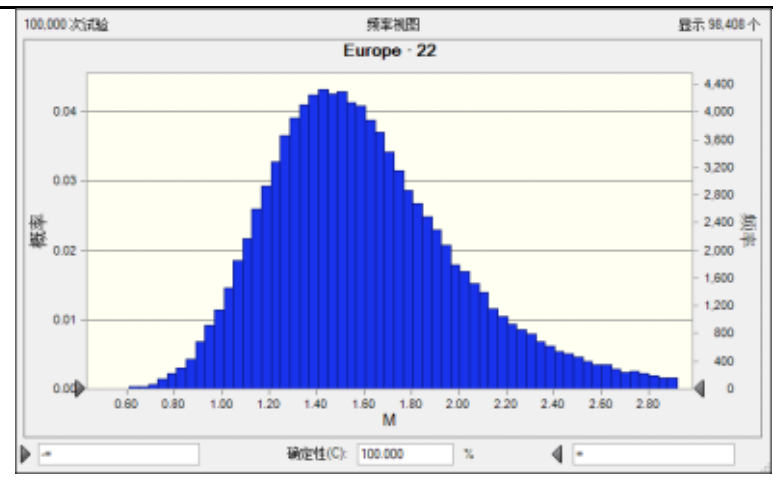

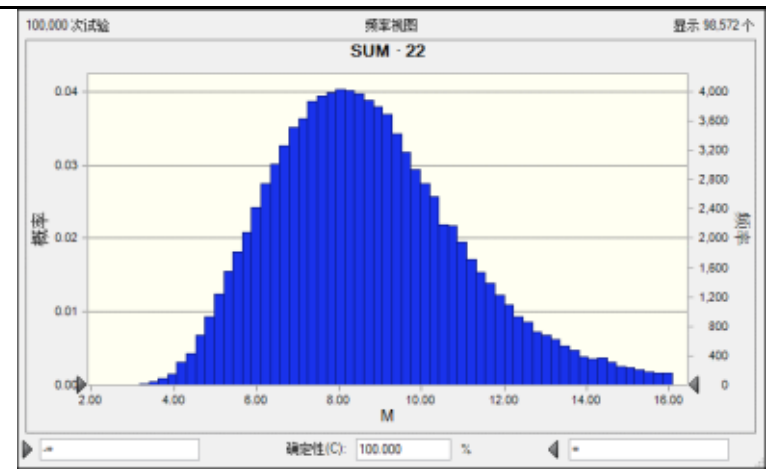

2004

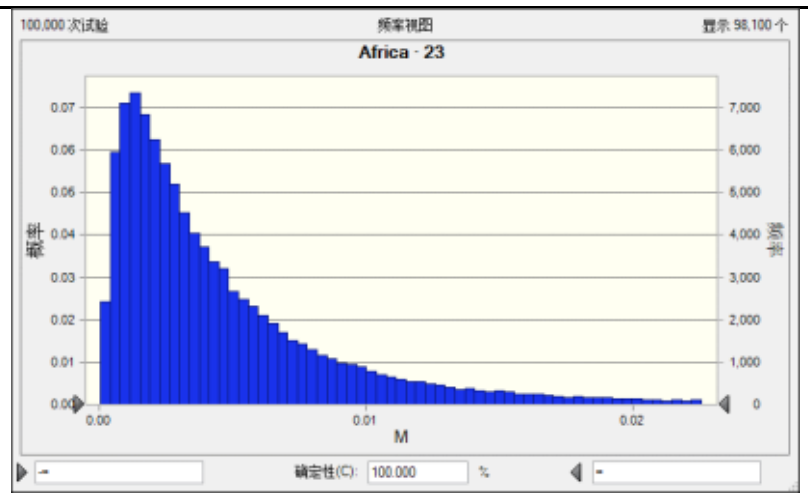

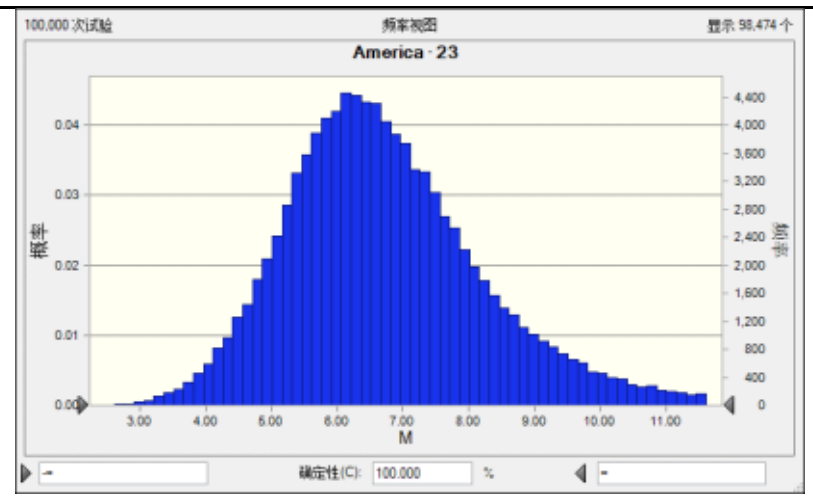

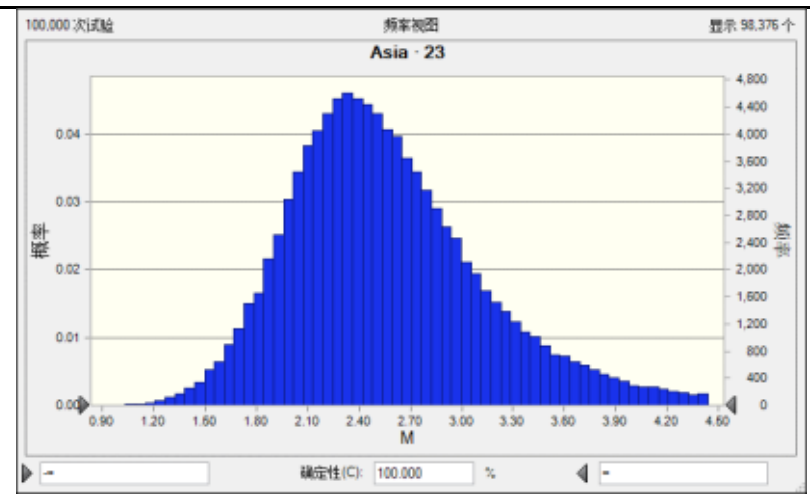

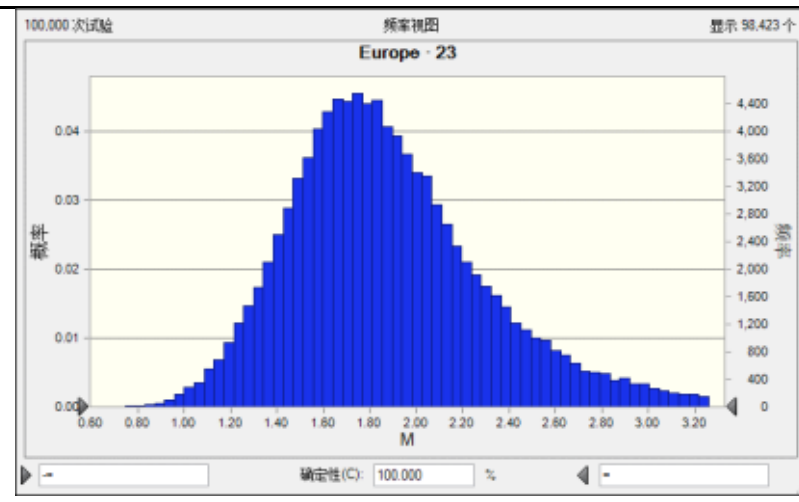

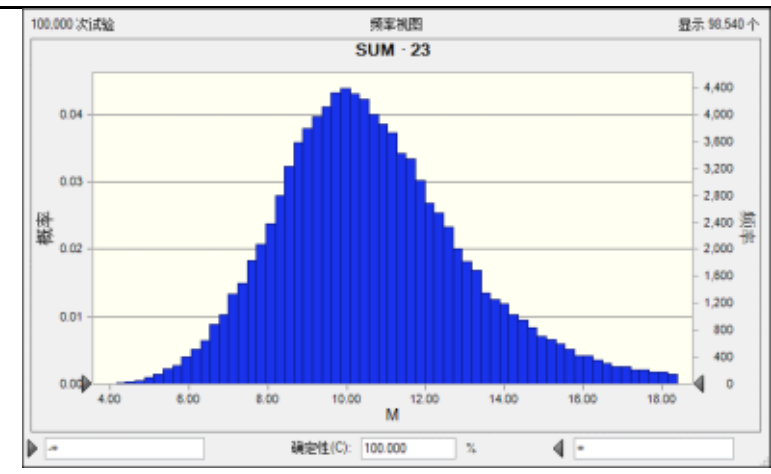

2005

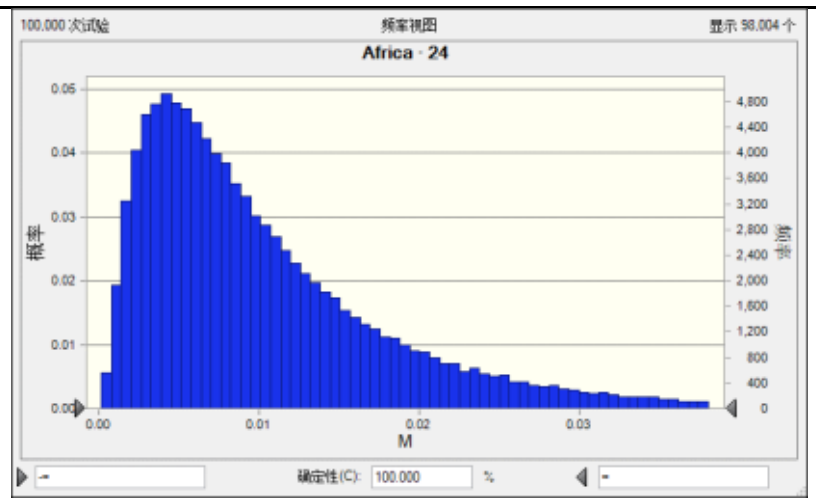

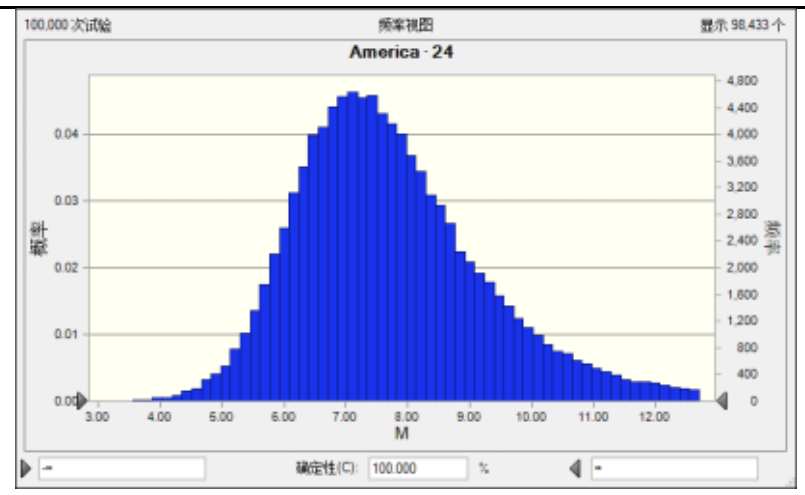

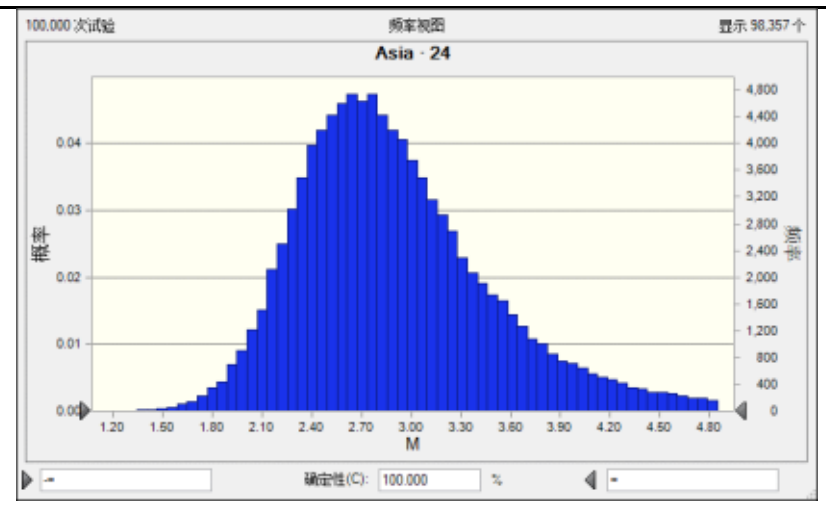

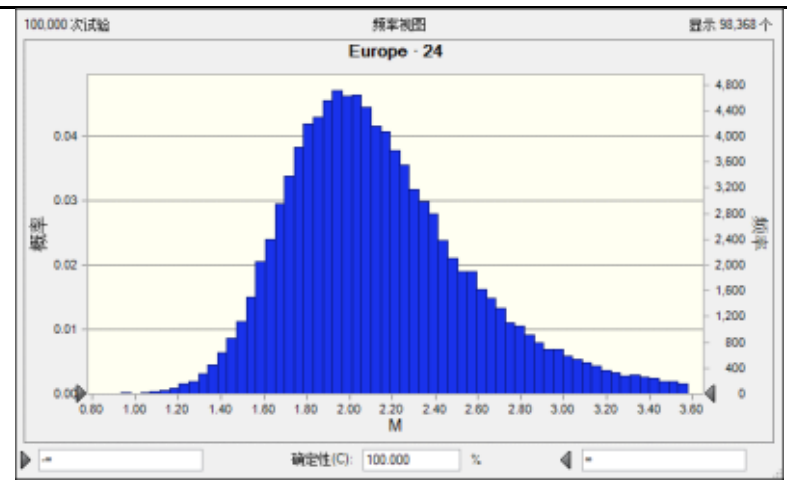

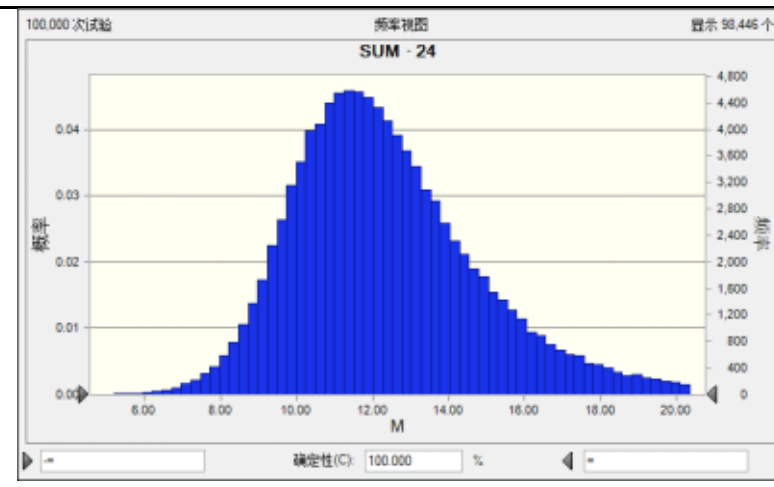

2006

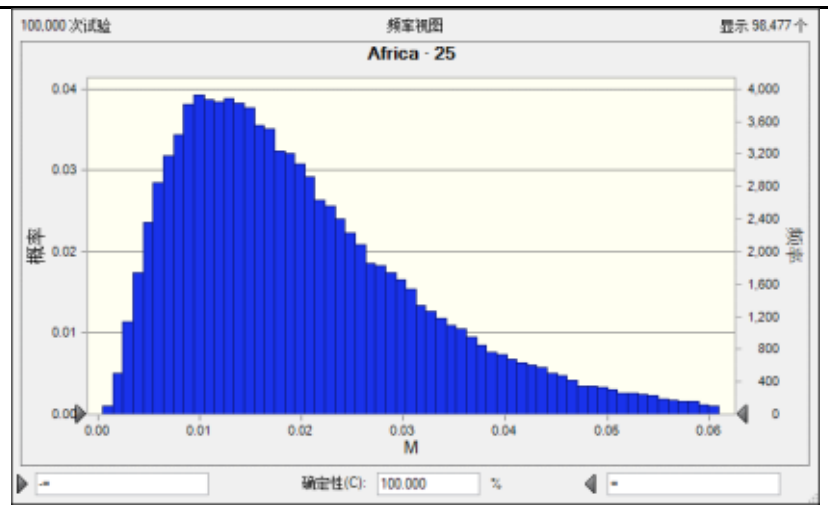

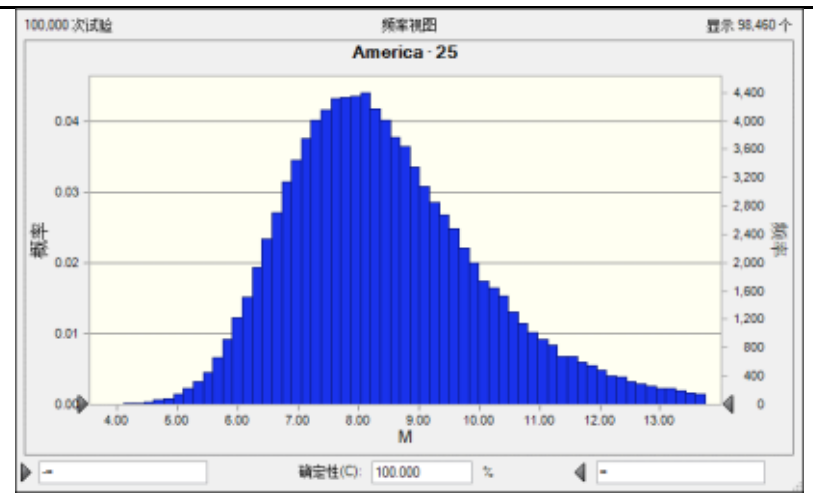

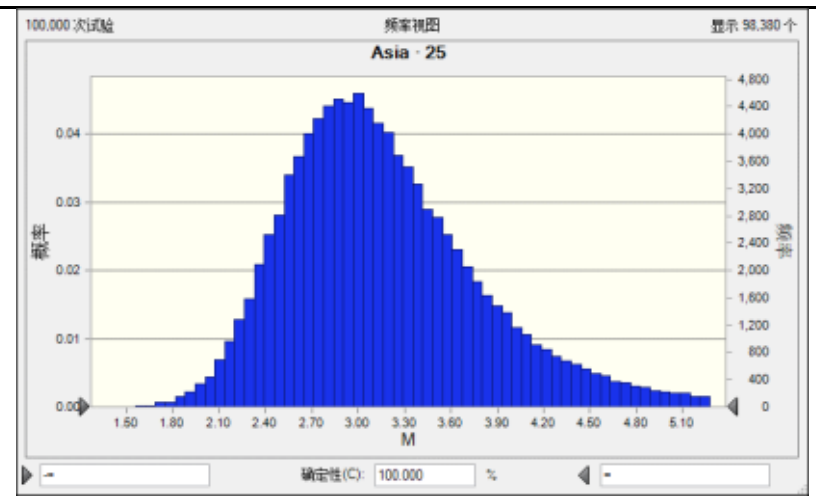

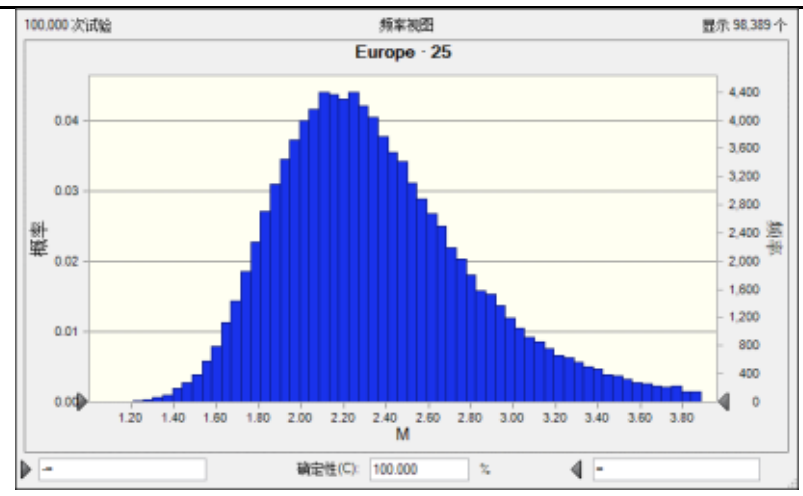

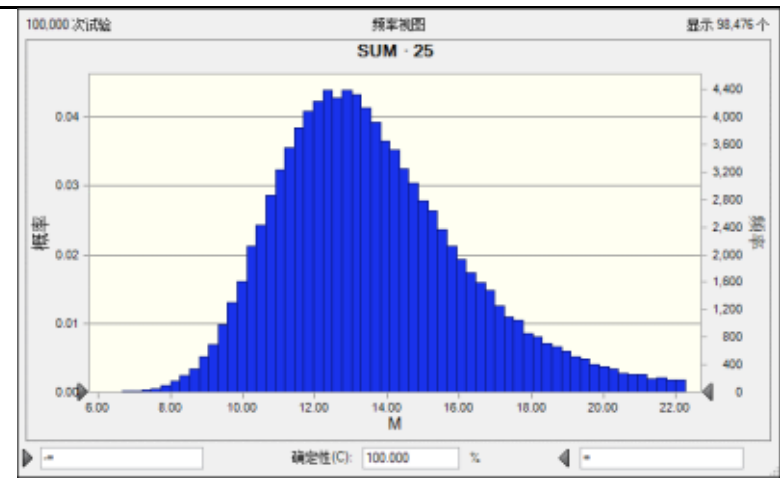

2007	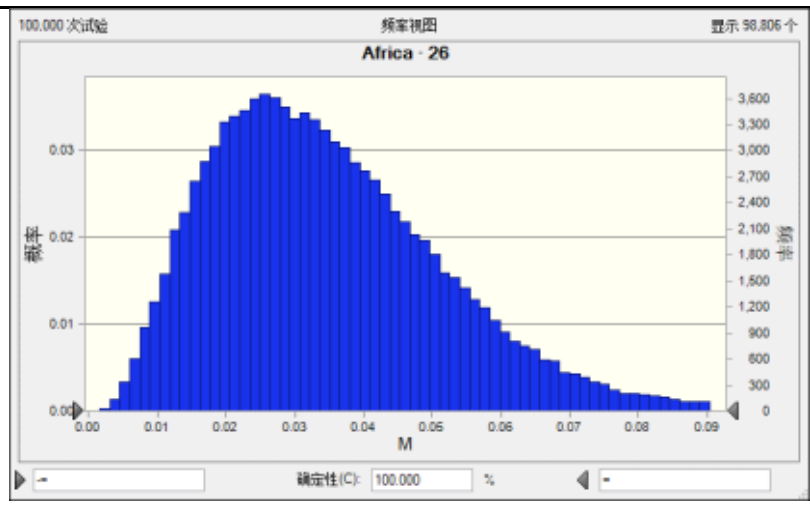	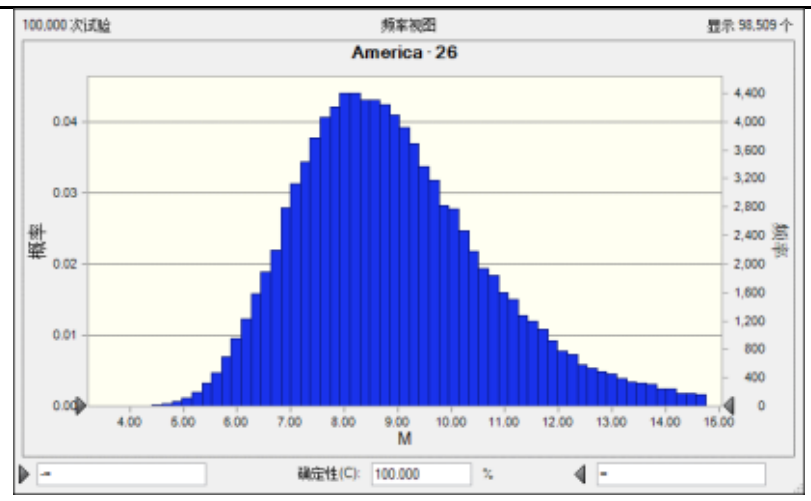	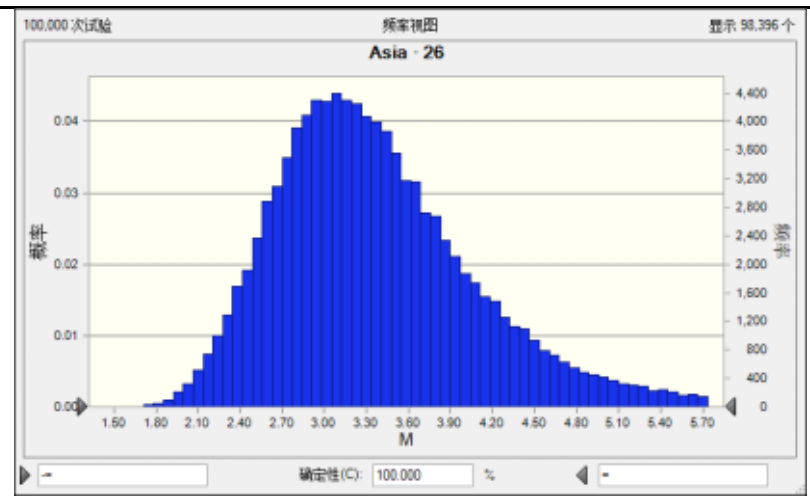	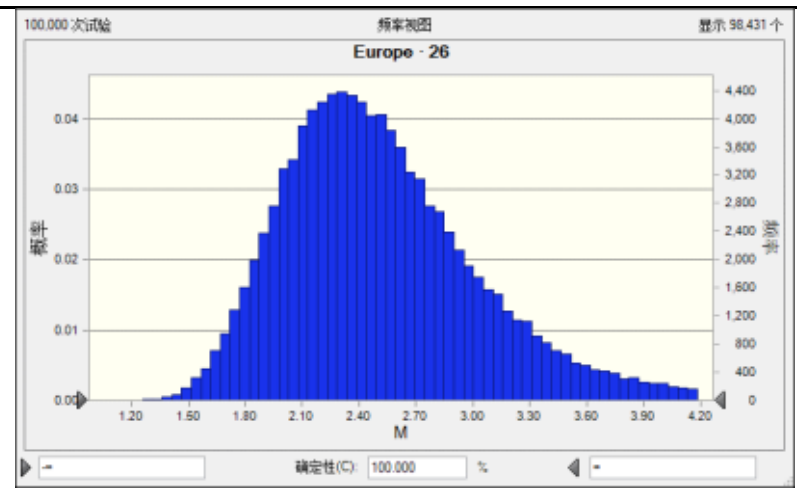	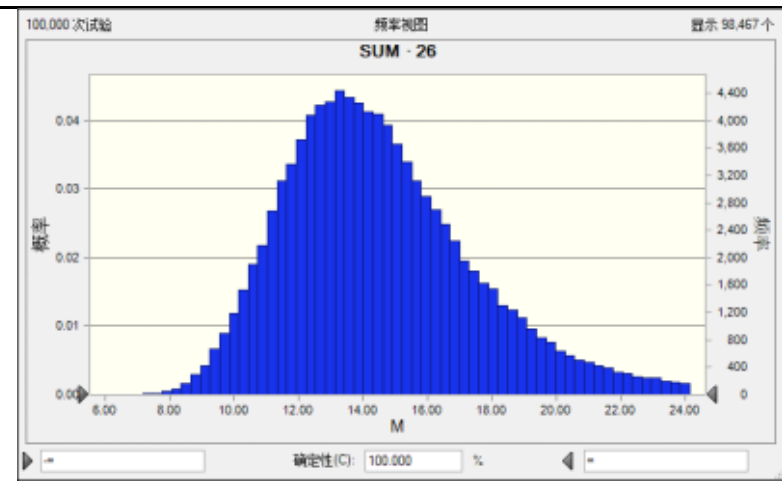
2008	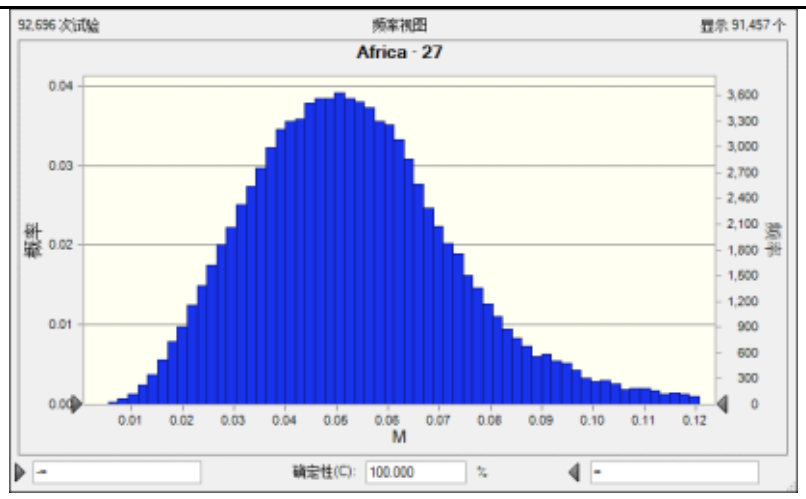	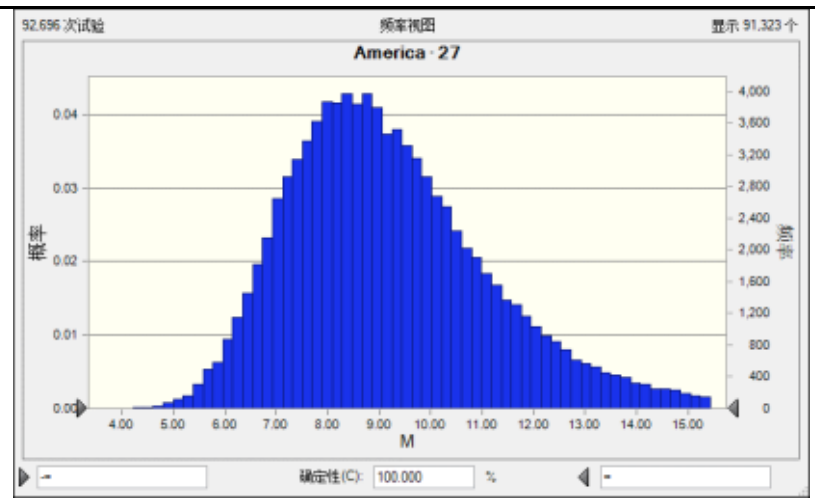	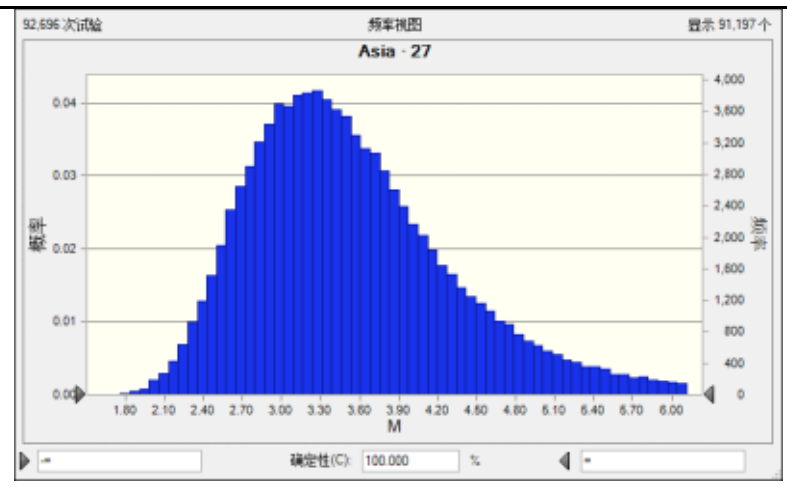	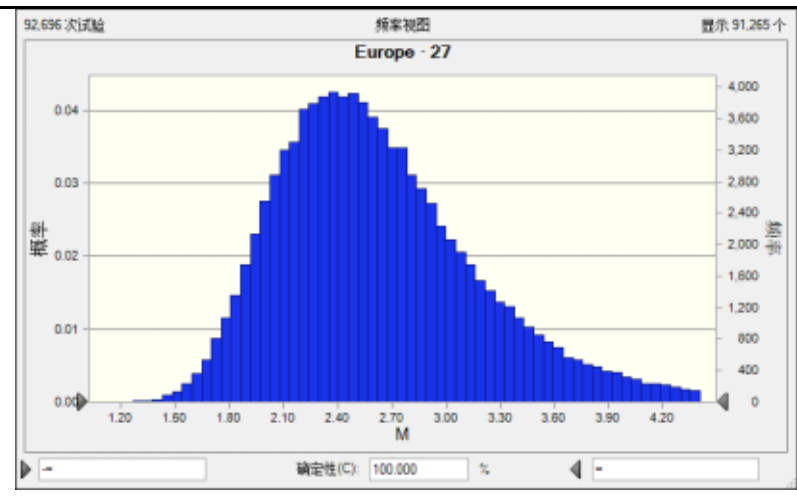	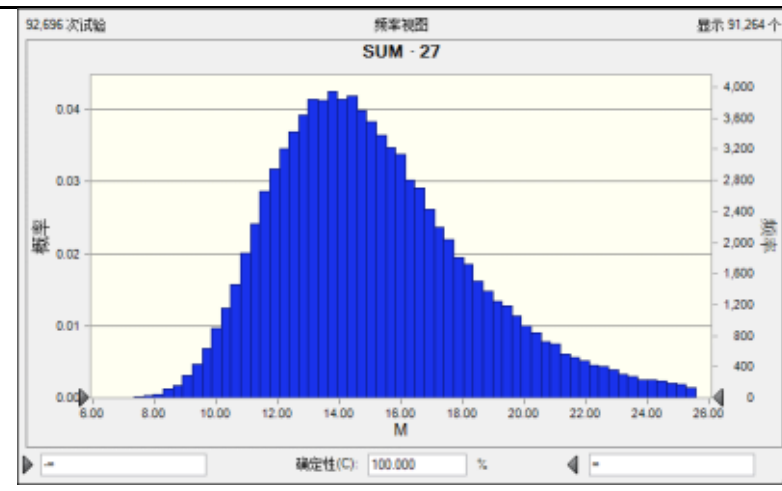
2009	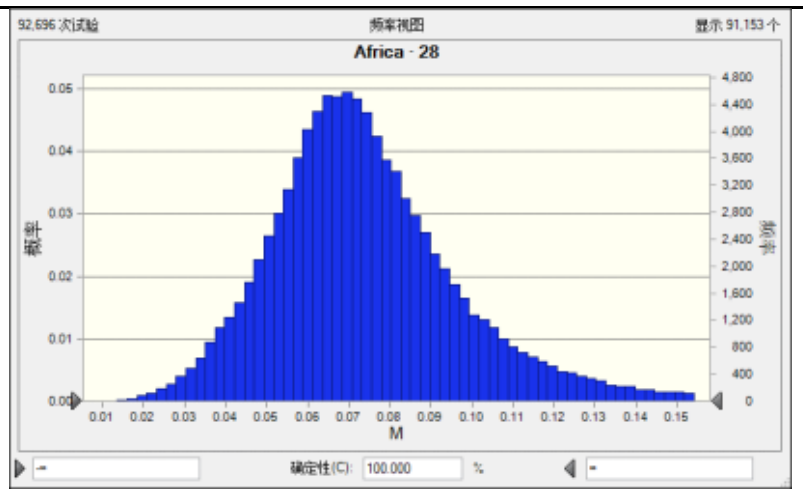	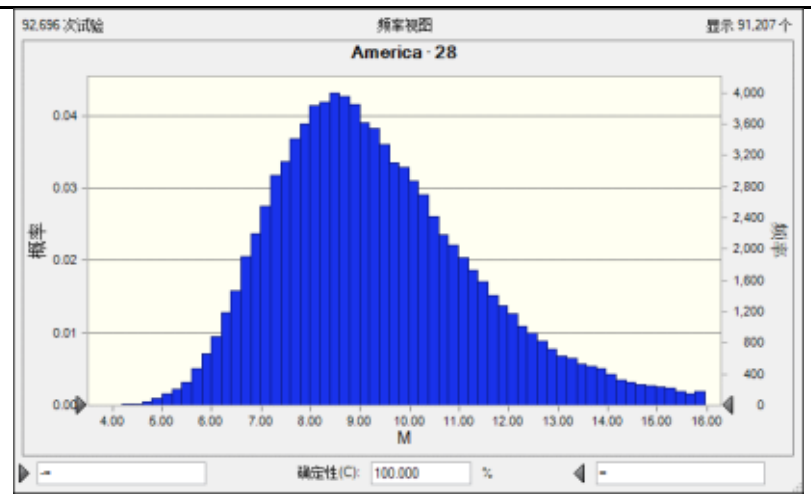	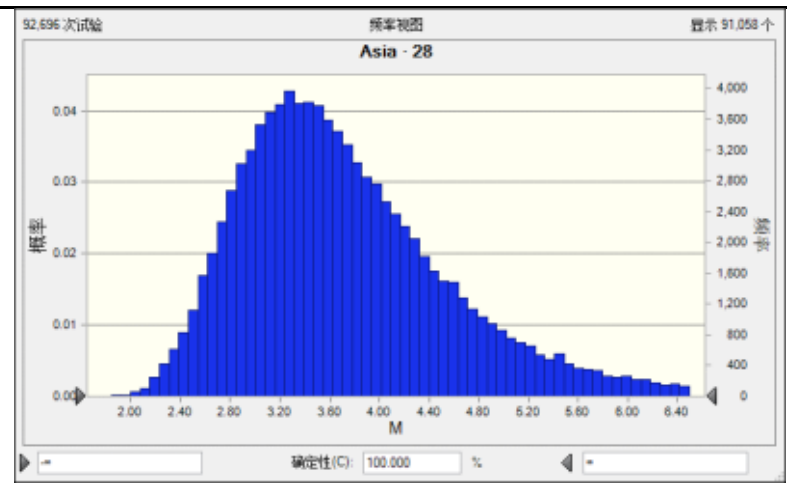	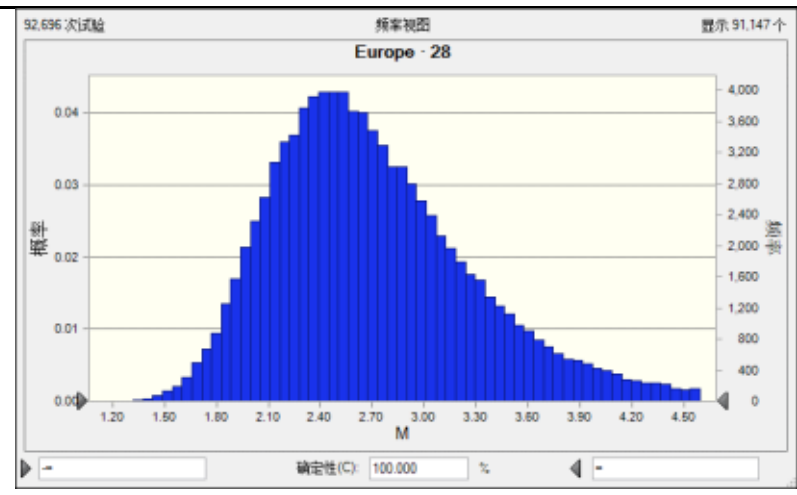	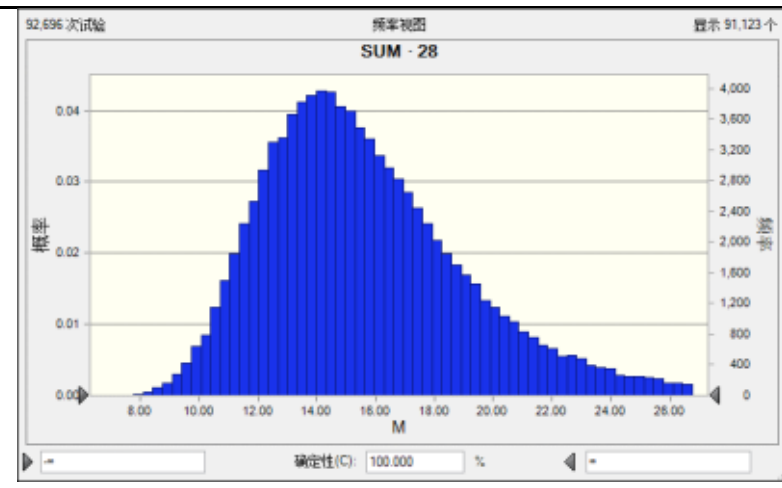
2010	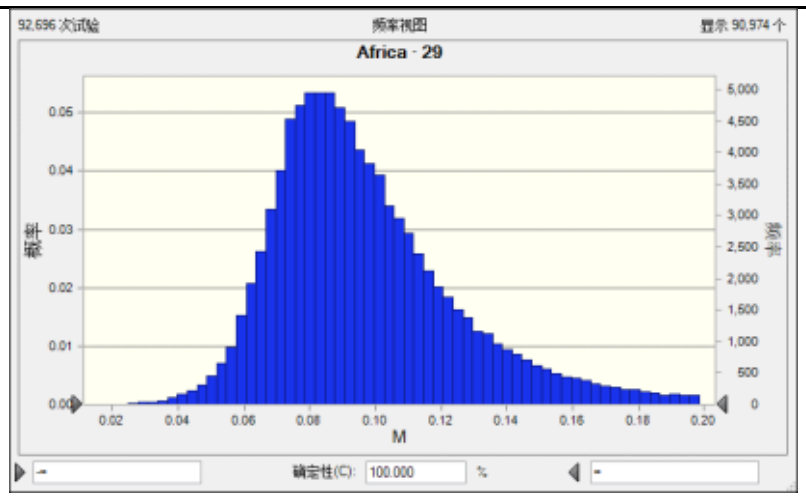	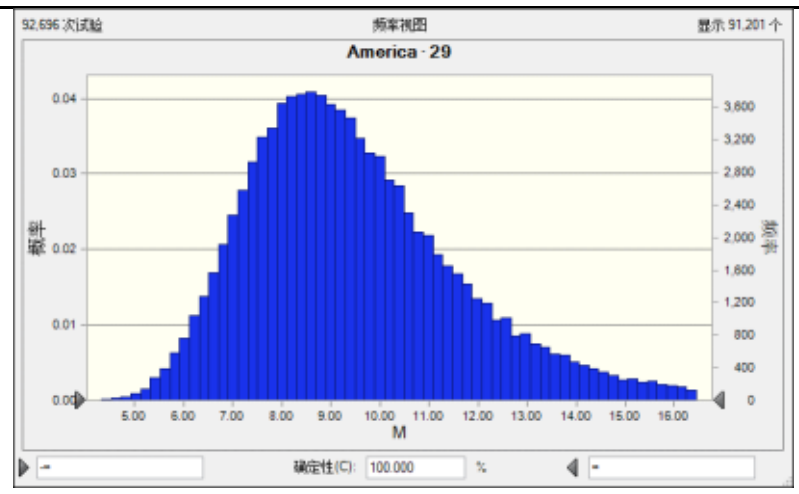	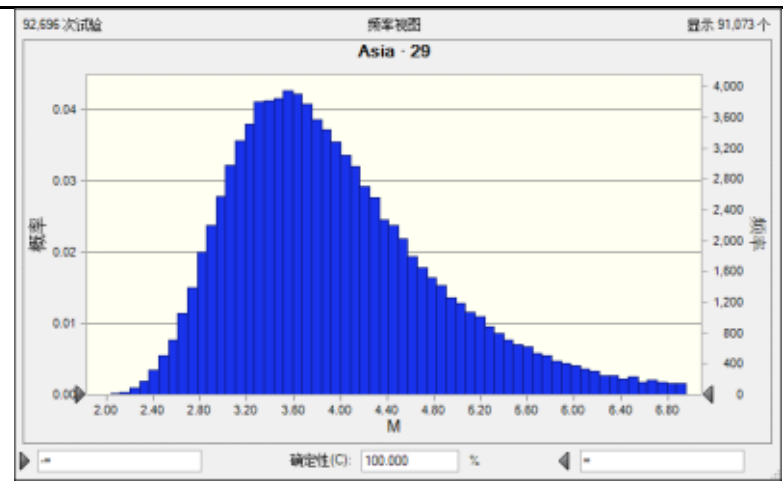	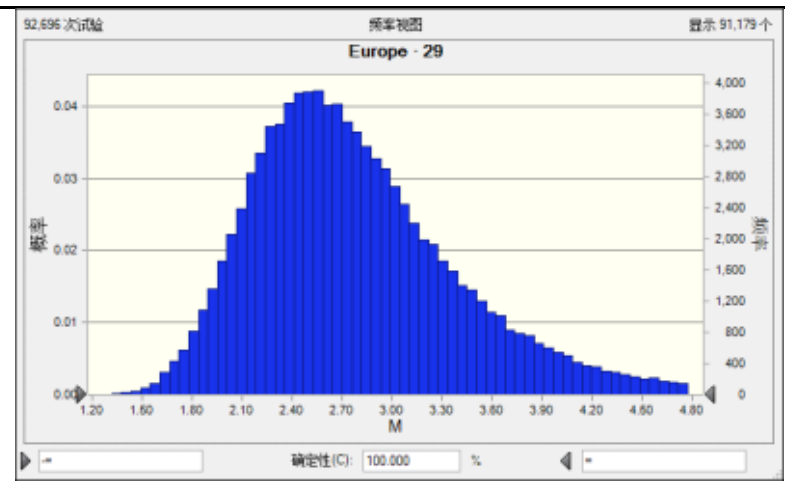	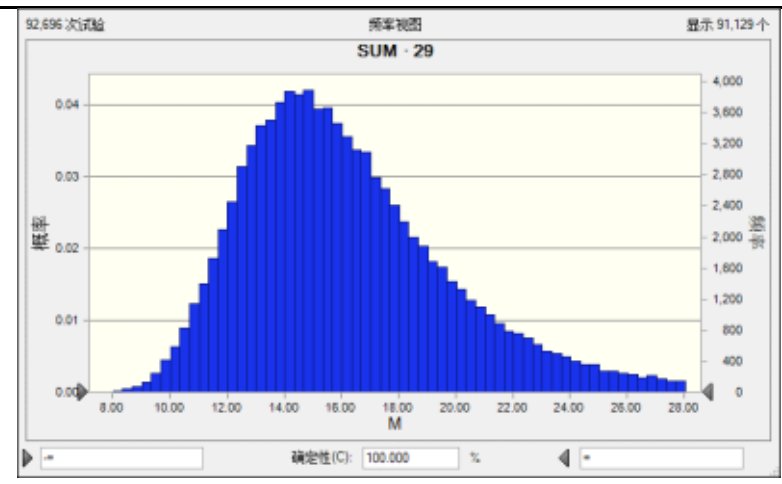
2011	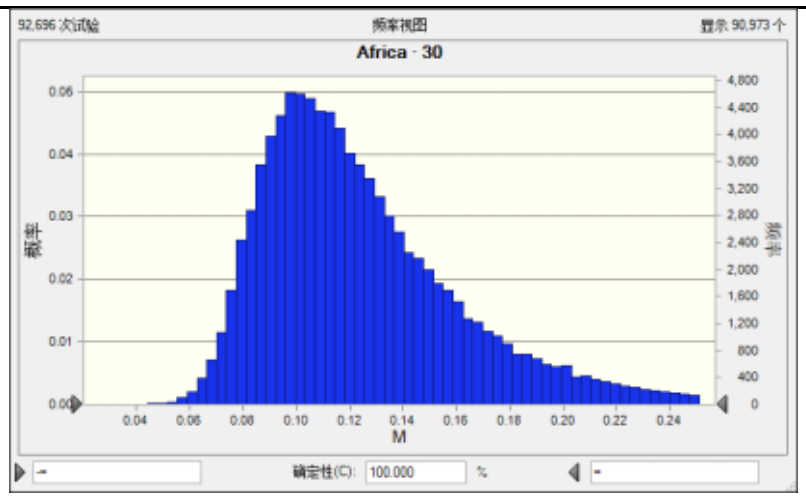	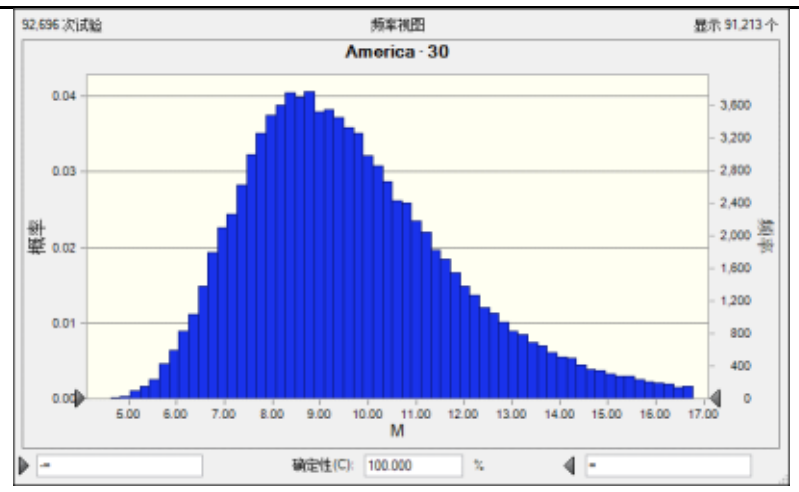	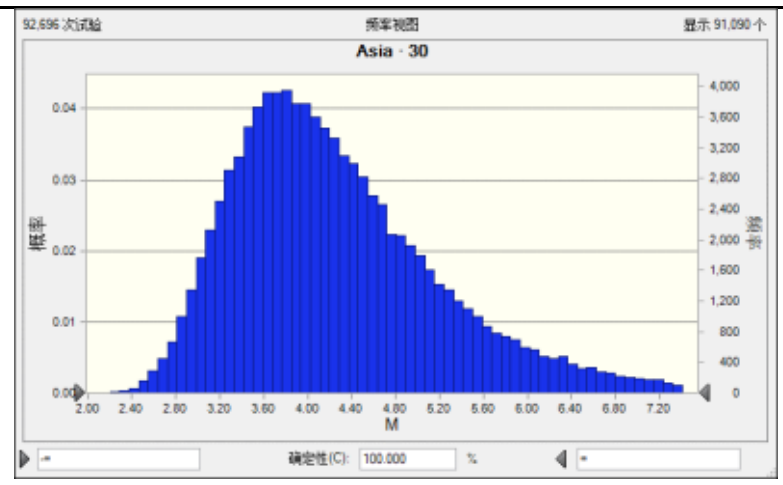	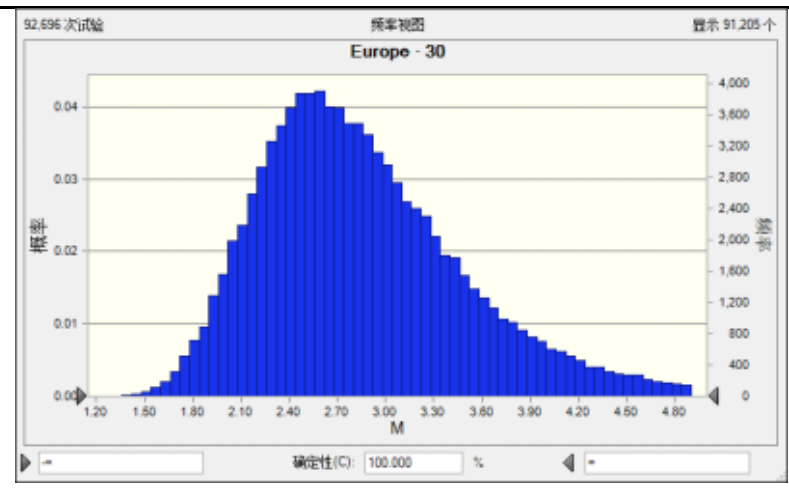	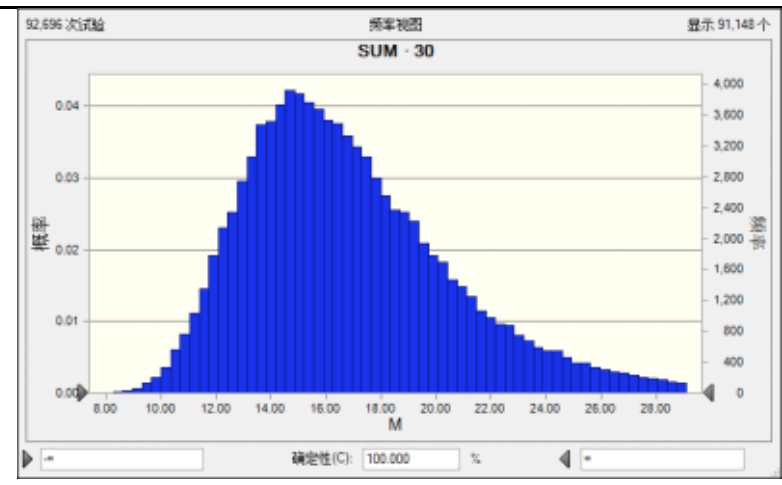

2012

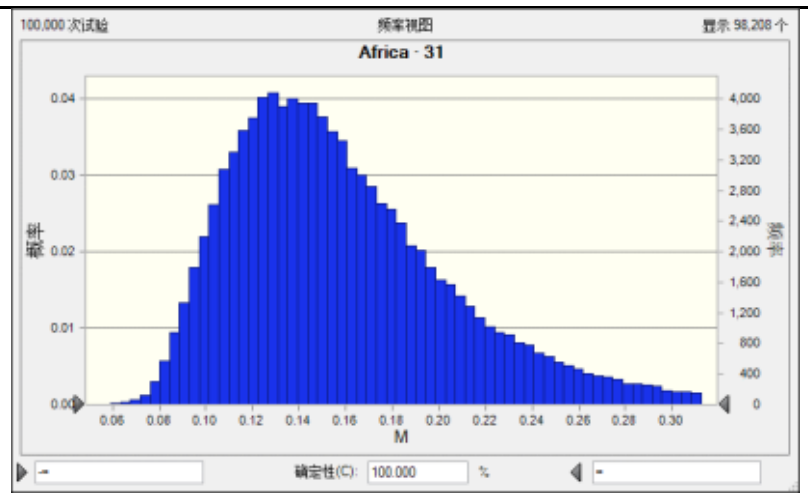

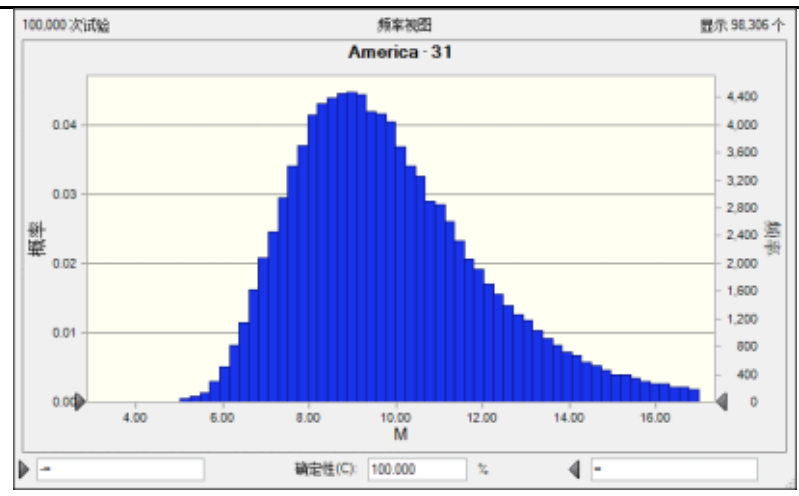

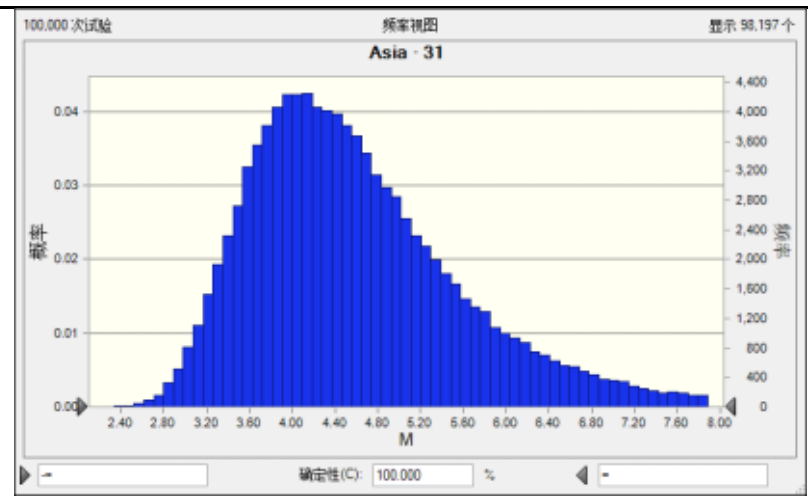

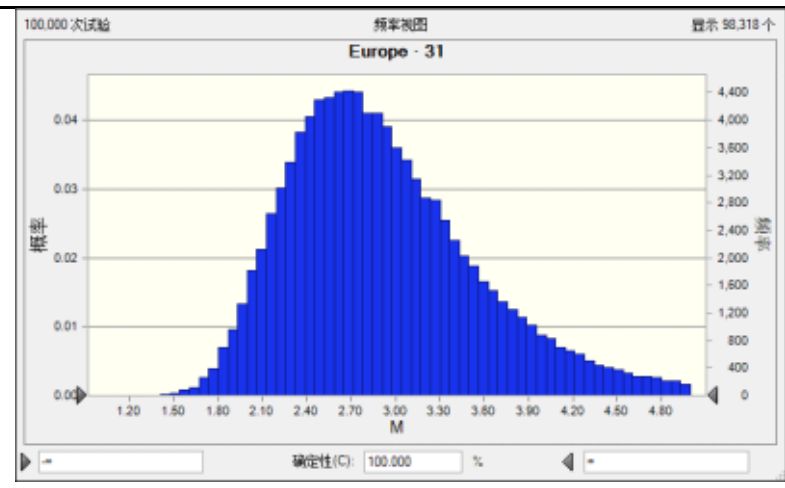

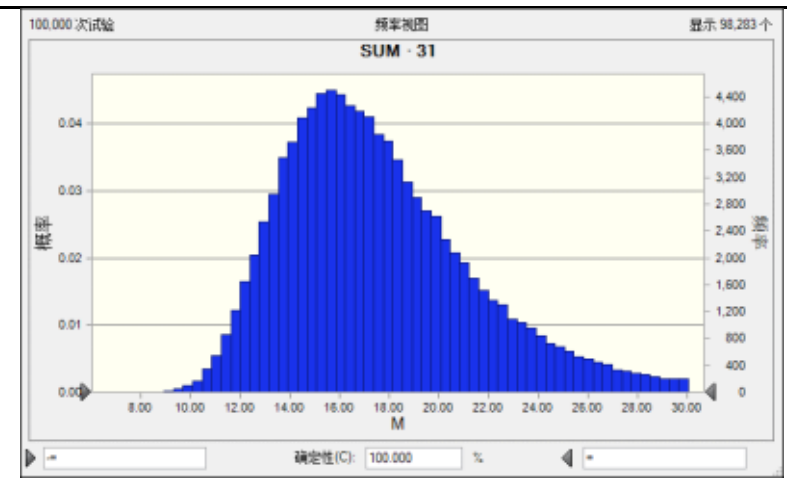

2013

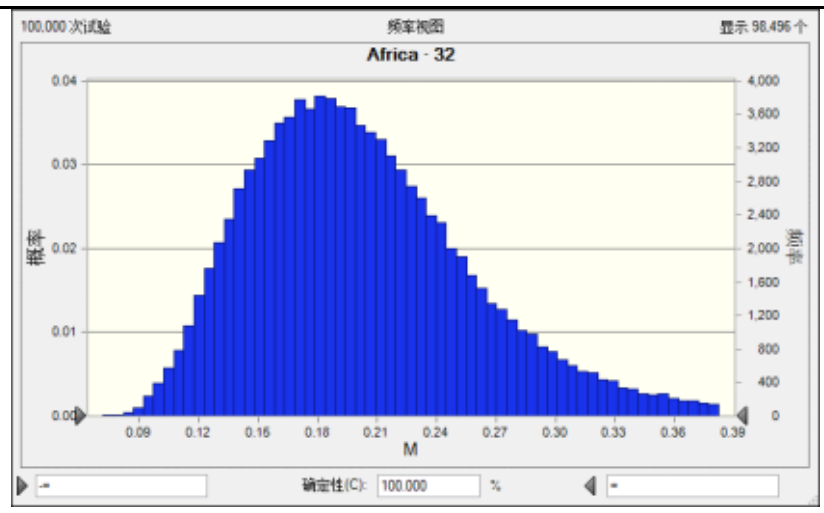

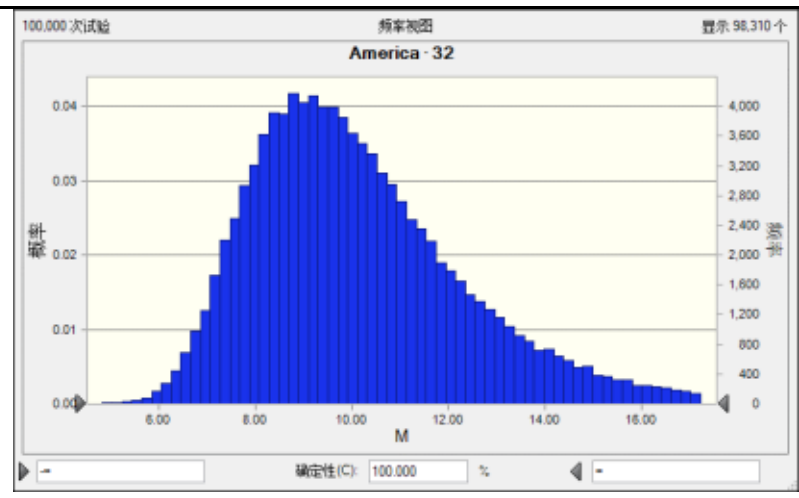

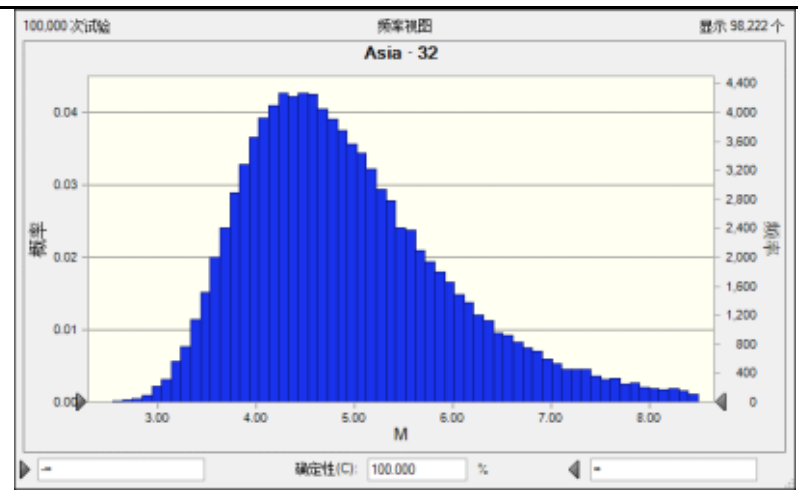

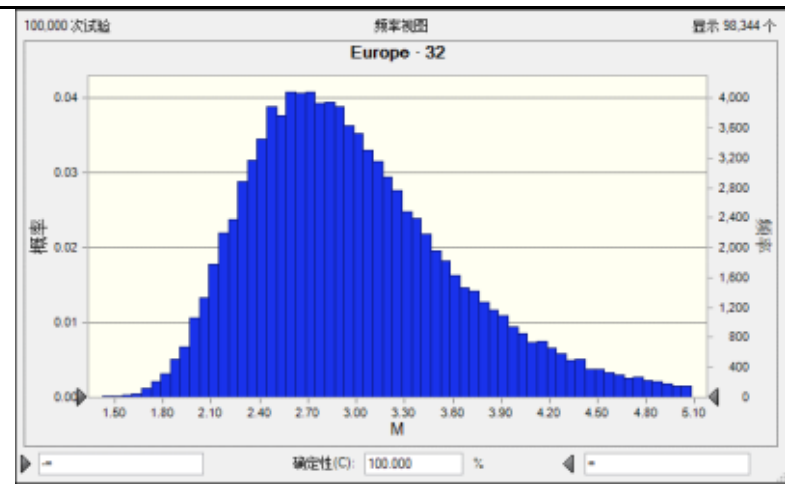

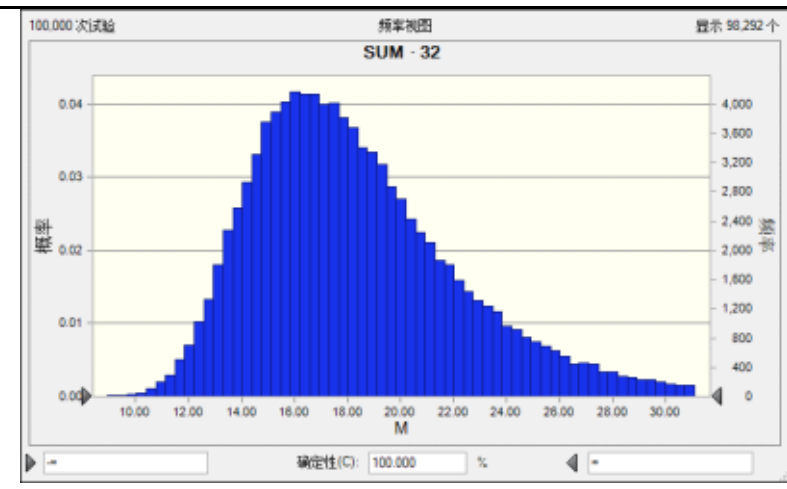

2014

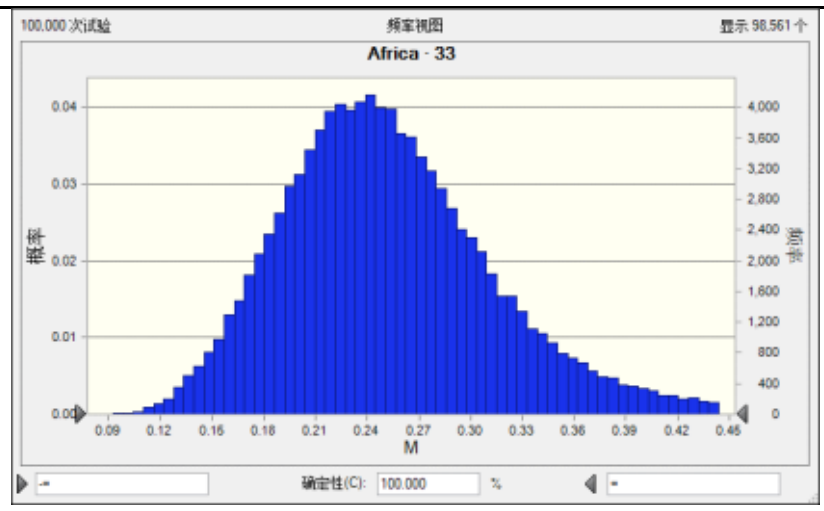

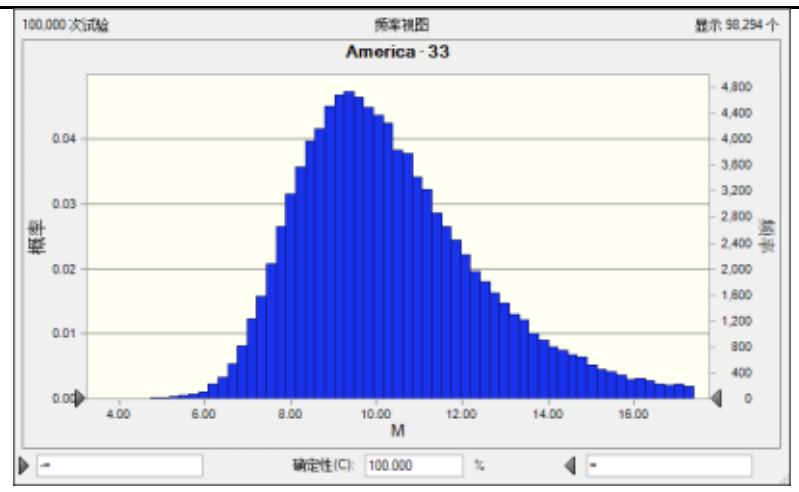

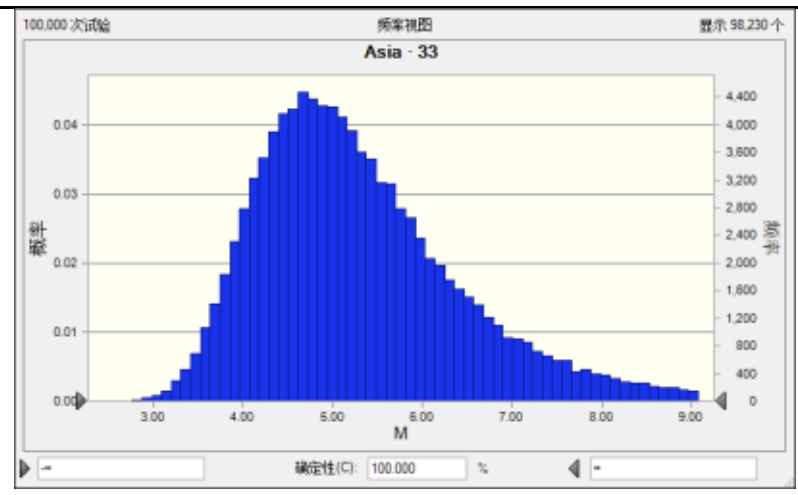

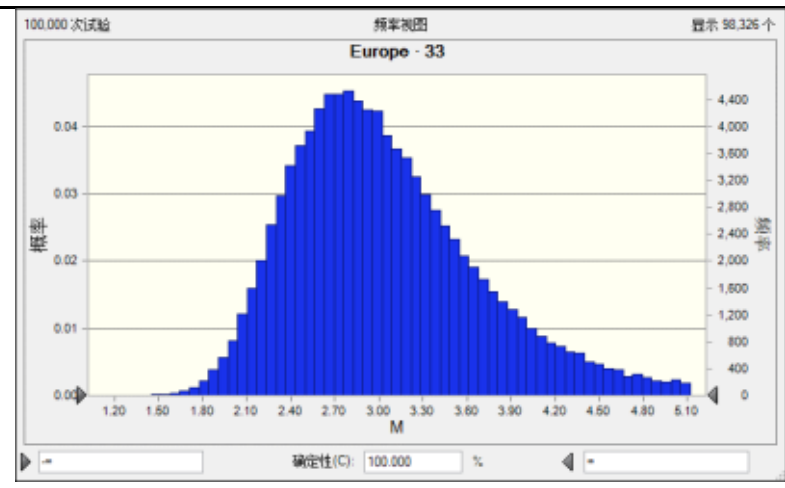

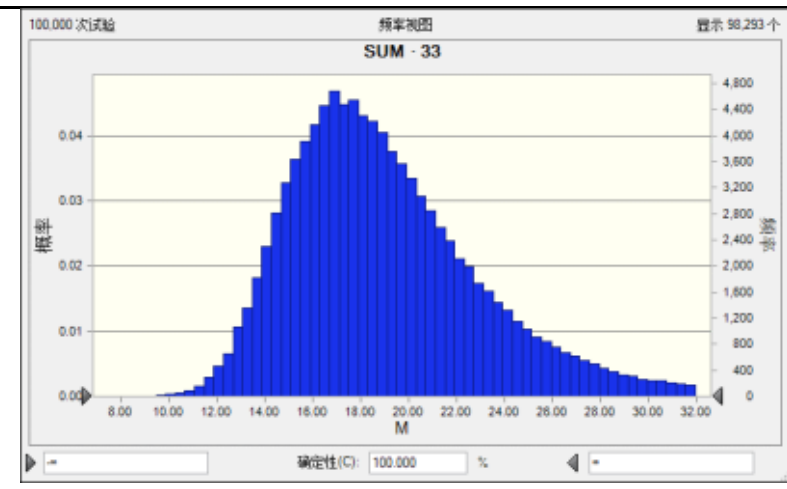

2015

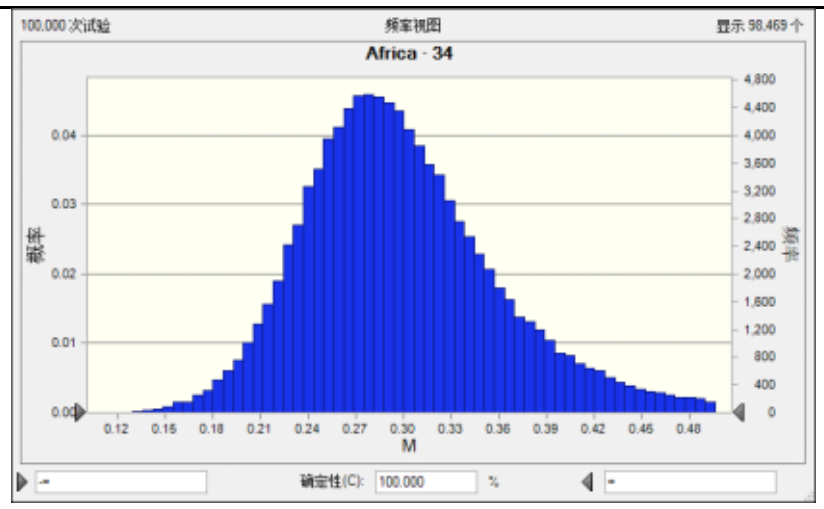

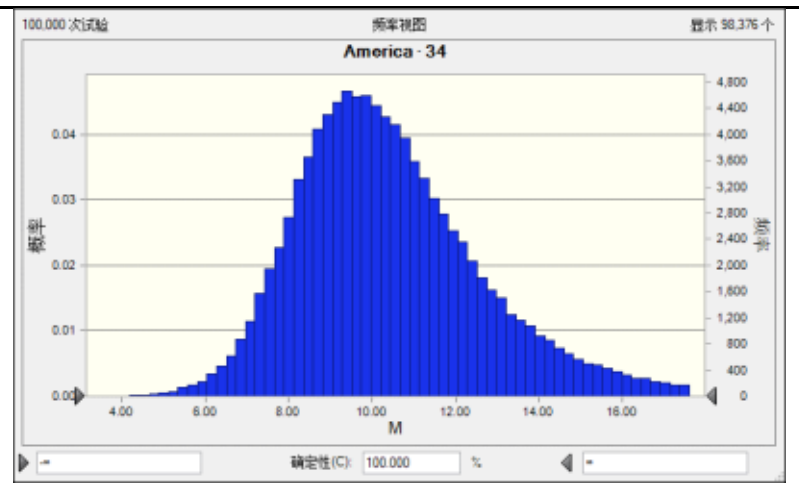

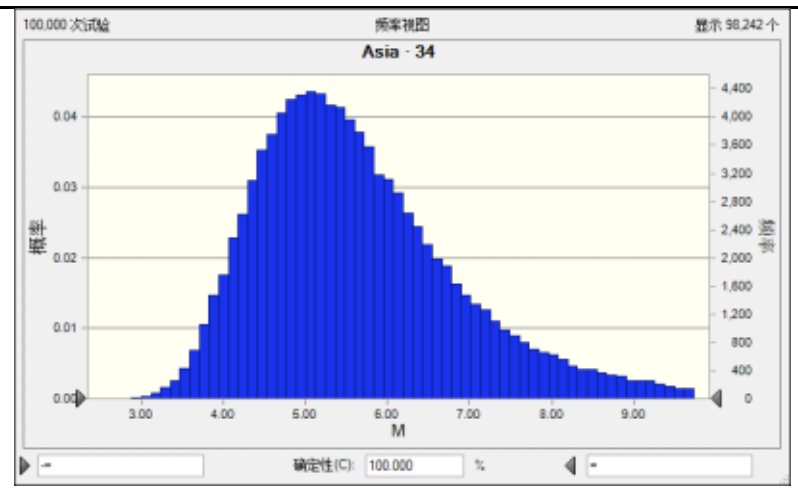

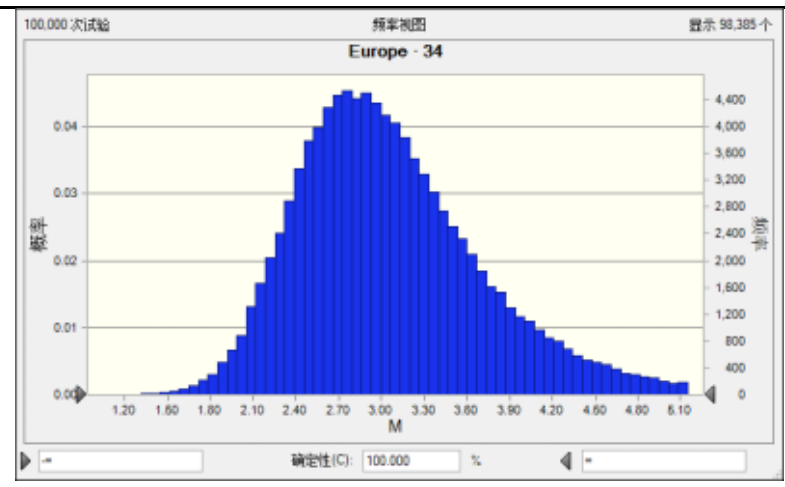

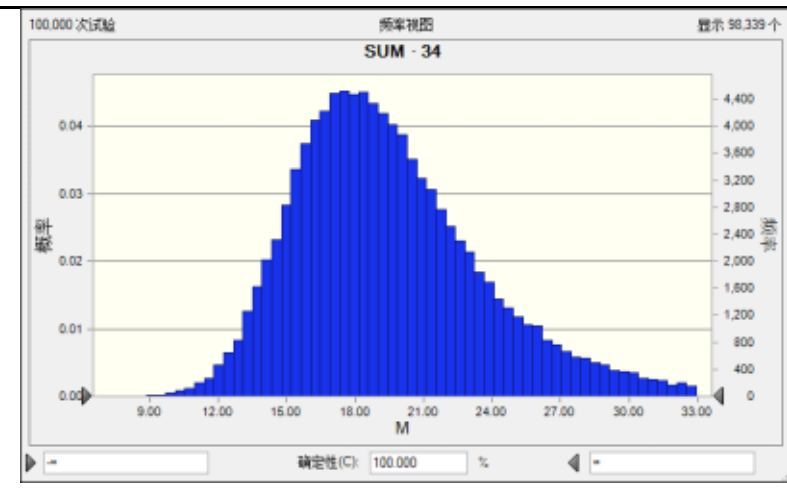

2016

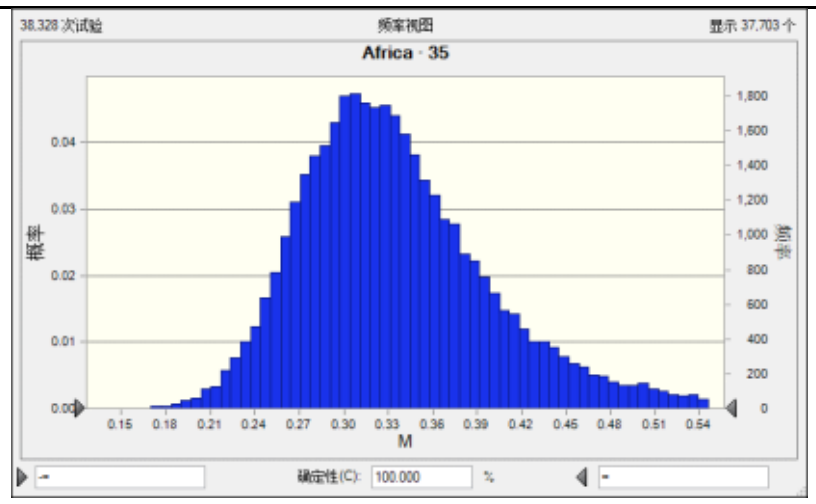

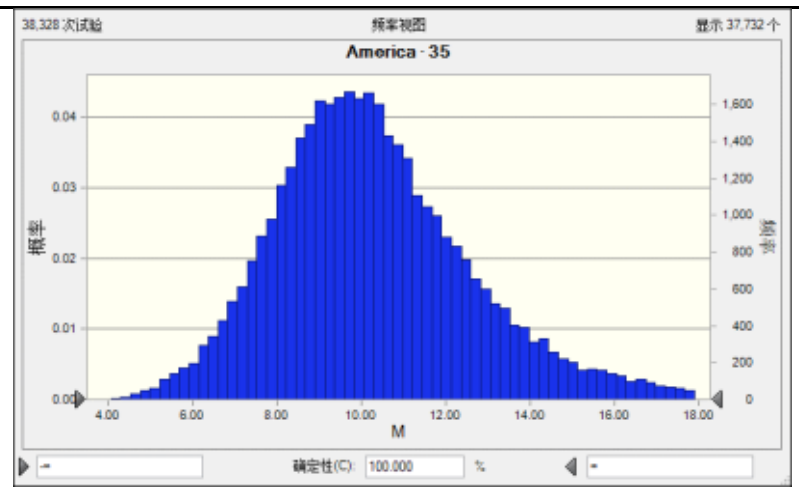

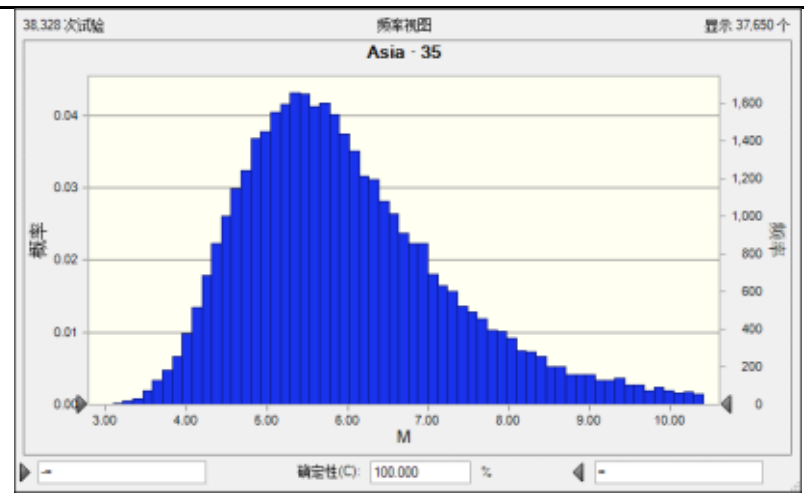

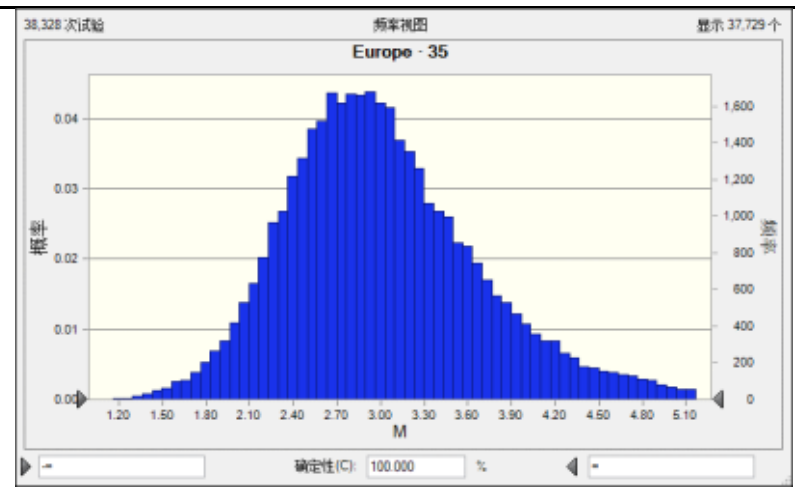

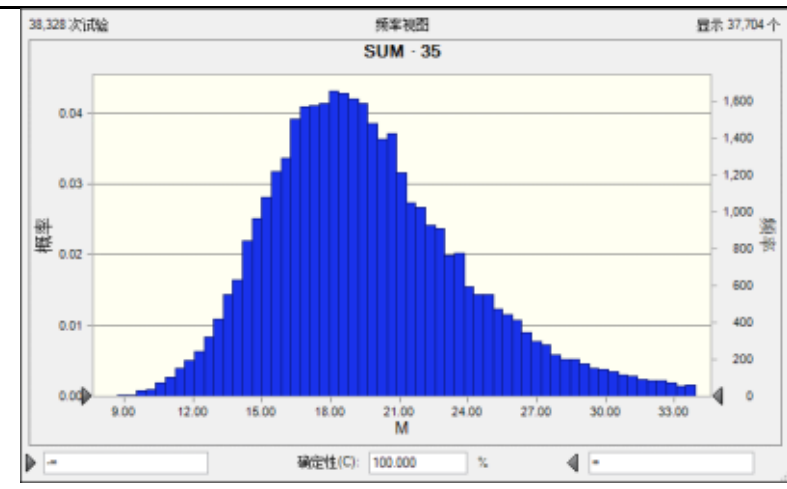

2017	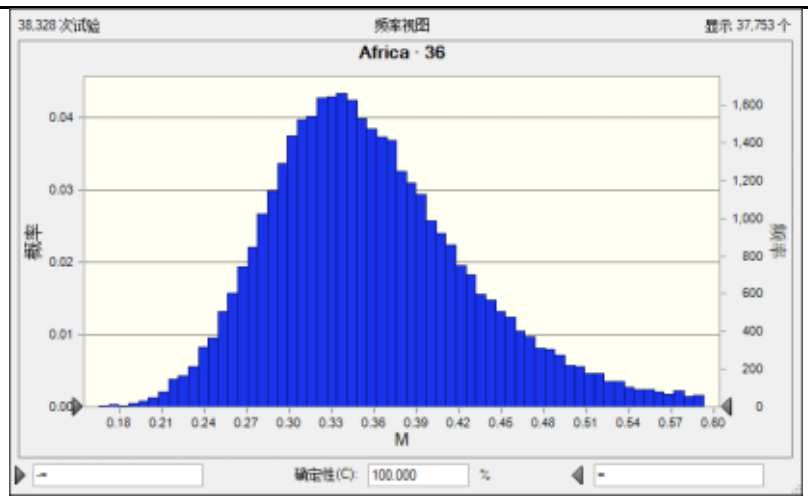	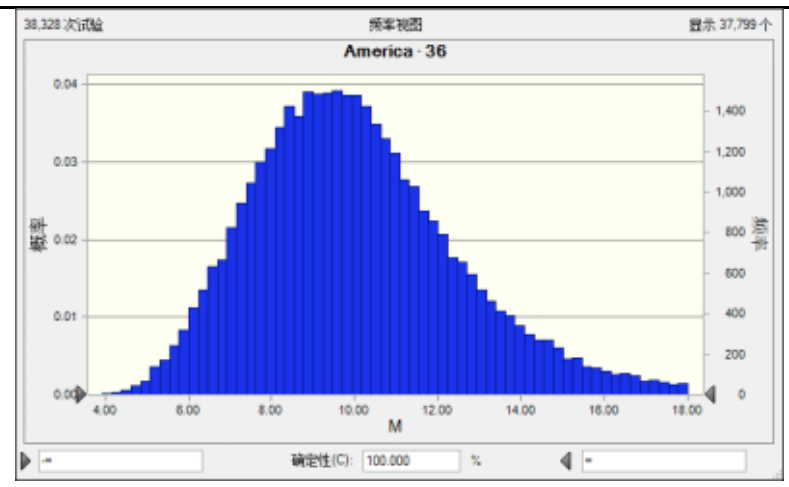	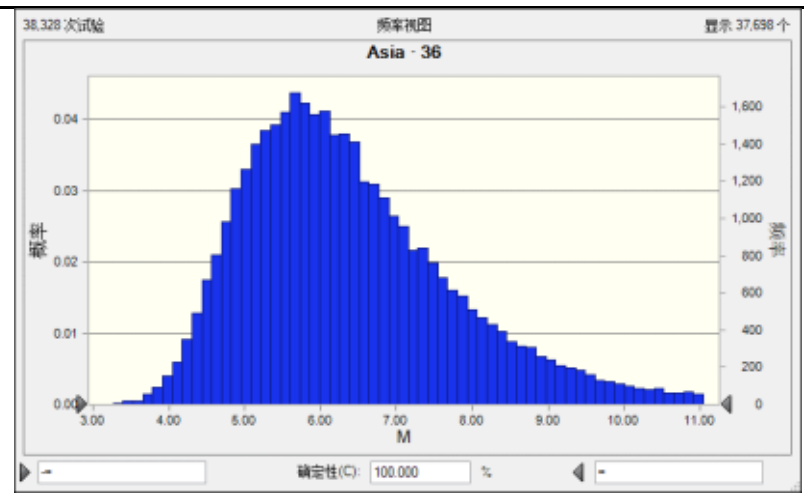	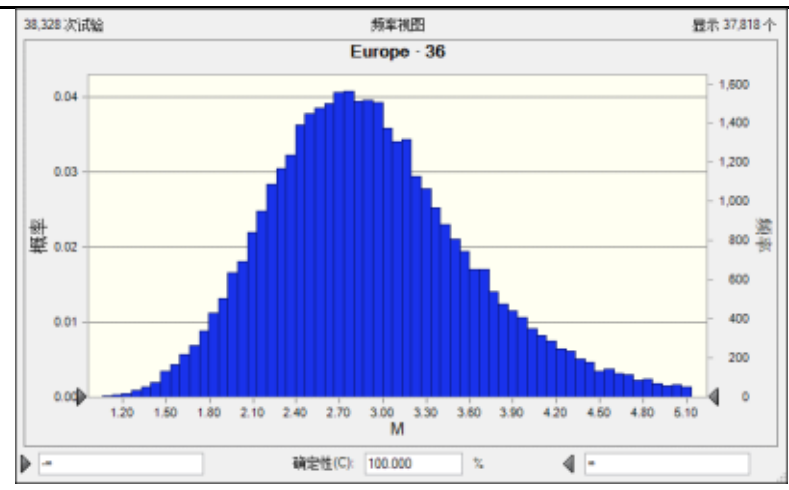	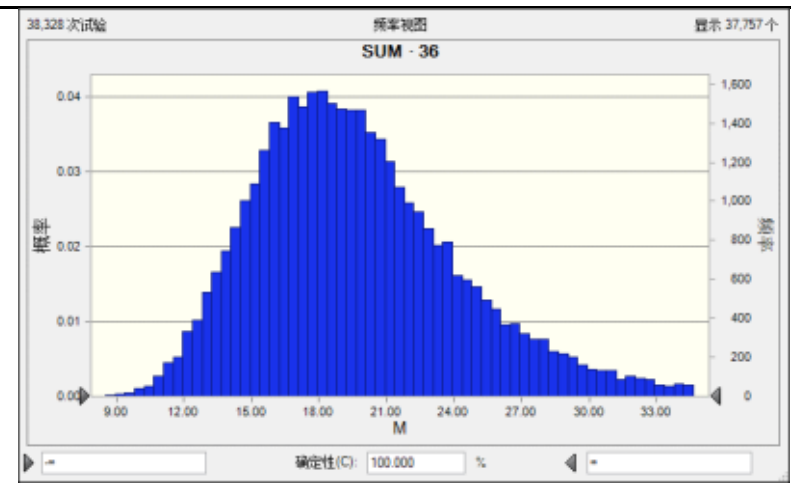
2018	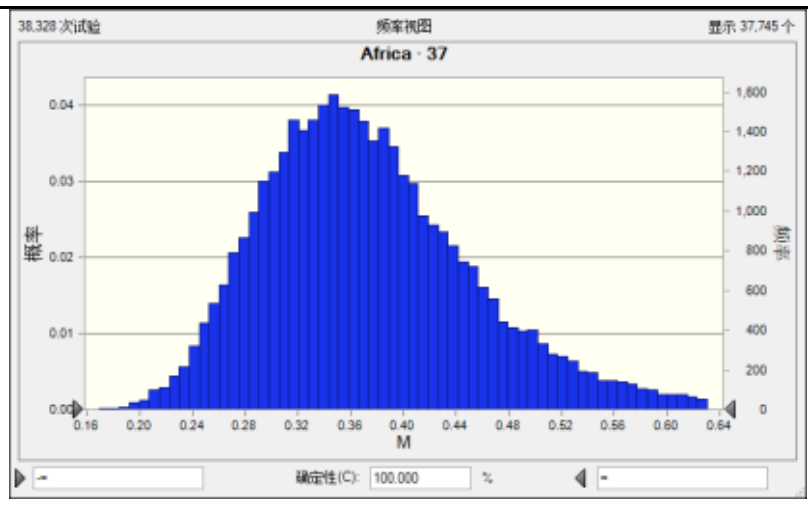	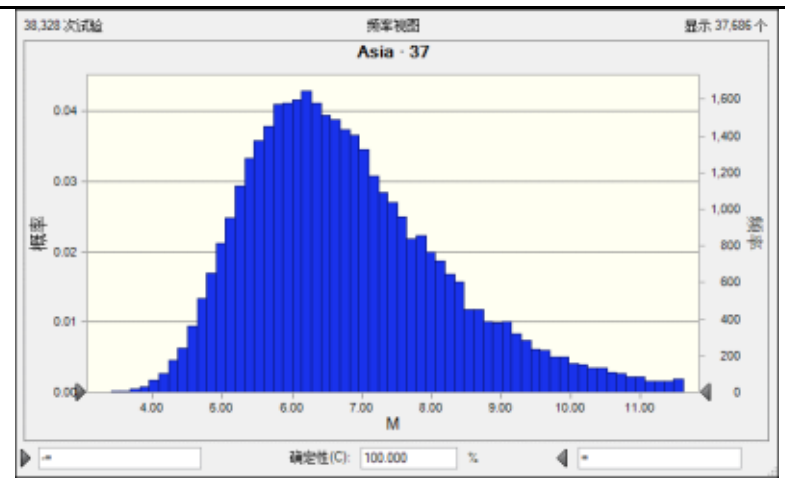	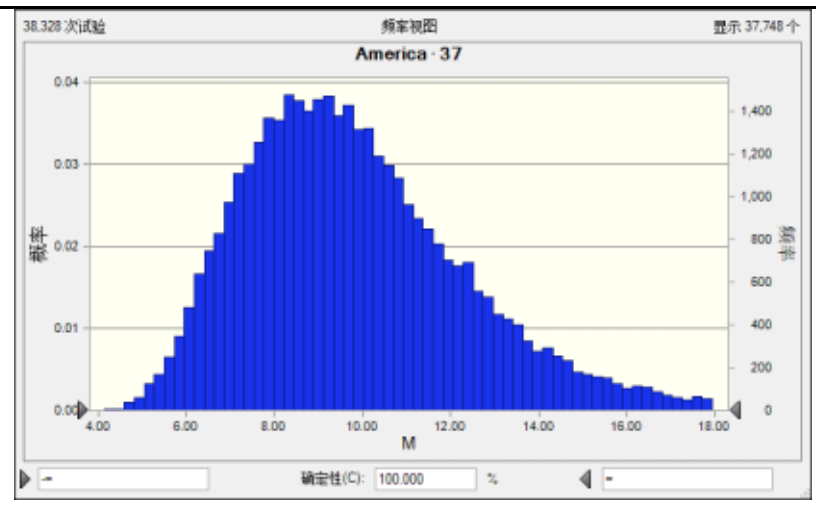	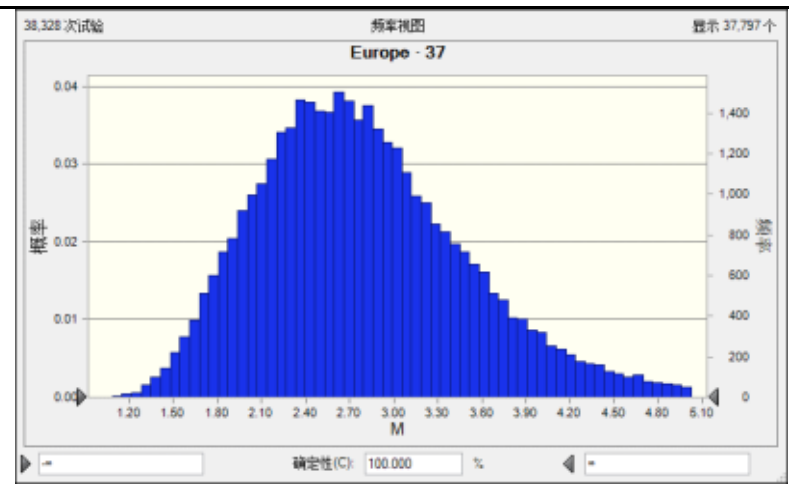	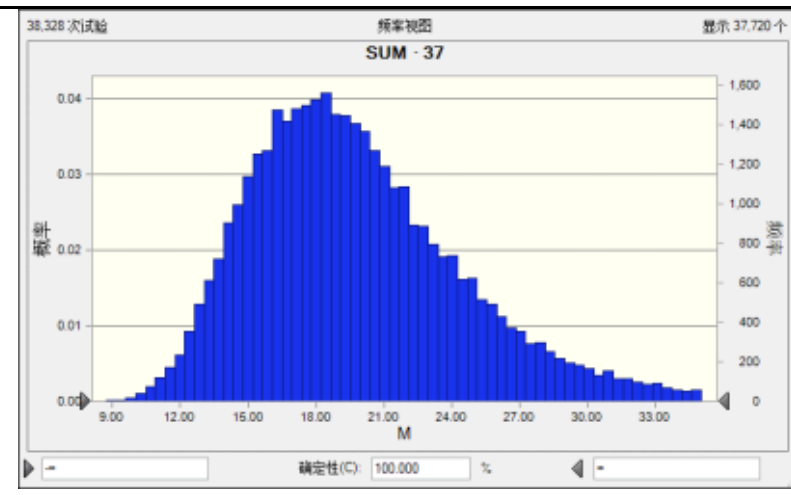
2019	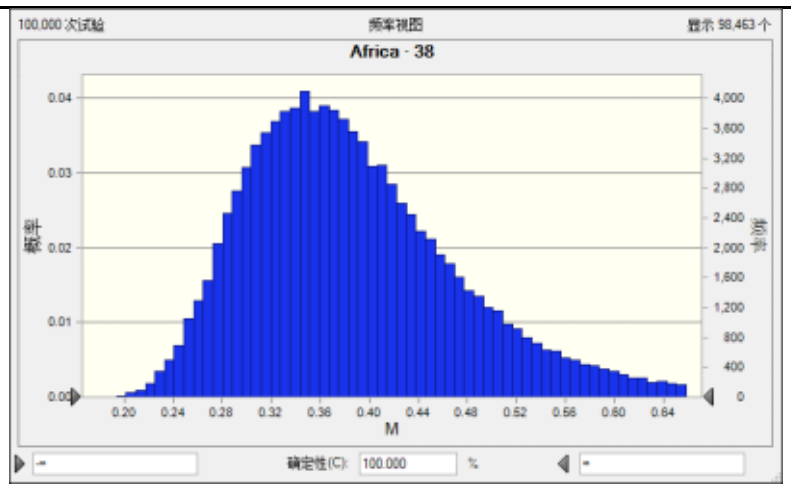	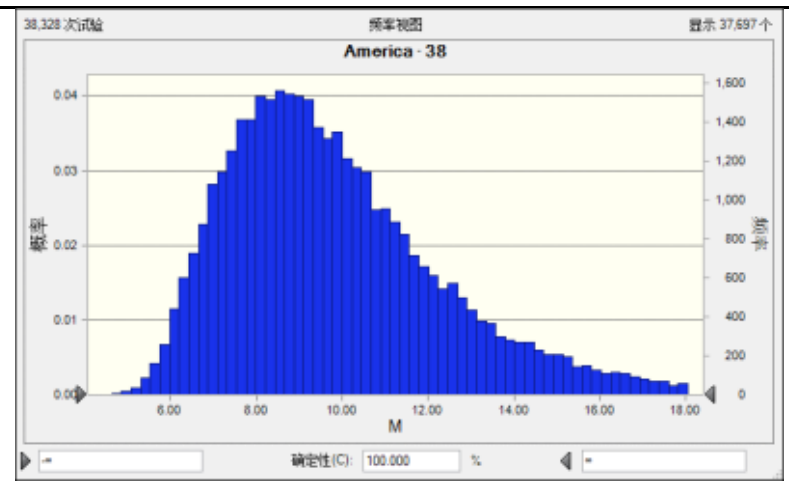	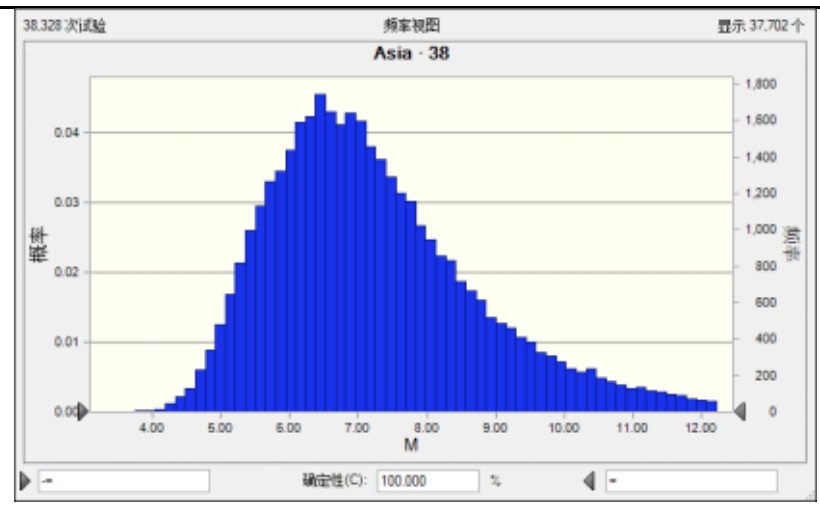	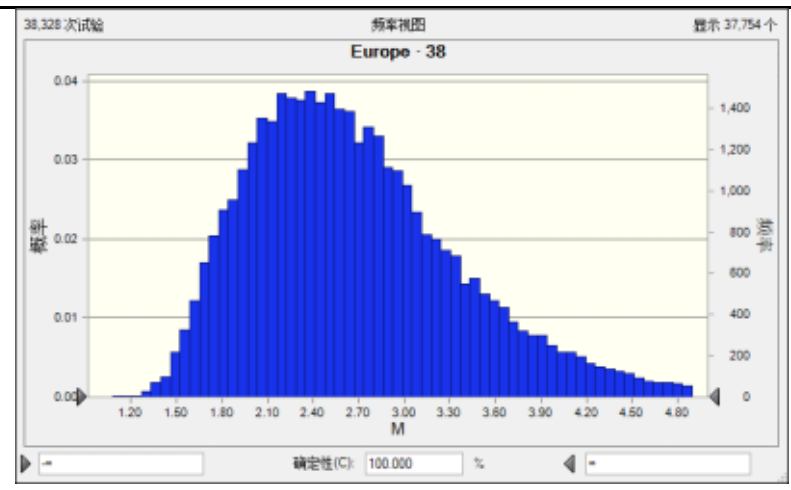	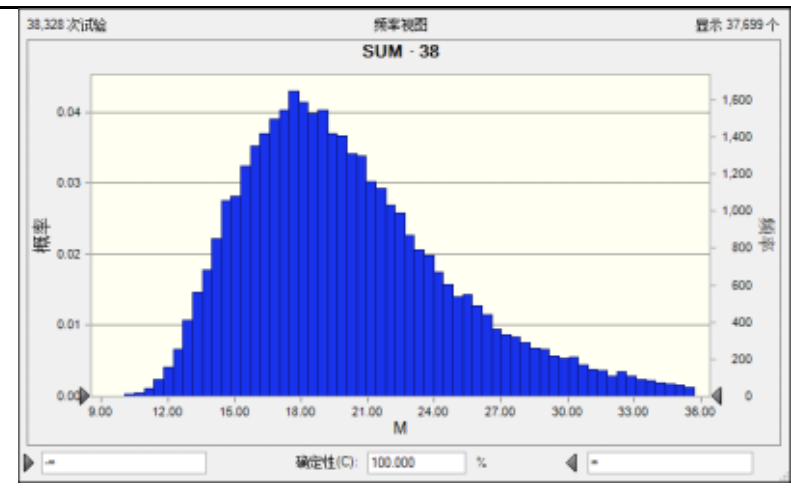
2020	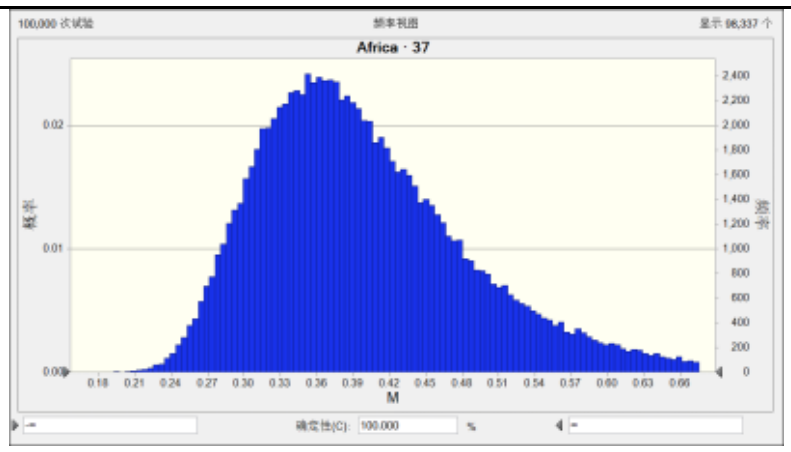	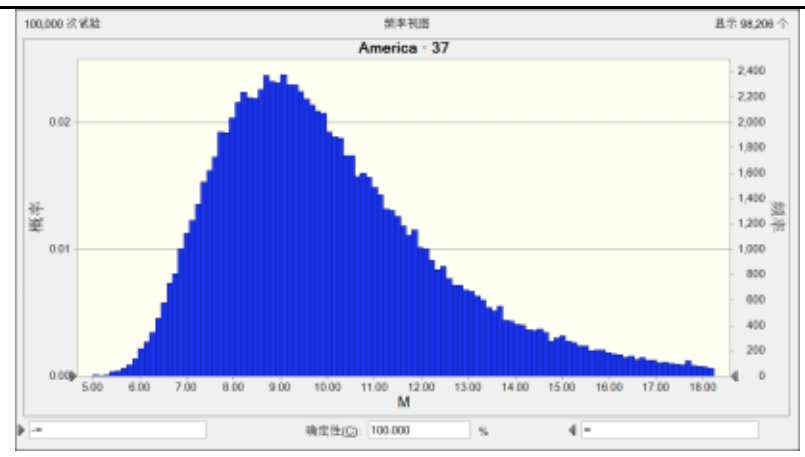	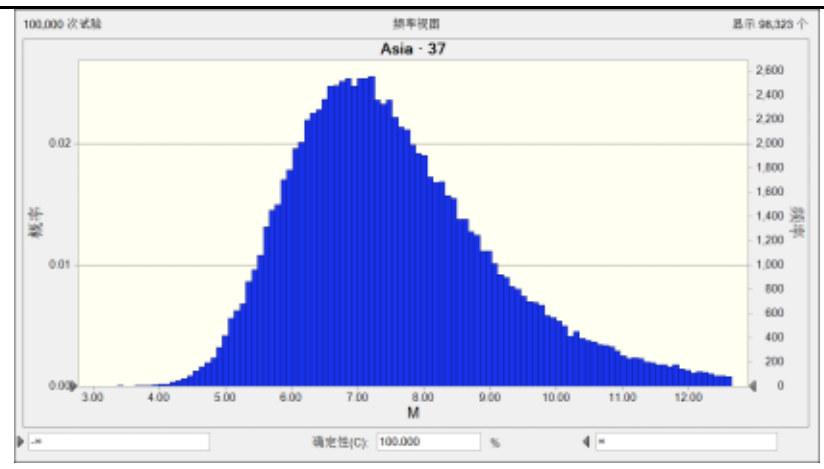	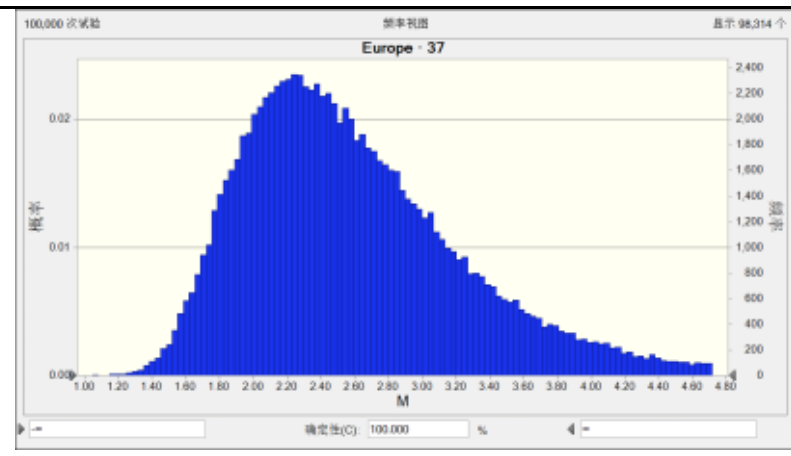	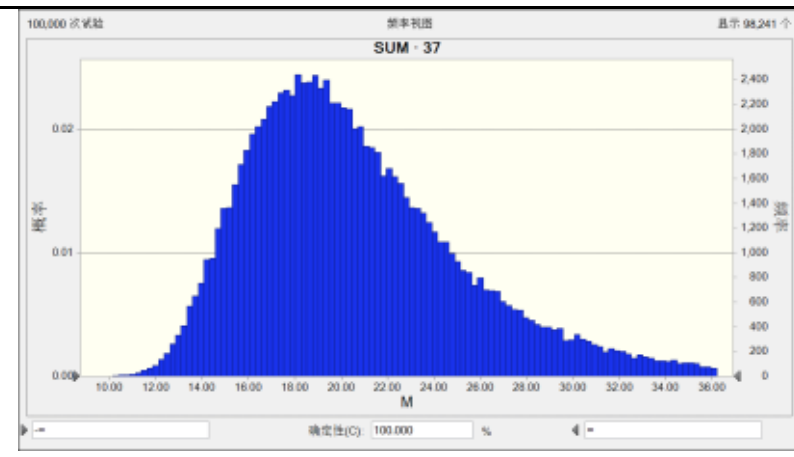
2021	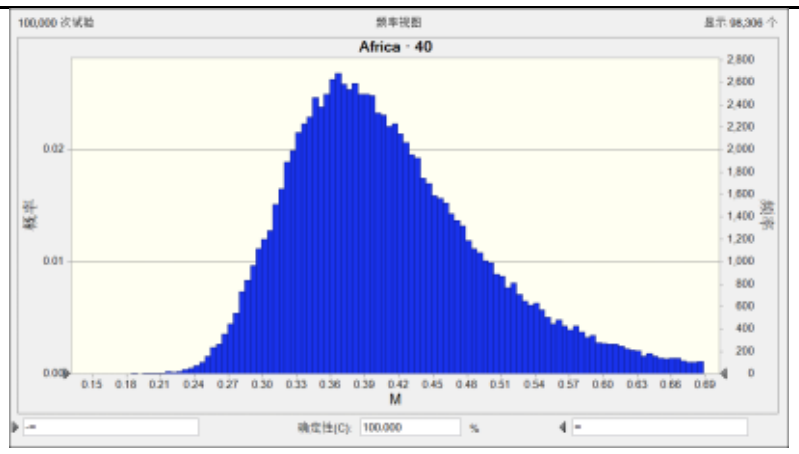	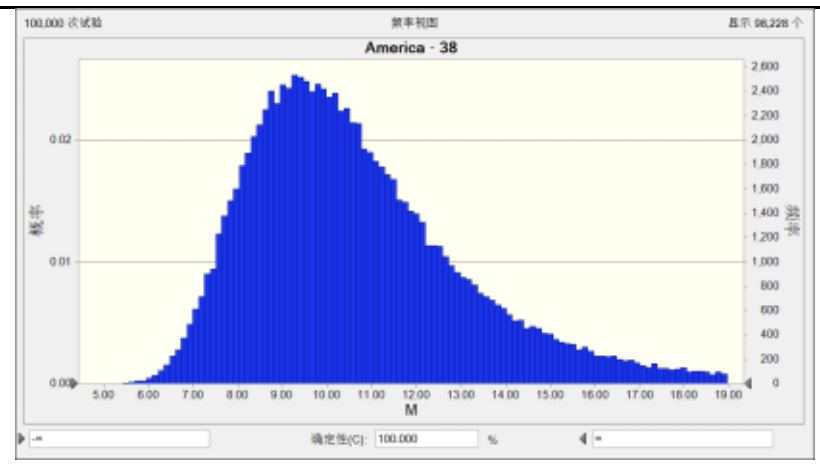	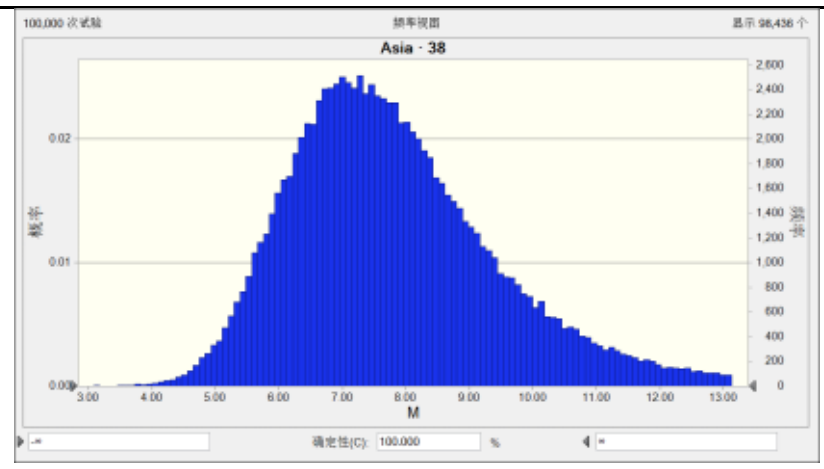	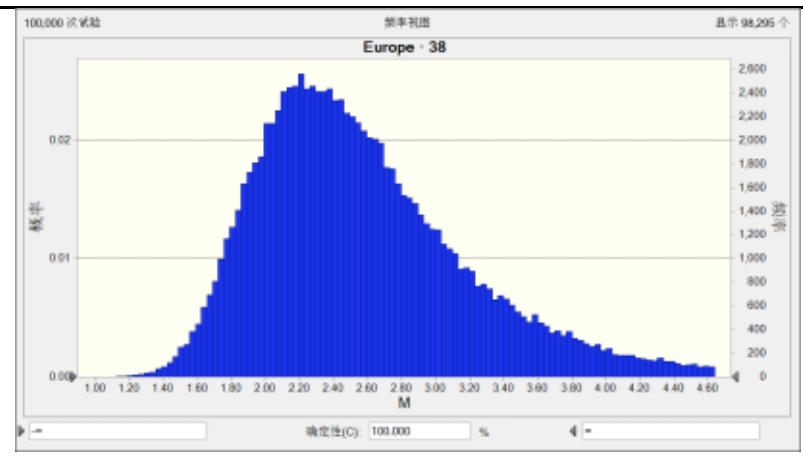	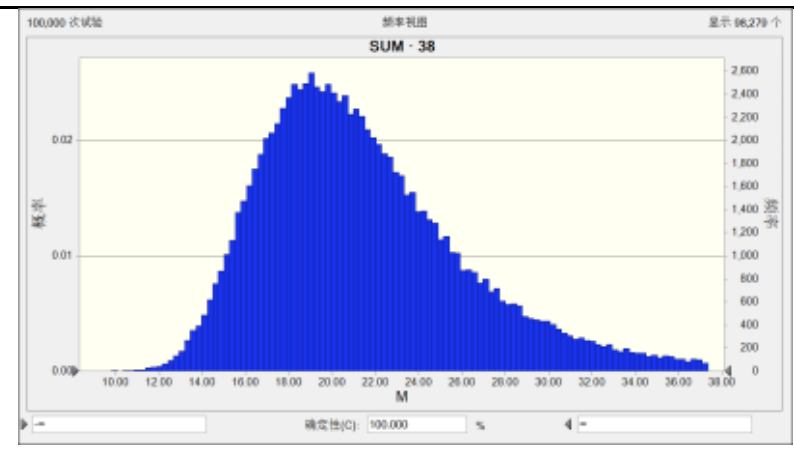

Table S15: Uncertainty analysis of EoL CVs generation in main countries in 2000-2025, Related to Figures 1, 2, and 3.

Year	U.S.	Japan	China	Germany	UK	France	India
2000	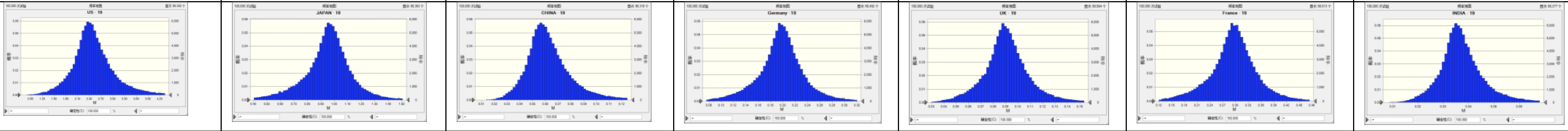						
2001	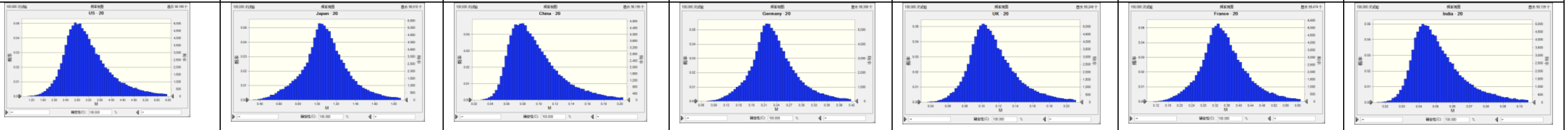						
2002	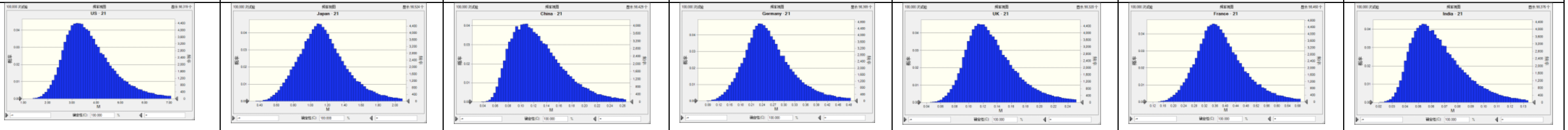						
2003	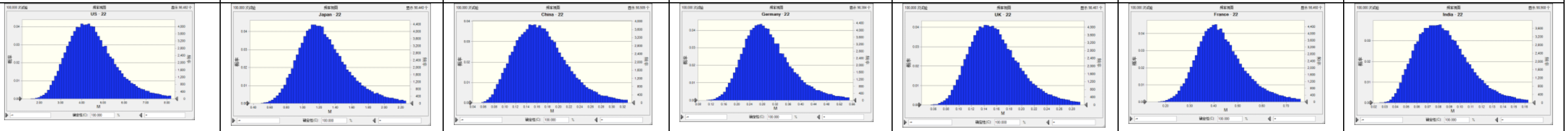						
2004	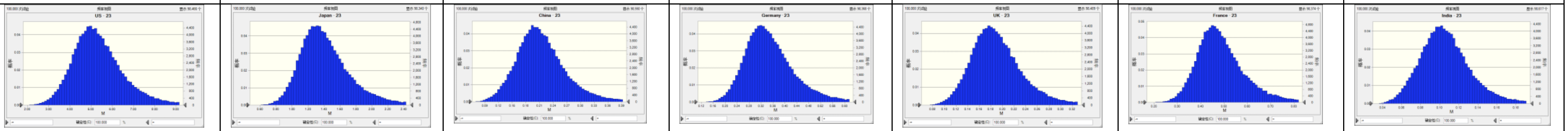						
2005	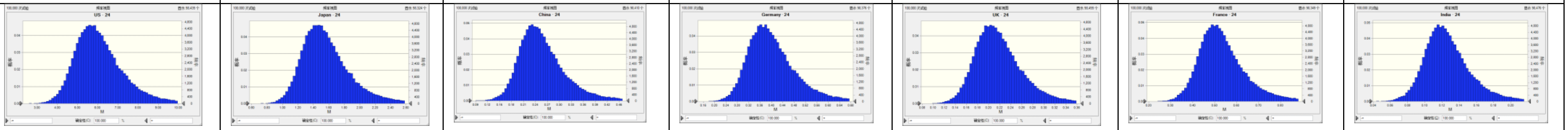						
2006	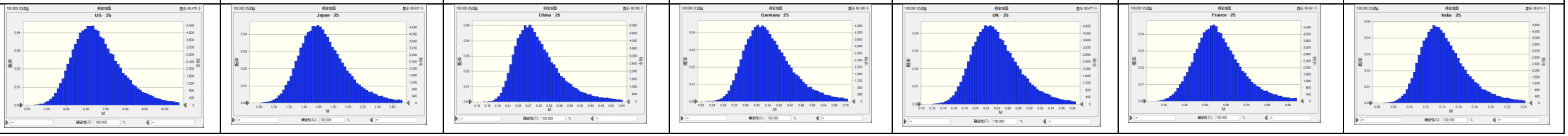						

2007	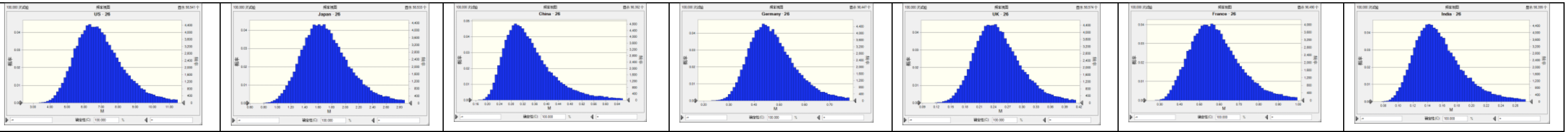
2008	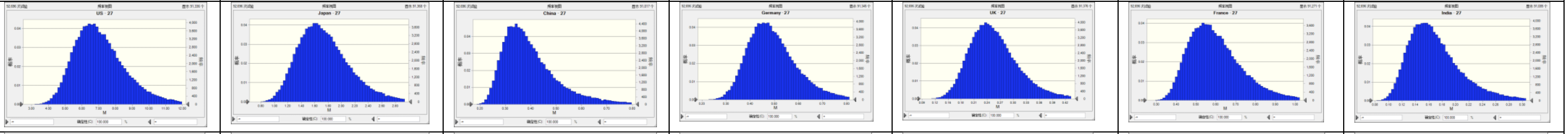
2009	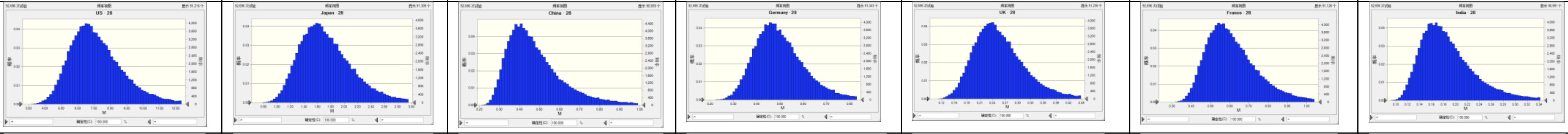
2010	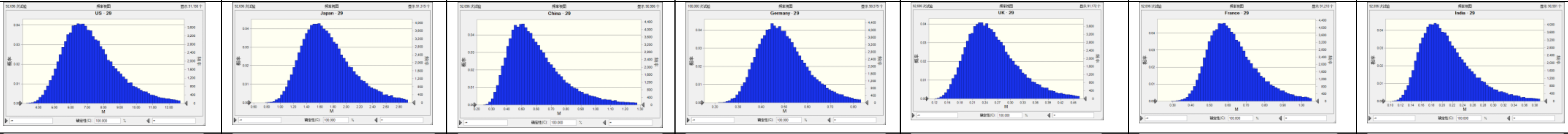
2011	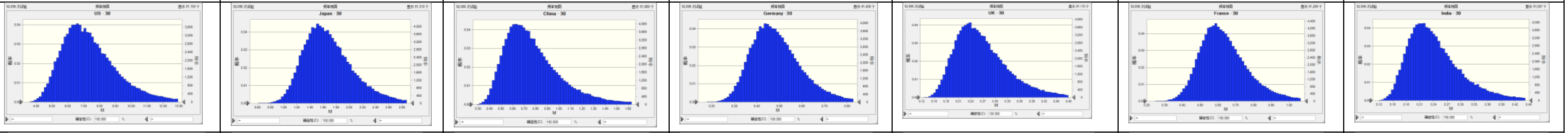
2012	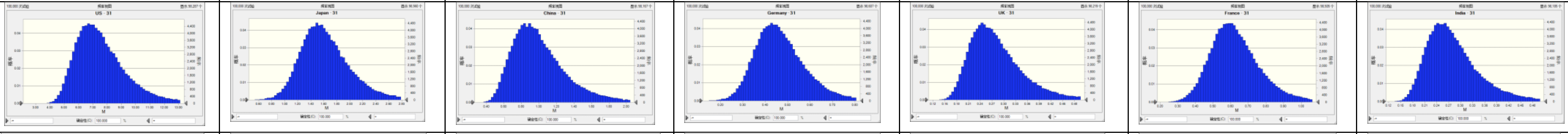
2013	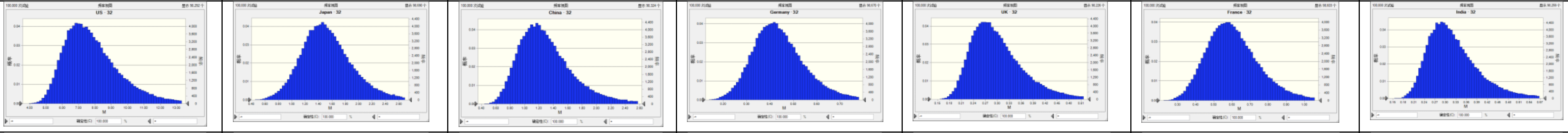
2014	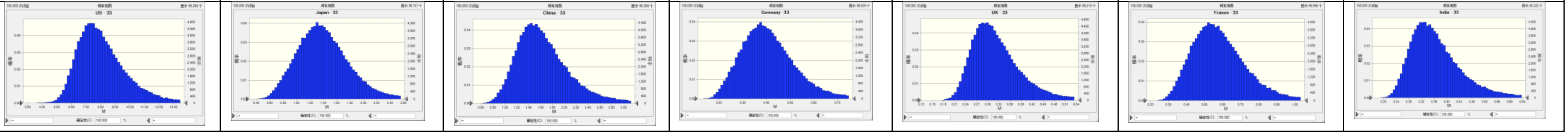

2015	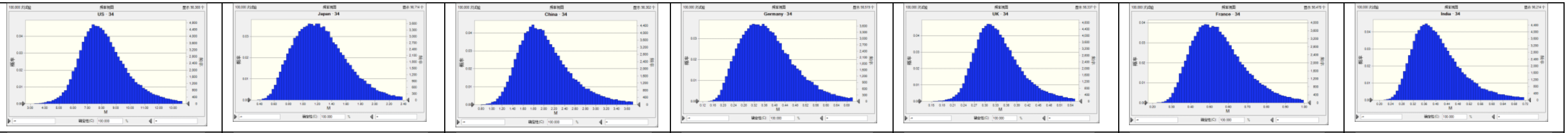
2016	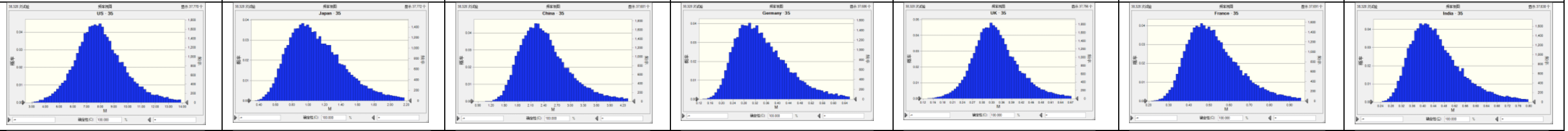
2017	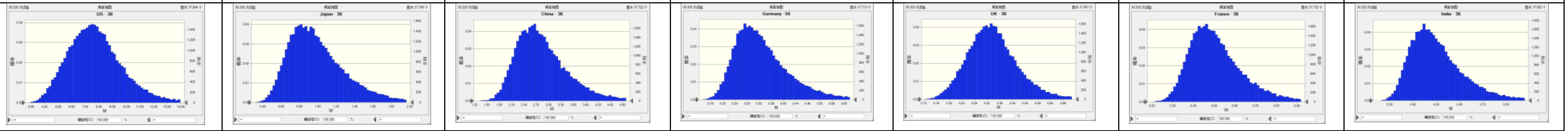
2018	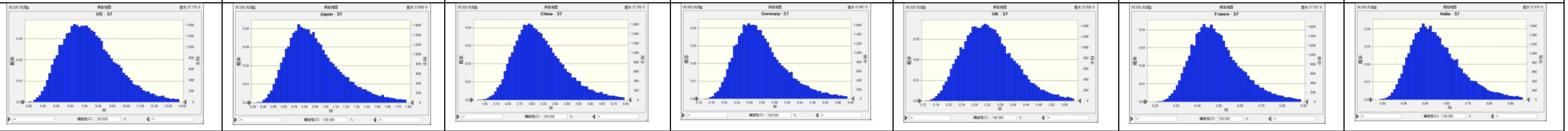
2019	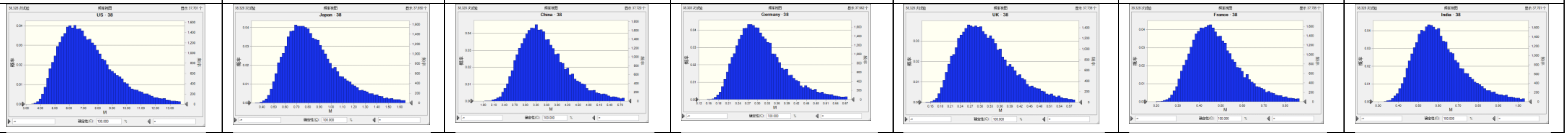
2020	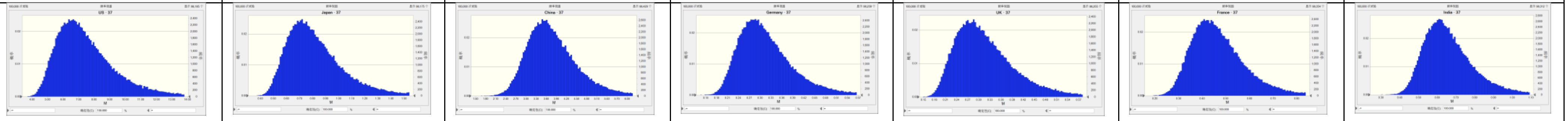
2021	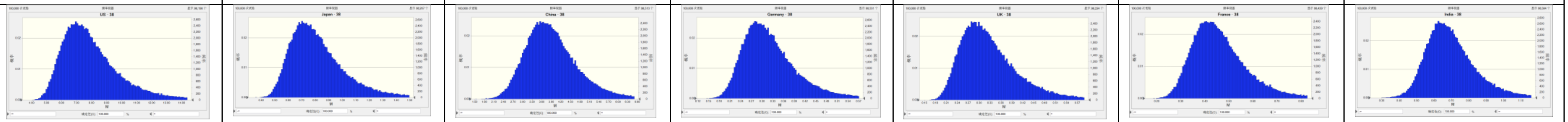
2022	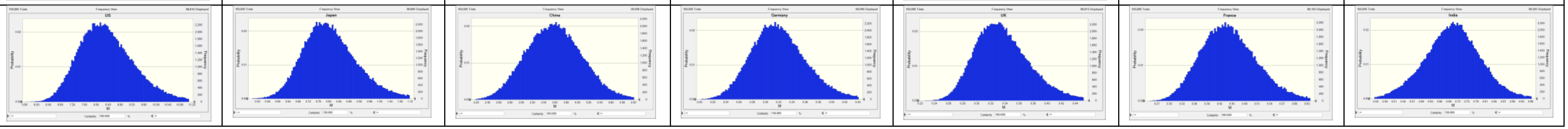

2023	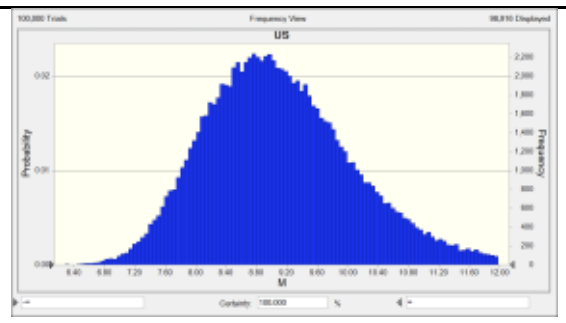	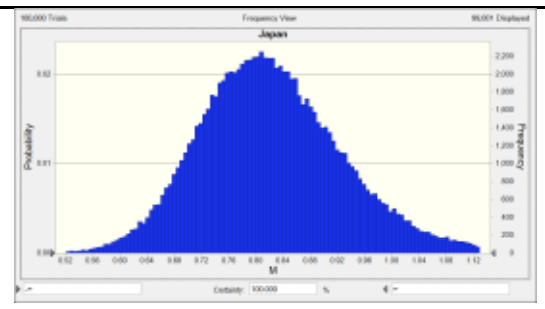	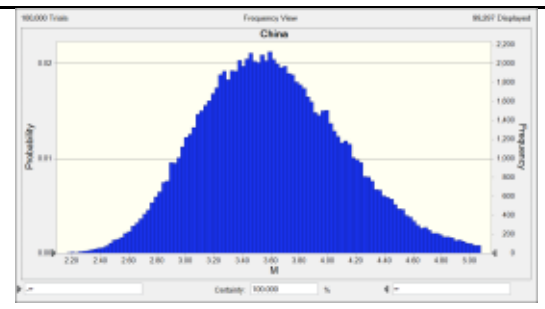	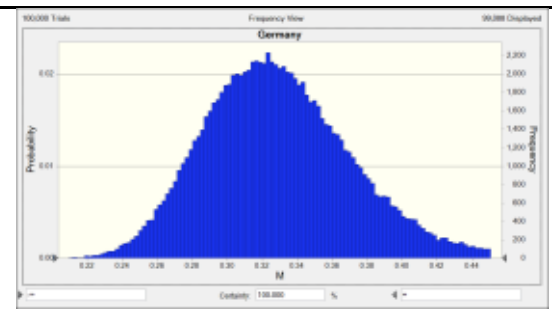	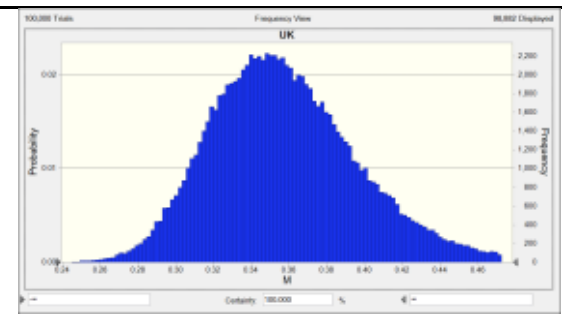	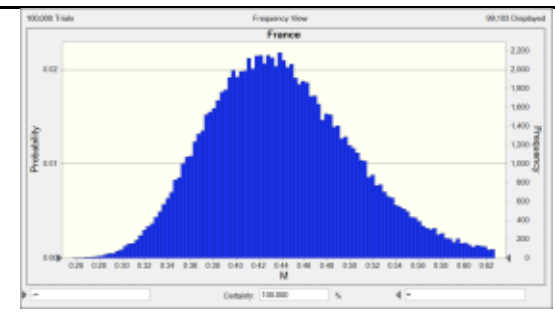	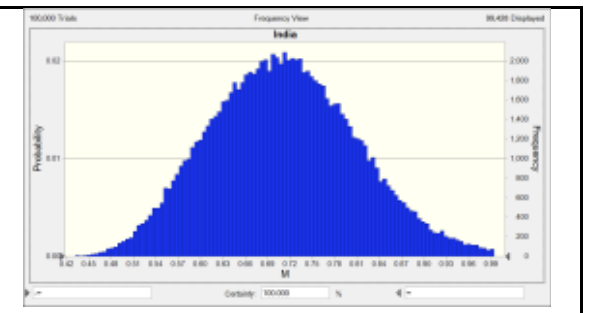
2024	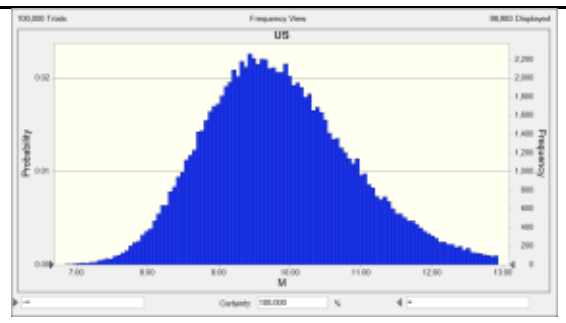	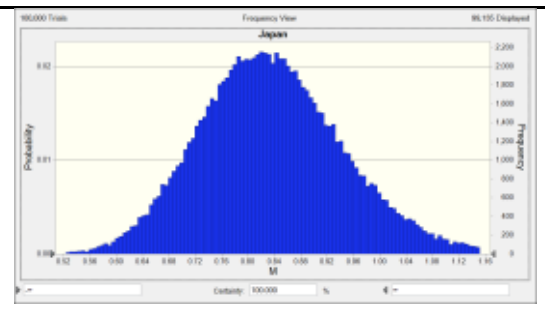	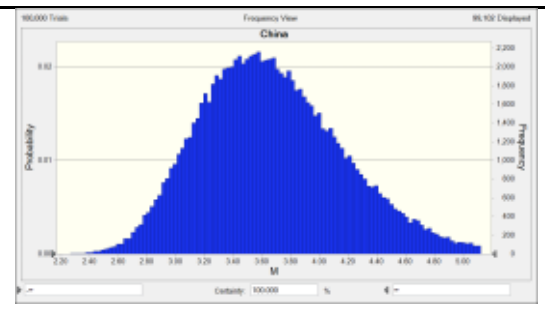	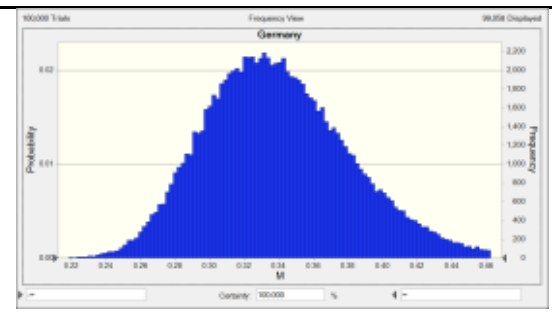	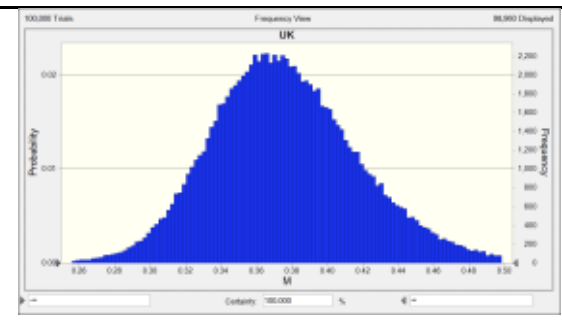	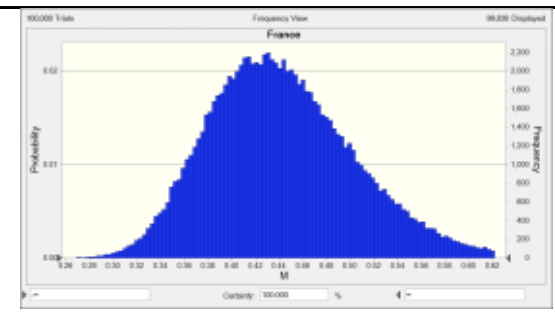	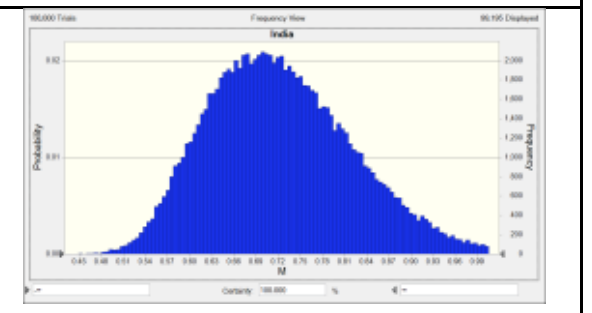
2025	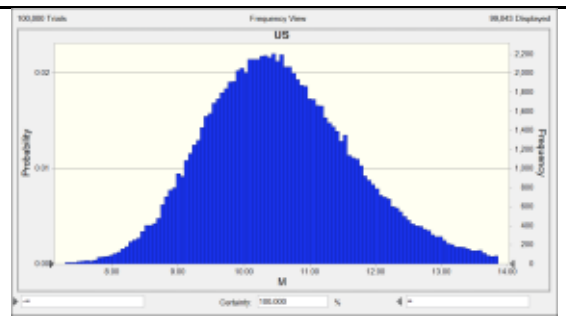	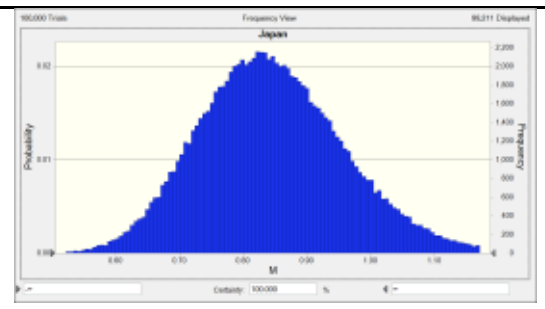	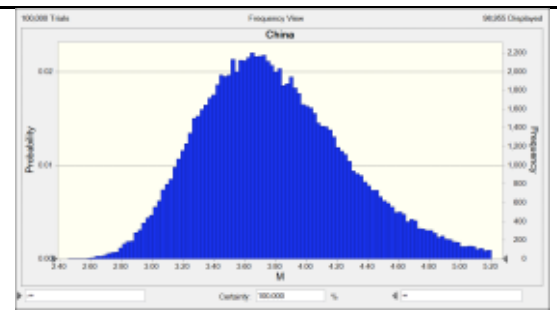	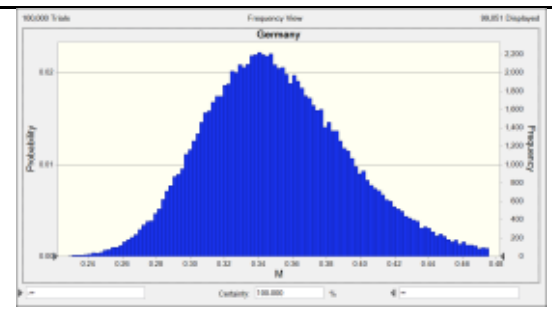	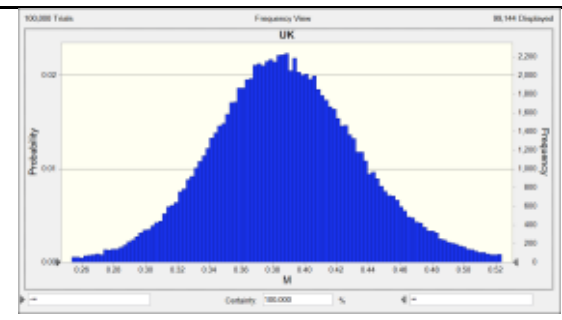	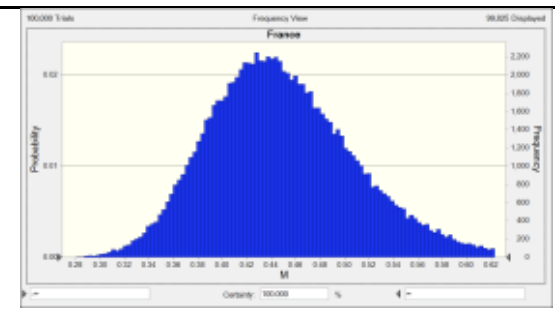	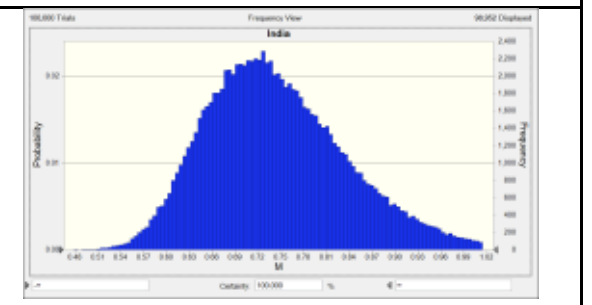

Table S16: Comparison between this study and other works in PCs, Related to Figure 4.

Year	UK			France			China					Italy			Netherlands			Japan				
	This study-Mean	This study-error	Reported data	This study-Mean	This study-error	Reported data	This study-Mean	This study-error	Xi et al, 2014 [13]	Zeng et al., 2020 [3]	Zhang et al., 2021 [14]	This study-Mean	This study-error	Reported data	This study	This study-error	Reported data	This study-Mean	This study-error	Yano et al., 2014 [15]	Xu et al., 2019 [16]	JARC*
2006	1.14	0.55	1.00	2.01	0.9	0.93	0.90	0.4	1.72		1.72	1.12	0.5	1.38	0.11	0.05	0.19	6.62	2.7	4.1		
2007	1.26	0.6	1.14	2.17	0.9	0.95	1.09	0.4	1.88		1.88	1.20	0.5	1.69	0.13	0.05	0.17	7.07	3	4.4		
2008	1.37	0.6	1.21	2.30	1.1	1.11	1.30	0.5	1.70		1.70	1.28	0.6	1.20	0.15	0.06	0.15	7.43	3.3	4		
2009	1.47	0.7	1.33	2.42	1.2	1.57	1.54	0.6	2.08		2.08	1.34	0.6	1.61	0.17	0.06	0.19	7.70	3.5	3.9		
2010	1.55	0.7	1.16	2.50	1.3	1.58	1.83	0.6	2.72	0.76	3.10	1.39	0.6	1.25	0.19	0.07	0.23	7.86	3.8	3.8		
2011	1.62	0.8	1.22	2.56	1.3	1.52	2.17	0.7	3.21	0.95	3.15	1.44	0.7	0.95	0.21	0.07	0.20	7.89	4	3.7		
2012	1.69	0.8	1.16	2.59	1.5	1.21	2.57	0.8		1.19	3.66	1.49	0.7	0.90	0.24	0.08	0.19	7.80	4.3	3.6		
2013	1.75	0.9	1.15	2.59	1.6	1.12	3.07	0.9		1.51	4.85	1.54	0.7	0.88	0.26	0.09	0.18	7.58	4.5	3.5		
2014	1.81	1	1.11	2.57	1.6	1.08	3.67	1		1.85	4.69	1.60	0.8	0.85	0.29	0.09	0.19	7.26	4.2	3.42		
2015	1.87	1	1.00	2.52	1.6	1.02	4.39	1.2		2.28	8.08	1.66	0.8	0.96	0.32	0.1	0.17	6.87	4.6	3.35	3.78	3.16
2016	1.93	1	1.10	2.47	1.6	1.05	5.26	1.4		2.86	5.39	1.73	0.8	0.98	0.35	0.14	0.20	6.43	4.4	3.28	3.73	3.1
2017	1.99	1.1	1.39	2.40	1.7	1.14	6.27	1.8		3.54	5.74	1.79	0.9	0.99	0.37	0.15	0.20	5.98	3.9	3.2	3.73	3.3
2018	2.04	1.1	1.41	2.33	1.7	1.57	7.44	2		4.09		1.84	0.9	1.03	0.40	0.15	0.21	5.55	3.5	3.1	3.7	3.38

Note: This study-error is derived from the uncertainty analysis in Tables S11-S14.

The reported data from Eurostat, https://ec.europa.eu/eurostat/statistics-explained/index.php/End-of-life_vehicle_statistics#Total_weight_of_end-of-life_vehicles;

*JARC, https://www.jarc.or.jp/renewal/wp-content/uploads/2018/10/mini10-questions-about-automobile-recycling-in-Japan.pdf

References

1. Cabrera Serrenho, A., and Allwood, J.M. (2016). Material Stock Demographics: Cars in Great Britain. Environmental Science & Technology *50*, 3002–3009. 10.1021/acs.est.5b05012.
2. Lewis, G.M., Buchanan, C.A., Jhaveri, K.D., Sullivan, J.L., Kelly, J.C., Das, S., Taub, A.I., and Keoleian, G.A. (2019). Green Principles for Vehicle Lightweighting. Environmental Science & Technology *53*, 4063–4077. 10.1021/acs.est.8b05897.
3. Zeng, X., Ali, S.H., Tian, J., and Li, J. (2020). Mapping anthropogenic mineral generation in China and its implications for a circular economy. Nat Commun *11*, 1544. 10.1038/s41467-020-15246-4.
4. Wu, C., Li, Y., Zhang, Y., Liu, Y., Huang, S., and Ju, Y. (2022). Long-term estimation of plastic material resources from end-of-life vehicles in China: a scenario analysis considering multiple industry standards. J Mater Cycles Waste Manag *24*, 1083–1094. 10.1007/s10163-022-01380-2.
5. Tilton, J.E. (2015). World metal demand: trends and prospects (Routledge).
6. Oguchi, M., and Fuse, M. (2015). Regional and Longitudinal Estimation of Product Lifespan Distribution: A Case Study for Automobiles and a Simplified Estimation Method. Environmental Science & Technology *49*, 1738–1743. 10.1021/es505245q.
7. Bento, A., Roth, K., and Zuo, Y. (2018). Vehicle Lifetime Trends and Scrappage Behavior in the U.S. Used Car Market. The Energy Journal *39*, 159–183. 10.5547/01956574.39.1.aben.
8. Sprung, M.J., Chambers, M., and Smith-Pickel, S. (2018). Transportation Statistics Annual Report 2018. Tech Report United States. Department of Transportation. Bureau of Transportation Statistics. 2018–12–01. https://rosap.ntl.bts.gov/view/dot/37861.
9. Ministry-of-Commerce (2012). Provisions on the Standards for Compulsory Retirement of Motor Vehicles. Department of Treaty and Law, Ministry of Commerce.
10. Chen, M., and Zhang, F. (2009). End-of-Life vehicle recovery in china: Consideration and innovation following the EU ELV directive. JOM *61*, 45–52. 10.1007/s11837-009-0040-8.
11. Yano, J., Muroi, T., and Sakai, S.-i. (2015). Rare earth element recovery potentials from end-of-life hybrid electric vehicle components in 2010–2030. J Mater Cycles Waste Manag, 1–10. 10.1007/s10163-015-0360-4.
12. Hao, H., Wang, H., Ouyang, M., and Cheng, F. (2011). Vehicle survival patterns in China. Science China Technological Sciences *54*, 625–629. 10.1007/s11431-010-4256-1.
13. Xi, H., Yang, Y., and Chen, Y. (2014). Forcasting the near-future obsolescence, increase, and stock of vehicle in China. Highways & Automotive Applications, 30–34.
14. Zhang, A., Tong, X., and Zeng, X. (2020). Recycling potentials of Platinum-group metals from end-of-life vehicle in China. China Environmental Science *40*, 4821–4830.
15. Yano, J., Hirai, Y., Okamoto, K., and Sakai, S.-i. (2014). Dynamic flow analysis of current and future end-of-life vehicles generation and lead content in automobile shredder residue. J Mater Cycles Waste Manag *16*, 52–61. 10.1007/s10163-013-0166-1.
16. Xu, G., Yano, J., and Sakai, S.-i. (2019). Recycling Potentials of Precious Metals from End-of-Life Vehicle Parts by Selective Dismantling. Environmental Science & Technology *53*, 733–742. 10.1021/acs.est.8b04273.
